# Immediate- or Delayed-Intensive Statin in Acute Cerebral Ischemia: The INSPIRES Randomized Clinical Trial

**DOI:** 10.1001/jamaneurol.2024.1433

**Published:** 2024-05-28

**Authors:** Ying Gao, Lingling Jiang, Yuesong Pan, Weiqi Chen, Jing Jing, Chunjuan Wang, S. Claiborne Johnston, Pierre Amarenco, Philip M. Bath, Yingying Yang, Tingting Wang, Shangrong Han, Xia Meng, Jinxi Lin, Xingquan Zhao, Liping Liu, Jinguo Zhao, Ying Li, Yingzhuo Zang, Shuo Zhang, Hongqin Yang, Jianbo Yang, Yuanwei Wang, Dali Li, Yanxia Wang, Dongqi Liu, Guangming Kang, Yongjun Wang, Yilong Wang

**Affiliations:** 1 Department of Neurology, Beijing Tiantan Hospital, Capital Medical University, Beijing, China; 2 China National Clinical Research Center for Neurological Diseases, Beijing, China; 3 Department of Neurology, University of California, San Francisco; 4 Department of Neurology and Stroke Center, Assistance Publique-Hôpitaux de Paris, Bichat Hospital, INSERM LVTS-U1148, University of Paris, Paris, France; 5 Population Health Research Institute, McMaster University, Hamilton, Ontario, Canada; 6 Stroke Trials Unit, Mental Health & Clinical Neuroscience, University of Nottingham, Nottingham, United Kingdom; 7 Department of Neurology, Weihai Wendeng District People’s Hospital, Weihai, China; 8 Department of Neurology, Sui Chinese Medical Hospital, Shangqiu, China; 9 Department of Neurology, Qinghe People’s Hospital, Xingtai, China; 10 Department of Neurology, Biyang People’s Hospital, Zhumadian, China; 11 Department of Neurology, Jiyuan Chinese Medical Hospital, Jiyuan, China; 12 Department of Neurology, The First Affiliated Hospital of Xi’an Jiaotong University, Xi’an, China; 13 Department of Neurology, The Affiliated Shuyang Hospital of Xuzhou Medical University, Shuyang Hospital, Suqian, China; 14 Department of Neurology, Mengzhou People’s Hospital, Jiaozuo, China; 15 Department of Neurology, Hejian People’s Hospital, Cangzhou, China; 16 Department of Neurology, Xiuwu People’s Hospital, Jiaozuo, China; 17 Advanced Innovation Center for Human Brain Protection, Capital Medical University, Beijing, China; 18 National Center for Neurological Disorders, Beijing, China; 19 Research Unit of Artificial Intelligence in Cerebrovascular Disease, Chinese Academy of Medical Sciences, Beijing, China; 20 Chinese Institute for Brain Research, Beijing, China; 21 Beijing Laboratory of Oral Health, Capital Medical University, Beijing, China; 22 Beijing Municipal Key Laboratory of Clinical Epidemiology, Capital Medical University, Beijing, China

## Abstract

**Importance:**

Comparisons are limited for immediate-intensive and delayed-intensive statin for secondary stroke prevention and neuroprotection in patients with acute mild ischemic stroke or transient ischemic attack (TIA) from atherosclerosis.

**Objective:**

To estimate whether immediate-intensive statin therapy is safe and can lower the risk of recurrent stroke compared with delayed-intensive statin in patients with acute mild ischemic stroke or high-risk TIA from atherosclerosis.

**Design, Setting, and Participants:**

The Intensive Statin and Antiplatelet Therapy for High-Risk Intracranial or Extracranial Atherosclerosis (INSPIRES) trial, a double-blind, placebo-controlled, 2 × 2 factorial, randomized clinical trial enrolled patients from September 2018 to October 2022. The trial was conducted at 222 hospitals in China. Patients aged 35 to 80 years with mild ischemic stroke or high-risk TIA of presumed atherosclerosis within 72 hours of symptom onset were assessed.

**Interventions:**

Patients were randomly assigned to receive immediate-intensive atorvastatin (80 mg daily on days 1-21; 40 mg daily on days 22-90) or 3-day delayed treatment (placebo for days 1-3, followed by placebo and atorvastatin, 40 mg daily on days 4-21, and then atorvastatin, 40 mg daily on days 22-90).

**Main Outcomes and Measures:**

The primary efficacy outcome was new stroke within 90 days, and a secondary efficacy outcome was poor functional outcome. Moderate to severe bleeding was the primary safety outcome.

**Results:**

A total of 11 431 patients were assessed for eligibility, and 6100 patients (median [IQR] age, 65 [57-71] years; 3915 men [64.2%]) were enrolled, with 3050 assigned to each treatment group. Within 90 days, new stroke occurred in 245 patients (8.1%) in the immediate-intensive statin group and 256 patients (8.4%) in the delayed group (hazard ratio, 0.95; 95% CI, 0.80-1.13). Poor functional outcome occurred in 299 patients (9.8%) and 348 patients (11.4%) in the immediate-intensive and delayed-intensive statin groups, respectively (odds ratio, 0.83; 95% CI, 0.71-0.98). Moderate to severe bleeding occurred in 23 of 3050 patients (0.8%) and 17 of 3050 patients (0.6%), in the immediate-intensive and delayed-intensive statin groups, respectively.

**Conclusions and Relevance:**

Immediate-intensive statin initiated within 72 hours did not reduce the risk of stroke within 90 days and may be associated with improved functional outcomes without significant difference in moderate to severe bleeding, compared with 3-day delayed-intensive statin in Chinese patients with acute mild ischemic stroke or TIA from atherosclerosis.

**Trial Registration:**

ClinicalTrials.gov Identifier: NCT03635749

## Key Points

**Question:**

Is immediate-intensive statin safe and effective compared with delayed-intensive statin in patients with acute cerebral ischemia from atherosclerosis?

**Findings:**

In this randomized clinical trial of 6100 patients, 8.1% of patients in the immediate-intensive statin group and 8.4% of patients in the delayed group had a new stroke within 90 days without a significant difference between groups.

**Meaning:**

The results do not support that immediate-intensive statin initiated within 72 hours reduces the risk of stroke within 90 days compared with 3-day delayed-intensive statin in patients with acute cerebral ischemia from atherosclerosis, without increasing risk of moderate to severe bleeding.

## Teaser

This randomized clinical trial investigates the safety and effectiveness of immediate- vs delayed-intensive statin in patients with acute cerebral ischemia from atherosclerosis.

## Introduction

Patients with acute ischemic stroke or transient ischemic attack (TIA) are at high risk of recurrent stroke and poor functional outcomes within 3 months.^1,2,3^ High levels of low-density lipoprotein cholesterol (LDL-C) and other atherogenic lipoproteins may contribute to this risk.^4^ The Stroke Prevention by Aggressive Reduction in Cholesterol Levels (SPARCL) and the Treat Stroke to Target (TST) trials indicated that administering intensive statin therapy to lower LDL-C levels in patients with stroke or TIA is effective in reducing the risk of stroke recurrence.^5^ Based on previous studies that focused on the nonacute phase, current guidelines recommend high-intensity statin therapy for secondary prevention in patients with atherosclerotic ischemic stroke.^6,7^ However, guidelines lack clear recommendations for the timing of statin administration in the acute phase, which had been discussed in acute coronary syndromes.^6,7,8^ Results from the Fast Assessment of Stroke and TIA to Prevent Early Recurrence (FASTER) study with small sample size (N = 392) showed no superiority of simvastatin initiated acutely to placebo.^9^ The use of immediate or delayed high-intensity statins within 72 hours of stroke remains controversial, particularly for those patients with acute mild stroke or high-risk TIA of presumed atherosclerotic cause.^10,11,12,13^ Thus, the safety and efficacy of immediate high-intensity statin therapy for reducing early recurrence are still uncertain.

In addition to their LDL-lowering effects, statins have been found to possess various cytoprotective benefits, including protection of endothelial function, antioxidant properties, and anti-inflammatory effects.^14,15^ Preclinical studies have shown that administering statins immediately after a stroke can reduce the size of the infarct and improve neurological outcomes.^16,17^ In humans, the SPARCL trial suggests that atorvastatin, 80 mg per day, may improve functional outcome at 5 years as compared with placebo in patients with recurrent stroke.^3^ Meta-analysis and observational studies have further indicated that early statin treatment is associated with good functional outcome and lower mortality.^10,18,19^ Conversely, discontinuation of statin treatment after admission has been linked to poorer functional outcomes, increased mortality, and dependence.^18,20^ However, there is a lack of high-quality RCTs providing evidence for the neuroprotective effects or for the effective improvement of functional outcomes in patients receiving acute statin treatment.

We conducted the Intensive Statin and Antiplatelet Therapy for High-Risk Intracranial or Extracranial Atherosclerosis (INSPIRES) trial. This study aimed to determine (1) whether immediate-intensive statin therapy initiated within 72 hours of symptom onset is safe and can lower the risk of recurrent stroke compared with delayed therapy in patients with mild ischemic stroke or high-risk TIA and atherosclerosis and (2) whether immediate-intensive statin therapy improves functional outcomes in these patients.

## Methods

### Study Design

The INSPIRES study was a multicenter, double-blind, placebo-controlled, 2 × 2 factorial, randomized clinical trial in which patients at 222 centers in China underwent randomization from September 2018 to October 2022. Results of the intensive statin arm are presented here whereas results of an arm comparing the combination of clopidogrel with aspirin vs aspirin alone will be published elsewhere. Details of the rationale and design of the INSPIRES study have been described previously.^21^ The protocol, statistical analysis plan, and information on committees, sites, and investigators are available in Supplement 1, Supplement 2, and the eAppendix in Supplement 3, respectively. The trial was approved by the ethics committee at Beijing Tiantan Hospital and all other study sites. The complete list of participating centers is listed in Supplement 4. Written informed consent was obtained from the participants or their representatives before enrollment in the trial. This trial followed the Consolidated Standards of Reporting Trials (CONSORT) reporting guidelines.

### Participants

Patients aged between 35 and 80 years without restrictions on ethnicity were eligible for enrollment if they had a mild ischemic stroke with a National Institutes of Health Stroke Scale (NIHSS) score of 5 or lower (range, 0-42, with higher scores indicating more severe stroke), a high-risk TIA with an ABCD2 score (risk score with age, blood pressure, clinical features, duration of TIA, and presence of diabetes) of 4 or higher between 24 and 72 hours after onset (range, 0-7, with higher scores indicating higher risk of stroke), or an ischemic stroke with a NIHSS score of 4 or 5 within 24 hours of ictus. In addition, the patients should meet at least 1 of the following criteria: (1) 50% or higher stenosis of a major intracranial or extracranial artery that likely accounted for the infarction and clinical presentation, confirmed by carotid duplex ultrasound or vascular imaging or (2) acute multiple infarctions of presumed large-artery atherosclerosis origin, including with nonstenotic unstable plaque, documented by computed tomography or magnetic resonance imaging of the head.

Patients were not eligible for the trial if they had a TIA or ischemic stroke of presumed cardioembolic or other determined etiology; if they had moderate to severe prestroke disability defined as a modified Rankin Scale (mRS) score greater than 2; if they received intravenous thrombolysis or endovascular therapy after onset; if they received fibrinogen therapy, anticoagulation therapy, or antiplatelet therapy except for clopidogrel and aspirin after onset; if they received dual antiplatelet therapy with aspirin and clopidogrel or intensive statin therapy within 2 weeks before randomization; or if they had severe hepatic or kidney dysfunction. A complete description of the inclusion and exclusion criteria for the trial is available in the eAppendix of Supplement 3.

### Randomization and Masking

Eligible participants were randomly assigned in a 1:1:1:1 ratio into 4 groups as follows: (1) intensive antiplatelet therapy plus immediate-intensive statin therapy, (2) intensive antiplatelet therapy plus delayed-intensive statin therapy, (3) standard antiplatelet therapy plus immediate-intensive statin therapy, and (4) standard antiplatelet therapy plus delayed-intensive statin therapy. A randomization sequence was computer generated centrally and stratified by participating centers via block randomization, with a block size of 8 with stratification for study sites, at the Statistics and Data Centre at the China National Clinical Research Center for Neurological Diseases (Beijing, China). All the participants and their representatives, investigators, the independent clinical event adjudication committee, and the data safety and monitoring board were masked to treatment allocation. Participants were assigned a random number corresponding to a medication package that was administered to the patient.

### Procedures

Participants in the immediate-intensive statin therapy group received atorvastatin, 80 mg daily, for days 1 to 21, followed by 40 mg daily for days 22 to 90. Participants in the delayed-intensive statin therapy group received an atorvastatin placebo for days 1 to 3, followed by atorvastatin placebo and atorvastatin, 40 mg daily, for days 4 to 21, and then 40 mg daily for days 22 to 90. After the 3-month study therapy, participants received standard care based on the latest guidelines at the discretion of the local investigator, and outcomes were followed up for an additional 9 months with continued information collection, which has yet to undergo analysis. A detailed flowchart of the assessment schedule is provided in the protocol (Supplement 1).

### Outcomes

The primary efficacy outcome was any new stroke (ischemic or hemorrhagic) at 90 days. Secondary efficacy outcomes included composite vascular event (stroke, myocardial infarction [MI], or vascular death), ischemic stroke, TIA, MI, vascular death, and poor functional outcome (mRS score of 2-6) within 3 months. The new stroke or TIA was also measured using a 6-level ordered category scale that incorporated vascular events with mRS score: 6 = death, 5 = fatal stroke (stroke with subsequent death), 4 = severe stroke (stroke followed by mRS score of 4 or 5), 3 = moderate stroke (stroke followed by mRS score of 2 or 3), 2 = mild stroke (stroke followed by mRS score of 0 or 1), 1 = TIA, and 0 = neither stroke nor TIA at 3 months.^22^ Definitions for efficacy outcomes can be found in Supplement 1.

The primary safety outcome was moderate to severe bleeding defined by the standards from the Global Utilization of Streptokinase and Tissue Plasminogen Activator for Occluded Coronary Arteries (GUSTO) criteria.^23^ The secondary safety outcomes included hepatotoxicity (alkaline phosphatase or aspartate aminotransferase level more than 3 times the upper limit of normal range), muscle toxicity (creatine kinase level more than 10 times the upper limit of normal range, presence of muscle pain, myopathy, or rhabdomyolysis), all-cause mortality, intracranial hemorrhage, any bleeding, and additional adverse or severe adverse events within 90 days.

All efficacy and safety outcome events were confirmed by an independent clinical event adjudication committee whose members were unaware of the study group assignments. The committee physicians adjudicated ischemic stroke subtypes, MI, and death according to available medical records, including imaging examinations.

### Sample Size Calculation

The minimal sample size for the trial is determined by the necessity that a clinically meaningful difference in effectiveness between treatment and control groups has to be detected. Based on previous studies, the risk of new stroke during 90 days is presumed to be 11.5% in the group with aspirin (with half delayed-intensive statin therapy and half immediate-intensive statin therapy) and 11.5% in the delayed-intensive statin therapy group (with half aspirin and half dual antiplatelet therapy), and 13% in the group with aspirin plus delayed-intensive statin therapy, dual antiplatelet therapy, and immediate-intensive statin therapy can reduce this risk by 22%. The effects of dual antiplatelet and immediate-intensive statin therapy will be similar and additive.^24,25,26,27,28^ We determined that a total of 6100 participants would provide 80% power to detect a relative risk reduction of 20% in the risk of stroke in the immediate intensive statin group, with a final 2-sided significance level of .05, assuming 5% overall rate of dropouts. The type I error level of the statistical significance was set at a 2-sided α of .05 in the final analysis.

### Statistical Analysis

The main efficacy and safety analyses were based on the intention-to-treat analysis principle. The cumulative risks of the primary outcome of stroke events during the 90-day period were assessed with Kaplan-Meier analyses and the log-rank test. Unadjusted differences between the 2 groups in incidences of stroke within 90 days were estimated by using a Cox proportional-hazards method, with pooled study centers (those with <20 enrolled participants were pooled together) set as a random effect, and hazard ratio (HR) and 95% CI were reported. Assessment of the interaction between the trial group and a logarithmic function of survival time and proportionality was conducted to verify that no evidence against the proportional hazards assumption was found. Participants were reviewed at their last follow-up evaluation when they experienced a clinical event, at the end of the trial, at the time of withdrawal from the trial, or at the last visit if primary outcome data were missing. If there were multiple events of the same type, the time to the first event was used. Similar methods were applied to compare the secondary efficacy outcomes of composite vascular events, ischemic stroke, hemorrhagic stroke, myocardial infarction, TIA, bleeding, and mortality. Shift analysis was conducted for comparison of the secondary outcomes of ordinal stroke or TIA combined with the mRS outcome between 2 treatment groups using ordinal logistic regression, with the proportionality assumption met, and the common odds ratio (OR) and its 95% CI were calculated. Poor functional outcome, hepatotoxicity, and muscle toxicity were compared using binary logistic regression with pooled study centers set as a random effect, and the OR and its 95% CI were calculated. Because of the low rates of mortality in each trial group, we did not conduct a competing risk analysis. Other adverse events and serious adverse events were compared using the χ^2^ test or Fisher exact test. Interactions between treatment assignment and prespecified subgroups for the primary outcome were estimated by including terms for treatment, subgroup, and treatment-by-subgroup interaction in the Cox model.

The statistical analysis plan did not include a provision for correcting for multiplicity when performing tests for secondary or other outcomes; results are reported as point estimates and 95% CIs. The widths of the CIs have not been adjusted for multiplicity; therefore, the intervals should not be used to infer definitive treatment effects for secondary outcomes. All statistical analyses were performed with SAS software, version 9.4 (SAS Institute Inc).

## Results

### Trial Population

**Figure 1.**
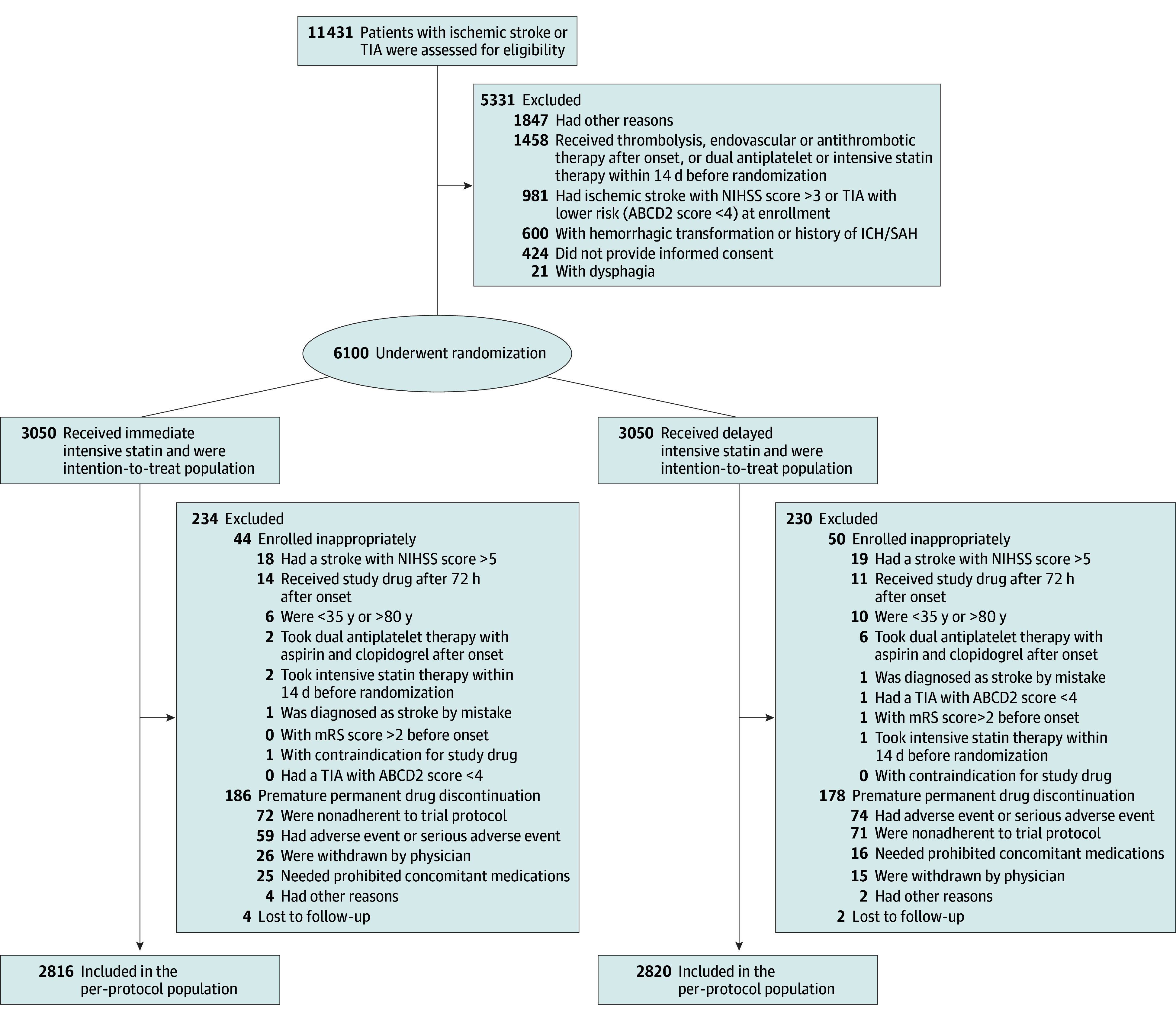
Enrollment and Randomization of Patients Patients who were enrolled inappropriately or discontinued trial drug were included in the intention-to-treat analysis, as were patients who died of a cause other than stroke or were lost to follow-up. The ABCD2 score assess the risk of stroke on the basis of age, blood pressure, clinical features, duration of transient ischemic attack (TIA), and presence of diabetes (range, 0-7, higher scores indicating higher risk of stroke) ICH indicates intracerebral hemorrhage; mRS, modified Rankin Scale; NIHSS, National Institutes of Health Stroke Scale; SAH, subarachnoid hemorrhage.

From September 17, 2018, through October 15, 2022, a total of 11 431 patients with ischemic stroke or TIA were assessed for eligibility, and 6100 (53.4%; median [IQR] age, 65 [57-71] years; 3915 men [64.2%]; 2185 women [35.8%]; 6100 Asian race [100%]; 6011 Han Chinese ethnicity [98.5%]) underwent randomization at 222 clinical sites; 3050 were randomly assigned to the immediate-intensive statin therapy group and 3050 to the delayed-intensive statin therapy group. Half of the participants in each group received clopidogrel-aspirin and half received aspirin alone. During the trial, there were instances of premature permanent discontinuation in 364 participants (5.9%), and 16 patients in the immediate-intensive statin therapy group and 23 patients in the delayed-intensive statin therapy group died of causes other than stroke. A total of 4 participants in the immediate-intensive statin group and 2 participants in the delayed-intensive statin group were lost to follow-up at 90 days; 7 participants had missing data regarding disability (Figure 1).

The baseline characteristics and concomitant treatment within 90 days were well balanced in the 2 groups (Table 1 and eTables 1 and 2 in Supplement 3). Most participants (5299 [86.9%]) presented with ischemic stroke, and 801 (13.1%) presented with TIA. Among participants with acute ischemic stroke, the median (IQR) NIHSS score was 2 (1-3) in the immediate-statin group and 2 (1-3) in the delayed-statin group. The median (IQR) time from symptom onset to randomization was 46.3 (29.4-56.5) hours and 46.4 (30.0-56.0) hours in the immediate- and delayed-intensive statin groups, respectively.

**Table 1.** Baseline Characteristics of the Patients

Characteristic	Immediate-intensive statin (n = 3050)	Delayed-intensive statin (n = 3050)
Age, median (IQR), y	65 (57-71)	65 (57-71)
Sex, No. (%)		
Women	1056 (34.6)	1129 (37.0)
Men	1994 (65.4)	1921 (63.0)
Body mass index, median (IQR)^a^	24.5 (22.6-26.7)	24.4 (22.6-26.6)
Medical history, No. (%)		
Hypertension	2044 (67.0)	2039 (66.9)
Diabetes	813 (26.7)	845 (27.7)
Dyslipidemia	115 (3.8)	111 (3.6)
Previous ischemic stroke	927 (30.4)	882 (28.9)
Previous TIA	47 (1.5)	50 (1.6)
Previous myocardial infarction	53 (1.7)	60 (2.0)
Current smoker, No. (%)	876 (28.7)	907 (29.7)
Application of agents before events, No. (%)^b^		
Lipid-lowering agents	302 (9.9)	285 (9.3)
Aspirin	403 (13.2)	390 (12.8)
Clopidogrel	22 (0.7)	21 (0.7)
Qualifying event, No. (%)		
TIA	429 (14.1)	372 (12.2)
Acute single ischemic infarction	583 (19.1)	591 (19.4)
Acute multiple ischemic infarctions	2038 (66.8)	2087 (68.4)
With ≥50% symptomatic stenosis, No./total No. (%)^c^		
Yes	2446/2979 (82.1)	2469/2989 (82.6)
No	533/2979 (17.9)	520/2989 (17.4)
NIHSS in qualifying ischemic stroke, No./total No. (%)^d^		
≤3	2000/2621 (76.3)	2033/2678 (75.9)
>3	621/2621 (23.7)	645/2678 (24.1)
ABCD2 score among patients with TIA, No./total No. (%)^e^		
≤5	339/429 (79.0)	302/372 (81.2)
>5	90/429 (21.0)	70/372 (18.8)
LDL-C level at baseline, mean (SD), mmol/L	2.56 (0.78)	2.67 (0.79)
LDL-C level at 90 d, mean (SD), mmol/L	1.98 (0.66)	1.99 (0.70)
Clopidogrel-aspirin assignment, No. (%)	1525 (50)	1525 (50)

Abbreviations: LDL-C, low-density lipoprotein cholesterol; NIHSS, National Institutes of Health Stroke Scale; TIA, transient ischemic attack.

SI conversion factor: To convert LDL-C to milligrams per deciliter, divide by 0.0259.

^a^ Calculated as weight in kilograms divided by height in meters squared.

^b^ Patients received medication within 1 month before symptom onset.

^c^ Data were missing in 132 cases due to the absence of both intracranial and extracranial arterial vascular assessments, or participants did not have more than 50% stenosis in intracranial (or extracranial) arteries but were missing in extracranial (or intracranial) vascular assessments.

^d^ Scores on the NIHSS range from 0 to 42 for patients with ischemic stroke, with higher scores indicating more severe stroke.

^e^ The ABCD2 score assesses the risk of stroke on the basis of age, blood pressure, clinical features, duration of TIA, and the presence or absence of diabetes mellitus for patients with transient ischemic attack, with scores ranging from 0 to 7 and higher scores indicating greater risk.

### Primary Outcome

**Figure 2.**
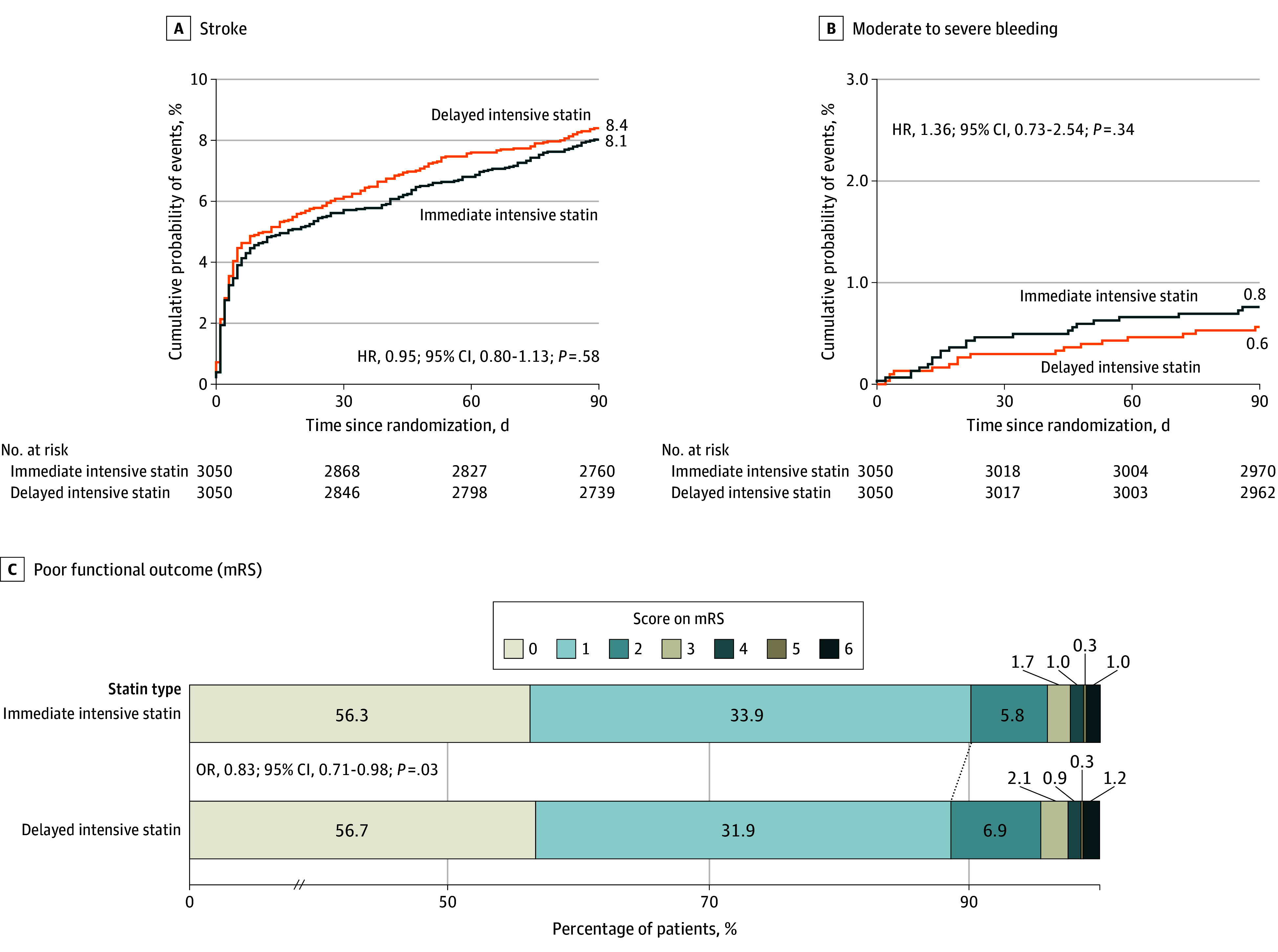
Cumulative Probability of Stroke (Primary Efficacy Outcome) and Moderate to Severe Bleeding and Distribution of Modified Rankin Scale (mRS) Score A, The probability of ischemic or hemorrhagic stroke. B, The probability of moderate to severe bleeding. C, The distribution of mRS score. HR indicates hazard ratio.

**Figure 3.**
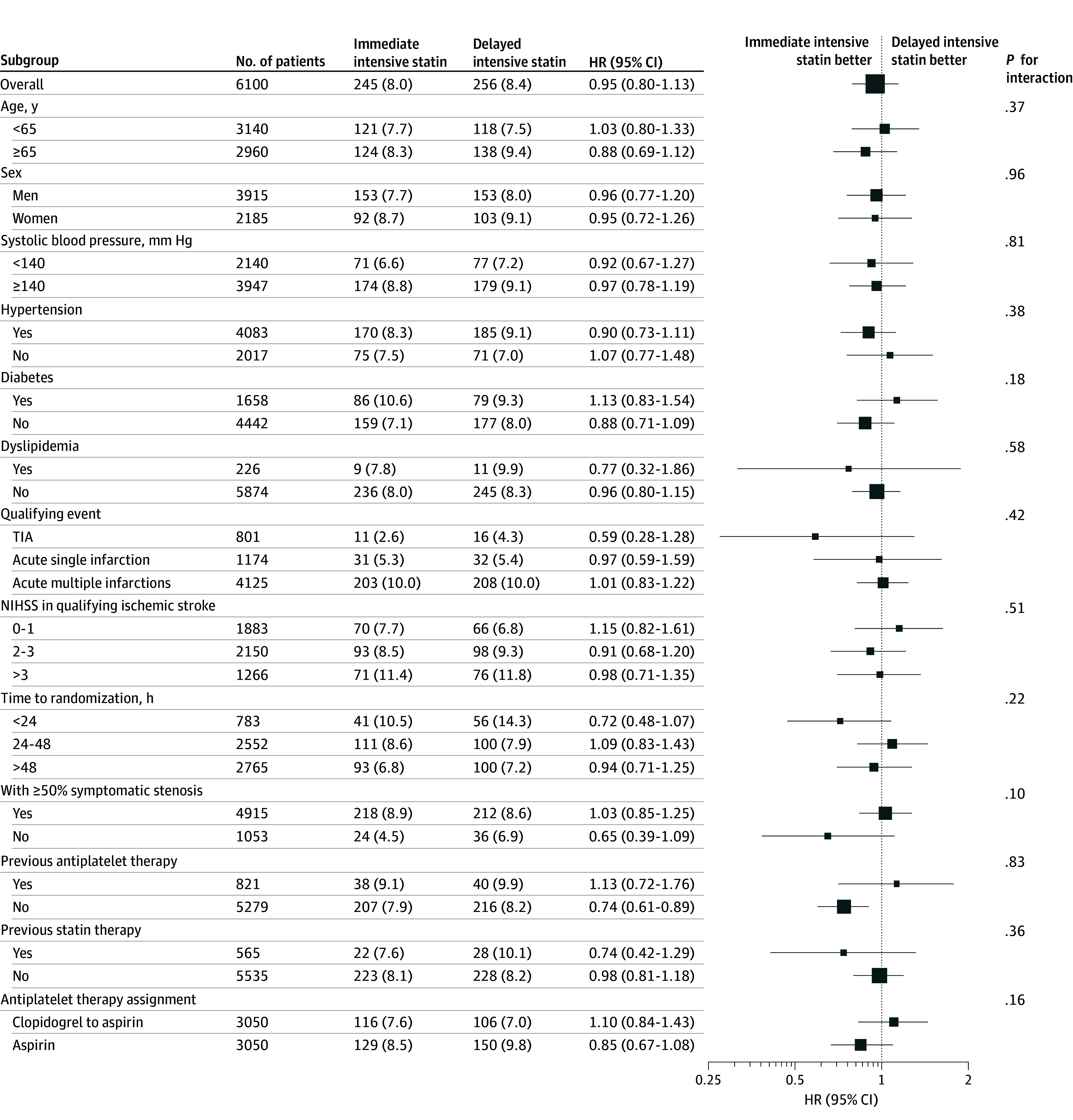
Hazard Ratio (HR) for the Stroke According to Prespecified Subgroups The trial was not powered to allow definite conclusions based on the results of the subgroup analyses. Systolic blood pressure data were missing in 8 patients in the immediate statin group and 5 patients in the delayed statin group. Data on 50% or greater symptomatic stenosis were missing in 71 patients in the immediate statin group and 61 patients in the delayed statin group. NIHSS indicates National Institutes of Health Stroke Scale.

The primary efficacy outcome, new stroke within 90 days, occurred in 245 of 3050 participants (8.1%) in the immediate-intensive statin group and in 256 of 3050 participants (8.4%) in the delayed-intensive statin group (HR, 0.95; 95% CI, 0.80-1.13; *P* = .58) (Figure 2A and Table 2). The trial center effect was nonnegligible (eFigure 3 in Supplement 3). The results of the per-protocol analysis of efficacy were consistent with those of the primary intention-to-treat analysis (eTable 3 in Supplement 3). There was no significant statin treatment by antiplatelet treatment assignment interaction on stroke (*P* value for interaction = .16). Analysis of predefined subgroups for the primary outcome is shown in Figure 3 and eFigure 1 in Supplement 3.

**Table 2.** Efficacy and Safety Outcomes

Outcome	Immediate-intensive statin (n = 3050)		Delayed-intensive statin (n = 3050)		Treatment effect (95% CI)^b^	*P* value
	Patients with event, No.	Event rate, %^a^	Patients with event, No.	Event rate, %^a^		
Primary outcome (full analysis set)						
Stroke (including ischemic and hemorrhagic stroke)	245	8.1	256	8.4	0.95 (0.80 to 1.13)	.58
Secondary outcomes (full analysis set)						
Composite vascular event (stroke, myocardial infarction, or vascular death)	251	8.2	260	8.6	0.96 (0.81 to 1.14)	.64
Ischemic stroke	235	7.7	247	8.1	0.95 (0.79 to 1.13)	.55
Recurrent stroke	173	5.8	191	6.4	0.90 (0.73 to 1.11)	.31
TIA with infarctions	10	0.3	6	0.2	1.65 (0.60 to 4.55)	.33
Progressive stroke	52	1.7	50	1.7	1.04 (0.71 to 1.54)	.84
Hemorrhagic stroke	11	0.4	9	0.3	1.22 (0.51 to 2.95)	.66
TIA	29	1.0	31	1.0	0.94 (0.56 to 1.55)	.79
Myocardial infarction	4	0.1	3	0.1	1.33 (0.30 to 5.95)	.71
Vascular death	18	0.6	18	0.6	1.00 (0.52 to 1.92)	>.99
Poor functional outcome (mRS 2-6), No./total No.^c^	299/3047	9.8	348/3046	11.4	0.83 (0.71 to 0.98)	.03
Early neurological deterioration (the change of NIHSS score at 7 d), median (IQR)^d^	0 (−1 to 0)		0 (−1 to 0)		−0.02 (−0.12 to 0.08)	.70
Ordinal stroke or TIA^e^					0.95 (0.80 to 1.13)	.59
Fatal stroke: score of 6 on mRS, No./total No.	16/3049	0.5	17/3049	0.6	NA	NA
Severe stroke: score of 4 or 5 on mRS, No./total No.	30/3049	1.0	25/3049	0.8	NA	NA
Moderate stroke: score of 2 or 3 on mRS, No./total No.	71/3049	2.3	100/3049	3.3	NA	NA
Mild stroke: score of 0 or 1 on mRS, No./total No.	127/3049	4.2	113/3049	3.7	NA	NA
TIA, No./total No.	28/3049	0.9	28/3049	0.9	NA	NA
No stroke or TIA, No./total No.	2777/3049	91.1	2766/3049	90.7	NA	NA
Primary safety outcomes (full analysis set)						
Moderate to severe bleeding^f^	23	0.8	17	0.6	1.36 (0.73 to 2.54)	.34
Secondary safety outcomes (full analysis set)						
Hepatotoxicity^g^	39	1.3	32	1.1	1.22 (0.76 to 1.95)	.41
Muscle toxicity^h^	2	0.07	1	0.03	2.00 (0.18 to 22.09)	.57
All-cause mortality	30	1.0	37	1.2	0.81 (0.50 to 1.31)	.39
Any bleeding^f^	77	2.5	80	2.6	0.95 (0.69 to 1.30)	.74
Intracranial hemorrhage	14	0.5	11	0.4	1.27 (0.58 to 2.81)	.55
Mild bleeding	58	1.9	63	2.1	0.90 (0.63 to 1.29)	.58

Abbreviations: mRS, modified Rankin Scale; NA, not applicable; NIHSS, National Institutes of Health Stroke Scale; TIA, transient ischemic attack.

^a^ The event rates of poor functional outcome (mRS 2-6), ordinal stroke or TIA, hepatotoxicity, and muscle toxicity are raw estimates, whereas the rates of other outcomes are Kaplan-Meier estimates of the percentage of patients with events at 90 days.

^b^ The odds ratios are shown for poor functional outcome (mRS 2-6), hepatotoxicity, and muscle toxicity. The common odds ratio is shown for ordinal stroke or TIA. β coefficient is shown for Early neurological deterioration (the change of NIHSS score at 7 days). Hazard ratios are shown for other outcomes. The widths of the CIs for secondary outcomes were not adjusted for multiplicity and may not be used for hypothesis testing.

^c^ mRS scores range from 0 to 6, with 0 indicating no symptoms; 1, symptoms without clinically significant disability; 2, slight disability; 3, moderate disability; 4, moderately severe disability; 5, severe disability; and 6, death. The mRS score data at 90 days were missing in 3 patients in the immediate-intensive statin group and 4 patients in the delayed-intensive statin group.

^d^ The change of NIHSS score at 7 days was analyzed by generalized linear models with continuous variables.

^e^ The severity of stroke or TIA is classified on a 6-level ordered categorical scale of combined vascular events with mRS. The mRS score at 90 days was missing in 1 patient with new stroke each in the immediate- and delayed-intensive statin groups.

^f^ Bleeding events were defined according to the Global Utilization of Streptokinase and Tissue Plasminogen Activator for Occluded Coronary Arteries criteria.

^g^ Hepatotoxicity was defined as alkaline phosphatase or aspartate aminotransferase level greater than 3 times the upper limit of normal value.

^h^ Muscle toxicity was defined as creatine kinase level greater than 10 times the upper limit of normal value, or presence of muscle pain, myopathy, or rhabdomyolysis.

### Secondary Outcomes

The secondary efficacy outcome of poor functional outcome at 90 days occurred in 299 participants (9.8%) in the immediate-intensive statin group and in 348 participants (11.4%) in the delayed-intensive statin group (OR, 0.83; 95% CI, 0.71-0.98) (Figure 2C, Table 2, and eFigure 2 in Supplement 3). More sensitivity analyses are shown in eTable 7 in Supplement 3. Composite vascular events within 90 days occurred in 251 participants (8.2%) in the immediate-intensive statin group and in 260 participants (8.6%) in the delayed-intensive statin group. Ischemic stroke occurred in 235 participants (7.7%) in the immediate-intensive statin group and in 247 participants (8.1%) in the delayed-intensive statin group. Other secondary end points are presented in Table 2. As for the analysis of prespecified subgroups for the poor functional outcome, there were significant interaction effects with statin treatment × diabetes (*P* value for interaction = .01), indicating possible treatment effect in participants without diabetes (eFigure 2 in Supplement 3).

### Adverse Events

The primary safety outcome, moderate to severe bleeding defined by the GUSTO criteria, occurred in 23 participants (0.8%) in the immediate-intensive statin group and 17 participants (0.6%) in delayed-intensive statin group (HR, 1.36; 95% CI, 0.73-2.54; *P* = .34) (Figure 2B and Table 2). The rate of secondary safety outcomes was similar across the 2 groups, including hepatotoxicity, muscle toxicity, all-cause mortality (Table 2), other adverse events (eTable 4 in Supplement 3), and severe adverse events (eTables 5 and 6 in Supplement 3). The overall adverse event occurred in 693 participants (22.7%) in the immediate-intensive statin group and 605 participants (19.8%) in the delayed-intensive statin group (*P* = .006) (eTable 4 in Supplement 3). The results of the per-protocol analysis of safety were consistent with those of the primary intention-to-treat analysis (eTable 3 in Supplement 3).

## Discussion

In this doubled-blind, placebo-controlled, randomized clinical trial in Chinese patients with mild ischemic stroke or high-risk TIA presumed to be caused by atherosclerosis, immediate-intensive statin therapy showed no significant difference compared with delayed-intensive statin therapy in reducing the risk of stroke. However, secondary analysis suggested that immediate treatment may be associated with reduced risk of poor functional outcome at 90 days, compared with delayed-intensive statin therapy. There were no substantial differences observed in the incidence of adverse events, such as bleeding, hepatotoxicity, or muscle toxicity, between the 2 groups.

Currently, guidelines recommend high-intensity statins for secondary prevention for patients with ischemic stroke.^7^ The mechanisms of improving outcomes by statins after ischemic events may include LDL-C lowering, neuroprotective effects, and enhancing endothelial function.^29,30^ However, evidence for the effect of statins in reducing stroke recurrence after immediate administration in patients with acute ischemic stroke remains unclear, particularly in the acute phase (within 72 hours). Previous trials did not focus on this critical period,^5,31^ and recent randomized clinical trials did not show a significant effect on reducing the risk of new stroke.^11,12^ However, retrospective analyses and meta-analyses have suggested a positive effect on survival.^32,33^ In this large-scale randomized controlled trial, we did not find significant difference of the incidence of new stroke between immediate-intensive statin and delayed-intensive statin group within 72 hours of stroke onset, which is similar to the results of the Effects of Very Early Use of Rosuvastatin in Preventing Recurrence of Ischemic Stroke (EUREKA) study.^12^

Previous animal experiments and randomized clinical trials indicated contradictory results of statin administration after stroke.^34^ Our study demonstrated that immediate-intensive statin therapy was marginally associated with improved functional outcomes at 90 days, different from the results of the Stroke Treatment With Acute Reperfusion and Simvastatin (STARS) and Administration of Statin on Acute Ischemic Stroke Patient (ASSORT) trials.^11,13^ Moreover, patients without a history of diabetes or dyslipidemia might receive the pronounced clinical benefit from immediate-intensive statin with better functional outcome. The discrepant results may be attributed to the differences in sample sizes of each trial, stroke etiology differences, and inadequate doses of statins in previous studies. Our study provides some evidence of the benefits of early intensive statin therapy for patients with acute ischemic stroke, but results will need to be repeated in a trial focused on functional outcome as a primary outcome.

Concerns have been raised about whether statins increase the risk of bleeding, especially in patients with prior intracerebral hemorrhage.^35,36,37,38^ Previous studies have produced conflicting results on this matter. The SPARCL trial and the Heart Prevention Study reported an increased risk of bleeding with statin treatment in patients with prior stroke, whereas other studies have found no evidence of such a risk.^35,39,40,41^ We found that immediate-intensive statin therapy did not increase the risk of bleeding within 90 days and appeared to be safe in the short term. This study did not provide evidence of increased risk of bleeding within 90 days in immediate-intensive statin therapy group.

### Limitations

One of the major limitations was that the population studied (mainly Han Chinese patients) may imply differences in prior and concurrent exposures and ethnocultural practices as compared with other populations, with low statin use at baseline. There may be limitation of potential underpower for interaction within the 2 × 2 design. Thus, we might necessarily assume no interaction here between antiplatelet therapy and lipid-lowering therapy. The large number of statistical tests in this study, lack of control for multiple hypothesis tests, small sample size in subgroups, lack of generalizability, low proportion (36%) of women enrolled, and site-to-site variability also need to be considered as potential limitations. Furthermore, we did not have enough power for the secondary outcome of functional outcome. With a negative primary outcome, the results of the secondary outcome (functional outcome) must be evaluated as hypothesis generating.

## Conclusions

In conclusion, this randomized clinical trial in Chinese patients with acute mild ischemic stroke or high-risk TIA from atherosclerosis revealed that immediate-intensive statin therapy administered within 72 hours after symptom onset did not significantly reduce the risk of subsequent stroke as compared with delayed-intensive statin therapy within 90 days. However, immediate-intensive statin therapy may be related to improved functional outcomes with no increase in risk of bleeding at 90 days as compared with delayed therapy.

## Notes

Supplement 1.
Trial Protocol.

Supplement 2.
Statistical Analysis Plan.

Supplement 3.
eAppendix.
**eFigure 1.** Hazard Ratio for Stroke in Prespecified Subgroups **eFigure 2.** Odds Ratio for Poor Functional Outcome in Prespecified Subgroups **eFigure 3.** Subgroup Analysis for Stroke by Center **eTable 1.** Baseline Characteristics of the Patients **eTable 2.** Concomitant Treatment Within 90 Days **eTable 3.** Efficacy and Safety Outcomes in Per-Protocol Population **eTable 4.** Number of Patients With Adverse Events by System Organ Class (Excluding Strokes) Up to 3-Month Visit **eTable 5.** Number of Patients With Serious Adverse Events By System Organ Class (Excluding Strokes) Up To 3-Month Visit **eTable 6.** Number of Patients With Adverse Events or Serious Adverse Events Leading to Premature Permanent Drug Discontinuation by System Organ Class Up to 3-Month Visit **eTable 7.** mRS Outcome Adjusted by the Baseline Proportion of TIA and Infarction and Baseline NIHSS Score

Supplement 4. **Nonauthor Collaborators.** INSPIRES Investigators

Supplement 5.
Data Sharing Statement.
