# Supplementary material for: Immediate- or Delayed-Intensive Statin in Acute Cerebral Ischemia: The INSPIRES Randomized Clinical Trial
Source: JAMA Neurol. 2024 May 28;81(7):741–51. doi: 10.1001/jamaneurol.2024.1433 (PMC11134282; doi:10.1001/jamaneurol.2024.1433)
Supplement: Supplement 1. — Trial Protocol. [file jamaneurol-e241433-s001.pdf]

Supplement 1

Protocol for: Clopidogrel and Aspirin in Acute Cerebral Ischemia from Atherosclerosis.

This trial protocol has been provided by the authors to give readers additional information about the work

This supplement contains the following items:

No.	Item	Version	Date	Page No.
1	Original protocol *	V2.0	Apr. 28th, 2018	2 to 79
2	Final protocol	V4.0	Jun. 6th, 2020	80 to 159
3	Summary of protocol amendments	-	-	160 to 163

*The protocol (Version 2.0) was drafted and approved by the ethics committees before the trial began.

Intensive Statin and Antiplatelet Therapy for High-risk Intracranial or Extracranial Atherosclerosis (INSPIRES)

Study Protocol

Principal Investigator: Yilong Wang, Yongjun Wang

Beijing Tiantan Hospital, Capital Medical University, Beijing, China

Protocol Version: 2.0

Apr. 28th, 2018

Table of Contents

Summary of The Trial Protocol 4
INSPIRES Abbreviated Vocabulary 11
1. Study Background 13
2. Study Purposes 20
3. Study Design 24
4. Study Outcomes： 26
5. Subjects Selection 27
6. Therapeutic Regimen 29
7. Study Implementation 30
8. Blood Sample Collection 36
9. Data Collection 37
10. Study Procedure 38
11. Ethics and Regulations 45
12. Research Organization 47
13. Quality Control and Quality Assurance 49
14. Data Preservation 50
15. Safety Monitoring of Data 51
16. Statistical Processing 52
17. Confidentiality and Announcement of Results 57
Reference: 58
Appendix 1. The Definition of Cardiac-cerebral Vascular Events 63
Appendix 2. Evaluation Method of Modified Rankin Scale 66
Appendix 3. NIHSS Score 68
Appendix 4. $ABCD^2$ Score 75
Appendix5. EQ-5D Scale 76
Appendix 6. GUSTO Bleeding Criteria 78
Appendix 7. Clinical Crucial Laboratory Abnormalities 79

Summary of The Trial Protocol

**Study title**	**Intensive Statin and Antiplatelet Therapy for High-risk Intracranial or Extracranial Atherosclerosis**
**Study purposes**	**Major purposes:**<br>**1.** To evaluate the efficacy and safety of intensive antiplatelet therapy versus standard antiplatelet therapy in reducing the risk of new stroke at 90 days in acute mild ischemic stroke or high-risk transient ischemic attack (TIA) patients attributed to extracranial or intracranial atherosclerosis.<br>**2.** To evaluate the efficacy and safety of immediate intensive statin therapy (atorvastatin 80mg/d) versus delayed intensive statin therapy (atorvastatin 40mg/d) in reducing the risk of new stroke at 90 days in acute mild ischemic stroke or high-risk TIA patients attributed to extracranial or intracranial atherosclerosis.<br>**3.** To evaluate the efficacy and safety of intensive antiplatelet combined with immediate intensive statin therapy (atorvastatin 80mg/d) versus standard antiplatelet combined with delayed intensive statin therapy (atorvastatin 40mg/d) in reducing the risk of new stroke at 90 days in acute mild ischemic stroke or high-risk TIA patients attributed to extracranial or intracranial atherosclerosis.<br>**Notes:**<br>• Intensive antiplatelet therapy refers to dual antiplatelet therapy with aspirin and clopidogrel;<br>• Standard antiplatelet therapy refers to antiplatelet monotherapy with aspirin;<br>• Immediate intensive statin therapy (atorvastatin 80mg/d) refers to lipid-lowering therapy since randomization with atorvastatin at a dose of 80 mg daily for the first 21 days, followed by 40 mg daily for days 22-90;<br>• Delayed intensive statin therapy (atorvastatin 40mg/d) refers to lipid-lowering therapy with atorvastatin at a dose of 40 mg daily from the 4th day after randomization to the 90th day;<br>• Acute mild ischemic stroke or high-risk TIA patients attributed to extracranial or intracranial atherosclerosis: acute mild stroke (NIHSS score≤5) or high-risk TIA ($ABCD^2$ score≥4) within 72 hours of onset meeting at least one of the following characteristics: 1) TIA with ≥50% stenosis of a major intracranial or extracranial artery that likely accounts for the clinical presentation; 2) Acute single cerebral infarction with ≥50% stenosis of a major intracranial or extracranial artery that likely accounts for the infarction and clinical presentation; 3)Acute multiple cerebral infarctions with stenosis of extracranial or intracranial arteries (stenosis degree unlimited) that likely accounts for the presentation.
**Study design**	Randomized, double-blind, placebo-controlled, multicenter, 2×2 factorial designed clinical trial.
**Total number of cases**	6100 cases
**Subjects**	Inclusion Criteria

**selection**	1. Age :35-80 years.<br>2. Less than 72 hours from the onset;<br>3. At least one of the followings (a-c)<br>a) High-risk TIA (ABCD2 ≥ 4) with ≥50% stenosis of a major intracranial or extracranial artery that likely accounts for clinical presentation.<br>b) Acute single cerebral infarction (NIHSS≤5) with ≥50% stenosis of a major intracranial or extracranial artery that likely accounts for the infarction and clinical presentation.<br>c) Acute multiple cerebral infarction (NIHSS≤5) with stenosis of extracranial or intracranial arteries (stenosis degree unlimited) that likely accounts for the presentation.<br>4. Written informed consent.<br>**Notes:**<br>• Intracranial large arteries include the intracranial portion of internal carotid arteries, middle cerebral arteries (M1/M2), anterior cerebral arteries (A1/A2), posterior cerebral arteries (P1/P2), intracranial portion of vertebral arteries, and the basilar artery. [1,2];The degree of intracranial artery stenosis is defined on magnetic resonance angiography (MRA), computerized tomography angiography (CTA) or digital substraction angiography (DSA) by criteria from the Warfarin–Aspirin Symptomatic Intracranial Disease (WASID) study [2];<br>• Extracranial large arteries include the extracranial portion of carotid arteries and vertebral arteries (including the portion from initiation of carotid arteries and vertebral arteries to the start of intracranial section, except the subclavian artery, aortic arch and brachiocephalic trunk). The degree of extracranial arteries stenosis is defined on carotid duplex ultrasound, CTA, CE-MRA or DSA by criteria from the North American Symptomatic Carotid Endarterectomy Trial (NASCET) [3];<br>• Acute ischemic stroke with multiple infarction lesions refers to more than one lesion appearing in different locations (separated in space or non-continuous on contiguous slices) on diffusion DWI/ADC imaging, however, it will be considered as the same one if the lesion is continuous on the adjacent levels[4];<br>• TIA: Sudden neurologic deficit lasting less than 24 hours, without non-ischemic causes.<br>• TIA patients with acute multiple cerebral infarctions will also be enrolled.
	**Exclusion criteria**<br>1. Presumed cardioembolic stroke or TIA (e.g. atrial fibrillation, heart valve prosthesis, atrial myxoma, endocarditis, etc.).<br>2. Other determined etiology of stroke or TIA (e.g. aortic dissection, cervico-cerebral artery dissection, vasculitis, vascular malformation, moyamoya disease/syndrome, fibromuscular dysplasia, etc.).<br>3. Non-vascular neurological diseases (e.g. intracranial tumor, multiple sclerosis, etc.).<br>4. Index infarction affects ≥ 50% of a cerebral lobe (e.g. parietal, frontal, occipital);<br>5. Hemorrhagic transformation after onset.<br>6. Contraindications to clopidogrel, aspirin or atorvastatin:<br>a) History of hypersensitivity;

b) Severe heart failure (NYHA classification: III- IV) or asthma;

c) Coagulation disorder or systemic bleeding;

d) History of drug-induced hematologic or hepatic abnormalities;

e) Leukopenia (< 2×10^9/L) or thrombocytopenia (<100×10^9/L);

f) Active liver disease;

g) Pregnancy or lactation period.

***Severe heart failure：** New York Heart Association (NYHA) Classification III and IV

I :Cardiac disease, but no symptoms and no limitation in ordinary physical activity, e.g. no shortness of breath when walking, climbing stairs etc.

II (mild heart failure): Mild symptoms (mild shortness of breath and/or angina) and slight limitation during ordinary activity.

III (moderate heart failure): Marked limitation in activity due to symptoms, even during less-than-ordinary activity, e.g. walking short distances (20–100 m). Comfortable only at rest.

IV (Severe heart failure): Severe limitations. Experiences symptoms even while at rest. Mostly bedbound patients.

7. Pre-existing disability with modified Rankin Scale (mRS) score >2.
8. Intra-arterial or intravenous thrombolysis or endovascular therapy after onset.
9. Defibrinogen therapy (e.g. defibrase and lumbrokinase) after onset.
10. Creatine kinase > 5 times the upper limit of normal value of onset.
11. Drug use related to statin metabolism within 14 days before randomization (e.g. immune-suppressive drugs, antifungal agents, fibrates).
12. Severe hepatic insufficiency (alanine transaminase or aspartate transaminase > 2 times the upper limit of normal value) or renal insufficiency (creatinine > 1.5 times the upper limit of normal value or glomerular filtration rate < 40 ml/min/1.73 m^2).
13. Dual antiplatelet therapy with aspirin and clopidogrel within 14 days before randomization (Patients who started aspirin plus clopidogrel without loading dose(300mg) of clopidogrel after onset are not excluded from the trial.).
14. High-intensity statin therapy within 14 days before randomization (e.g. atorvastatin ≥ 40mg/d, rosuvastatin ≥ 20mg/d).
15. History of intracranial hemorrhage (e.g. intracerebral or subarachnoid hemorrhage).
16. Gastrointestinal bleeding or major surgery within 90 days.
17. History of intracranial or extracranial angioplasty.
18. Planned long-term use of antiplatelet drugs or non-steroidal anti-inflammatory drugs except for study drugs.
19. Planned surgery or revascularization that may need to stop taking the study drugs within the next 90 days.
20. Anticipated life expectancy < 90 days.
21. Currently participating in any other investigational drug or device study.

	22. Unable to complete the follow-up (e.g. dementia, alcoholism, substance abuse, severe mental disease).
**Therapeutic regimen**	Patients who meet the requirements are randomly assigned to one of the following four groups according to the ratio of 1:1:1:1:<br>A: Intensive antiplatelet therapy + immediate intensive statin therapy (atorvastatin 80mg/d)<br>B: Intensive antiplatelet therapy + delayed intensive statin therapy (atorvastatin 40mg/d)<br>C: Standard antiplatelet therapy + immediate intensive statin therapy (atorvastatin 80mg/d)<br>D: Standard antiplatelet therapy + delayed intensive statin therapy (atorvastatin 40mg/d)<br>**Intensive antiplatelet therapy：**<br>Day 1： Clopidogrel 300mg/day+asprin100-300mg/ day<br>Day2 - Day21±2: Clopidogrel 75mg/day+ aspirin 100mg/day<br>Day22±2 -Day 90: Clopidogrel 75mg/day+ aspirin placebo<br>**Standard antiplatelet therapy：**<br>Day 1： Aspirin 100-300mg/day + clopidogrel placebo<br>Day 2 - 90： Aspirin 100mg/day+ clopidogrel placebo<br>**Immediate intensive statin therapy (atorvastatin 80mg/d)：**<br>Day 1 -Day 21±2： Atorvastatin 80mg/day<br>Day22±2 -Day 90： Atorvastatin 40mg/day<br>**Delayed intensive statin therapy (atorvastatin 40mg/d)：**<br>Day 1 - Day3： Atorvastatin placebo<br>Day4 - Day21±2： Atorvastatin 40mg/day + Atorvastatin calcium placebo<br>Day22±2 -Day 90： Atorvastatin 40mg/day
**Study Outcomes**	**Primary outcome:**<br>Stroke (ischemic or hemorrhagic)<br>**Secondary outcomes:**<br>• Composite vascular events： stroke (ischemic or hemorrhagic), myocardial infarction, or cardiovascular death.<br>• Ischemic stroke<br>• TIA<br>• Severity of stroke or TIA on an ordinal scale (a six-level ordered category scale combined vascular events with mRS score: fatal stroke (stroke with subsequent death), severe stroke (stroke followed by mRS of 4-5), moderate stroke (stroke followed by mRS of 2-3), mild stroke (stroke followed by mRS of 0-1), TIA, and no stroke/TIA)<br>• Myocardial infarction

	• Vascular death<br>• All-cause death<br>• Poor functional outcome (mRS score 2-6)<br>• Poor quality of life (EuroQoL-5 Dimensions index score≤0.5)<br>• Early neurological deficits (increase in NIHSS score at 7days)
	**Primary safety outcomes:**<br>Moderate to severe bleeding (Appendix 6)<br>**Secondary safety outcomes:**<br>• Intracranial hemorrhage<br>• Hepatotoxicity： alanine transaminase or aspartate transaminase > 3 times the upper limit of normal value after statin treatment.<br>• Muscle toxicity: creatine kinase > 10 times the upper limit of normal value, or the presence of muscle pain, myopathy or rhabdomyolysis.<br>• Death<br>• Other AEs/SAEs
**Statistical analysis**	Statistical analysis will be conducted with SAS 9.4. The two-sided $P<0.05$ is considered statistically significant.<br>**1.Major null hypothesis：**<br>1) In patients with acute mild ischemic stroke or high-risk TIA patients attributed to extracranial or intracranial atherosclerosis treated within 72 hours of ictus, there is no difference in the risk of a new stroke within 90 days between subjects with intensive antiplatelet therapy and those with standard antiplatelet therapy.<br>2) In patients with acute mild ischemic stroke or high-risk TIA patients attributed to extracranial or intracranial atherosclerosis treated within 72 hours of ictus, there is no difference in the risk of a new stroke within 90 days between subjects with immediate intensive statin therapy (atorvastatin 80mg/d) and delayed intensive statin therapy (atorvastatin 40mg/d).<br>3) In patients with acute mild ischemic stroke or high-risk TIA patients attributed to extracranial or intracranial atherosclerosis treated within 72 hours of ictus, there is no difference in the risk of a new stroke within 90 days between subjects with intensive antiplatelet combined with immediate intensive statin therapy (atorvastatin 80mg/d) and standard antiplatelet combined with delayed intensive statin therapy (atorvastatin 40mg/d).<br><br>**2. Data sets of statistical analysis ： intention-to-treat (ITT) population**<br><br>**3. Statistical analysis methods：**<br><br>3.1 The balance analysis of basic values (comparison between groups of baseline

	characteristics)<br>1) The measurement data will be tested by t test or Wilcoxon rank sum test;<br>2) The enumeration data between groups are compared with chi-square test, Fisher's exact probability method or Wilcoxon rank sum test.<br><br>3.2 Efficacy analysis<br>1) Primary outcome:<br>• Kaplan-Meier analyses will be used to evaluate survival curves for the primary endpoint, and survival curves will be compared by another factor stratification of the analytic design and using the Cox regression model Wald test. First, marginal effects will be compared between intensive and standard antiplatelet therapy, and also between immediate intensive statin therapy (atorvastatin 80mg/d) and delayed intensive statin therapy (atorvastatin 40mg/d). These are independent comparisons, they have no effect on the type I error rate. For comparisons of two therapies, the primary outcome of stroke will be considered statistically significant if the p-value is < 0.05. Second, if either intensive antiplatelet or immediate intensive statin therapy (atorvastatin 80mg/d) is statistically significant, the effects between combined intensive antiplatelet and immediate intensive statin treatment (atorvastatin 80mg/d) and combined standard antiplatelet and delayed intensive statin treatment (atorvastatin 40mg/d) will be tested.<br>2) Secondary outcomes:<br>a. Kaplan-Meier curve will be used to simulate the cumulative risk of 90-day follow-up combined with secondary endpoint events such as vascular events, ischemic stroke, TIA, myocardial infarction, vascular death and all-cause death. Cox proportional risk model will be used to calculate HR and the 95% confidence interval and the Log-rank test would be used to evaluate the efficacy;<br>b. For ordinal stroke or TIA, ordinal logistic regression will be used to calculate OR and 95% confidence interval. For the poor functional outcome (mRS score 2-6) and early neurological deficits (increase in NIHSS score at 7days) indexes, logistic regression will be used to calculate OR and 95% confidence interval.<br>c. Extreme values will be checked for validity and sensitivity analyses will be performed.<br>A two-sided test will be used for all statistics, and $P<0.05$ is considered statistically significant.<br>3.3 Safety analysis<br>Safety evaluation will be analyzed using safety data set. Moderate to severe bleeding, intracranial hemorrhage, and overall mortality will be calculated using the Kaplan-Meier curve to simulate the 3-month cumulative risk, and the Cox proportional hazards model to calculate the HR and 95% confidence interval. For other adverse events and laboratory abnormalities, the cases which was normal before the treatment and abnormal after the

	treatment would be mainly analyzed and listed, in addition to the comparison of differences before and after treatment.
**Follow-up plan**	Face to face interviews will be performed at baseline, Day7 ± 2, Day14 (or hospital discharge) , Day 21±2, and Day90 ± 7, and telephone interviews will be performed at the 6th month±14 days and 12th month±14 days after randomization.
**Study duration**	60 months

Reference

1. Wong KS, Chen C, Ng PW, et al. Low-molecular-weight heparin compared with aspirin for the treatment of acute ischaemic stroke in Asian patients with large artery occlusive disease: a randomized study. Lancet Neurol. 2007 May;6(5):407-13.

2. Chimowitz MI, Lynn MJ, Howlett-Smith H, et al. Comparison of warfarin and aspirin for symptomatic intracranial arterial stenosis. N Engl J Med, 2005,352(13):1305-1316.

3. North American Symptomatic Carotid Endarterectomy Trial Collaborators. Beneficial effect of carotid endarterectomy in symptomatic patients with high-grade carotid stenosis. N Engl J Med. 1991 Aug 15;325(7):445-53.

4. Amarenco P, Lavallée PC, Labreuche J, et al. One-Year Risk of Stroke after Transient Ischemic Attack or Minor Stroke. N Engl J Med. 2016 Apr 21;374(16):1533-42.

INSPIRES Abbreviated Vocabulary

Abbreviation	Interpretation
$ABCD^2$ score	To predict risk of stroke within 2 days after the onset of transient ischemic attack (TIA), and to predict the recurrence risk of mild stroke in the short term
ACEI	Angiotensin-converting enzyme inhibitor
AE	Adverse event
ALT	Alanine aminotransferase
ARB	Angiotensin-receptor blockade
ASA	Aspirin, Acetylsalicylic acid
ASSORT	Administration of Statin on Acute Ischemic Stroke Patient
AST	Aspartate aminotransferase
BP	Blood pressure
CABG	Coronary artery bypass graft
CEMRA	Contrast-enhanced magnetic resonance angiography
CHANCE	Clopidogrel in high-risk patients with acute non-disabling cerebrovascular events
CI	Confidence interval
CK	Creatine kinase
CLAIR	Clopidogrel plus aspirin versus aspirin alone for reducing embolisation in patients with acute symptomatic cerebral or carotid artery stenosis
COMPRESS	Combination of Clopidogrel and Aspirin for Prevention of Recurrence in Acute Atherothrombotic Stroke Study
CRF	Case report form
CRO	Contract Research Organization
CT	Computed Tomography
CTA	Computed tomography angiography
CYP2C19	Cytochrome P450, family2， subfamily C， polypeptide 19

CYP3A	Cytochrome P450， family 3, subfamily A
DSA	Digital subtraction angiography
DSMB	Data and Safety Monitoring Board
DWI	Diffusion-weighted imaging
EQ-5D	EuroQoL-5 Dimensions
GUSTO	Global Utilization of Streptokinase and Tissue Plasminogen Activator for Occluded Coronary Arteries
HR	Hazard ratio
ICH	Intracerebral hemorrhage
LDL-C	Low-density lipoprotein cholesterol
MRA	Magnetic resonance angiography
MRI	Magnetic resonance imaging
mRS	Modified Rankin Scale
NIHSS	National Institute of Health Stroke Score
NASCET	North American Symptomatic Carotid Endarterectomy Trial
OR	Odds ratio
PPI	Proton-pump inhibitor
rt-PA	Recombinant tissue-type plasminogen activitor
SAE	Serious adverse event
SAH	Subarachnoid hemorrhage
SAMMPRIS	Stenting and Aggressive Medical Management for Preventing Recurrent Stroke in Intracranial Stenosis
SPARCL	Stroke Prevention by Aggressive Reduction in Cholesterol Levels
TCD	Transcranial doppler
WASID	Warfarin - Aspirin for Symptomatic Intracranial Disease

1. Study Background

1.1 Burden and recurrence risk of acute mild ischemic stroke

Ischemic stroke is associated with higher rates of morbidity, recurrence, disability, and mortality, especially in Asian populations. Among them, non-disabling ischemic cerebrovascular events are the most common (accounting for more than 50% of all cerebrovascular diseases) and the recurrence rate is as high as 10-20%. Previous studies have found that early onset, severe artery stenosis and multiple infarctions are high risk factors for stroke recurrence in these patients.

(1) The early stage of onset: In the cohort studies, stroke recurrence in patients with ischemic stroke was mostly within 30 days after onset.[1-3] In patients with TIA or minor stroke of large-artery atherosclerosis origin, the risk of recurrence is high within 7 days after onset.[3,4]

(2) Severe artery stenosis: Early cohort studies showed that the risk of stroke recurrence caused by large-artery atherosclerotic stenosis was greater than that caused by other etiological types. [1,2,5,6] The TIA.org study published in 2016 showed that the recurrent stroke risk in 90 days and 1 year of TIA and minor stroke caused by large-artery stenosis is the highest.[3] The results from the imaging subgroup of the Clopidogrel in High-Risk Patients with Acute Nondisabling Cerebrovascular Events (CHANCE) suggested that the risk of stroke recurrence in patients with TIA and minor stroke with intracranial arterial stenosis in 90 days was significantly higher than those without intracranial stenosis.[4] The results of the WASID study indicated that the risk of stroke recurrence in patients with symptomatic intracranial artery stenosis (stenosis rate $\geq$ 70%) is higher than those with a stenosis rate of 50-70%.[7] Besides, the Chinese Intracranial Atherosclerosis Study (CICAS) reported that the proportion of patients with extracranial or intracranial arterial stenosis occlusion was as high as 46.6%. Of these cases, 37.5% had only intracranial arterial stenosis, 4.9% had only extracranial arterial stenosis, and 9.1% had intracranial and extracranial arterial stenosis. Patients with large-artery atherosclerotic stenosis had higher risk of recurrence and poorer prognosis. During 12-month follow-up, it was found that the risk of recurrent stroke increased with the severity of stenosis. [8]

(3) Multiple infarctions: A large number of studies showed that the risk of stroke recurrence is high in ischemic stroke combined with acute multiple infarctions. [9,10] Results of the TIA.org study and the CHANCE subgroup analysis indicated that multiple infarctions was an important imaging marker for the risk of stroke recurrence in TIA and minor stroke at 90 days. Results of studies on large-artery stenosis showed that patients with acute multiple infarctions from artery-to-artery embolism had a high risk of recurrent stroke. [8,11-13] Therefore, the recurrence risk estimator at 90 days (RRE-90) identified multiple infarctions as an important imaging marker for risk assessment of stroke recurrence.

1.2 Current status of drug therapy for acute mild ischemic stroke

Despite the high incidence and recurrence of stroke, effective therapies are extremely limited for ischemic cerebrovascular disease. The medication of ischemic cerebrovascular disease mainly relies on thrombolytic therapy in the super acute stage, antithrombotic therapy in the acute stage and rehabilitation treatment. However, most patients with ischemic stroke are not eligible for thrombolytic therapy because of its narrow "time window". Therefore, effective drug therapy is still needed to further reduce the risk

of stroke recurrence in acute ischemic stroke. Existing evidence-based medicine includes:

1.2.1 Antiplatelet therapy

1.2.1.1 Dual antiplatelet therapy (DAPT) for patients with ischemic stroke within 24 hours of onset.

The antiplatelet drugs recommended by 2014 AHA/ASA Guidelines for the Prevention of Stroke in Patients with Stroke and Transient Ischemic Attack include aspirin, clopidogrel, compound preparation with aspirin and dipyridamole, and ticlopidine. [14] Cilostazol is recommended as secondary prevention therapy for non-cardiogenic ischemic stroke in Chinese Guidelines for the Prevention of Stroke in Patients with Stroke and Transient Ischemic Attack.

The CHANCE study enrolled 5170 patients with acute mild ischemic stroke (NIHSS socre ≤3) or high-risk TIA who were randomized and received antiplatelet therapy within 24 hours after symptom onset and found that the combined clopidogrel and aspirin continued for 21 days significantly decreased the incidence of stroke within 90 days, compared with aspirin monotherapy. (8.2% vs. 11.7%; HR 0.68; 95% CI, 0.57 to 0.81; P<0.001). [15]

1.2.1.2 Patients with ischemic stroke within 3 days of onset may also benefit from DAPT

A meta-analysis of 14 studies, including the CHANCE study, involving a total of 9,012 patients with ischemic stroke compared the efficacy and safety of dual antiplatelet therapy versus mono-antiplatelet therapy within 3 days of onset. The results suggested that dual antiplatelet therapy significantly reduced the risk of stroke recurrence (RR, 0.69; 95%CI, 0.60-0.80; P<0.001) and composite outcomes of stroke, TIA, acute coronary syndrome, and all-cause death (RR, 0.71; 95%CI 0.63-0.81; P<0.001) and there was no significant increase in the risk of major bleeding. [16] Our previous meta-analysis also found similar results.

The COMPRESS study enrolled patients with large-artery atherosclerotic stroke within 2 days of onset. The primary outcome was the presence of new ischemic lesion (both symptomatic and asymptomatic) on MRI at the 30th day. The results showed that, compared with aspirin alone, aspirin combined with clopidogrel did not reduce the new ischemic lesion on MRI at 30th day and did not increase the risk of bleeding. [17] However, some limitations of the study restrict the evaluation of efficacy, such as a small sample size and the lack of loading dose for clopidogrel at the early stage of administration, which may reduce the efficacy of the DAPT greatly.

1.2.1.3 Patients with atherosclerosis or multiple infarctions might benefit most from DAPT

The CARRESS study enrolled 230 SICAS patients with TIA and ischemic stroke. 107 patients with micro-embolism were randomized, and 51 patients were given clopidogrel and aspirin for 7 days and 56 patients were given aspirin alone for 7 days. [18] The results showed that DAPT significantly reduced the number of micro-embolism monitored by TCD compared to aspirin, without increased risk of intracranial hemorrhage. The CLAIR study enrolled 98 patients with TIA and ischemic stroke (NIHSS score was less than 8, symptomatic large-artery atherosclerotic stenosis with microembolism within 7 days of onset), and the results showed that the combination of clopidogrel and aspirin for 7 days significantly reduced the microembolism compared with aspirin, without increase in the risk of intracranial hemorrhage. [19] The subsequent subgroup analysis of intracranial arterial stenosis found similar results. [20] The above two

studies based on subclinical outcomes showed that DAPT is a potential treatment of SICAS and short-term treatment did not increase the risk of bleeding.

For patients with TIA or minor ischemic stroke with intracranial arterial stenosis, subgroup analysis of the CHANCE study showed that the HR of stroke was 0.79 (0.47-1.32) in the group of aspirin and clopidogrel treatment, and 1.12 (0.56 -2.25) in the group of aspirin treatment in patients with intracranial atherosclerosis in 90 days.[4] The P-value of interaction analysis was 0.522, which suggested that DAPT failed to effectively reduce the risk of stroke recurrence at 90 days in high risk patients with non-disabling cerebrovascular events of intracranial arterial stenosis. This subgroup had a small sample size, but the survival curve showed that intracranial arterial stenosis, rather than non-intracranial arterial stenosis, is likely to benefit more from DAPT compared with aspirin alone, which further provided important basis for clinical trials. The subgroup analysis also suggested that short-term DAPT did not increase the risk of bleeding. Another subgroup analysis of the CHANCE trail showed that multiple infarcts was an important imaging marker of stroke recurrence and patients with multiple cerebral infarctions and intracranial artery stenosis were at the highest risk of recurrence.[21] Meanwhile, patients with multiple cerebral infarctions may benefit most from DAPT. The risks of stroke recurrence were 10.1% and 18.8% respectively in the groups of DAPT and aspirin alone (HR: 0.5; 95%CI: 0.3-0.96; P=0.04), which suggested that patients with multiple cerebral infarctions may be the most suitable target population of DAPT, especially patients with intracranial arterial atherosclerotic stenosis.[22]

1.2.1.4 New oral antiplatelet agents and anticoagulants remain to be explored

The Acute Stroke or Transient Ischemic Attack Treated with Aspirin or Ticagrelor and Patient Outcomes (SOCRATES) trial published in 2016 enrolled minor stroke patients within 24 hours of onset and randomly assigned them to ticagrelor group and aspirin group. The patients were treated for 90 days and the primary outcomes were composite events (stroke, myocardial infarction, and death). The results showed that, for patients with high-risk TIA or minor ischemic stroke within 24 hours of onset, ticagrelor did not significantly reduce the 90-day combined risk of stroke, myocardial infarction and death compared with aspirin, and did not increase risk of any severe bleeding events. [23] A subgroup analysis of the SOCRATES trial showed that compared with aspirin, ticagrelor could reduce the risk of stroke recurrence in TIA and minor ischemic stroke of large-artery stenosis. However, the results were not recommended by guideline because it was a subgroup of analysis. [24]

1.2.2 Statin therapy

1.2.2.1 Secondary prevention study for statin

Stroke Prevention with Aggressive Reductions in Cholesterol Levels (SPARCL) trial is the only study of the efficacy and safety of statin in patients with TIA or ischemic stroke so far. The study enrolled 4731 patients with non-cardiogenic (without atrial fibrillation or other cardiogenic embolism) stroke and (or) TIA, and they were randomized to the treatment of atorvastatin 80 mg/d or placebo, with an average follow-up of 4.9 years. The results showed that the baseline level of serum low-density lipoprotein cholesterol (LDL-C) was 3.3-3.5mmol/L, and after treatment, the level of LDL-C decreased to

1.9mmol/L in atorvastatin group, and 3.4 mmol/L in the placebo group. Intensive atorvastatin therapy significantly reduced the risk of recurrent stroke in patients with stroke and (or) TIA by 16% (P = 0.03), and the risk of primary coronary events by 35% (P = 0.003). This benefit was achieved on the basis of active antihypertensive and antiplatelet therapy. [25]

The carotid stenosis subgroup of the SPARCL study [33] revealed that patients with stroke and (or) TIA combined with carotid stenosis may benefit more from intensive atorvastatin therapy.[26] In the subgroup analysis, intensive atorvastatin(80 mg/d) treatment significantly reduced the risk of recurrent stroke by 33% (P = 0.02), reduced the risk of coronary events by 42% (P < 0.01), and reduced the risk of carotid endarterectomy by 56% (P = 0.006) in 1007 patients (21%) with stroke and (or) TIA combined with carotid stenosis . SPARCL subgroup analyses according to different types of stroke showed that intensive atorvastatin (80 mg/d) had a different degree of benefit for stroke patients with different subtypes, while patients with large-artery atherosclerotic stenosis may benefit more from statins. [27]

1.2.2.2 Reversal effect of statins on carotid plaque

High resolution magnetic resonance imaging (HRMRI) has been widely used in monitoring the effect of statins on carotid atherosclerotic plaques. Early studies mainly focused on changes in atherosclerotic plaque burden (luminal area, wall area, vessel wall thickness, and normalized wall index) before and after medical therapy. [28-33] Most studies found that statin significantly decreased the LDL-C level in serum, and improved the atherosclerotic plaque burden significantly over a certain period of time. Underhill et al reported that after the treatment of rosuvastatin for 2 years, the LDL-C level decreased significantly in atherosclerotic patients and the proportion of the lipid-rich necrotic core (LRNC)in the wall was decreased by 41.4% than that of baseline (P = 0.005). [28] Du et al found that LRNC decreased by an average of 7.3% (P = 0.044) after the treatment of statin for 90 days, which suggested that statin therapy may stabilize atherosclerosis plaques in the first 90 days. [29]

1.2.2.3 Study on statin therapy in acute stage

In addition to their LDL-lowering effects, statins have been found to possess various cytoprotective benefits, including protection of endothelial function, antioxidant properties, and anti-inflammatory effects.[34] Preclinical studies have shown that administering statins immediately after a stroke can reduce the size of the infarct and improve neurological outcomes. [35,36]

Currently, the studies of statin therapy for ischemic cerebrovascular disease were based on cohort studies and randomized controlled exploratory trials of small sample size. The prospective North Dublin Population Stroke Study published in 2011 enrolled 448 patients with ischemic stroke, and the results showed that initiating statin therapy within 72 h after stroke was significantly associated with the decrease of short-term and long-term mortality. [37] A randomized controlled trial published in 2007 enrolled 89 ischemic stroke patients admitted within 24 hours of onset with chronic statin therapy. Patients were randomly assigned either to statin withdrawal for the first 3 days after admission or to immediately receive atorvastatin. The trial found that statin withdrawal was associated with increased risk of death or dependency at 90 days (OR, 4.66; 95% CI, 1.46-14.91).[38] A recent Meta-analysis found that statin administration during hospitalization was associated with good functional outcomes (OR, 1.31;

95%, CI, 1.12-1.53; P = 0.001). The Meta-analysis also revealed that the treatment of statins before stroke is related to better functional outcome (OR: 1.50; 95% CI: 1.29-1.75; P < 0.001) and lower mortality (OR: 0.42; 95%; CI:0.21-0.82; P = 0.0108). Similar results were found in patients with large-artery atherosclerosis and small-artery occlusion, but was not found in patients with cardiogenic embolic stroke.[39]

A recent study enrolled 516 patients with acute ischemic stroke from large-artery atherosclerosis (TOAST subtype) and analyzed the impact of statin pretreatment on short-term outcomes. The study is a multicenter, prospective trial. The study concluded that statins pretreatment before stroke was independently associated with better functional outcomes (mRS score 0-1; OR: 2.44; 95%CI: 1.07-5.53), lower mortality (OR: 0.24; 95%CI: 0.08-0.75) and the rate of stroke recurrence (OR: 0.11; 95%CI: 0.22-0.46) within 1 month.[40] Therefore, this study suggests that statins pretreatment has a positive effect on prevention of stroke.

However, several small-sample randomized controlled trials have found no improvements in stroke recurrence and functional outcomes from early statin initiation. The EUREKA study included 316 patients with ischemic stroke in Korea, who received rosuvastatin with a dose of 20mg daily or placebo within 48 hours of onset and found no significant difference in occurrence of new ischemic lesions on DWI at 5 or 14 days between the two groups.[41] The STARS trial, a randomized controlled trial conducted in Spain, enrolled 104 patients with ischemic stroke with NIHSS score 4-22. The results suggested that simvastatin(40mg/d) started within 12 hours of onset did not improve the proportion of independent patients (mRS score≤2) at 90 days compared with placebo.[42] The ASSORT study conducted in Japan enrolled 257 patients with ischemic stroke (NIHSS score≤20) and randomly assigned to receive statin initiated within 24 hours or 7-day delayed statin, the results of which showed no significant difference in risk of disability between the two therapies.[43] This may be related to the small sample sizes of several studies, the variety of stroke causes enrolled, the severity of the disease, and the insufficient dosage of statins. **It is necessary to conduct large-scale randomized clinical trials for intensive statin therapy in the acute phase of large-artery atherosclerotic ischemic stroke.**

1.1 Current research questions and research hypotheses

1.3.1 Intensive antiplatelet therapy in patients with acute ischemic stroke or high-risk TIA from atherosclerosis

The INSPIRES study, in which patients with acute ischemic stroke or high-risk TIA from atherosclerosis were treated with antiplatelet therapy was conducted to answer the questions unsolved in the CHANCE study:

1）Whether patients with ischemic stroke from atherosclerosis with higher NIHSS scores may benefit from DAPT?

The CLAIR study expanded the NIHSS score of enrolled patients to 8, and the result showed no increased risk of bleeding after DAPT. The SOCRATES trial expanded the enrolled population NIHSS score to 5. Therefore, patients with high NIHSS score may benefit from DAPT. **The INSPIRES trial**

enrolled patients with expanded NIHSS score to 5.

2) Whether patients with ischemic stroke from atherosclerosis with extended time windows of onset may benefit from DAPT?

Meta-analysis published in 2013 showed that ischemic stroke patients enrolled within 72 hours of onset benefit from DAPT. **Therefore, the INSPIRES trial expands the time window of onset to 72 hours.**

3) Whether ischemic stroke with mild stenosis but multiple infarcts may benefit from DAPT?

The CHANCE imaging subgroup analysis showed that patients with multiple infarcts significantly benefited from DAPT, rather than patients with minor stroke with single infarction and TIA. Therefore, **multiple infarctions from embolization may be an important target for DAPT**.

Although results of the CHANCE trial confirmed that, in patients with TIA or minor stroke treated within 24 hours of onset, DAPT with clopidogrel and aspirin reduced the risk of stroke in the first 90 days compared with aspirin alone and the risk of bleeding did not increase. However, it has not been added to the indication in the package insert of clopidogrel. Some physicians in the real world follow the guidelines, but some follow the instructions. Therefore, patients will be enrolled in this study on a voluntary basis.

1.3.2 Intensive lipid-lowering therapy in patients with acute ischemic stroke or high-risk TIA from atherosclerosis

The SPARCL study did not answer the question of the efficacy and safety of statin therapy for ischemic cerebrovascular disease in the acute phase, because it enrolled patients with ischemic stroke and TIA within 6 months of onset, with a mean time of 87 days. It remains unclear for immediate intensive statin therapy to reduce the early recurrence risk in patients with ischemic stroke from large-artery atherosclerosis.

Moreover, atorvastatin at doses of 40mg/d and 80mg/d are both recommended as intensive statin treatment in the 2018 American Heart Association/ American Stroke Association Guidelines for the Early Management of Patients with Acute Ischemic Stroke. [44] However, the difference between the two doses in terms of therapeutic effect has not been observed in Asian patients. Some studies have shown that statin intolerance varies in race. Compared with Western populations, Chinese have higher incidence of intensive statin intolerance, which makes it difficult for guideline promotion. [45] Therefore, it is necessary to discuss the efficacy and safety of intensive statin treatment with different doses of atorvastatin in acute phase of ischemic stroke. Meanwhile, 2018 American Heart Association/ American Stroke Association Guidelines for the Early Management of Patients with Acute Ischemic Stroke show that high-intensity statin therapy should be given in the acute phase for patients with TIA and ischemic stroke, but there is a lack of evidence on when to initiate the statin therapy. The ASSORT study explored the efficacy and safety of delayed statins, but due to its small sample size, it was not used as evidence for guideline recommendations [47]. Hence, it is necessary to discuss the efficacy and safety of immediate or delay intensive statin treatment in reducing stroke recurrence and improving functional outcomes.

Based on the above evidence, we made the following 3 hypotheses: in patients with acute ischemic

stroke or TIA with high-risk symptomatic intracranial or extracranial atherosclerosis, ①Intensive antiplatelet therapy reduces the risk of stroke recurrence at 90 days without increasing the risk of bleeding compared with standard antiplatelet therapy; ②Immediate intensive statin therapy (atorvastatin 80mg/d) reduces the risk of stroke at 90 days compared with delayed intensive statin therapy(atorvastatin 40mg/d); ③Intensive antiplatelet therapy combined with immediate intensive statin therapy(atorvastatin 80mg/d) reduces the risk of stroke at 90 days compared with standard antiplatelet therapy combined with delayed intensive statin therapy(atorvastatin 40mg/d).

Therefore, a randomized, double-blind, placebo-controlled, multicenter, 2*2 factorial design trial was conducted to evaluate the efficacy and safety of intensive antiplatelet therapy versus standard antiplatelet therapy in reducing the risk of new stroke at 90 days in acute mild ischemic stroke or high-risk TIA patients attributed to intracranial or extracranial arterial atherosclerosis; to evaluate the efficacy and safety of immediate intensive statin therapy versus delayed intensive statin therapy in reducing the risk of stroke at 90 days in acute mild ischemic stroke or high-risk TIA patients attributed to intracranial or extracranial arterial atherosclerosis; To evaluate the effectiveness of intensive antiplatelet therapy combined with immediate intensive statin therapy versus standard antiplatelet therapy combined with delayed intensive statin therapy in reducing the risk of stroke at 90 days in acute mild ischemic stroke or high-risk TIA patients attributed to intracranial or extracranial arterial atherosclerosis.

2. Study Purposes

2.1 Primary purposes:

2.1.1 To evaluate the efficacy and safety of intensive antiplatelet therapy versus standard antiplatelet therapy in reducing the risk of new stroke at 90 days in acute mild ischemic stroke or high-risk TIA patients attributed to extracranial or intracranial atherosclerosis.

2.1.2 To evaluate the efficacy and safety of immediate intensive statin therapy (atorvastatin 80mg/d) versus delayed intensive statin therapy (atorvastatin 40mg/d) in reducing the risk of new stroke at 90 days in acute mild ischemic stroke or high-risk TIA patients attributed to extracranial or intracranial atherosclerosis.

2.1.3 To evaluate the efficacy and safety of intensive antiplatelet combined with immediate intensive statin therapy (atorvastatin 80mg/d) versus standard antiplatelet combined with delayed intensive statin therapy (atorvastatin 40mg/d) in reducing the risk of new stroke at 90 days in acute mild ischemic stroke or high-risk TIA patients attributed to extracranial or intracranial atherosclerosis.

Notes:

- Intensive antiplatelet therapy refers to dual antiplatelet therapy with aspirin and clopidogrel;
- Standard antiplatelet therapy refers to antiplatelet monotherapy with aspirin;
- Immediate intensive statin therapy (atorvastatin 80mg/d) refers to lipid-lowering therapy from the onset with atorvastatin at a dose of 80 mg daily and last for the first 21 days, followed by 40 mg daily for days22-90;
- Delayed intensive statin therapy (atorvastatin 40mg/d) refers to lipid-lowering therapy with atorvastatin at a dose of 40 mg for days 4-90);
- Acute mild ischemic stroke or high-risk TIA patients attributed to extracranial or intracranial atherosclerosis: acute mild stroke (NIHSS score≤5) or high-risk TIA ($ABCD^2$ score≥4) within 72 hours of onset meeting at least one of the following characteristics: 1) TIA with ≥50% stenosis of a major intracranial or extracranial artery that likely accounts for the clinical presentation; 2) Acute single cerebral infarction with ≥50% stenosis of a major intracranial or extracranial artery that likely accounts for the infarction and clinical presentation; 3)Acute multiple cerebral infarctions with stenosis of extracranial or intracranial arteries (stenosis degree unlimited) that likely accounts for the presentation.

2.2 Secondary purposes:

2.2.1 To evaluate the efficacy of intensive antiplatelet therapy versus standard antiplatelet therapy, immediate intensive statin therapy (atorvastatin 80mg/d) versus delayed intensive statin therapy (atorvastatin 40mg/d), intensive antiplatelet therapy combined with immediate intensive statin therapy (atorvastatin 80mg/d) versus standard antiplatelet therapy combined with delayed intensive statin therapy respectively (atorvastatin 40mg/d) at 90 days in acute mild ischemic stroke or high-risk TIA patients attributed to intracranial or extracranial atherosclerosis on the incidence of (definitions shown in appendix 1, 2 and 5):

- Combined vascular events: stroke (ischemic or hemorrhagic), myocardial infarction, and cardiovascular death.
- Ischemic stroke
- TIA
- Severity of stroke or TIA on an ordinal scale: a six-level ordered category scale combined vascular events with mRS score at 90 days: fatal stroke (stroke with subsequent death), severe stroke (stroke followed by mRS of 4-5), moderate stroke (stroke followed by mRS of 2-3), mild stroke (stroke followed by mRS of 0-1), TIA, and no stroke/TIA
- Myocardial infarction
- Vascular death
- All-cause death
- Poor functional outcome (mRS score 2-6)
- Poor quality of life (EQ-5D scale index score≤0.5)

2.2.2 To evaluate the efficacy in decreasing early neurological deficits (increase in NIHSS score at 7days), stroke recurrence, all-cause mortality and poor functional outcome (mRS score 2-6) at 1 year of intensive antiplatelet therapy versus standard antiplatelet therapy, immediate intensive statin therapy (atorvastatin 80mg/d) versus delayed intensive statin therapy (atorvastatin 40mg/d), intensive antiplatelet therapy combined with immediate intensive statin therapy (atorvastatin 80mg/d) versus standard antiplatelet therapy combined with delayed intensive statin therapy (atorvastatin 40mg/d) respectively in acute mild ischemic stroke or high-risk TIA patients attributed to intracranial or extracranial atherosclerosis. The new stroke or TIA is classified on a six-level ordered category scale combined vascular events with mRS score at 1 year: fatal stroke (stroke with subsequent death), severe stroke (stroke followed by mRS of 4-5), moderate stroke (stroke followed by mRS of 2-3), mild stroke (stroke followed by mRS of 0-1), TIA, and no stroke/TIA.

2.3 Safety purposes

2.3.1 To evaluate safety of intensive antiplatelet therapy versus standard antiplatelet therapy for 90 days in acute mild ischemic stroke or high-risk TIA patients attributed to intracranial or extracranial atherosclerosis on the incidence of:

- Moderate to severe bleeding (Appendix 6)
- Intracranial hemorrhage
- Hepatotoxicity: Alanine aminotransferase (ALT) or Aspartate aminotransferase (AST) > 3 times the upper limit of normal value.
- Muscle toxicity: Creatine kinase (CK) > 10 times the upper limit of normal value, or the presence of muscle pain, myopathy, or rhabdomyolysis.
- Death
- Other adverse events (AEs) / severe adverse events (SAEs)

2.3.2 To evaluate safety of immediate intensive statin therapy(atorvastatin 80mg/d) versus delayed

intensive statin therapy (atorvastatin 40mg/d) for 90 days in acute mild ischemic stroke or high-risk TIA patients attributed to intracranial or extracranial atherosclerosis on the incidence of:

- Moderate to severe bleeding (Appendix 6)
- Intracranial hemorrhage
- Hepatotoxicity: Alanine aminotransferase (ALT) or Aspartate aminotransferase (AST) > 3 times the upper limit of normal value.
- Muscle toxicity: Creatine kinase(CK) > 10 times the upper limit of normal value, or the presence of muscle pain, myopathy, or rhabdomyolysis.
- Death
- Other AEs / SAEs

2.3.3 To evaluate the safety of intensive antiplatelet therapy combined with immediate intensive statin therapy (atorvastatin 80mg/d) versus standard antiplatelet therapy combined with delay intensive statin therapy (atorvastatin 40mg/d) for 90 days in acute mild ischemic stroke or high-risk TIA patients attributed to intracranial or extracranial atherosclerosis on the incidence of:

- Moderate to severe bleeding (Appendix 6)
- Intracranial hemorrhage
- Hepatotoxicity: ALT or AST > 3 times the upper limit of normal value.
- Muscle toxicity: CK > 10 times the upper limit of normal value, or the presence of muscle pain, myopathy, or rhabdomyolysis.
- Death
- Other adverse events / severe adverse events

2.4 Subgroup analysis

2.4.1 To evaluate the efficacy of intensive antiplatelet therapy versus standard antiplatelet therapy, immediate intensive statin therapy (atorvastatin 80mg/d) versus delayed intensive statin therapy (atorvastatin 40mg/d), and intensive antiplatelet combined with immediate intensive statin therapy (atorvastatin 80mg/d) versus standard antiplatelet combined with delayed intensive statin therapy (atorvastatin 40mg/d) in reversing intracranial atherosclerotic artery stenosis and stabilizing the atherosclerotic vulnerable plaque in the high resolution MRI subgroup.

2.4.2 To evaluate the efficacy of intensive antiplatelet therapy versus standard antiplatelet therapy for 90 days on the incidence of the primary outcome in different subgroups:

- Subjects aged > 65 years vs. those aged ≤65 years.
- Female vs. male patients.
- Those with NIHSS score 4-5 vs. those with NIHSS score ≤3 at admission.
- Those randomized within 24 hours of onset vs. those randomized between 24 and 72 hours since onset.
- Those with ischemic stroke/TIA related to extracranial artery atherosclerosis vs. those related to intracranial artery atherosclerosis.

- Those with intracranial stenosis vs. those without intracranial stenosis.
- Those with extracranial stenosis vs. those without extracranial stenosis.
- Those with multiple infarctions vs. those with single infarction vs. those without infarction.
- Those with severe stenosis (⩾70%) vs. those with moderate stenosis (50%-69%).
- Those with hypertension vs. those who are normotensive.
- Diabetic patients vs. nondiabetics.
- Those with dyslipidaemia vs. without dyslipidaemia.
- Those with atherogenic dyslipidaemia (HDL-C<40 mg/dL and TG>200 mg/dL) vs. without atherogenic dyslipidaemia.
- Those with statin therapy within 1 month before randomization vs. without statin therapy.

In addition, relevant subgroups will be examined for genetic variability and biomarker characteristics.

2.4.3 To evaluate the efficacy of immediate intensive statin therapy (atorvastatin 80mg/d) versus delayed intensive statin therapy (atorvastatin 40mg/d) for 90 days on the incidence of the primary outcome in subgroups which is the same as those in 2.4.2.

3. Study Design

3.1 Study design

- A Randomized, double-blind, placebo-controlled, multicenter, 2×2 factorial trial
- The trial is intended to enroll 6100 subjects and complete follow-up of all subjects within 5 years.
- Centralized, unified and randomized grouping.
- Subjects will be randomly assigned to the following four groups:

  A: Intensive antiplatelet therapy +immediate intensive statin therapy (atorvastatin 80mg/d)

  B: Intensive antiplatelet therapy +delayed intensive statin therapy (atorvastatin 40mg/d)

  C: Standard antiplatelet therapy +immediate intensive statin therapy (atorvastatin 80mg/d)

  D: Standard antiplatelet therapy +delayed intensive statin therapy (atorvastatin 40mg/d)

Identity of study medication:

Groups	Date after Randomization	Dosage of study medication
Intensive antiplatelet therapy	Day 1	Clopidogrel 300mg/ day + aspirin 100-300mg/ day
	Day 2 - Day21±2	Clopidogrel 75mg/ day + aspirin 100mg/ day
	Day22±2 - Day 90	Clopidogrel 75mg/ day + aspirin placebo
Standard antiplatelet therapy	Day 1	Aspirin 100-300mg/ day + clopidogrel placebo
	Day 2 - Day 90	Aspirin 100mg/ day + clopidogrel placebo
Immediate intensive statin therapy	Day 1 - Day21±2	Atorvastatin 80mg/ day
	Day22±2 - Day 90	Atorvastatin 40mg/ day
Delayed intensive statin therapy	Day 1 - Day3	Atorvastatin placebo
	Day 4 - Day21±2	Atorvastatin 40mg/ day + atorvastatin placebo
	Day22±2 - Day 90	Atorvastatin 40mg/ day

Research design(drawing)

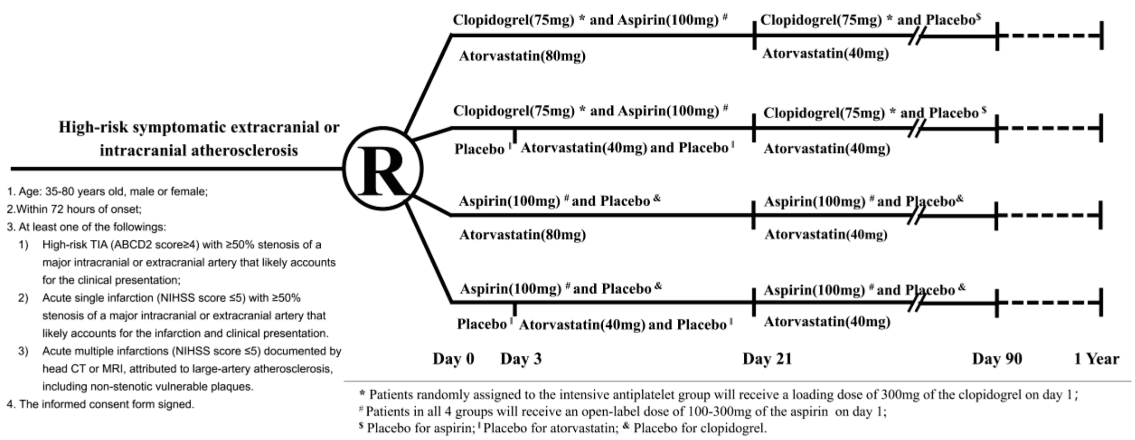

3.2 Follow-up plan

Subjects will receive a face-to-face visit at baseline, Day7±2, Day14 (or hospital discharge) , Day 21±2 and Day90 ± 7 , and telephone visits at the 6th month±14 days and 12th month±14 days after randomization. In addition, patients will be interviewed when new neurologic symptoms or suspicious events occur, including worsening of index ischemic events, new transient or persistent neurological symptoms.

3.3 Study execute time

Project Work Description	Estimated Completion Time
Study design, ethical review, study registration, sub-center recruitment	2018-06
The project is officially launched and the first subject is enrolled	2018-07
All subjects are enrolled and grouped	2021-12
90-day follow-up are finished for all subjects	2022-03
Data cleaning and database locking	2023-03
Clinical trial report completion	2023-06
Results publication	2023-12

4. Study Outcomes：

4.1 Primary outcome:

Stroke (ischemic or hemorrhagic)

4.2 Secondary outcomes:

- Combined vascular events： Stroke (ischemic or hemorrhagic), myocardial infarction, or cardiovascular death
- Ischemic stroke
- TIA
- Severity of stroke or TIA on an ordinal scale: (a six-level ordered category scale combined vascular events with mRS score: fatal stroke (stroke with subsequent death), severe stroke (stroke followed by mRS of 4-5), moderate stroke (stroke followed by mRS of 2-3), mild stroke (stroke followed by mRS of 0-1), TIA, and no stroke/TIA)
- Myocardial infarction
- Vascular death
- All-cause death
- Poor functional outcome (mRS score 2-6)
- Poor quality of life (EQ-5D scale index score≤0.5)
- Early neurological deficits (increase in NIHSS score at 7days)

4.3 Primary safety outcome:

Moderate to severe bleeding (Appendix 6)

4.4 Secondary safety outcomes:

- Intracranial hemorrhage
- Hepatotoxicity: ALT or AST > 3 times the upper limit of normal value;
- Muscle toxicity: CK > 10 times the upper limit of normal value, or the presence of muscle pain, myopathy, or rhabdomyolysis.
- Death
- Other AEs / SAEs

5. Subjects Selection

5.1 Inclusion Criteria

1. Age :35-80 years;
2. Less than 72 hours from the onset;
3. At least one of the followings (a-c)

a) High-risk TIA (ABCD2 ≥ 4) with ≥50% stenosis of a major intracranial or extracranial artery that likely accounts for clinical presentation.

b) Acute single cerebral infarction (NIHSS≤5) with ≥50% stenosis of a major intracranial or extracranial artery that likely accounts for the infarction and clinical presentation.

c) Acute multiple cerebral infarction (NIHSS≤5) with stenosis of extracranial or intracranial arteries (stenosis degree unlimited) that likely accounts for the presentation.

4. Written informed consent.

Notes:

- Intracranial large arteries include the intracranial portion of internal carotid arteries, middle cerebral arteries (M1/M2) , anterior cerebral arteries (A1/A2), posterior cerebral arteries (P1/P2), intracranial portion of vertebral arteries, and basilar artery; [46,47] The degree of intracranial artery stenosis is defined on MRA, CTA or DSA by criteria from the Warfarin–Aspirin Symptomatic Intracranial Disease (WASID) study.[46]
- Extracranial large arteries include the extracranial portion of carotid arteries and vertebral arteries (including the portion from initiation of carotid arteries and vertebral arteries to the start of intracranial section, except the subclavian artery, aortic arch and brachiocephalic trunk). The degree of extracranial arteries stenosis is defined on carotid duplex ultrasound, CEMRA, CTA, or DSA by criteria from the North American Symptomatic Carotid Endarterectomy Trial (NASCET).[48]
- Acute ischemic stroke with multiple infarction lesions refers to more than one lesion appearing in different locations (separated in space or non-continuous on contiguous slices) on diffusion DWI/ADC imaging however, it will be considered as the same one if the lesion is continuous on the adjacent levels.[3]
- TIA: Sudden neurologic deficit lasting less than 24 hours, without non-ischemic causes.
- TIA patients with acute multiple cerebral infarctions will also be enrolled.

5.2 Exclusion criteria

1. Presumed cardioembolic stroke or TIA (e.g. atrial fibrillation, heart valve prosthesis, atrial myxoma, endocarditis, etc.).
2. Other determined etiology of stroke or TIA (e.g. aortic dissection, cervico-cerebral artery dissection, vasculitis, vascular malformation, moyamoya disease/syndrome, fibromuscular dysplasia, etc.).
3. Non-vascular neurological diseases (e.g. intracranial tumor, multiple sclerosis, etc.).
4. Index infarction affects ≥ 50% of a cerebral lobe (e.g. parietal, frontal, occipital).
5. Hemorrhagic transformation after onset.
6. Contraindications to clopidogrel, aspirin or atorvastatin:
    a. History of hypersensitivity;
    b. Severe heart failure (NYHA classification: III- IV) or asthma;

c. Coagulation disorder or systemic bleeding;

d. History of drug-induced hematologic or hepatic abnormalities;

e. Leukopenia (< 2×10^9/L) or thrombocytopenia (<100×10^9/L);

f. Active liver disease;

g. Pregnancy or lactation period;

***Severe heart failure：** New York Heart Association (NYHA) Classification III and IV

I Cardiac disease, but no symptoms and no limitation in ordinary physical activity, e.g. no shortness of breath when walking, climbing stairs etc.

II (mild heart failure) Mild symptoms (mild shortness of breath and/or angina) and slight limitation during ordinary activity.

III (moderate heart failure) Marked limitation in activity due to symptoms, even during less-than-ordinary activity, e.g. walking short distances (20–100 m). Comfortable only at rest.

IV (Severe heart failure) Severe limitations. Experiences symptoms even while at rest. Mostly bedbound patients.

7. Pre-existing disability with mRS score>2.
8. Intra- arterial or intravenous thrombolysis or endovascular therapy after onset.
9. Defibrinogen therapy (e.g. defibrase and lumbrokinase) after onset.
10. Creatine kinase > 5 times the upper limit of normal value of onset.
11. Drug use related to statin metabolism within 14 days before randomization (e.g. immune-suppressive drugs, antifungal agents, fibrates).
12. Severe hepatic insufficiency (ALT or AST > 2 times the upper limit of normal value) or renal insufficiency (creatinine > 1.5 times the upper limit of normal value or glomerular filtration rate < 40 ml/min/1.73 m2).
13. Dual antiplatelet therapy with aspirin and clopidogrel within 14 days before randomization (Patients who started aspirin plus clopidogrel without loading dose(300mg) of clopidogrel after onset are not excluded from the trial.).
14. High-intensity statin therapy within 14 days before randomization (e.g. atorvastatin ≥ 40mg/d, rosuvastatin ≥ 20mg/d).
15. History of intracranial hemorrhage (e.g. intracerebral or subarachnoid hemorrhage).
16. Gastrointestinal bleeding or major surgery within 90 days.
17. History of intracranial or extracranial angioplasty.
18. Planned long-term use of antiplatelet drugs or non-steroidal anti-inflammatory drugs except for study drugs.
19. Planned surgery or revascularization that may need to stop taking the study drugs within the next 90 days.
20. Anticipated life expectancy < 90 days.
21. Currently participating in any other investigational drug or device study.
22. Unable to complete the follow-up (e.g. dementia, alcoholism, substance abuse, severe mental disease).

6. Therapeutic Regimen

6.1 Drug information and specification

Drugs	Specifications	Dosage form	Administration route	Production Company	Storage	Term of validity
Clopidogrel	75 mg	tablet	oral	Sanofi Winthrop Industries	No special requirement	36months
Aspirin	100 mg	tablet	oral	Bayer S.p.A.	Below 25℃	36 months
Atorvastatin	20mg	tablet	oral	Beijing Jialin Pharmaceutical CO.,LTD	Below 25℃	36 months

6.2 Drug dosage

Groups	Date after Randomization	Dosage of study medication
Intensive antiplatelet therapy	Day1	Clopidogrel 300mg/day+asprin100-300mg/day
	Day2 - Day21±2	Clopidogrel 75mg/day+ aspirin 100mg/day
	Day22±2 - Day90	Clopidogrel 75mg/ day + aspirin placebo
Standard antiplatelet therapy	Day1	Aspirin 100-300mg/ day + clopidogrel placebo
	Day2 - Day90	Aspirin 100mg/day+ clopidogrel placebo
Immediate intensive statin therapy	Day1 - Day21±2	Atorvastatin 80mg/day
	Day22±2 - Day90	Atorvastatin 40mg/day
Delayed intensive statin therapy	Day1 - Day3	Atorvastatin placebo
	Day4 - Day21±2	Atorvastatin 40mg/day + atorvastatin placebo
	Day22±2 - Day90	Atorvastatin 40mg/day

6.3 Regimen

Administration: oral; The final 4 treatment groups according to the 2×2 factorial design are as follows (enrolled patients were randomized into the following 4 groups, blinded for both investigators and patients):

A: Intensive antiplatelet therapy + immediate intensive statin therapy (atorvastatin 80mg/d)

B: Intensive antiplatelet therapy + delayed intensive statin therapy (atorvastatin 40mg/d)

C: Standard antiplatelet therapy + immediate intensive statin therapy (atorvastatin 80mg/d)

D: Standard antiplatelet therapy + delayed intensive statin therapy (atorvastatin 40mg/d)

7. Study Implementation

7.1 Randomization procedures

Random number lists will be generated by the Statistics and Data Center at the China National Clinical Research Center for Neurological Diseases (Beijing, China). Regimens for patients will be packed according to the random code lists. Patients will be assigned to four groups with the approximate ratio of 1:1:1:1, and receive: A intensive antiplatelet therapy+ immediate intensive statin therapy (atorvastatin 80mg/d), B intensive antiplatelet therapy+ delayed intensive statin therapy (atorvastatin 40mg/d), C standard antiplatelet therapy+ immediate intensive statin therapy (atorvastatin 80mg/d), or D standard antiplatelet therapy+ delayed intensive statin therapy (atorvastatin 40mg/d) respectively. Randomization will be centralized. We applied randomized block design to generate random number to ensure the ratio between the four groups will be approximately 1:1:1:1. If a center runs out of its random code lists, a new block will be placed to the center for new random numbers. Each patient eligible to the INSPIRES trial will be assigned a random number in order strictly and the corresponding study drug box.

7.2 Blinding and Emergency Unblinding

7.2.1 Description of Blinding

Two clopidogrel tablets (75mg clopidogrel and matching placebo) used in this trial were indistinguishable (they were identical in size, shape, color, appearance and smell).

Two aspirin tablets (100mg aspirin and matching placebo) used in this trial were indistinguishable (they were identical in size, shape, color, appearance and smell).

Two atorvastatin tablets (20mg atorvastatin and matching placebo) used in this trial were indistinguishable (they were identical in size, shape, color, appearance and smell).

Centers are not able to apply unblinding with biological experiment in this study. Investigators shall never unblind the random code unless special situations occur such as SAE, when disclosure of the study drug information is important for the treatment of the subject. Efficacy and safety outcomes will be submitted to Adjudication Committee, who should be blinded to the randomization, for the final determination.

7.2.2 Emergency unblinding procedure

Emergency unblinding should only be undertaken when it is essential to identify the study drug information for effective treatment of the patient who suffer from AEs or SAEs. Contact should be made with the Monitoring Committee in advance if possible.

If the investigator finds it essential for the treatment to perform unblinding, he/she should inform the Monitoring Committee and sponsor as soon as possible. All calls should be recorded by the Contract Research Organization (CRO).

The date, time and reason for unblinding should be documented by the investigator and study drug must be discontinued after emergency unblinding.

7.3 Study drug management

7.3.1 Study drug supply and storage

Clopidogrel, aspirin, atorvastatin calcium, and placebo for the study should be purchased by sponsors. The study drug will be stored in a safe place, under the direct management of the investigator or other authorized persons, and stored under the conditions described on the drug label.

7.3.2 Study drug packaging and labeling

7.3.2.1 Study drug packaging

According to the design of the study protocol and follow-up period, the study drugs will be divided into two boxes according to the different phases of the trial, i.e. box 1 and box 2. Each box will contain three study drugs respectively, as follows.:

- **Box1 will provide the drug from day1 to day21 after randomization**
  a) Active clopidogrel (75mg/ tablet) or clopidogrel placebo without open label. A total of 4 plates (7 tablets/ plate). Patients should take 4 tablets on the first day and 1 tablet per day for the rest days.
  b) Active aspirin (100mg/tablet) . A total of 2 plates (15 tablets/ plate). 1-3 tablets should be taken under the direction of doctors on the first day and 1 tablet per day for the rest days.
  c) Active atorvastatin (20mg/tablet) or atorvastatin placebo without open label. There are two types of packaging: 2 plates (7 tablets/ plate) of active atorvastatin or atorvastatin placebo, and 20 bags (4 tablets/ bag) of active atorvastatin or mixture of 2 tablets of active atorvastatin and 2 tablets of atorvastatin placebo. Plates are used for days1 to 3 at a dose of 4 tablets daily. Bags are used for days 4 to 21 at a dose of 1 bag daily.
- **Box 2 will provide the drug from day 22 to day 90 after randomization**
  a) Active clopidogrel (75mg/tablet) or clopidogrel placebo without open label. A total of 11 plates (7 tablets/ plate). Patients need to take 1 tablet per day.
  b) Active aspirin (100mg/tablet) or aspirin placebo without open label. A total of 5 plates (15 tablets/ plate). Patients need to take 1 tablet per day.
  c) Active atorvastatin (20mg/tablet). A total of 21 plates (7 tablets/ plate), patients need to take 2 tablets per day.

Finally, Box1 and Box2 will be packed in a bigger box, which will be randomized and dispensed for each patient.

7.3.2.2 Drug label

The drug box label should contain:

- Abbreviation of the study and logo of the study
- Random code
- Time of medication use, like box1: “drug used at day 1-21 after randomization”, box2: “drug used at day 22-90 after randomization”; The medication bags of atorvastatin should be marked with: “drug used at day 4-21 after randomization”
- Dosage form, dosage and administration route of the study drug

- Instructions for usage
- Storage conditions
- Expiration date
- "Please take the medication with the instruction of the doctor and bring the remaining tablets back for counting at the next visit"
- "Keep away from children to avoid accidental ingestion by children"
- "For the INSPIRES trial use only"

7.3.3 Responsibility for drug management

Investigators, pharmacist and anyone who store and dispense the drug should make sure the study drugs are stored and dispensed safely in accordance with the demands of the sponsor and the protocol as well.

It is investigators' responsibility to make sure the study drug be dispensed in accordance with the protocol and document the dispensing and retrieving properly.

Any quality defect in dispensing and usage of the study drug (including appearance, labeling, validity period etc.) should be reported to the sponsors in time.

In no circumstance should the study drug be given to the third party. The study drug cannot be used or handled by any means other than as required by the clinical trial protocol.

7.3.4 Compliance of treatment and reliability

The information of the usage of all drugs including study medication and concomitant medication should be recorded in the appropriate location on the CRF. Subjects will receive drugs for the first period that is sufficient for the first 21 days after randomization. On the day 21±2, the patient will receive the second period drugs that is sufficient to the end of the study. At day 21±2 exchange of study drug and day 90±7 visit, subjects should take back the residual study drug and the empty package. Pill count should be applied for the retrieving drug and documented in the CRF.

Compliance will be accessed by the investigator at each visit using pill-counting for the retrieving drug.

Investigator should record the usage of study and concomitant medication for individual in the corresponding page in CRF.

Time of discontinuation and recovery should be documented.

7.3.5 Return and destruction of the drug

Investigators (or pharmacists) should establish a detailed record for return of the study drugs and investigators and the monitoring team should jointly confirm the record.

Investigators shall not destroy the study drugs (including not used and partly used), unless being authorized in writing by the principal investigator.

Investigators will adopt the recall procedure to recall study drugs if any defect were found in quality. It is the responsibility of the investigator to comply with any requests made by the principle investigator in order to recall the study drug and eliminate potential hazards.

7.4 Concomitant medication and intervention

Any interventions (including medication, surgery, vascular interventions,) performed after randomization,

as well as any medication changes during the study should be documented in the CRF.

7.4.1 Concomitant medication contraindicated

1) Anti-platelet therapy: Ticagrelor, dipyridamole, cilostazol. ticlopidine, prasugrel, GPIIb/IIIa receptor antagonist, ozagrel etc.

2) All forms of anticoagulant drugs.

3) Thrombolytic therapy: rt-PA, urokinase and streptokinase etc. Patents who received intravenous or intra-arterial thrombolysis within 24 hours before randomization should not be included. If patients received intravenous or intra-arterial thrombolysis after randomization, the study antiplatelet agents must be discontinued for at least 24 hours before restart. While atorvastatin in the study is not affected.

4) Batroxobin, defibrase, snake venom preparation, lumbrukinase etc.

5) Non-steroidal anti-inflammatory drugs (NSAIDs, inhibitors of Cox1 and Cox2). Usage of NSAIDs (inhibitors of Cox1 and Cox2) within 7 days after randomization is violation to the protocol. If necessary, NSAIDs should be initiated after 24 hours after discontinuation of study drugs for less than 5 days, and should not be used within 8 days after randomization.

6) Potent inhibitor of CYP3A: Ketoconazole, nefazodone, ritonavir, saquinavir, atazanavir, nelfinavir, itraconazole, voriconazole, clarithromycin, telithromycin except for erythromycin and azithromycin;

7) Potent inducer of CYP3A: Rifampicin, dexamethasone, phenytoin, carbamazepine, oxcarbazepine, phenobarbitone;

8) Potent inhibitor of CYP2C19: Omeprazole, esomeprazole, fluvoxamine, fluoxetine, moclobemide, fluconazole, naphthyridine, ciprofloxacin, cimetidine, chloromycin;

9) Potent inhibitor of P-glycoprotein (P-gp): Verapamil, quinidine, cyclosporine;

10) Substrates of P-glycoprotein (P-gp): Digoxin. If necessary, If digoxin is absolutely necessary, reduce the digoxin dose as appropriate and monitor digoxin blood levels closely;

11) Other statins, drugs or food that affect the metabolism of statins: Rosuvastatin, simvastatin, pravastatin, pitavastatin, lovastatin, fiber acid derivative (fibrates), niacin or immunosuppressive regulating the lipid, oral contraceptive, hypocol.

12) Other relative drugs: evening primrose oil, curcumin, capsaicin staggerweed, ginkgo, garlic, the root of red-rooted salvia, cholestyramine.

13) Chinese patent drug： Chinese medicines with antiplatelet and antithrombotic effects in the instructions, including tablets and injections.

7.4.2 Concomitant medication permitted

Medications not listed in 7.4.1 can be used in this study. Patients with comorbidities (such as hypertension, diabetes, coronary heart disease, seizures etc.) can use diuretic, beta-blockers, ACEI, ARB, CCB, vasodilator, anti-diabetes agents (including insulin), anti-seizures agents and so on. The diagnosis, medications, dosage and administration should be documented in the CRF in detail. When other treatments have to be added due to special circumstances (e.g., another disease during the study period), they must be described in the case report form, including the diagnosis, medications, dosage and

administration.

1) When an emergency occurs, such as acute coronary syndrome (ACS) or when percutaneous coronary intervention (PCI) is necessary to intensify antiplatelet therapy, the dose of antiplatelet agents could be increased temporally. The study dose will be restored after the condition has stabilized, and the drug adjustment will be recorded in the CRF.

2) H2 receptor blockers: All except cimetidine.

3) Proton pump inhibitors (PPIs): PPIs could be used if necessary except omeprazole and lansoprazole, for example, dexlansoprazole, lansoprazole, pantoprazole.

4) 5-serotonin reuptake inhibitors (SSRIs): When using paroxetine, sertraline and citalopram, attention should be paid on preventing hemorrhage due these drugs may increase the risk of the hemorrhagic event.

7.4.3 Surgery and invasive procedures

If surgery or invasive procedures (including vascular surgery, angiography or angioplasty of coronary, internal and external carotid artery) is necessary within 90 days after randomization: for procedures with major hemorrhagic risk, the study drug should be discontinued for at least 5 days prior to the procedure and restarted when the investigator deems the risk of bleeding to be low after operation. For other surgical/ invasive procedures, the study drug should be continued or suspended according to the investigator's judgment.

7.5 Temporary, permanent discontinuation of the study drug(s) or loss to follow-up

Study drug should be maintained as long as possible. Judgment for temporary discontinuation should be made when facing withdrawal. Permanent discontinuation is the last resort. Any forms of discontinuation of the study drug should be documented in CRF in detail. Subjects should participate in the study as long as possible in any situations. After randomization, study drug discontinuation for any reason does not constitute withdrawal from the study or stopping follow-up, evaluation and visit should be continued.

7.5.1 Temporary discontinuation of study drug

If the study drug is temporarily discontinued, it should be restarted as soon as medically justified in the opinions of the investigators.

Study drug should be temporarily discontinued in the situations listed below:

1) Severe thrombocytopenia (platelet count <50,000/uL, 50×10^9/L). The study drug should be restarted once the patient recovers from the severe thrombocytopenia.

2) Severe bleeding events (shown in appendix 6)

3) Use of contraindicated concomitant medications (listed in 7.4.1).

4) Operation and procedures probably causing major bleeding (listed in 7.4.3).

5) Abnormal liver function (transaminase level above twice the normal value). The study drug should be restarted to patients once the transaminase level is back to normal.

For other operation and procedures, continuation and discontinuation should be justified in the opinions of the investigators.

Once investigators justify the study drug unrelated to adverse events and with no contraindication for discontinuation, study drugs should be restarted to the patient as soon as possible in close and reasonable monitoring. The time of temporary discontinuation of study drug should be carefully documented in the CRF.

7.5.2 Discontinuation from the study drug permanently

1) Patient's decision. Patients can discontinue the study anytime they want unless it will affect their treatment.

2) Investigator's decision. Including but not limited to the following circumstances:

a) Primary outcomes (ischemic or hemorrhage stroke) occur
b) Pregnancy or preparation for pregnancy
c) Incorrect enrollment that does not meet the inclusion/exclusion criteria puts the patient at higher risk
d) Serious noncompliance
e) Emergence of adverse events that investigators believe may put patients at higher risk of continuing treatment
f) Definite indications for anticoagulant agent
g) Investigators decide it is inappropriate to restart the study drug after discontinuation

7.5.3 Measures after permanent discontinuation

The investigators should provide follow-up medical care for all patients who are permanently discontinuation from the study, and should inquire the cause of drug withdrawal, presence of the primary outcomes and adverse events in detail. Besides, investigators should follow up on primary outcomes and adverse events and record in the CRF. Investigators should recall the remaining unused study drugs.

Patients discontinuing from study drugs before the last visit should accomplish the 90-day follow up as planned or when recovered from adverse events to normal/stable condition (the later time point should be chosen). Investigators should document all definite withdrawal carefully in the CRF.

7.5.4 Loss to follow-up

Investigators should try their best to keep contact with every patient, making sure the reason of loss to follow-up and their health situation. All CRFs of missed patients should be recorded until the last follow-up visit.

7.6 Quit from study

Those who quit the study should not be included again. The randomization number and study drugs of this subject should not be used again. Randomized patients must not be replaced. Investigators should confirm the withdrawal along with the monitoring committee. Subjects who were randomized and had one or more doses recorded were required to complete the calendar visits according to the protocol.

8. Blood Sample Collection

Blood sample collection refers to serum, plasma and leukocyte (for detailed information, refer to the “collection and management of the biological sample”) that requires to be collected to the corresponding laboratory and transported thereafter with cold-chain to Beijing Tiantan Hospital for centralized storage and analysis. The time of collection should be documented carefully in the CRF.

9. Data Collection

Investigators should finish the CRF promptly as instructed by the protocol and make sure those documents are accurate, complete and without delay. The MRI data of all included patients will be collected in the DICOM form with complete sequence information (sequences T1+T2+FLAIR+DWI/ADC+MRA are required, SWI/GRE-T2* if qualified). Data collected from vascular test, transthoracic echocardiography, 24hrs Holter and lab tests should be uploaded as original photo or in the DICOM form.

10. Study Procedure

All subjects should be screened by investigators with a screening table containing the sub-center number. The screening table will be used to analysis representativeness of subjects included in the study in each sub-center.

10.1 Screening and enrollment

- Patients meeting the inclusion criteria and not the exclusion criteria should sign the informed consent document.
- Confirm the time of the onset of the initial event
- Perform laboratory tests assessment, including the first emergency routine, emergency renal function (serum creatinine, blood urea nitrogen), emergency liver function (serum transaminase) and emergency blood clotting after onset.
- Perform imaging assessment: brain histology assessment, and/or intracranial artery assessment, and/or extracranial artery assessment. Brain histology assessment includes head CT or MRI for diagnosis and differential diagnosis of TIA and cerebral infarction and possible cerebral infarction model evaluation. Intracranial and extracranial artery assessment examination are used for screening. Intracranial artery assessment examination includes any test of MRA、CTA、DSA. Extracranial artery assessment examination includes any test of neck vascular ultrasound, CEMRA, CTA on the aortic arch, DSA;

Note: If a patient is found to be arterial stenosis only by assessing the intracranial arteries and meets the inclusion criteria, he/she can be enrolled without extracranial artery assessment examination. Similarly, if a patient is found to be arterial stenosis only by assessing the extracranial arteries and meets the inclusion criteria, he/she can be enrolled without intracranial artery assessment examination.

(As these radiological tests are recommended as routine tests for ischemic stroke and TIA patients, these examinations will be at patients' own expense)

A thorough explanation of the information of the study will be outlined and shown by the investigators in the written material.

10.2 Day of Randomization

- Record basic demographic information, medical and medication history and physical examination of subjects
- Conduct $ABCD^2$ score (for TIA patients), EQ-5D score and neurological examination (including mRS and NIHSS).
- Apply 12-lead electrocardiogram (ECG) to rule out atrial fibrillation, sick sinus syndrome and second or third degree atrioventricular block.
- Subjects obtain random code, and receive a drug box (for phase 1) in accordance to the randomization number to supply the study drug used from day 1 to day 21. Study drugs should be taken as soon as the randomization is finished (within 1 hour after randomization). Instructions for

taking the first study drug should be given by the investigator. (The first dose includes the total dose of medication taken by the patient after the onset of illness, taken in the ambulance and emergency room, and taken after admission to the hospital).

- Fasting laboratory tests should be completed within 24 hours after randomization (Including routine blood counts, biochemical panel [hepatic function, kidney function, blood lipid, creatine kinase], glycated hemoglobin, homocysteine, coagulation function). Fasting venous blood sample should be collected at the same time, and serum, plasma and white blood cells should be extracted and stored separately (Instructions are listed in the biological sample collection handbook). If not, 72 hours after randomization is the final deadline.
- Complete the initial diagnosis of the initial event for subjects.
- High-resolution MRI of intracranial arteries (high-resolution MRI subgroup only): need to be done within 24 hours of randomization completion.

10.3 Day7 visit

- Record NIHSS score.
- Finish brain histological examinations: including MRT and CT scan. If the brain MRI isn't done before randomization, it should be finished within 7 days after randomization, with complete sequence information (sequences T1+T2+FLAIR+DWI/ADC+MRA are required, SWI/GRE-T2* if qualified). If head MRI is finished, head CT would not be necessary. If the vascular assessment was completed prior to randomization, it doesn't need to be repeated.
- Finish vascular assessments: assessments of intracranial arteries (including MRA, CTA, DSA) and assessments of extracranial arteries (including neck vascular ultrasound, CEMRA, CTA on the arch, DSA), at least one item each is necessary. If the assessment was completed prior to randomization, there is no requirement to review.
- Cardiac examinations: including echocardiography (transthoracic or transesophageal) and electrocardiography (12-lead ECG or long-range HOLTER).
- Finish high-resolution intracranial arterial MRI (only in high-resolution MRI subgroups): examination needs to be completed within 24 hours since randomization.
- Finish laboratory tests: blood routine, biochemistry (including liver function, renal function, blood lipids, creatine kinase) and coagulation function. If the test is completed within 24 hours of screening or randomization, there is no requirement to review.

10.4 Day 14 or Hospital discharge visit

- Record NIHSS score.
- Record the final diagnosis of this event.
- Record concurrent medication since last follow-up.
- Evaluate subjects' compliance.
- Record new strokes and new vascular events since the last follow-up.
- Record adverse events, bleeding events since the last follow-up.

- Make an appointment of day 21±2 visit.

10.5 Day 21±2 visit

- Record concurrent medication since last follow-up.
- Evaluate subjects' compliance.
- Record new strokes and new vascular events since the last follow-up.
- Record adverse events, bleeding events since the last follow-up.
- Recall of remaining study drugs in Box1, dispense study drug Box2 (study drugs from day22±2 to day90±7 after randomization). Study physicians should instruct patients in the second phase of medication administration.
- Make an appointment of day90 ± 7 visit.

. **10.6 Day 90±7 visit**

- Finish clinical scores (NIHSS, mRS, EQ-5D).
- Finish intracranial arterial high-resolution MRI (only for high resolution MRI subgroups).
- Finish laboratory tests: blood routine and biochemical panel (including liver function, renal function, blood lipids, creatine kinase). Fasting venous blood sample should be collected at the same time, and serum, plasma and white blood cells should be extracted and stored separately (Instructions are listed in the biological sample collection handbook).
- Record concurrent medication since last follow-up.
- Evaluate the compliance of the subject since the last visit.
- Record new strokes and new vascular events since the last follow-up.
- Record adverse events, bleeding events since the last follow-up.
- Retrieve study drug box of phase 2 (Study drug from day 22±2 to day 90).
- Summarize the whole study.
- The subject and the investigator decide which anti-platelet drug should be used after the study together.
- Inform the subject for long-term follow-up, make an appointment of the next visit: the 6th month ± 14 days after administration.

10.7 6th month± 14 days visit

- Record personal history of the subject, especially smoking and drinking history.
- Record concurrent medication since last follow-up.
- Record new strokes and new vascular events since the last follow-up.
- Record adverse events, bleeding events since the last follow-up.
- Make an appointment of the 12th month ± 14 days visit.

10.8 12th month± 14 days visit

- Record clinical scores (mRS, EQ-5D).
- Record personal history of the subject, especially smoking and drinking history.

- Record concurrent medication since last follow-up.
- Record new strokes and new vascular events since the last follow-up.
- Record adverse events, bleeding events since the last follow-up.

10.9 Clinical outcomes assessment

If suspected neurologic symptoms occur (including deterioration of the initial ischemic cerebrovascular disease, new onset transient or persistent neurological symptoms), full data should be submitted within 72 hours by the sub-center and should include:

- Physical examination: vital signs (bilateral brachial artery systolic/diastolic pressure, heart rate, pulse) and neurological examination.
- NIHSS score.
- Head CT or MRI (sequences T1+T2+FLAIR+DWI/ADC+MRA are required, SWI/GRE-T2 if qualified) shall be performed and stored in DICOM form, which should be sent to the radiology department for further assessment; CT is enough for the diagnosis of hemorrhagic stroke while MRI is strongly recommended for ischemic events.
- If suspected cardiovascular events occur, cardiac function should be assessed (including ECG, serum cardiac enzyme and troponin). If myocardial infarction is highly suspected, data should be collected and uploaded to CRO for further assessment within 72 hours since the incident.
- Collect information including the use of study drugs, concurrent medication and adverse events.

10.10 Adverse event handling process

10.10.1 Adverse event/serious adverse event handling principles

- Determine whether the adverse event is related to the study drug: if it is related to the study drug, the investigator should suspend the study drug, let the clinician determine the treatment plan according to the subject's condition, closely observe the subject's condition, and decide whether to resume the study drug according to the subject's recovery. If the adverse event is not related to the study drug, the patient could continue the study drug, and the clinician need determine the treatment plan according to the subject's condition.
- Collect information on which the event was based, such as imaging images and reports, test results, etc.; complete the relevant event form.
- If the event is a serious adverse event, it should be reported in accordance with the serious adverse event handling process.
- If it is a bleeding event, the investigator need to confirm the severity of bleeding according to the GUSTO criteria in the study protocol and fill out the relevant event report form.
- Complete the follow-up visits and record the relevant information.

10.10.2 Reporting of serious adverse events

Investigators needn’t report serious adverse events for the following conditions that occur after the subject's first dose of the study drug through the study follow-up period.

- Life-threatening, permanent or severe disability or loss of function, or hospitalization or prolonged

hospitalization resulting from a recurrence of ischemic stroke.

- Life-threatening, permanent or severe disability or loss of function, or hospitalization or prolonged hospitalization resulting from a secondary endpoint event as defined in this study.
- Congenital anomalies or birth defects discovered after enrollment.
- Subjects who are hospitalized or have an extended hospital stay due to transfer to a rehabilitation unit, etc. for long-term post-stroke rehabilitation.

Serious adverse events need to be reported by the investigator if any of the following occur after the subject's first dose of the study drug through the study follow-up period.

- All-cause Death.
- Life-threatening, permanent or severe disability or loss of function, or hospitalization or prolonged hospitalization resulting from a recurrence of hemorrhagic stroke.
- Other unanticipated serious adverse events.

If any of these serious adverse events are reported, the investigator must collect the relevant information, complete a Serious Adverse Event Report Form and report it to the Ethics Committee within 24 hours of being informed.

10.11 Table of study procedure

Measures	Screening and randomization (1st visit)	Treatment period				Observation period	
		Day7 (2nd visit)	Day 14 or Hospital discharge (3rd visit)	Day 21±2 (4th visit)	Day 90±7 (5th visit)	6th month± 14 days (6th visit)	12th month ± 14 days (7th visit)
Inclusion/Exclusion	√						
Informed consent	√						
Onset	√						
First blood pressure and auxiliary examination	√						
Basic information	√						
Past medical history	√						
Personal/family history	√						
Medication history before randomization	√						
Physical examination	√						
$ABCD^2$ for TIA only	√						
NIHSS	√	√	√		√		
mRS	√				√		√
EQ-5D scale	√				√		√
Imaging assessment for enrollment	√[1]						
Laboratory test	√[2]	√[3]			√[3]		
ECG	√	√					
Randomization	√						
First medication time after randomization	√						
Primary diagnosis	√						
Final diagnosis			√				
Drug dispense/Retrieve	√			√	√		
Blood specimen (fasting)	√[4]				√[4]		
HR-MRI subgroup	√[5]				√[5]		
Endpoints		√	√	√	√	√	√
AEs/SAEs		√	√	√	√	√	√
Drug compliance		√	√	√	√		

Concurrent medication		√	√	√	√	√	√

1. Imaging assessments include brain histology and assessments of intracranial and extracranial arteries. Brain histological examination includes head MRI or CT scan for diagnosis of TIA and cerebral infarction, and mode assessment for cerebral infarction (single infarction or multiple infarctions). If MRI cannot be completed before randomization, it must be done within 7 days after randomization. The brain MRI must include the T1+T2+FLAIR+DWI/ADC+MRA sequences. GRE-T2* or SWI shall be done according to the conditions of each sub-center. If head MRI is completed, head CT is not required. Intracranial and extracranial artery assessments are used for screening. Intracranial artery assessment examinations include any test of MRA、CTA、DSA. Extracranial artery assessment examinations include any test of neck vascular ultrasound, CEMRA, CTA on the aortic arch, DSA. **Note**: If a patient applying only one assessment for intracranial or extracranial artery is found to meet the inclusion criteria, he or she can be included without performing both intracranial and extracranial artery assessment. For example, if a TIA patient has only finished a neck vascular ultrasound, and finds out the responsibility artery stenosis ≥50% and is in accord with the standard set of circumstances, the patient can be recruited without assessment of intracranial arteries. **All the above assessments need to be completed within 72 hours after randomization and needs no repetition if they are completed before randomization. The cervical vascular ultrasound should be uploaded in original photo, other image data should be uploaded to Beijing Tiantan hospital with DICOM format.**
2. Emergency laboratory assessment should be finished at screening, including emergency blood routine, emergency liver function (serum transaminases), emergency renal function (creatinine, urea nitrogen) and emergency blood clotting. The fasting laboratory tests (including biochemical kits, glycosylated hemoglobin, homocysteine, etc.) should be completed in the early morning of the second day after randomization. If not, 72 hours after randomization is the final deadline.
3. Routine blood counts, biochemical panel (including alanine transaminase, aspartate aminotransferase, alkaline phosphatase, creatine kinase, creatine kinase-MB) and coagulation function should be performed on day7 (If the test had been completed within 24 hours of screening or randomization, there was no requirement to review it).Routine blood counts and biochemical panel (including alanine transaminase, aspartate aminotransferase, alkaline phosphatase, creatine kinase, creatine kinase-MB, low-density lipoprotein cholesterol, high-density lipoprotein cholesterol, total cholesterol, triglyceride) should be performed on day 90±7 after randomization to monitor hepatotoxicity and muscle toxicity of statin.
4. Fasting venous blood should be collected within 24 hours of randomization (If not, 72 hours after randomization is the final deadline) and 90±7 days after randomization.
5. High-resolution MRI examinations should be performed for patient enrolled in the subgroup of high-resolution MRI, within 24 hours and 90 days ± 7 hours after randomization.

11. Ethics and Regulations

11.1 Ethical principles

This clinical study will follow the rules of the Declaration of Helsinki. This clinical Study will be conducted in accordance with the principles laid down by the 18th World Medical Assembly, the ICH guidelines for Good Clinical Practice. This protocol has been reviewed by an independent Ethics Committee before being conducted. The investigators should fully inform the subjects of all pertinent aspects, including the aim, procedures and risk of this study. A written information is required giving approval opinion. All patients should be informed to the fullest extent possible about the study, and that they have the right to withdraw from this study. The signed and dated written Informed Consent should be well-kept for further investigation. Personal privacy and data confidentiality should be protected during this study.

11.2 Laws and regulations

This clinical study will be conducted in compliance with all international laws and regulations, and national laws and regulations of the countries in which the clinical study is performed, as well as any applicable guidelines.

11.3 Informed consent procedures

The investigator should fully inform the subjects of all pertinent aspects of the clinical study permitted by the Ethics Committee. All subjects should be informed to the fullest extent possible about the study, in a language and terms they are able to understand. If the subject can’t express his own will, the above introduction and explanation should be provided to legal representative of the subject.

Prior to a subject’s participation in the Clinical Trial, a written Informed Consent Form should be signed, name filled in and personally dated by the patient or by the patient’s legally acceptable representative, and by the person who conducted the informed consent discussion. If the subjects or legal representative can't read, an impartial witness must be present throughout the process of witness. A copy of the signed and dated written Informed Consent Form will be provided to the patient.

Approval and favorable opinion from the Sponsor is required before the informed consent being submitted to the Ethics Committee.

11.4 Institutional Review Board/Independent Ethics Committee (IRB/IEC)

Before the trial, the Investigator or the Sponsor must submit this Clinical Trial Protocol to the appropriate Ethics Committee (IRB/IEC), and is required to forward to the Sponsor a copy of the written and dated approval/favorable opinion signed by the Chairman with Ethics Committee (IRB/IEC) composition.

The Clinical Trial (study number, Clinical Trial Protocol title and version number), the documents reviewed (Clinical Trial Protocol, Informed Consent Form, Investigator’s Brochure, Investigator’s CV, etc.), the list of voting members and their qualifications and the date of the review should be stated on the written (IRB/IEC) approval/favorable opinion.

Investigational Product must not be released to sub-centers prior to the start of the trial. The Clinical Trial must not begin until a copy of the written and dated approval/favorable opinion is received by the Sponsor.

During the Clinical Trial, any amendment or modification to the Clinical Trial Protocol should be submitted to the Ethics Committee (IRB/IEC) , and execute after being approved. Any event likely to affect the safety of patients or the continuation of the Clinical Trial, particularly any change in safety outcomes, should also be reported to IRB/IEC. All updates to the Investigator's Brochure will be sent to the Ethics Committee (IRB/IEC).

If requested, a progress report and a summary of the Clinical Trial's outcome at the end of the Clinical Trial shall be sent to the Ethics Committee (IRB/IEC) annually.

12. Research Organization

12.1 Research organization

- **Main investigator**

Prof. Yilong Wang， Department of Neurology, Beijing Tiantan Hospital, Capital Medical University.

Prof. Yongjun Wang， Department of Neurology, Beijing Tiantan Hospital, Capital Medical University.

- **Steering Committee**

- Steering Committee will provide scientific and strategic recommendations, and will be responsible for the design and conduct of the study and publication of the main study results.
- Steering Committee will maintain contact with investigators to ensure high quality data collection, conduct and management.
- Steering Committee will approve the main protocol and guidelines before conducting the study
- Steering Committee will discuss and report progression of the study in telephone conference or face-to-face conference periodically.
- The member of the Steering Committee and their duties will be described and confirmed before the study being conducted.

- **Executive Committee**

Executive Committee will evaluate the progression and blinded-data, and give appropriate advice to the study. Main decisions should be made in face-to-face conferences of the Executive Committee. The member of the Executive Committee and their duties will be described and confirmed before the study being conducted.

- **Data and Safety Monitoring Board (DSMB)**

To meet the study's highest ethical responsibility and safety to its subjects, a data safety monitoring board (DSMB) will monitor results during the study periodically. The board includes academic members such as biostatisticians, and they have no formal involvement or conflict of interest with the subjects, the investigators or the clinical sites. The members of the DSMB and their duties will be described and confirmed before the study being conducted. A report should be submitted to the chairman of the committee immediately after the DSMB conference.

- **Event Adjudication Committee**

Clinical Endpoint events (stroke, MI, death, major bleeding) and safety outcomes will be reviewed by independent specialists (in neurology and cardiology). The member of the Event Adjudication Committee (member, qualification, duties) should be qualified by Steering and Executive Committee.

The radiology information of the clinical event should be first assessed locally and meanwhile the original picture should be submitted to the main investigator. The adjudication report should include radiology report.

12.2 Qualifications and Training

The Executive Committee should make sure every clinical center receives GCP training, as well as screening, follow-up and outcome evaluation training (such as NIHSS, mRS). The main investigator and coordinator of each sub-center should finish the training and be certified before the study as required.

ALL investigators should receive training and be certified

- Study Procedure
- Guidelines for diagnosis of ischemic stroke and TIKA
- $ABCD^2$ score
- Inclusion and Exclusion Criteria
- mRS scoring
- NIHSS scoring
- TOAST classification
- Primary adjudication of the outcomes event
- Definition of GUSTO bleeding criteria
- EQ-5D scale
- Collection and procession of biologic specimen (blood)
- Collection of radiology information in DICOM form

Investigators and coordinators in each clinical site must be trained and qualified before conducting the study. PI of the study center and the main investigator should attend a periodically telephone conference to solve the problem meted in the study. Qualification shall be certificated by training center.

Detailed manuals will be used as the main references for the investigators. It serves as the instruction of the training, and will be updated on the “INSPIRES” website throughout the entire research period. Executive Committee and coordinators will keep contact with investigators through telephone, e-mail and fax, to help them solve the problem. The INSPIRES Executive and Monitoring Committee will answer the problems each sub-center meets and deliver it to all sub-centers together.

The Executive Committee will inspect all clinical centers in the field and make sure the accuracy and integrity of the data. Every clinical center shall be inspected at least one time during the study and shall be inspected more if the quality of data is poor or there is problem in subject inclusion.

13. Quality Control and Quality Assurance

13.1 Responsibilities of investigators

The Investigator(s) undertake(s) to perform the Clinical Trial in accordance with this Clinical Trial Protocol, ICH guidelines for Good Clinical Practice and the applicable regulatory requirements.

The Investigator is required to ensure compliance with all procedures required by the Clinical Trial Protocol and with all study procedures provided by the Sponsor (including security rules). The Investigator agrees to provide reliable data and all information requested by the Clinical Trial Protocol (with the help of the Case Report Form [CRF], Discrepancy Resolution Form [DRF] or other appropriate instrument) in an accurate and legible manner according to the instructions provided and to ensure direct access to source documents by Sponsor representatives.

The Investigator may appoint such other individuals as he/she may deem appropriate as Sub-Investigators to assist in the conduct of the Clinical Trial in accordance with the Clinical Trial Protocol. All Sub-Investigators shall be appointed and listed in a timely manner. The Sub-Investigators will be supervised by and under the responsibility of the Investigator. The Investigator will provide them with a copy of the Clinical Trial Protocol and all necessary information. The sponsors shall be responsible for the health authority, taking any possible methods to ensure the appropriate management of the clinical study, these methods including Ethics, compliance of the study plan, the integrity and validity of the clinical data.

13.2 Study Monitoring

The main responsibility of the Monitoring Committee is to assist the investigators and sponsors in the all aspects of the clinical study in a highly ethics, scientific, professional and normalization manner. According to the ICH guidelines for Good Clinical Practice, the Monitoring Team must check the Case Report Form entries against the source documents.

At regular intervals during the Clinical Trial, the site will be contacted, through monitoring visits, letters or telephone calls, by a representative of the Monitoring Team to review study progress, investigator and patient compliance with Clinical Trial Protocol requirements and any emergent problems. During these monitoring visits, the following but not exhaustive list of points will be scrutinized with the investigator: patient informed consent, patient recruitment and follow-up, Serious Adverse Event documentation and reporting, Investigational Product allocation, patient compliance with the Clinical Trial Protocol and the Investigational Product regimen, concomitant therapy use and quality of data.

14. Data Preservation

All CRFs and imagine data should be delivered to data management center by the inspector and should be checked and received by the directors of the data storage center. All CRFs which should be kept in this center should be completed entirely in a neat, legible manner to ensure accurate interpretation of data. After the data entry was completed, the CRFS should be saved by the research center.

15. Safety Monitoring of Data

Corresponding data security monitoring plan will be developed based on the risk of the Clinical Trial. All adverse events will be recorded in detail, properly handled and tracked until events are resolved properly or the condition becomes stable. Serious adverse events and other events should be reported to the ethics committee, competent authorities, sponsors, and drug supervision and management departments according to applicable regulations. The main investigators will regularly review all adverse events. Researchers meeting will be held to assess the risks and benefits of the study if necessary. For studies that exceeds the minimum risk, independent data monitor will be assigned to monitor the research data. An independent data safety inspection committee will be established for high-risk research to monitor the accumulated safety data and effectiveness, making recommendations on whether to proceed with the proposal or not.

During the clinical trial, the subject's data should be collected anonymously on the CRFs. Subjects can only be identified as the subject number and the “pinyin” abbreviation of name. Due to security or administrative instructions, those who accidentally knows the identity of the subject should be responsible for confidentiality along with the investigators. Informed Consent Forms allow authorized sponsors, ethical committees, and authoritative agencies to directly access relevant original data on CRFs (such as patient medical records, appointment records, original laboratory records, etc.). The above personnel should follow the professional secrecy regulations and must keep confidential data of patients’ personally identifiable information or medical information.

16. Statistical Processing

Main null hypothesis

1) In patients with acute mild ischemic stroke or high-risk TIA patients attributed to extracranial or intracranial atherosclerosis treated within 72 hours of ictus, there is no difference in the risk of a new stroke within 90 days between subjects with intensive antiplatelet therapy and those with standard antiplatelet therapy.
2) In patients with acute mild ischemic stroke or high-risk TIA patients attributed to extracranial or intracranial atherosclerosis treated within 72 hours of ictus, there is no difference in the risk of a new stroke within 90 days between subjects with immediate intensive statin therapy (atorvastatin 80mg/d) and delayed intensive statin therapy (atorvastatin 40mg/d).
3) In patients with acute mild ischemic stroke or high-risk TIA patients attributed to extracranial or intracranial atherosclerosis treated within 72 hours of ictus, there is no difference in the risk of a new stroke within 90 days between subjects with intensive antiplatelet combined with immediate intensive statin therapy (atorvastatin 80mg/d) and standard antiplatelet combined with delayed intensive statin therapy (atorvastatin 40mg/d).

16.1 Estimate Sample Size

The minimal sample size for the trial is determined by the necessity that a clinically meaningful difference in effectiveness between treatment and control groups has to be detected. Based on previous studies, the risk of new stroke during 90 days is presumed to be 11.5% in the group with standard antiplatelet therapy (with half delayed intensive statin therapy and half early high-intensity statin therapy) and 11.5% in the delayed intensive statin therapy group (with half standard antiplatelet therapy and half dual antiplatelet therapy) and 13% in the group with standard antiplatelet therapy plus delayed intensive statin therapy, intensive antiplatelet therapy and immediate intensive statin therapy can reduce this risk by 22%, and the effects of intensive antiplatelet and lipid lowering therapy will be similar and additive. With a sample size of 6100 subjects, a two-sided α of 0.05 and 5% loss to follow up, we will have 97% power to detect that the risk is decreased by 35% in the group with dual antiplatelet therapy plus immediate intensive statin therapy compared to standard antiplatelet therapy plus delayed intensive therapy, and 80% power to detect that the risk is reduced by 20% by intensive antiplatelet therapy compared to standard antiplatelet therapy, and immediate intensive statin compared to delayed intensive statin therapy, respectively.

Table1 For different risk rates (12% to 14%) of the results in the control group, the sample size required for observing intensive antiplatelet combining intensive statin therapy

Power	A single set of sample size (no expulsion)	A single set of sample size (5% expulsion rate)	Four groups of sample size	The stroke recurrence rate in treatment group	The stroke recurrence rate in control group	Relative risk
0.966	1448	1525	6100	0.078	0.120	0.65

0.975	1448	1525	6100	0.085	0.130	0.65
0.985	1448	1525	6100	0.091	0.140	0.65
0.824	1448	1525	6100	0.096	0.130	0.74

Table2 For different risk rates (12% to 14%) of the results in the control group, the sample size required for the marginal effect analysis of observed intensive antiplatelet combining intensive statin therapy

Power	A single set of sample size (no expulsion)	A single set of sample size (5% expulsion rate)	Four groups of sample size	The stroke recurrence rate in treatment group	The stroke recurrence rate in control group	Relative risk
0.807	2896	3050	6100	0.0930	0.1157	0.80

Explanations of data sources on recurrence risk and the risk of stroke recurrence after treatment:

At present, evidence in early risks of recurrent stroke in high-risk symptomatic intracranial or extracranial artery stenosis patients is still lacking. The data currently available for reference include: a) In the studies of WASID and SAMMPRIS, the recurrence risk of stroke in the medication treatment group with symptomatic intracranial artery stenosis (stenosis rate > 70%) were 21.9% and 12.6% at 1 year, respectively. [49,50] The recurrence risk of 12.6% at 1 year in SAMMPRIS research was based on the combination of dual anti-platelet therapy, intensive lipid-lowering and blood-pressure-lowering therapy. Therefore, for the recurrence risk of the standard therapy group, 13% was a conservative figure in our study. [51] b) The subgroup study of the CHANCE Trial has revealed a recurrence risk of 12.5% in patients with intracranial arterial stenosis (stenosis rate > 50%) at day 90.[4] In the CHANCE trial, patients with intracranial arterial stenosis associated with multiple infarcts had a recurrent stroke risk of a striking number of 18% at 90 days.[21] Therefore, as the recurrence risk of the standard therapy group, 13% was a conservative data in our study.

Concerning that dual anti-platelet therapy reduces the risk of recurrence of 22%, the CHANCE study confirmed that dual anti-platelet therapy decreased the risk of stroke recurrence by 90 days for TIA and minor stroke by 32% compared with anti-platelet monotherapy. [15] In the CHANCE subgroup, compared with anti-platelet monotherapy, dual anti-platelet therapy decreased the relative risk of stroke recurrence by 21% in patients with intracranial arterial stenosis, and by 50% in patients with multiple cerebral infarctions. [4,22] Therefore, with dual anti-platelet therapy, our estimate of a relative reduction of 22% in recurrent stroke risk was a conservative data in our study.

Concerning the source of evidence for intensive lipid-lowering combined with intensive antiplatelet therapy decreases the risk of stroke by 35%, a comparative study of SAMMPRIS and WASID before and after pointed out that the risks of recurrent stroke were 21.9% and 12.6% in the intensive drug therapy group and anti-platelet monotherapy group at 1 year, respectively. The risk was decreased by about 50%.

Therefore, the estimated reduction of recurrence risk by 35% was a conservative data in our study. [50]

16.2 Data collection and record

All requirements of the test program in the CRF table must be provided, and the parts not provided should be explained.

Electronic Data Capture (EDC) System will be used to input the data in CRF. All test protocol requirements in the CRF table must be provided, and the part not provided should be explained. The administrator will check the data and correct the obvious errors. Other errors or missing data in the project will be returned to the research center. The research center will verify the original information and relevant information to answer and correct the data query form. The process of queries, responses and corrections will be completed and recorded through the EDC system.

As for paper-based information such as incident report forms, researchers must fill in the data requested by the test program in black or blue pen in carbonless copies of the case report forms to ensure that the copy is clear and readable. If the information needs to be modified, it should not be altered or overwritten. The correct information should be written beside the original information and signed and dated by the modifier. The inspector will make sure the accuracy and completeness of the case report forms, and guide the researchers to make necessary corrections and supplementary questions.

After receiving the data, the sponsor will carry out computer data analysis, additional requirements may arise, and researchers must answer these requirements by confirming or modifying data. The content of the corrections will be added to the EDC through the investigator.

16.3 statistical analysis

This section is an overview of the statistical analysis. It provides general provisions on how to collect data and display data in clinical studies. The final version of the statistical analysis plan (SAP) will be completed before the database is locked. SAP will specify all "pre-defined, planned analyzes".

16.3.1 Statistical analysis of data sets

Full Analysis Set (FAS): The full analysis set is the main efficacy evaluation population of this study, and all valid variables will be analyzed by FAS. According to the basic principle of intention-to-treat (ITT) analysis, all subjects randomized into groups and those with more than one medication record and efficacy evaluation will be included in the full analysis set. When choosing FAS to analyze, the estimate of the missing value, the last observation carried forward (LOCF) estimation method can be used.

Per Protocol Set (PPS): Includes all subjects who complete the protocol or have no serious breaches of the trial. The exact definition of a serious violation of the program will be finalized at the time of data review and may generally include the following situations (but not limited to these cases): failing to meet the main inclusion criteria, serious disturbance of drug efficacy after treatment, poor compliance, exceeding the time window of following-up and so on. PPS is the secondary analysis of the effectiveness of the crowd, but if the results are inconsistent with the whole analysis set, detailed analysis of the

inconsistent results are needed.

Safety Data Set (SS): A safety data set is defined as a subject that receives drug treatment at least once. In the entire safety analysis, patients with incorrect treatment (for example, randomized to the standard antiplatelet combining delayed intensive statin therapy groups were given enhanced antiplatelet therapy) would be assigned to the actual treatment group.

16.3.2 Statistical analysis methods

16.3.2.1 The balance analysis of basic values (comparison between groups of baseline indicators)

1) The measurement data will be using t test or Wilcoxon rank sum test;

2) Enumeration data between groups will be compared with chi-square test, Fisher's exact probability method or Wilcoxon rank sum test, when appropriate.

16.3.2.2Efficacy analysis

1) Primary outcome:

Survival curves will be estimated for the primary outcome using the Kaplan-Meier procedure and compared using a Cox regression model Wald test, stratified by the opposite arm of the factorial design. First, marginal effects will be compared between intensive and standard antiplatelet therapy, and also between immediate intensive statin therapy (atorvastatin 80mg/d) and delayed intensive statin therapy (atorvastatin 40mg/d). As these are independent comparisons, they have no effect on the type I error rate. For both treatment comparisons, the primary outcome of stroke will be judged to be statistically significant if the treatment test p-value is < 0.05. Second, if either intensive antiplatelet or immediate intensive statin treatment (atorvastatin 80mg/d) is statistically significant, the effects between combined intensive antiplatelet and immediate intensive statin treatment (atorvastatin 80mg/d) and combined standard antiplatelet and delayed intensive statin treatment (atorvastatin 40mg/d) will be tested. Cox regression model stratified by the opposite arm of the factorial design, will be used in the curative effect assessment and evaluation between the subgroups.

2) Secondary outcomes:

a. Kaplan-Meier curve will be used to simulate the cumulative risk of 90-day follow-up combined with secondary endpoint events such as vascular events, ischemic stroke, TIA, myocardial infarction, vascular death and all-cause death. Cox proportional risk model will be used to calculate HR and the 95% confidence interval and the Log-rank test would be used to evaluate the efficacy;
b. For ordinal stroke or TIA, ordinal logistic regression will be used to calculate OR and 95% confidence interval. For the poor functional outcome (mRS score 2-6) and early neurological deficits (increase in NIHSS score at 7days) indexes, logistic regression will be used to calculate OR and 95% confidence interval.
c. The validity of the extreme value would be examined, and the sensitivity analysis would be carried out.

3) Safety analysis

The overall of safety assessment is all used test medications and the safety follow-up case should be

recorded at least once. The safety evaluation data includes adverse reactions observed during the trial and changes in laboratory data before and after treatment.

Safety evaluation will be analyzed using safety data set.

Moderate to severe bleeding, intracranial hemorrhage, and overall mortality will be calculated using the Kaplan-Meier curve to simulate the 3-month cumulative risk, and the Cox proportional hazards model to calculate the HR and 95% confidence interval.

For other adverse events and laboratory abnormalities, the cases which was normal before the treatment and abnormal after the treatment would be mainly analyzed and listed, in order to the comparison of differences before and after treatment.

17. Confidentiality and Announcement of Results

The main investigators possess complete intellectual property rights. Information of the subjects are strictly protected through the research process and data analysis process. The Executive Committee will publish the results in accordance with the regulations and procedures. After the database is locked, the test results will be published as soon as possible. With 6100 patients, this study will investigate detailed data of the treatments, medical measures and clinical outcomes. The biostatistics experts participating in the study have the right to access the data set but are unable to identify any patient in this trial. Finally, the comma-separated text format of the disk storage data (including the text format of the data dictionary) will be sent to the intention of the third party to deal with.

REFERENCE:

1. Lovett JK, Coull AJ, Rothwell PM. Early risk of recurrence by subtype of ischemic stroke in population-based incidence studies. *Neurology*. 2004;62:569-573. doi: 10.1212/01.wnl.0000110311.09970.83
2. Petty GW, Brown RD, Jr., Whisnant JP, Sicks JD, O'Fallon WM, Wiebers DO. Ischemic stroke subtypes : a population-based study of functional outcome, survival, and recurrence. *Stroke*. 2000;31:1062-1068. doi: 10.1161/01.str.31.5.1062
3. Amarenco P, Lavallee PC, Labreuche J, Albers GW, Bornstein NM, Canhao P, Caplan LR, Donnan GA, Ferro JM, Hennerici MG, et al. One-Year Risk of Stroke after Transient Ischemic Attack or Minor Stroke. *The New England journal of medicine*. 2016;374:1533-1542. doi: 10.1056/NEJMoa1412981
4. Liu L, Wong KS, Leng X, Pu Y, Wang Y, Jing J, Zou X, Pan Y, Wang A, Meng X, et al. Dual antiplatelet therapy in stroke and ICAS: Subgroup analysis of CHANCE. *Neurology*. 2015;85:1154-1162. doi: 10.1212/wnl.0000000000001972
5. Ois A, Cuadrado-Godia E, Rodriguez-Campello A, Giralt-Steinhauer E, Jimenez-Conde J, Lopez-Cuina M, Ley M, Soriano C, Roquer J. Relevance of stroke subtype in vascular risk prediction. *Neurology*. 2013;81:575-580. doi: 10.1212/WNL.0b013e31829e6f37
6. Liu X, Xu G, Wu W, Zhang R, Yin Q, Zhu W. Subtypes and one-year survival of first-ever stroke in Chinese patients: The Nanjing Stroke Registry. *Cerebrovascular diseases (Basel, Switzerland)*. 2006;22:130-136. doi: 10.1159/000093241
7. Kasner SE, Chimowitz MI, Lynn MJ, Howlett-Smith H, Stern BJ, Hertzberg VS, Frankel MR, Levine SR, Chaturvedi S, Benesch CG, et al. Predictors of ischemic stroke in the territory of a symptomatic intracranial arterial stenosis. *Circulation*. 2006;113:555-563. doi: 10.1161/circulationaha.105.578229
8. Wang Y, Zhao X, Liu L, Soo YO, Pu Y, Pan Y, Wang Y, Zou X, Leung TW, Cai Y, et al. Prevalence and outcomes of symptomatic intracranial large artery stenoses and occlusions in China: the Chinese Intracranial Atherosclerosis (CICAS) Study. *Stroke*. 2014;45:663-669. doi: 10.1161/strokeaha.113.003508
9. Kang DW, Latour LL, Chalela JA, Dambrosia J, Warach S. Early ischemic lesion recurrence within a week after acute ischemic stroke. *Ann Neurol*. 2003;54:66-74. doi: 10.1002/ana.10592
10. Wen HM, Lam WW, Rainer T, Fan YH, Leung TW, Chan YL, Wong KS. Multiple acute cerebral infarcts on diffusion-weighted imaging and risk of recurrent stroke. *Neurology*. 2004;63:1317-1319. doi: 10.1212/01.wnl.0000140490.22251.b6
11. Kang DW, Chu K, Ko SB, Kwon SJ, Yoon BW, Roh JK. Lesion patterns and mechanism of ischemia in internal carotid artery disease: a diffusion-weighted imaging study. *Arch Neurol*. 2002;59:1577-1582. doi: 10.1001/archneur.59.10.1577

12. Lee DK, Kim JS, Kwon SU, Yoo SH, Kang DW. Lesion patterns and stroke mechanism in atherosclerotic middle cerebral artery disease: early diffusion-weighted imaging study. *Stroke*. 2005;36:2583-2588. doi: 10.1161/01.Str.0000189999.19948.14
13. Wong KS, Gao S, Chan YL, Hansberg T, Lam WW, Droste DW, Kay R, Ringelstein EB. Mechanisms of acute cerebral infarctions in patients with middle cerebral artery stenosis: a diffusion-weighted imaging and microemboli monitoring study. *Ann Neurol*. 2002;52:74-81. doi: 10.1002/ana.10250
14. Kernan WN, Ovbiagele B, Black HR, Bravata DM, Chimowitz MI, Ezekowitz MD, Fang MC, Fisher M, Furie KL, Heck DV, et al. Guidelines for the prevention of stroke in patients with stroke and transient ischemic attack: a guideline for healthcare professionals from the American Heart Association/American Stroke Association. *Stroke*. 2014;45:2160-2236. doi: 10.1161/str.0000000000000024
15. Wang Y, Wang Y, Zhao X, Liu L, Wang D, Wang C, Wang C, Li H, Meng X, Cui L, et al. Clopidogrel with aspirin in acute minor stroke or transient ischemic attack. *The New England journal of medicine*. 2013;369:11-19. doi: 10.1056/NEJMoa1215340
16. Wong KS, Wang Y, Leng X, Mao C, Tang J, Bath PM, Markus HS, Gorelick PB, Liu L, Lin W, et al. Early dual versus mono antiplatelet therapy for acute non-cardioembolic ischemic stroke or transient ischemic attack: an updated systematic review and meta-analysis. *Circulation*. 2013;128:1656-1666. doi: 10.1161/circulationaha.113.003187
17. Hong KS, Lee SH, Kim EG, Cho KH, Chang DI, Rha JH, Bae HJ, Lee KB, Kim DE, Park JM, et al. Recurrent Ischemic Lesions After Acute Atherothrombotic Stroke: Clopidogrel Plus Aspirin Versus Aspirin Alone. *Stroke*. 2016;47:2323-2330. doi: 10.1161/strokeaha.115.012293
18. Markus HS, Droste DW, Kaps M, Larrue V, Lees KR, Siebler M, Ringelstein EB. Dual antiplatelet therapy with clopidogrel and aspirin in symptomatic carotid stenosis evaluated using doppler embolic signal detection: the Clopidogrel and Aspirin for Reduction of Emboli in Symptomatic Carotid Stenosis (CARESS) trial. *Circulation*. 2005;111:2233-2240. doi: 10.1161/01.Cir.0000163561.90680.1c
19. Wong KS, Chen C, Fu J, Chang HM, Suwanwela NC, Huang YN, Han Z, Tan KS, Ratanakorn D, Chollate P, et al. Clopidogrel plus aspirin versus aspirin alone for reducing embolisation in patients with acute symptomatic cerebral or carotid artery stenosis (CLAIR study): a randomized, open-label, blinded-endpoint trial. *Lancet Neurol*. 2010;9:489-497. doi: 10.1016/s1474-4422(10)70060-0
20. Wang X, Lin WH, Zhao YD, Chen XY, Leung TW, Chen C, Fu J, Markus H, Hao Q, Wong KS. The effectiveness of dual antiplatelet treatment in acute ischemic stroke patients with intracranial arterial stenosis: a subgroup analysis of CLAIR study. *Int J Stroke*. 2013;8:663-668. doi: 10.1111/j.1747-4949.2012.00828.x
21. Pan Y, Meng X, Jing J, Li H, Zhao X, Liu L, Wang D, Johnston SC, Wang Y, Wang Y. Association of multiple infarctions and ICAS with outcomes of minor stroke and TIA. *Neurology*. 2017;88:1081-1088. doi: 10.1212/wnl.0000000000003719

22. Jing J, Meng X, Zhao X, Liu L, Wang A, Pan Y, Li H, Wang D, Johnston SC, Wang Y, et al. Dual Antiplatelet Therapy in Transient Ischemic Attack and Minor Stroke With Different Infarction Patterns: Subgroup Analysis of CHANCE Randomized Clinical Trial. *JAMA Neurol*. 2018. doi: 10.1001/jamaneurol.2018.0247
23. Johnston SC, Amarenco P, Albers GW, Denison H, Easton JD, Evans SR, Held P, Jonasson J, Minematsu K, Molina CA, et al. Ticagrelor versus Aspirin in Acute Stroke or Transient Ischemic Attack. *The New England journal of medicine*. 2016;375:35-43. doi: 10.1056/NEJMoa1603060
24. Amarenco P, Albers GW, Denison H, Easton JD, Evans SR, Held P, Hill MD, Jonasson J, Kasner SE, Ladenvall P, et al. Efficacy and safety of ticagrelor versus aspirin in acute stroke or transient ischaemic attack of atherosclerotic origin: a subgroup analysis of SOCRATES, a randomized, double-blind, controlled trial. *Lancet Neurol*. 2017;16:301-310. doi: 10.1016/s1474-4422(17)30038-8
25. Amarenco P, Bogousslavsky J, Callahan A, 3rd, Goldstein LB, Hennerici M, Rudolph AE, Sillesen H, Simunovic L, Szarek M, Welch KM, et al. High-dose atorvastatin after stroke or transient ischemic attack. *The New England journal of medicine*. 2006;355:549-559. doi: 10.1056/NEJMoa061894
26. Sillesen H, Amarenco P, Hennerici MG, Callahan A, Goldstein LB, Zivin J, Messig M, Welch KM. Atorvastatin reduces the risk of cardiovascular events in patients with carotid atherosclerosis: a secondary analysis of the Stroke Prevention by Aggressive Reduction in Cholesterol Levels (SPARCL) trial. *Stroke*. 2008;39:3297-3302. doi: 10.1161/strokeaha.108.516450
27. Amarenco P, Benavente O, Goldstein LB, Callahan A, 3rd, Sillesen H, Hennerici MG, Gilbert S, Rudolph AE, Simunovic L, Zivin JA, et al. Results of the Stroke Prevention by Aggressive Reduction in Cholesterol Levels (SPARCL) trial by stroke subtypes. *Stroke*. 2009;40:1405-1409. doi: 10.1161/strokeaha.108.534107
28. Underhill HR, Yuan C, Zhao XQ, Kraiss LW, Parker DL, Saam T, Chu B, Takaya N, Liu F, Polissar NL, et al. Effect of rosuvastatin therapy on carotid plaque morphology and composition in moderately hypercholesterolemic patients: a high-resolution magnetic resonance imaging trial. *American heart journal*. 2008;155:584.e581-588. doi: 10.1016/j.ahj.2007.11.018
29. Du R, Cai J, Zhao XQ, Wang QJ, Liu DQ, Leng WX, Gao P, Wu HM, Ma L, Ye P. Early decrease in carotid plaque lipid content as assessed by magnetic resonance imaging during treatment of rosuvastatin. *BMC cardiovascular disorders*. 2014;14:83. doi: 10.1186/1471-2261-14-83
30. Lee JM, Robson MD, Yu LM, Shirodaria CC, Cunnington C, Kylintireas I, Digby JE, Bannister T, Handa A, Wiesmann F, et al. Effects of high-dose modified-release nicotinic acid on atherosclerosis and vascular function: a randomized, placebo-controlled, magnetic resonance imaging study. *J Am Coll Cardiol*. 2009;54:1787-1794. doi: 10.1016/j.jacc.2009.06.036
31. Corti R, Fayad ZA, Fuster V, Worthley SG, Helft G, Chesebro J, Mercuri M, Badimon JJ. Effects of lipid-lowering by simvastatin on human atherosclerotic lesions: a longitudinal study by high-resolution, noninvasive magnetic resonance imaging. *Circulation*. 2001;104:249-252.
32. Corti R, Fuster V, Fayad ZA, Worthley SG, Helft G, Smith D, Weinberger J, Wentzel J, Mizsei G, Mercuri M, et al. Lipid lowering by simvastatin induces regression of human atherosclerotic lesions:

two years' follow-up by high-resolution noninvasive magnetic resonance imaging. *Circulation*. 2002;106:2884-2887. doi: 10.1161/01.cir.0000041255.88750.f0

33. Sibley CT, Vavere AL, Gottlieb I, Cox C, Matheson M, Spooner A, Godoy G, Fernandes V, Wasserman BA, Bluemke DA, et al. MRI-measured regression of carotid atherosclerosis induced by statins with and without niacin in a randomized controlled trial: the NIA plaque study. *Heart*. 2013;99:1675-1680. doi: 10.1136/heartjnl-2013-303926
34. Oesterle A, Laufs U, Liao JK. Pleiotropic Effects of Statins on the Cardiovascular System. *Circulation research*. 2017;120:229-243. doi: 10.1161/circresaha.116.308537
35. Amin-Hanjani S, Stagliano NE, Yamada M, Huang PL, Liao JK, Moskowitz MA. Mevastatin, an HMG-CoA reductase inhibitor, reduces stroke damage and upregulates endothelial nitric oxide synthase in mice. *Stroke*. 2001;32:980-986. doi: 10.1161/01.str.32.4.980
36. Chen J, Zhang ZG, Li Y, Wang Y, Wang L, Jiang H, Zhang C, Lu M, Katakowski M, Feldkamp CS, et al. Statins induce angiogenesis, neurogenesis, and synaptogenesis after stroke. *Ann Neurol*. 2003;53:743-751. doi: 10.1002/ana.10555
37. D NC, Callaly EL, Duggan J, Merwick Á, Hannon N, Sheehan Ó, Marnane M, Horgan G, Williams EB, Harris D, et al. Association between acute statin therapy, survival, and improved functional outcome after ischemic stroke: the North Dublin Population Stroke Study. *Stroke*. 2011;42:1021-1029. doi: 10.1161/strokeaha.110.596734
38. Blanco M, Nombela F, Castellanos M, Rodriguez-Yáñez M, García-Gil M, Leira R, Lizasoain I, Serena J, Vivancos J, Moro MA, et al. Statin treatment withdrawal in ischemic stroke: a controlled randomized study. *Neurology*. 2007;69:904-910. doi: 10.1212/01.wnl.0000269789.09277.47
39. Hong KS, Lee JS. Statins in Acute Ischemic Stroke: A Systematic Review. *J Stroke*. 2015;17:282-301. doi: 10.5853/jos.2015.17.3.282
40. Tsivgoulis G, Katsanos AH, Sharma VK, Krogias C, Mikulik R, Vadikolias K, Mijajlovic M, Safouris A, Zompola C, Faissner S, et al. Statin pretreatment is associated with better outcomes in large artery atherosclerotic stroke. *Neurology*. 2016;86:1103-1111. doi: 10.1212/wnl.0000000000002493
41. Heo JH, Song D, Nam HS, Kim EY, Kim YD, Lee KY, Lee KJ, Yoo J, Kim YN, Lee BC, et al. Effect and Safety of Rosuvastatin in Acute Ischemic Stroke. *J Stroke*. 2016;18:87-95. doi: 10.5853/jos.2015.01578
42. Montaner J, Bustamante A, García-Matas S, Martínez-Zabaleta M, Jiménez C, de la Torre J, Rubio FR, Segura T, Masjuán J, Cánovas D, et al. Combination of Thrombolysis and Statins in Acute Stroke Is Safe: Results of the STARS Randomized Trial (Stroke Treatment With Acute Reperfusion and Simvastatin). *Stroke*. 2016;47:2870-2873. doi: 10.1161/strokeaha.116.014600
43. Yoshimura S, Uchida K, Daimon T, Takashima R, Kimura K, Morimoto T. Randomized Controlled Trial of Early Versus Delayed Statin Therapy in Patients With Acute Ischemic Stroke: ASSORT Trial (Administration of Statin on Acute Ischemic Stroke Patient). *Stroke*. 2017;48:3057-3063. doi: 10.1161/strokeaha.117.017623

44. Grundy SM, Stone NJ, Bailey AL, Beam C, Birtcher KK, Blumenthal RS, Braun LT, de Ferranti S, Faiella-Tommasino J, Forman DE, et al. 2018 AHA/ACC/AACVPR/AAPA/ABC/ACPM/ADA/AGS/APhA/ASPC/NLA/PCNA Guideline on the Management of Blood Cholesterol: A Report of the American College of Cardiology/American Heart Association Task Force on Clinical Practice Guidelines. *Circulation*. 2019;139:e1082-e1143. doi: 10.1161/cir.0000000000000625
45. HPS2-THRIVE randomized placebo-controlled trial in 25 673 high-risk patients of ER niacin/laropiprant: trial design, pre-specified muscle and liver outcomes, and reasons for stopping study treatment. *Eur Heart J*. 2013;34:1279-1291. doi: 10.1093/eurheartj/eht055
46. Chimowitz MI, Lynn MJ, Howlett-Smith H, Stern BJ, Hertzberg VS, Frankel MR, Levine SR, Chaturvedi S, Kasner SE, Benesch CG, et al. Comparison of warfarin and aspirin for symptomatic intracranial arterial stenosis. *The New England journal of medicine*. 2005;352:1305-1316. doi: 10.1056/NEJMoa043033
47. Wong KS, Chen C, Ng PW, Tsoi TH, Li HL, Fong WC, Yeung J, Wong CK, Yip KK, Gao H, et al. Low-molecular-weight heparin compared with aspirin for the treatment of acute ischaemic stroke in Asian patients with large artery occlusive disease: a randomized study. *Lancet Neurol*. 2007;6:407-413. doi: 10.1016/s1474-4422(07)70079-0
48. Barnett HJM, Taylor DW, Haynes RB, Sackett DL, Peerless SJ, Ferguson GG, Fox AJ, Rankin RN, Hachinski VC, Wiebers DO, et al. Beneficial effect of carotid endarterectomy in symptomatic patients with high-grade carotid stenosis. *The New England journal of medicine*. 1991;325:445-453.
49. Chimowitz MI, Lynn MJ, Derdeyn CP, Turan TN, Fiorella D, Lane BF, Janis LS, Lutsep HL, Barnwell SL, Waters MF, et al. Stenting versus aggressive medical therapy for intracranial arterial stenosis. *The New England journal of medicine*. 2011;365. doi: 10.1056/NEJMoa1105335
50. Chaturvedi S, Turan TN, Lynn MJ, Derdeyn CP, Fiorella D, Janis LS, Chimowitz MI. Do Patient Characteristics Explain the Differences in Outcome Between Medically Treated Patients in SAMMPRIS and WASID? *Stroke*. 2015;46:2562-2567. doi: 10.1161/strokeaha.115.009656
51. Waters MF, Hoh BL, Lynn MJ, Kwon HM, Turan TN, Derdeyn CP, Fiorella D, Khanna A, Sheehan TO, Lane BF, et al. Factors Associated With Recurrent Ischemic Stroke in the Medical Group of the SAMMPRIS Trial. *JAMA Neurol*. 2016;73:308-315. doi: 10.1001/jamaneurol.2015.4315

Appendix 1. The Definition of Cardiac-cerebral Vascular Events

Event	Definition
**Stroke**	A sudden onset of focal or global brain, spinal cord or retinal vascular damage, resulting in symptoms and signs of acute nervous system defects, which is associated with cerebral circulation disorders.
**Ischemic Stroke**	Acute focal cerebral or retinal infarction meeting any of the following conditions:<br>(1) Clinical signs or radiological evidence of acute onset of new focal neurological damage lasting longer than 24 hours, excluding other non-ischemic etiologies (such as brain infections, brain injuries, brain tumors, seizures, severe metabolic diseases, degenerative diseases of the nervous system and side effects of drugs);<br>(2) Acute cerebral or retinal ischemic events, excluding other non-ischemic etiologies, focal symptoms or signs sustaining less than 24 hours, but with radiological evidence of new infarction;<br>(3) The worsening of pre-existing symptoms of vascular origin ischemic stroke (i.e. NIHSS increased ≥ 4 based on primary ischemic stroke, excluding the hemorrhagic transformation after infarction or symptomatic intracranial hemorrhage) persisting for more than 24 hours, with or without deterioration of ischemic lesions on MRI or CT. Etiologic typing is based on the TOAST criteria.
**Transient Ischemic Attack**	Neurologic deficit caused by sudden focal brain or retinal ischemia that can fully recover, lasting less than 24 hours, with no evidence of new cerebral infarction on imaging (CT or MR). Other non-ischemic causes (such as brain infections, brain injuries, brain tumors, epilepsy, severe metabolic diseases, or degenerative neurological diseases) are excluded.
**Hemorrhagic Stroke**	Hemorrhagic stroke is defined as acute neurological dysfunction of the focal or whole brain or spinal cord caused by non-traumatic brain parenchymal, intraventricular, and subarachnoid hemorrhage.
**Hemorrhagic Transformation after Cerebral Infraction**	Any non-traumatic extravascular hemorrhage in acute / subacute infarcts, which could cause related neurological symptoms (symptomatic) or non-neurological symptoms (asymptomatic). Among them:<br>(1) <u>Ischemic stroke transformed into symptomatic hemorrhagic stroke:</u> The following two conditions must be met at the same time:<br>a. Imaging evidence (CT or MRI) of extravascular hemorrhage in the infarct area;<br>b. Symptoms are related to hemorrhagic transformation. The hemorrhagic transformation must be able to partially explain the clinical manifestations of the patient's neurological performance, such as:<br>i). Symptoms cannot be fully explained by infarct size and location

	ii). Clinical deterioration referring to an increase of 4 points or more in NIHSS score after the initial ischemic event, or death, which is caused by hemorrhagic transformation;<br>iii). Clinical symptoms caused by volume effect secondary to hemorrhagic transformation;<br>(2) Ischemic stroke transformed into asymptomatic hemorrhagic stroke: The following two conditions must be met at the same time:<br>a. Imaging evidence (CT or MRI) of extravascular hemorrhage in the infarct area;<br>b. Hemorrhagic transformation does not cause symptoms, or cause symptoms with an increase of less than 4 points in NIHSS score after the initial ischemic event.
**Myocardial Infraction**	Acute myocardial infarction diagnosed by the third universal definition (Thygesen, 2012)<br>If there is clinical evidence of myocardial necrosis consistent with acute myocardial ischemia (MI), acute MI should be diagnosed. It can be diagnosed if it meets any of the following criteria:<br>(1) A rise and/ or fall of cardiac biomarkers (preferably troponin [cTn]) values with at least one value above the 99th percentile URL, and any of the followings is required:<br>a. Clinical symptoms of myocardial ischemia;<br>b. New myocardial ischemic changes in the ECG, including new ST-segment changes or left bundle branch block (LBBB) [According to whether there is ST-segment elevation in the ECG, it is classified as acute ST-segment elevation myocardial infarction (STEMI) and non-ST segment elevation myocardial infarction (NSTEMI)];<br>c. Pathological Q wave detected in ECG;<br>d. Imaging demonstration of new loss of viable myocardium or new regional wall motion abnormality;<br>e. Coronary thrombosis confirmed by angiography or autopsy.<br>(2) Cardiac death with symptoms suggestive of myocardial ischaemia and presumed new ischaemic ECG changes or new LBBB, but death occurring before cardiac biomarkers could be obtained, before cardiac biomarker could rise, or in rare cases cardiac biomarkers were not collected.<br>(3) Myocardial infarction related to percutaneous coronary intervention (PCI) is arbitrarily defined by elevation of cTn values >5×99th percentile URL in patients with normal baseline values (≤99th percentile URL) or a rise of cTn values >20% if the baseline values are elevated and are stable or falling. In addition, any of the followings is required:<br>a. Symptoms suggestive of myocardial ischemia;<br>b. New ischemic ECG changes or new LBBB;

	c. Angiographic loss of patency of a major coronary artery or a side branch or persistent slow- or no-flow or embolization;<br>d. Imaging demonstration of new loss of viable myocardium or new regional wall motion abnormality.<br>(4) Myocardial infarction related to stent thrombosis is detected by coronary angiography or autopsy in the setting of myocardial ischemia and with a rise and/or fall of cardiac biomarkers values with at least one value above the 99th percentile URL.<br>(5) Myocardial infarction related to coronary artery bypass grafting (CABG) is arbitrarily defined by elevation of cardiac biomarker values＞10×99th percentile URL in patients with normal baseline cTn values (≤99th percentile URL). In addition, any of the followings is required:<br>a. new pathological Q waves or new LBBB;<br>b. angiographic documented new graft or new native coronary artery occlusion;<br>c. imaging evidence of new loss of viable myocardium or new regional wall motion abnormality
**Vascular Death**	Vascular death includes sudden cardiac death, death due to stroke, acute myocardial infarction, heart failure, pulmonary embolism, cardiac/cerebrovascular intervention or surgery (unrelated to acute MI) and other cardiovascular causes [e.g. arrhythmia irrelevant with sudden cardiac death, aortic aneurysm rupture, or peripheral artery disease].<br>Any death of unknown/unclear cause within 30 d after stroke, myocardial infarction, or cardio-cerebrovascular operation/surgery will be regarded as death due to stroke, myocardial infarction, or cardio-cerebrovascular operation/surgery, respectively.

Appendix 2. Evaluation Method of Modified Rankin Scale

The modified Rankin scale is used to measure functional recovery after stroke. Boldface shows the formal definition of each level. Italics provide further guidance to reduce the possible errors among observers, but the form of interview is not restricted. Please take in account only the symptoms that occur after stroke. If the patient does not require outside help, if he is able to walk with the aid of certain assistive devices, then he is considered to be able to walk independently.

If the two level seems equally applicable to the patient and further questions are not likely to make the absolute right choice, the more severe one should be selected.

0- No symptoms

Although there may be mild symptoms, the patient is not aware of any new functional limitations or symptoms after the stroke.

1- No significant disability. Able to carry out all usual activities, despite some symptoms.

Some patients have symptoms caused by stroke, whether physical or cognitive (such as influencing speech, reading and writing; or body movement or feeling; or vision; or swallowing; or emotion), but they are still able to continue to engage in all previous work, social and leisure activities. The key question used to distinguish between grades 1 and 2 (see below) can be, "is there anything you often did, but you can't do it after the stroke?". Activities with frequencies exceeding once a month are considered "often".

2- Slight disability; Able to look after own affairs without assistance, but unable to carry out all previous activities.

Patients are able to finish activities (such as driving, dancing, reading, or work) before stroke and can no longer able to finish after stroke, but they still can take care of themselves every day without the help of others. Patients can dress, walk, dine, go to the bathroom, prepare simple food, shop, travel locally, etc. Their life require no supervision. It is assumed that patients at this level can live alone for a week or more without care.

3- Moderate disability; Requires some help, but able to walk unassisted

At this level, patients can walk independently (assisted by walking machinery), dress independently, go to the bathroom, dine and so on, but more complex tasks need to be done with the help of others. For example, they need someone else to do the shopping , cooking cleaning, and visiting them more than once a week to ensure the completion of these activities. What needs assistance is not only to take care of the body, but also to give advice: for example, patients at this level will need supervision or encouragement to deal with finances.

4- Moderately severe disability; Unable to attend to own bodily needs without assistance, and unable to walk unassisted. Patients require others to help with their daily lives in walking, dressing, going to the bathroom or eating. Patients need to be taken care of at least once a day, usually twice or more times, or must live very close to caregivers. To differentiate between grades 4

and 5 (see below), consider whether they can routinely live alone for a proper amount of time during the day.<br>**5- Severe disability; Requires constant nursing care and attention, bedridden, incontinent.** Although no trained nurses are neccessary, they need to be taken care of throughout the day and night.

Appendix 3. NIHSS Score

Administer stroke scale items in the order listed. Record performance in each category after each subscale exam. Do not go back and change scores. Follow directions provided for each exam technique. Scores should reflect what the patient does, not what the clinician thinks the patient can do. The clinician should record answers while administering the exam and work quickly. Except where indicated, the patient should not be coached (ie, repeated requests to patient to make a special effort).

	Examination	Grade	Score
1a	Level of consciousness:<br>The investigator must choose a response if a full evaluation is prevented by such obstacles as an endotracheal tube, language barrier, orotracheal trauma/bandages. A 3 is scored only if the patient makes no movement (other than reflexive posturing) in response to noxious stimulation.	0 = **Alert;** keenly responsive.<br>1 = **Not alert;** but arousable by minor stimulation to obey, answer, or respond.<br>2 = **Not alert;** requires repeated stimulation to attend, or is obtunded and requires strong or painful stimulation to make movements (not stereotyped).<br>3 = Responds only with reflex motor or autonomic effects or totally unresponsive, flaccid, and areflexic.	
1b	**1b. LOC questions:**The patient is asked the month and his/her age. The answer must be correct - there is no partial credit for being close. Aphasic and stuporous patients who do not comprehend the questions will score 2. Patients unable to speak because of endotracheal intubation, orotracheal trauma, severe dysarthria from any cause, language barrier, or any other problem not secondary to aphasia are given a 1. It is important that only the initial answer be graded and that the examiner not "help" the patient with verbal or non-verbal cues.	0 = **Answers** both questions correctly.<br>1 = **Answers** one question correctly.<br>2 = **Answers** neither question correctly.	
1c	**LOC commands:**The patient is asked to open and close the eyes and then to grip and release the non-paretic hand. Substitute another one step command if the hands cannot be used. Credit is given if an unequivocal attempt is made but not completed due to weakness. If the patient does not respond to command, the task should be demonstrated to him or her (pantomime), and the result scored (ie, follows none, one or two commands). Patients with trauma, amputation, or other physical impediments should be given suitable one-step commands. Only the first attempt is scored.	0 = **Performs** both tasks correctly.<br>1 = **Performs** one task correctly.<br>2 = **Performs** neither task correctly.	
2	**Best gaze:** Only horizontal eye movements will be tested. Voluntary or reflexive (oculocephalic) eye movements will be scored, but caloric testing is not done. If the patient has a conjugate deviation of the eyes that can be overcome by voluntary or reflexive activity, the score will be 1. If a patient has an isolated peripheral nerve paresis (CN III, IV or VI), score a 1. Gaze is testable in all aphasic patients. Patients with ocular trauma, bandages, pre-existing blindness, or other disorder of visual acuity or fields should be tested with reflexive movements, and a choice made by the investigator. Establishing eye contact and then moving about the patient from side to side will occasionally clarify the presence of a partial gaze palsy.	0 = **Normal.**<br>1 = **Partial gaze palsy;**gaze is abnormal in one or both eyes, but forced deviation or total gaze paresis is not present.<br>2 = **Forced deviation,** or total gaze paresis not overcome by the oculocephalic maneuver.	
3	**Visual:** Visual fields (upper and lower quadrants) are tested by confrontation, using finger counting or visual	0 = No vision loss	

	threat, as appropriate. Patients may be encouraged, but if they look at the side of the moving fingers appropriately, this can be scored as normal. If there is unilateral blindness or enucleation, visual fields in the remaining eye are scored. Score 1 only if a clear-cut asymmetry, including quadrantanopia, is found. If patient is blind from any cause, score 3. Double simultaneous stimulation is performed at this point. If there is extinction, patient receives a 1, and the results are used to respond to item 11.	1 = Partial hemianopia<br>2 = Complete hemianopia<br>3 = Bilateral Blindness, (blind, including cortical blindness)	
4	**Facial palsy:** Ask - or use pantomime to encourage - the patient to show teeth or raise eyebrows and close eyes. Score symmetry of grimace in response to noxious stimuli in the poorly responsive or non-comprehending patient. If facial trauma/bandages, orotracheal tube, tape or other physical barriers obscure the face, these should be removed to the extent possible.	0 = **Normal** symmetrical movements.<br>1 = **Minor paralysis**(flattened nasolabial fold, asymmetry on smiling).<br>2 = **Partial paralysis** (total or near-total paralysis of lower face).<br>3 = **Complete paralysis** of one or both sides (absence of facial movement in the upper and lower face).	
5	**Motor arm:** The limb is placed in the appropriate position: extend the arms (palms down) 90 degrees (if sitting) or 45 degrees (if supine). Drift is scored if the arm falls before 10 seconds. The aphasic patient is encouraged using urgency in the voice and pantomime, but not noxious stimulation. Each limb is tested in turn, beginning with the non-paretic arm. Only in the case of amputation or joint fusion at the shoulder, the examiner should record the score as 9, and clearly write the explanation for this choice.	0 = **No drift;** limb holds 90 (or 45) degrees for full 10 seconds.<br>1 = **Drift;** limb holds 90 (or 45) degrees, but drifts down before full 10 seconds; does not hit bed or other support.<br>2 = **Some effort against gravity;** limb cannot get to or maintain (if cued) 90 (or 45) degrees, drifts down to bed, but has some effort against gravity.<br>3 = **No effort against gravity;** limb falls.<br>4 = **No movement.**<br>9 = **Amputation** or joint fusion, explain:________________	5a left arm<br>_____<br>5B right arm<br>_____
6	**Motor leg:** The limb is placed in the appropriate position: hold the leg at 30 degrees (always tested supine). Drift is scored if the leg falls before 5 seconds. The aphasic patient is encouraged using urgency in the voice and pantomime, but not noxious stimulation. Each limb is tested in turn, beginning with the non-paretic leg. Only in the case of amputation or joint fusion at the hip, the examiner should record the score as 9, and clearly write the explanation for this choice.	0 = **No drift;** leg holds 30-degree position for full 5 seconds.<br>1 = **Drift;** leg falls by the end of the 5-second period but does not hit bed.<br>2 = **Some effort against gravity;** leg falls to bed by 5 seconds, but has some effort against gravity.<br>3 = **No effort against gravity;** leg falls to bed immediately.<br>4 = **No movement.**<br>9 = **Amputation** or joint fusion, explain:________________	6a left leg<br>_____<br>6a right leg<br>_____
7	**Limb ataxia:** This item is aimed at finding evidence of a unilateral cerebellar lesion. Test with eyes open. In case of visual defect, ensure testing is done in intact visual field. The finger-nose-finger and heel-shin tests are performed on both sides, and ataxia is scored only if	0 = **Absent.**<br>1 = **Present in one limb.**<br>2 = **Present in two limbs.**	

	present out of proportion to weakness. Ataxia is absent in the patient who cannot understand or is paralyzed. Only in the case of amputation or joint fusion, the examiner should record the score as 9, and clearly write the explanation for this choice. In case of blindness, test by having the patient touch nose from extended arm position.	9 = **Amputation** or joint fusion, explain:________________	
8	**Sensory:** Sensation or grimace to pinprick when tested, or withdrawal from noxious stimulus in the obtunded or aphasic patient. Only sensory loss attributed to stroke is scored as abnormal and the examiner should test as many body areas (arms [not hands], legs, trunk, face) as needed to accurately check for hemisensory loss. A score of 2, "severe or total sensory loss," should only be given when a severe or total loss of sensation can be clearly demonstrated. Stuporous and aphasic patients will, therefore, probably score 1 or 0. The patient with brainstem stroke who has bilateral loss of sensation is scored 2. If the patient does not respond and is quadriplegic, score 2. Patients in a coma (item 1a=3) are automatically given a 2 on this item.	0 = Normal, without sensory loss<br>0 = **Normal;** no sensory loss.<br>1 = **Mild-to-moderate sensory loss;** patient feels pinprick is less sharp or is dull on the affected side; or there is a loss of superficial pain with pinprick, but patient is aware of being touched.<br>2 = **Severe to total sensory loss;** patient is not aware of being touched in the face, arm, and leg.	
9	**Best language:** A great deal of information about comprehension will be obtained during the preceding sections of the examination. For this scale item, the patient is asked to describe what is happening in the attached picture, to name the items on the attached naming sheet and to read from the attached list of sentences. Comprehension is judged from responses here, as well as to all of the commands in the preceding general neurological exam. If visual loss interferes with the tests, ask the patient to identify objects placed in the hand, repeat, and produce speech. The intubated patient should be asked to write. The patient in a coma (item 1a=3) will automatically score 3 on this item. The examiner must choose a score for the patient with stupor or limited cooperation, but a score of 3 should be used only if the patient is mute and follows no one-step commands.	0 = **No aphasia;** normal.<br>1 = **Mild-to-moderate aphasia;** some obvious loss of fluency or facility of comprehension, without significant limitation on ideas expressed or form of expression. Reduction of speech and/or comprehension, however, makes conversation about provided materials difficult or impossible. For example, in conversation about provided materials, examiner can identify picture or naming card content from patient's response.<br>2 = **Severe aphasia;** all communication is through fragmentary expression; great need for inference, questioning, and guessing by the listener. Range of information that can be exchanged is limited; listener carries burden of communication. Examiner cannot identify materials provided from patient response.<br>3 = **Mute, global aphasia;** no usable speech or auditory comprehension.	
10	**Dysarthria:** If patient is thought to be normal, an adequate sample of speech must be obtained by asking patient to read or repeat words from the attached list. If the patient has severe aphasia, the clarity of articulation of spontaneous speech can be rated. Only if the patient is intubated or has other physical barriers to producing speech, the examiner should record the score as 9, and clearly write an explanation for this choice. Do not tell the patient why he or she is being tested.	0 = **Normal.**<br>1 = **Mild-to-moderate dysarthria;** patient slurs at least some words and, at worst, can be understood with some difficulty.<br>2 = **Severe dysarthria;** patient's speech is so slurred as to be unintelligible in the absence of or out of proportion to any dysphasia, or is mute/anarthric.<br>9 = **Intubated** or other physical barrier, explain:________________	

11	**Extinction and inattention (formerly neglect):**Sufficient information to identify neglect may be obtained during the prior testing. If the patient has a severe visual loss preventing visual double simultaneous stimulation, and the cutaneous stimuli are normal, the score is normal. If the patient has aphasia but does appear to attend to both sides, the score is normal. The presence of visual spatial neglect or anosognosia may also be taken as evidence of abnormality. Since the abnormality is scored only if present, the item is never untestable.	0 = **No abnormality.**<br>1 = **Visual, tactile, auditory, spatial, or personal inattention** or extinction to bilateral simultaneous stimulation in one of the sensory modalities.<br>2 = **Profound hemi-inattention or extinction to more than one modality;** does not recognize own hand or orients to only one side of space.	
Total			

Attachment:

1. 9th, 10th inspection's chart

Reading inspection chart 1

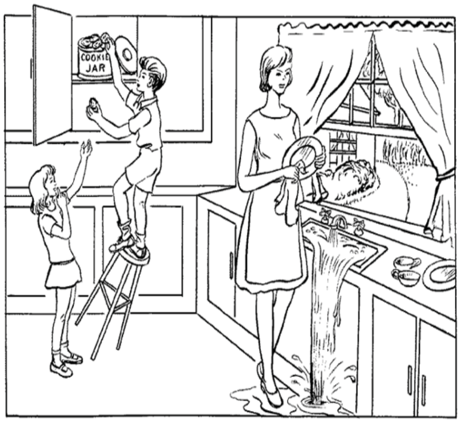

Reading inspection chart 2

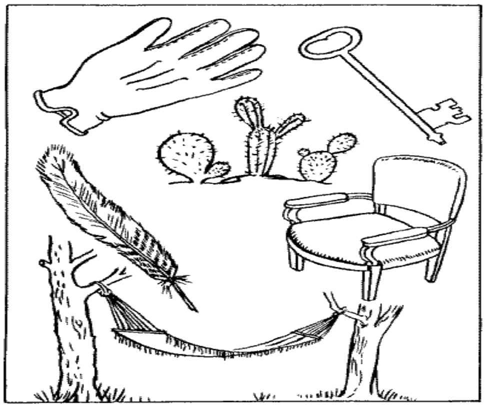

Reading inspection chart 3

Please read the following sentences:

know

Walk down the stairs

Home cooking

Review at school

Deliver a wonderful speech

Reading inspection chart 4

Please read the following words

Mom

earth

Aircraft

silk

Start on time

Eat grapes without spitting grapes

2 How NIHSS scores are rated for special conditions?

➢ How is the NIHSS score assessed in uncooperative patients?

If a patient is uncooperative on an item, document it clearly. All untested items are to be reviewed by the medical inspector and discussed with the examiner if necessary.

➢ How is the NIHSS score assessed in coma patients?

If patients scores a 3 in this factor, the default coma scores should be used when applicable.

1a = 3 if the patient makes only reflexive posturing movements to repeated painful stimuli, or, they are totally unresponsive. If 1a = 3, other items should be evaluated as:

- 1b- level of consciousness questions: score 2
- 1c- level of consciousness commands: score 2
- 2- best gaze: see if the patient's best gaze can be balanced by oculoucephalogyric reflex. If balanced, score 1; if not, score 2.
- 3- visual: use visual threat to evaluate.
- 4- facial palsy: score 3
- 5 and 6- motor arm and motor leg: score 4 for each limb
- 7- limb ataxia: score only when there exists ataxia. If the patient fails due to muscle weakness, score 0.
- 8- sensory: score 2
- 9- best language: score 3
- 10- dysarthria: score 2
- 11- extinction and inattention: score 2 as coma means losing all cognitive abilities

➢ How is the NIHSS score assessed in coma patients?

- 1b - Consciousness level questioning: 2 points for aphasics who cannot understand the question. Scoring can only be based on the initial response. This item can never be scored as "unable to check".
- 1c - Level of Consciousness Instructions: If the patient does not respond to instructions, the examiner demonstrates (gestures) and then scores based on the outcome (e.g., compliance with 0, 1, or 2 instructions). The patient is then scored based on the outcome (e.g., compliance with 0, 1, or 2 commands). Only the first attempt can be scored and the question can be asked only once.
- 2 - Optimal Gaze: For all aphasic individuals, gaze is checked. In aphasic patients, it is helpful to establish eye contact and walk around the bed. This item is an exception to the principle of observing the first response and the inability to train. If the patient is unable to actively gaze, head-eye movements, eye gaze and tracking the examiner's methods can be used to provide a stronger examination stimulus.
- 3-Field of vision: not limited by aphasia.
- 4-Facial palsy: Patients with aphasia are scored based on symmetry of expression during injurious

stimulation.

- 5 - Upper limb movements: for aphasic patients guided by voice or gesture without injurious stimulation. If you measure the non-hemiplegic side of the limb first, the aphasic patient may understand what you are trying to measure.
- 6 - Lower extremity movements: Use voice or gesture guidance for the aphasic person, without injurious stimuli. If you measure the non-hemiplegic limb first, the aphasic person may understand what you are trying to measure.
- 7 - Limb ataxia: If the patient cannot understand, score 0. If the limb is initially moved passively by the examinee, the aphasic patient often completes the examination correctly. The aphasic patient often completes the examination correctly if the limb is initially moved by the examiner.
- 8 - Sensory: examines the aphasic person's avoidance of injurious stimuli. A score of 2 can only be given if a severe or complete sensory deficit is clearly established. given. Therefore, the aphasic person may also be scored 1 or 0.
- 9 - Best Language: Ask the patient to look at pictures and talk, name objects on cards, and read sentences from the utterance list. Slight aphasia is scored as 1 point. Use all the materials provided Use all materials provided to decide whether to choose 1 or 2 points. It is estimated that the patient missed more than 2/3 of the named objects and sentences or performed very few and simple one-step instructions. If the patient missed more than 2/3 of the named objects and sentences or performed very few and simple one-step instructions, a score of 2 was assigned.
- 10 - Dysarthria: Patients with aphasia are scored based on their spontaneous speech and by having them repeat the words you read aloud. If there is severe aphasia, the Scores are based on the clarity of articulation in spontaneous speech.
- 11 - Sensory fade and loss of attention (formerly neglect): If aphasic, but do notice bilaterally, score normal. Because abnormalities are recorded only when the performance is abnormal, this item must be scored as normal. abnormality is recorded only when there is abnormal performance, so this item must be measurable

3 How to calculate the total score of NIHSS?

When calculating total score, the following items should not be included in the total score:

- Item 5, 6 in limb movements “9= amputation or fusion of joints"
- Item 7 of ataxia “9=Amputation or joint fusion, explain:”
- Item 10 of dysarthria “ Intubated or other physical barrier, explain:__”

Appendix 4. ABCD^2 Score

Items		Options	Score
A	Age	≥ 60 years	1
		< 60years	0
B	First Blood Pressure after TIA	SBP≥ 140mmHg or DBP≥ 90mmHg	1
		SBP< 140mmHg and DBP< 90mmHg	0
C	Clinical Symptom	Unilateral motor weakness	2
		Speech disturbance (either dysarthria or dysphasia or both) without weakness	1
		Other symptoms	0
D	Duration of TIA symptoms	≥ 60 minutes	2
		10-59 minutes	1
		< 10minutes	0
D	Diabetes	with	1
		without	0

Appendix5. EQ-5D Scale

Which of the followings best describes your state of health today?

Mobility

○ I have no problems in walking about

○ I have some problems in walking about

○ I am confined to bed

Self-care

○ I have no problems with self-care

○ I have some problems washing or dressing myself

○ I am unable to wash or dress myself

Usual activities (e.g. work, study, housework, family or leisure activities)

○ I have no problems with performing my usual activities

○ I have some problems with performing my usual activities

○ I am unable to perform my usual activities

Pain / discomfort

○ I have no pain or discomfort

○ I have moderate pain or discomfort

○ I have extreme painful or uncomfortable

Anxiety / depression

○ I am not anxious or depressed

○ I am moderately anxious or depressed

○ I am extremely anxious or depressed

We would like to know how good or bad your health is TODAY.

This scale is numbered from 0 to 100.

100 means the best health you can imagine. 0 means the worst health you can imagine.

Mark an X on the scale to indicate how your health is TODAY.

Now, please write the number you marked on the scale in the box below.

How good or bad your own health is today?

How is your health today?

The questionnaire was completed by:

○1 patients themselves

○2 patients with the help of the third

○3 agents (family members of patients)

The best health you can imagine

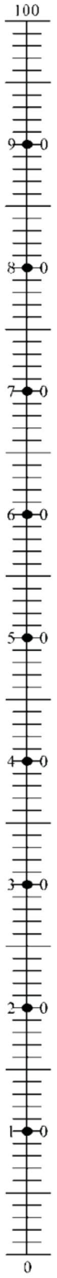

The worst health you can imagine

Appendix 6. GUSTO Bleeding Criteria

If the patient's bleeding events are consistent with multiple classifications, it should be classified as the most severe one.

1. Severe bleeding

Fatal or intracranial hemorrhage or bleeding resulting in haemodynamic changes requiring blood or fluid transfusions, cardiac stress therapy, ventricular assist devices, surgery, or cardiopulmonary resuscitation.

2. Moderate bleeding

Bleeding requiring transfusion therapy but not resulting in hemodynamic changes that require intervention.

3. Minor bleeding

Bleeding that does not require transfusion or fluid therapy and does not result in hemodynamic changes. This includes subcutaneous bleeding, small hematomas or bruises at the puncture site.

During the study, all transfusion of blood products will be recorded on the case report form, including infusion units and capacity.

1, Hb reference range 130 to 180 g/L (male); 120 to 160 g/L (female)

2, Consider the impact of blood transfusion, Hb measurements must adjust the infusion of PRBC or whole blood between two hematological tests. Suppose 1 units of blood transfusion cause Hb to increase by 10 g/L. Thus, when the blood transfusion is performed between the two blood tests, the actual values of Hb can be calculated by using the following formula: **Hb = [baseline Hb -Hb after blood transfusion]+ [infusion unit x Hb conversion coefficient *]**

*Hb conversion factor = 10 g/L

Appendix 7. Clinical Crucial Laboratory Abnormalities

Bleeding tendency	Prothrombin time more than 1.5 times or platelet count < 10 x 10^9 / L
Medium and severe anemia	Hemoglobin (Hb) < 90 g/L
Abnormal liver function	More than 2 times higher than normal transaminase
Abnormal renal function	Serum creatinine > 1.5 mg/dl or creatinine clearance < 50 ml/min

Intensive Statin and Antiplatelet Therapy for High-risk Intracranial or Extracranial Atherosclerosis (INSPIRES)

Study Protocol

Principal Investigators： Yilong Wang, Yongjun Wang

Beijing Tiantan Hospital, Capital Medical University, Beijing, China

Protocol Version: 4.0

Jun. 6th, 2020

Table of Contents

Summary of The Trial Protocol 82
INSPIRES Abbreviated Vocabulary 89
1. Study Background 92
2. Study Purposes 99
3. Study Design 103
4. Study Outcomes 105
5. Subjects Selection 106
6. Therapeutic Regimen 109
7. Study Implementation 110
8. Blood Sample Collection 116
9. Data Collection 117
10. Study Procedure 118
11. Ethics and Regulations 125
12. Research Organization 127
13. Quality Control and Quality Assurance 129
14. Data Preservation 130
15. Safety Monitoring of Data 131
16. Statistical Processing 132
17. Confidentiality and Announcement of Results 137
Reference: 138
Appendix 1. The Definition of Cardiac-cerebral Vascular Events 144
Appendix 2. Evaluation Method of Modified Rankin Scale 146
Appendix 3. NIHSS Score 148
Appendix 4. $ABCD^2$ Score 155
Appendix5. EQ-5D Scale 156
Appendix 6. GUSTO Bleeding Criteria 158
Appendix 7. Clinical Crucial Laboratory Abnormalities 159

Summary of The Trial Protocol

**Study title**	**Intensive Statin and Antiplatelet Therapy for High-risk Intracranial or Extracranial Atherosclerosis**
**Study purposes**	**Major purposes:**<br><br>**1.** To evaluate the efficacy and safety of intensive antiplatelet therapy versus standard antiplatelet therapy in reducing the risk of new stroke at 90 days in acute mild ischemic stroke or high-risk TIA patients attributed to extracranial or intracranial atherosclerosis.<br>**2.** To evaluate the efficacy and safety of immediate intensive statin therapy (atorvastatin 80mg/d) versus delayed intensive statin therapy (atorvastatin 40mg/d) in reducing the risk of new stroke at 90 days in acute mild ischemic stroke or high-risk TIA patients attributed to extracranial or intracranial atherosclerosis.<br>**3.** To evaluate the efficacy and safety of intensive antiplatelet combined with immediate intensive statin therapy (atorvastatin 80mg/d) versus standard antiplatelet combined with delayed intensive statin therapy (atorvastatin 40mg/d) in reducing the risk of new stroke at 90 days in acute mild ischemic stroke or high-risk TIA patients attributed to extracranial or intracranial atherosclerosis.<br>**Notes:**<br>• Intensive antiplatelet therapy refers to dual antiplatelet therapy with aspirin and clopidogrel;<br>• Standard antiplatelet therapy refers to antiplatelet monotherapy with aspirin;<br>• Immediate intensive statin therapy (atorvastatin 80mg/d) refers to lipid-lowering therapy since randomization with atorvastatin at a dose of 80 mg daily for the first 21 days, followed by 40 mg daily for days 22-90;<br>• Delayed intensive statin therapy (atorvastatin 40mg/d) refers to lipid-lowering therapy with atorvastatin at a dose of 40 mg daily from the 4th day after randomization to the 90th day;<br>• Acute mild ischemic stroke or high-risk TIA patients attributed to extracranial or intracranial atherosclerosis: at least one of the following characteristics:<br>a) Mild ischemic stroke (NIHSS score 4–5) within 24 hours after onset and either of the following imaging characteristics:<br>1) Acute single infarction with ≥50% stenosis of a major intracranial or extracranial artery that likely accounts for the infarction and clinical presentation.<br>2) Acute multiple infarctions documented by head CT or MRI, attributed to large-artery atherosclerosis, including non-stenotic vulnerable plaques.<br>b) Mild ischemic stroke (NIHSS score ≤5) or high-risk transient ischemic attack (TIA, $ABCD^2$ score ≥4) within 24-72 hours after onset and meet any of the following imaging characteristics:<br>1) TIA with ≥50% stenosis of a major intracranial or extracranial artery that likely accounts for the clinical presentation.<br>2) Acute single infarction with ≥50% stenosis of a major intracranial or extracranial

	artery that likely accounts for the infarction and clinical presentation.<br>3) Acute multiple infarctions documented by head CT or MRI, attributed to large-artery atherosclerosis, including non-stenotic vulnerable plaques.
**Study design**	Randomized, double-blind, placebo-controlled, multicenter, 2×2 factorial designed clinical trial.
**Total number of cases**	6100 cases
**Subjects selection**	Inclusion Criteria<br>2. Age :35-80 years.<br>3. At least one of the following (a-b):<br>a) Mild ischemic stroke (NIHSS score 4-5) within 24 hours after onset and either of the following imaging characteristics:<br>1) Acute single infarction with ≥50% stenosis of a major intracranial or extracranial artery that likely accounts for the infarction and clinical presentation.<br>2) Acute multiple infarctions documented by head CT or MRI, attributed to large-artery atherosclerosis, including non-stenotic vulnerable plaques.<br>b) Mild ischemic stroke (NIHSS score ≤5) or high-risk of TIA ($ABCD^2$ score≥4) within 24-72 hours after onset and meet any of the following imaging characteristics:<br>1) TIA with ≥50% stenosis of a major intracranial or extracranial artery that likely accounts for the clinical presentation;<br>2) Acute single infarction with ≥50% stenosis of a major intracranial or extracranial artery that likely accounts for the infarction and clinical presentation;<br>3) Acute multiple infarctions documented by head CT or MRI, attributed to large-artery atherosclerosis, including non-stenotic vulnerable plaques.<br>3. Written informed consent.<br>**Notes:**<br>• Intracranial large arteries include the intracranial portion of internal carotid arteries, middle cerebral arteries (M1/M2), anterior cerebral arteries (A1/A2), posterior cerebral arteries (P1/P2), intracranial portion of vertebral arteries, and the basilar artery. [1,2];The degree of intracranial artery stenosis is defined on magnetic resonance angiography (MRA), computerized tomography angiography (CTA) or digital substraction angiography (DSA) by criteria from the Warfarin–Aspirin Symptomatic Intracranial Disease (WASID) study [2];<br>• Extracranial large arteries include the extracranial portion of carotid arteries and vertebral arteries (including

	the portion from initiation of carotid arteries and vertebral arteries to the start of intracranial section, except the subclavian artery, aortic arch and brachiocephalic trunk). The degree of extracranial arteries stenosis is defined on carotid duplex ultrasound, CTA, CE-MRA or DSA by criteria from the North American Symptomatic Carotid Endarterectomy Trial (NASCET) [3];<br>• Acute ischemic stroke with multiple infarction lesions refers to more than one lesion appearing in different locations (separated in space or non-continuous on contiguous slices) on diffusion DWI/ADC imaging, however, it will be considered as the same one if the lesion is continuous on the adjacent levels[4];<br>• TIA: Sudden neurologic deficit lasting less than 24 hours, without non-ischemic causes.<br>• TIA patients with acute multiple cerebral infarctions will also be enrolled.
	**Exclusion criteria**<br>1. Presumed cardioembolic stroke or TIA (e.g. atrial fibrillation, heart valve prosthesis, atrial myxoma, endocarditis, etc.).<br>2. Other determined etiology of stroke or TIA (e.g. aortic dissection, cervico-cerebral artery dissection, vasculitis, vascular malformation, moyamoya disease/syndrome, fibromuscular dysplasia, etc.).<br>3. Non-vascular neurological diseases (e.g. intracranial tumor, multiple sclerosis, etc.).<br>4. Index infarction affects ≥ 50% of a cerebral lobe (e.g. parietal, frontal, occipital);<br>5. Hemorrhagic transformation after onset.<br>6. Contraindications to clopidogrel, aspirin or atorvastatin:<br>h) History of hypersensitivity;<br>i) Severe heart failure (NYHA classification: III- IV) or asthma;<br>j) Coagulation disorder or systemic bleeding;<br>k) History of drug-induced hematologic or hepatic abnormalities;<br>l) Leukopenia (< 2×10^9/L) or thrombocytopenia (<100×10^9/L);<br>m) Active liver disease;<br>n) Pregnancy or lactation period.<br>***Severe heart failure：** New York Heart Association (NYHA) Classification III and IV<br>I :Cardiac disease, but no symptoms and no limitation in ordinary physical activity, e.g. no shortness of breath when walking, climbing stairs etc.<br>II (mild heart failure): Mild symptoms (mild shortness of breath and/or angina) and slight limitation during ordinary activity.<br>III (moderate heart failure): Marked limitation in activity due to symptoms, even during less-than-ordinary activity, e.g. walking short distances (20–100 m). Comfortable only at rest.<br>IV (Severe heart failure): Severe limitations. Experiences symptoms even while at rest. Mostly bedbound patients.<br><br>7. Pre-existing disability with modified Rankin Scale (mRS) score >2.<br>8. Intra-arterial or intravenous thrombolysis or endovascular therapy after onset.

	9. Defibrinogen therapy (e.g. defibrase and lumbrokinase), anticoagulation therapy (e.g. argatroban), or antiplatelet therapy (e.g. ticagrelor, tirofiban) except for clopidogrel and aspirin after onset.<br>10. Creatine kinase > 5 times the upper limit of normal value of onset.<br>11. Drug use related to statin metabolism within 14 days before randomization (e.g. immune-suppressive drugs, antifungal agents, fibrates).<br>12. Severe hepatic insufficiency (alanine transaminase or aspartate transaminase > 2 times the upper limit of normal value) or renal insufficiency (creatinine > 1.5 times the upper limit of normal value or glomerular filtration rate < 40 ml/min/1.73 m^2).<br>13. Dual antiplatelet therapy with aspirin and clopidogrel within 14 days before randomization (Patients who started aspirin plus clopidogrel without loading dose(300mg) of clopidogrel after onset are not excluded from the trial.).<br>14. High-intensity statin therapy within 14 days before randomization (e.g. atorvastatin ≥ 40mg/d, rosuvastatin ≥ 20mg/d).<br>15. History of intracranial hemorrhage (e.g. intracerebral or subarachnoid hemorrhage).<br>16. Gastrointestinal bleeding or major surgery within 90 days.<br>17. History of intracranial or extracranial angioplasty.<br>18. Planned long-term use of antiplatelet drugs or non-steroidal anti-inflammatory drugs except for study drugs.<br>19. Planned surgery or revascularization that may need to stop taking the study drugs within the next 90 days.<br>20. Anticipated life expectancy < 90 days.<br>21. Currently participating in any other investigational drug or device study.<br>22. Unable to complete the follow-up (e.g. dementia, alcoholism, substance abuse, severe mental disease).
**Therapeutic regimen**	Patients who meet the requirements are randomly assigned to one of the following four groups according to the ratio of 1:1:1:1:<br>A: Intensive antiplatelet therapy + immediate intensive statin therapy (atorvastatin 80mg/d)<br>B: Intensive antiplatelet therapy + delayed intensive statin therapy (atorvastatin 40mg/d)<br>C: Standard antiplatelet therapy + immediate intensive statin therapy (atorvastatin 80mg/d)<br>D: Standard antiplatelet therapy + delayed intensive statin therapy (atorvastatin 40mg/d)<br>**Intensive antiplatelet therapy：**<br>Day 1： Clopidogrel 300mg/day+asprin100-300mg/ day<br>Day2 - Day21±2: Clopidogrel 75mg/day+ aspirin 100mg/day

	Day22±2 -Day 90: Clopidogrel 75mg/day+ aspirin placebo<br>**Standard antiplatelet therapy：**<br>Day 1： Aspirin 100-300mg/day + clopidogrel placebo<br>Day 2 - 90： Aspirin 100mg/day+ clopidogrel placebo<br>**Immediate intensive statin therapy (atorvastatin 80mg/d)：**<br>Day 1 -Day 21±2： Atorvastatin 80mg/day<br>Day22±2 -Day 90： Atorvastatin 40mg/day<br>**Delayed intensive statin therapy (atorvastatin 40mg/d)：**<br>Day 1 - Day3： Atorvastatin placebo<br>Day4 - Day21±2： Atorvastatin 40mg/day + Atorvastatin calcium placebo<br>Day22±2 -Day 90： Atorvastatin 40mg/day
**Study Outcomes**	**Primary outcome:**<br>Stroke (ischemic or hemorrhagic)<br>**Secondary outcomes:**<br>• Composite vascular events：stroke (ischemic or hemorrhagic), myocardial infarction, or cardiovascular death.<br>• Ischemic stroke<br>• TIA<br>• Severity of stroke or TIA on an ordinal scale (a six-level ordered category scale combined vascular events with mRS score: fatal stroke (stroke with subsequent death), severe stroke (stroke followed by mRS of 4-5), moderate stroke (stroke followed by mRS of 2-3), mild stroke (stroke followed by mRS of 0-1), TIA, and no stroke/TIA)<br>• Myocardial infarction<br>• Vascular death<br>• All-cause death<br>• Poor functional outcome (mRS score 2-6)<br>• Poor quality of life (EuroQoL-5 Dimensions index score≤0.5)<br>• Early neurological deficits (increase in NIHSS score at 7days)
	**Primary safety outcome:**<br>Moderate to severe bleeding (Appendix 6)<br>**Secondary safety outcomes:**<br>• Intracranial hemorrhage<br>• Hepatotoxicity： alanine transaminase or aspartate transaminase > 3 times the upper limit of normal value after statin treatment.<br>• Muscle toxicity: creatine kinase > 10 times the upper limit of normal value, or the presence of muscle pain, myopathy or rhabdomyolysis.<br>• Death<br>• Other AEs/SAEs

**Statistical analysis**	Statistical analysis will be conducted with SAS 9.4. The two-sided P<0.05 is considered statistically significant.<br>**1.Major null hypothesis:**<br>1) In patients with acute mild ischemic stroke or high-risk TIA patients attributed to extracranial or intracranial atherosclerosis treated within 72 hours of ictus, there is no difference in the risk of a new stroke within 90 days between subjects with intensive antiplatelet therapy and those with standard antiplatelet therapy.<br>2) In patients with acute mild ischemic stroke or high-risk TIA patients attributed to extracranial or intracranial atherosclerosis treated within 72 hours of ictus, there is no difference in the risk of a new stroke within 90 days between subjects with immediate intensive statin therapy (atorvastatin 80mg/d) and delayed intensive statin therapy (atorvastatin 40mg/d).<br>3) In patients with acute mild ischemic stroke or high-risk TIA patients attributed to extracranial or intracranial atherosclerosis treated within 72 hours of ictus, there is no difference in the risk of a new stroke within 90 days between subjects with intensive antiplatelet combined with immediate intensive statin therapy (atorvastatin 80mg/d) and standard antiplatelet combined with delayed intensive statin therapy (atorvastatin 40mg/d).<br><br>**2. Data sets of statistical analysis : intention-to-treat (ITT) population**<br><br>**3. Statistical analysis methods:**<br>3.1 The balance analysis of basic values (comparison between groups of baseline characteristics)<br>1) The measurement data will be tested by t test or Wilcoxon rank sum test;<br>2) The enumeration data between groups are compared with chi-square test, Fisher's exact probability method or Wilcoxon rank sum test.<br>3.2 Efficacy analysis<br>1) Primary outcome:<br>• Kaplan-Meier analyses will be used to evaluate survival curves for the primary endpoint, and survival curves will be compared by another factor stratification of the analytic design and using the Cox regression model Wald test. First, marginal effects will be compared between intensive and standard antiplatelet therapy, and also between immediate intensive statin therapy (atorvastatin 80mg/d) and delayed intensive statin therapy (atorvastatin 40mg/d). These are independent comparisons, they have no effect on the type I error rate. For comparisons of two therapies, the primary outcome of stroke will be considered statistically significant if the p-value is < 0.05. Second, if either intensive antiplatelet or immediate intensive statin therapy (atorvastatin 80mg/d) is statistically significant, the effects between combined intensive antiplatelet and immediate intensive

	statin treatment (atorvastatin 80mg/d) and combined standard antiplatelet and delayed intensive statin treatment (atorvastatin 40mg/d) will be tested.<br>2) Secondary outcomes:<br>a. Kaplan-Meier curve will be used to simulate the cumulative risk of 90-day follow-up combined with secondary endpoint events such as vascular events, ischemic stroke, TIA, myocardial infarction, vascular death and all-cause death. Cox proportional risk model will be used to calculate HR and the 95% confidence interval and the Log-rank test would be used to evaluate the efficacy;<br>b. For ordinal stroke or TIA, ordinal logistic regression will be used to calculate OR and 95% confidence interval. For the poor functional outcome (mRS score 2-6) and early neurological deficits (increase in NIHSS score at 7days) indexes, logistic regression will be used to calculate OR and 95% confidence interval.<br>c. Extreme values will be checked for validity and sensitivity analyses will be performed.<br>A two-sided test will be used for all statistics, and $P<0.05$ is considered statistically significant.<br>3.3 Safety analysis<br>Safety evaluation will be analyzed using safety data set. Moderate to severe bleeding, intracranial hemorrhage, and overall mortality will be calculated using the Kaplan-Meier curve to simulate the 3-month cumulative risk, and the Cox proportional hazards model to calculate the HR and 95% confidence interval. For other adverse events and laboratory abnormalities, the cases which was normal before the treatment and abnormal after the treatment would be mainly analyzed and listed, in addition to the comparison of differences before and after treatment.
**Follow-up plan**	Face to face interviews will be performed at baseline, Day7±2, Day14 (or hospital discharge) and Day90 ± 7, and telephone interviews will be performed at the 12th month±14 days after randomization.
**Study duration**	60 months

Reference

1. Wong KS, Chen C, Ng PW, et al. Low-molecular-weight heparin compared with aspirin for the treatment of acute ischaemic stroke in Asian patients with large artery occlusive disease: a randomized study. Lancet Neurol. 2007 May;6(5):407-13.

2. Chimowitz MI, Lynn MJ, Howlett-Smith H, et al. Comparison of warfarin and aspirin for symptomatic intracranial arterial stenosis. N Engl J Med, 2005,352(13):1305-1316.

3. North American Symptomatic Carotid Endarterectomy Trial Collaborators. Beneficial effect of carotid endarterectomy in symptomatic patients with high-grade carotid stenosis. N Engl J Med. 1991 Aug 15;325(7):445-53.

4. Amarenco P, Lavallée PC, Labreuche J, et al. One-Year Risk of Stroke after Transient Ischemic Attack or Minor Stroke. N Engl J Med. 2016 Apr 21;374(16):1533-42.

INSPIRES Abbreviated Vocabulary

Abbreviation	Interpretation
$ABCD^2$ score	To predict risk of stroke within 2 days after the onset of transient ischemic attack (TIA), and to predict the recurrence risk of mild stroke in the short term
ACEI	Angiotensin-converting enzyme inhibitor
AE	Adverse event
ALT	Alanine aminotransferase
ARB	Angiotensin-receptor blocker
ASA	Aspirin, Acetylsalicylic acid
ASSORT	Administration of Statin on Acute Ischemic Stroke Patient
AST	Aspartate aminotransferase
BP	Blood pressure
CABG	Coronary artery bypass graft
CEMRA	Contrast-enhanced magnetic resonance angiography
CHANCE	Clopidogrel in high-risk patients with acute non-disabling cerebrovascular events
CI	Confidence interval
CK	Creatine kinase
CLAIR	Clopidogrel plus aspirin versus aspirin alone for reducing embolisation in patients with acute symptomatic cerebral or carotid artery stenosis
COMPRESS	Combination of Clopidogrel and Aspirin for Prevention of Recurrence in Acute Atherothrombotic Stroke Study
CRF	Case report form
CRO	Contract Research Organization
CT	Computed Tomography
CTA	Computed tomography angiography

CYP2C19	Cytochrome P450, family2, subfamily C, polypeptide 19
CYP3A	Cytochrome P450, family 3, subfamily A
DSA	Digital subtraction angiography
DSMB	Data and Safety Monitoring Board
DWI	Diffusion-weighted imaging
EQ-5D	EuroQoL-5 Dimensions
GUSTO	Global Utilization of Streptokinase and Tissue Plasminogen Activator for Occluded Coronary Arteries
HR	Hazard ratio
ICH	Intracerebral hemorrhage
IRB	Institutional Review Board
LDL-C	Low-density lipoprotein cholesterol
MRA	Magnetic resonance angiography
MRI	Magnetic resonance imaging
mRS	Modified Rankin Scale
NIHSS	National Institute of Health Stroke Scale
NASCET	North American Symptomatic Carotid Endarterectomy Trial
OR	Odds ratio
PPI	Proton-pump inhibitor
rt-PA	Recombinant tissue-type plasminogen activitor
SAE	Serious adverse event
SAH	Subarachnoid hemorrhage
SAMMPRIS	Stenting and Aggressive Medical Management for Preventing Recurrent Stroke in Intracranial Stenosis
SPARCL	Stroke Prevention by Aggressive Reduction in Cholesterol Levels

TCD	Transcranial doppler
WASID	Warfarin - Aspirin for Symptomatic Intracranial Disease

1. Study Background

1.1 Burden and recurrence risk of acute mild ischemic stroke

Ischemic stroke is associated with higher rates of morbidity, recurrence, disability, and mortality, especially in Asian populations. Among them, non-disabling ischemic cerebrovascular events are the most common (accounting for more than 50% of all cerebrovascular diseases) and the recurrence rate is as high as 10-20%. Previous studies have found that early onset, severe artery stenosis and multiple infarctions are high risk factors for stroke recurrence in these patients.

(1) The early stage of onset: In the cohort studies, stroke recurrence in patients with ischemic stroke was mostly within 30 days after onset.[1-3] In patients with TIA or minor stroke of large-artery atherosclerosis origin, the risk of recurrence is high within 7 days after onset.[3,4]

(2) Severe artery stenosis: Early cohort studies showed that the risk of stroke recurrence caused by large-artery atherosclerotic stenosis was greater than that caused by other etiological types. [1,2,5,6] The TIA.org study published in 2016 showed that the recurrent stroke risk in 90 days and 1 year of TIA and minor stroke caused by large-artery stenosis is the highest.[3] The results from the imaging subgroup of the Clopidogrel in High-Risk Patients with Acute Nondisabling Cerebrovascular Events (CHANCE) suggested that the risk of stroke recurrence in patients with TIA and minor stroke with intracranial arterial stenosis in 90 days was significantly higher than those without intracranial stenosis.[4] The results of the WASID study indicated that the risk of stroke recurrence in patients with symptomatic intracranial artery stenosis (stenosis rate $\geq$ 70%) is higher than those with a stenosis rate of 50-70%.[7] Besides, the Chinese Intracranial Atherosclerosis Study (CICAS) reported that the proportion of patients with extracranial or intracranial arterial stenosis occlusion was as high as 46.6%. Of these cases, 37.5% had only intracranial arterial stenosis, 4.9% had only extracranial arterial stenosis, and 9.1% had intracranial and extracranial arterial stenosis. Patients with large-artery atherosclerotic stenosis had higher risk of recurrence and poorer prognosis. During 12-month follow-up, it was found that the risk of recurrent stroke increased with the severity of stenosis. [8]

(3) Multiple infarctions: A large number of studies showed that the risk of stroke recurrence is high in ischemic stroke combined with acute multiple infarctions. [9,10] Results of the TIA.org study and the CHANCE subgroup analysis indicated that multiple infarctions was an important imaging marker for the risk of stroke recurrence in TIA and minor stroke at 90 days. Results of studies on large-artery stenosis showed that patients with acute multiple infarctions from artery-to-artery embolism had a high risk of recurrent stroke. [8,11-13] Therefore, the recurrence risk estimator at 90 days (RRE-90) identified multiple infarctions as an important imaging marker for risk assessment of stroke recurrence.

1.2 Current status of drug therapy for acute mild ischemic stroke

Despite the high incidence and recurrence of stroke, effective therapies are extremely limited for ischemic cerebrovascular disease. The medication of ischemic cerebrovascular disease mainly relies on thrombolytic therapy in the super acute stage, antithrombotic therapy in the acute stage and rehabilitation treatment. However, most patients with ischemic stroke are not eligible for thrombolytic therapy because

of its narrow "time window". Therefore, effective drug therapy is still needed to further reduce the risk of stroke recurrence in acute ischemic stroke. Existing evidence-based medicine includes:

1.2.1 Antiplatelet therapy

1.2.1.1 Dual antiplatelet therapy(DAPT) for patients with ischemic stroke within 24 hours of onset.

The antiplatelet drugs recommended by 2014 AHA/ASA Guidelines for the Prevention of Stroke in Patients with Stroke and Transient Ischemic Attack include aspirin, clopidogrel, compound preparation with aspirin and dipyridamole, and ticlopidine. [14] Cilostazol is recommended as secondary prevention therapy for non-cardiogenic ischemic stroke in Chinese Guidelines for the Prevention of Stroke in Patients with Stroke and Transient Ischemic Attack.

The CHANCE study enrolled 5170 patients with acute mild ischemic stroke (NIHSS socre ≤3) or high-risk TIA who were randomized and received antiplatelet therapy within 24 hours after symptom onset and found that the combined clopidogrel and aspirin continued for 21 days significantly decreased the incidence of stroke within 90 days, compared with aspirin monotherapy. (8.2% vs. 11.7%; HR 0.68; 95% CI, 0.57 to 0.81; P<0.001). [15]

1.2.1.2 Patients with ischemic stroke within 3 days of onset may also benefit from DAPT

A meta-analysis of 14 studies, including the CHANCE study, involving a total of 9,012 patients with ischemic stroke compared the efficacy and safety of dual antiplatelet therapy versus mono-antiplatelet therapy within 3 days of onset. The results suggested that dual antiplatelet therapy significantly reduced the risk of stroke recurrence (RR, 0.69; 95%CI, 0.60-0.80; P<0.001) and composite outcomes of stroke, TIA, acute coronary syndrome, and all-cause death (RR, 0.71; 95%CI 0.63-0.81; P<0.001) and there was no significant increase in the risk of major bleeding. [16] Our previous meta-analysis also found similar results.

The COMPRESS study enrolled patients with large-artery atherosclerotic stroke within 2 days of onset. The primary outcome was the presence of new ischemic lesion (both symptomatic and asymptomatic) on MRI at the 30th day. The results showed that, compared with aspirin alone, aspirin combined with clopidogrel did not reduce the new ischemic lesion on MRI at 30th day and did not increase the risk of bleeding. [17] However, some limitations of the study restrict the evaluation of efficacy, such as a small sample size and the lack of loading dose for clopidogrel at the early stage of administration, which may reduce the efficacy of the DAPT greatly.

1.2.1.3 Patients with atherosclerosis or multiple infarctions might benefit most from DAPT

The CARRESS study enrolled 230 SICAS patients with TIA and ischemic stroke. 107 patients with micro-embolism were randomized, and 51 patients were given clopidogrel and aspirin for 7 days and 56 patients were given aspirin alone for 7 days. [18] The results showed that DAPT significantly reduced the number of micro-embolism monitored by TCD compared to aspirin, without increased risk of intracranial hemorrhage. The CLAIR study enrolled 98 patients with TIA and ischemic stroke (NIHSS score was less than 8, symptomatic large-artery atherosclerotic stenosis with microembolism within 7 days of onset), and the results showed that the combination of clopidogrel and aspirin for 7 days significantly reduced the microembolism compared with aspirin, without increase in the risk of intracranial hemorrhage. [19] The

subsequent subgroup analysis of intracranial arterial stenosis found similar results.[20] The above two studies based on subclinical outcomes showed that DAPT is a potential treatment of SICAS and short-term treatment did not increase the risk of bleeding.

For patients with TIA or minor ischemic stroke with intracranial arterial stenosis, subgroup analysis of the CHANCE study showed that the HR of stroke was 0.79 (0.47-1.32) in the group of aspirin and clopidogrel treatment, and 1.12 (0.56 -2.25) in the group of aspirin treatment in patients with intracranial atherosclerosis in 90 days.[4] The P-value of interaction analysis was 0.522, which suggested that DAPT failed to effectively reduce the risk of stroke recurrence at 90 days in high risk patients with non-disabling cerebrovascular events of intracranial arterial stenosis. This subgroup had a small sample size, but the survival curve showed that intracranial arterial stenosis, rather than non-intracranial arterial stenosis, is likely to benefit more from DAPT compared with aspirin alone, which further provided important basis for clinical trials. The subgroup analysis also suggested that short-term DAPT did not increase the risk of bleeding. Another subgroup analysis of the CHANCE trail showed that multiple infarcts was an important imaging marker of stroke recurrence and patients with multiple cerebral infarctions and intracranial artery stenosis were at the highest risk of recurrence.[21] Meanwhile, patients with multiple cerebral infarctions may benefit most from DAPT. The risks of stroke recurrence were 10.1% and 18.8% respectively in the groups of DAPT and aspirin alone (HR: 0.5; 95%CI: 0.3-0.96; P=0.04), which suggested that patients with multiple cerebral infarctions may be the most suitable target population of DAPT, especially patients with intracranial arterial atherosclerotic stenosis.[22]

1.2.1.4 New oral antiplatelet agents and anticoagulants remain to be explored

The Acute Stroke or Transient Ischemic Attack Treated with Aspirin or Ticagrelor and Patient Outcomes (SOCRATES) trial published in 2016 enrolled minor stroke patients within 24 hours of onset and randomly assigned them to ticagrelor group and aspirin group. The patients were treated for 90 days and the primary outcomes were composite events (stroke, myocardial infarction, and death). The results showed that, for patients with high-risk TIA or minor ischemic stroke within 24 hours of onset, ticagrelor did not significantly reduce the 90-day combined risk of stroke, myocardial infarction and death compared with aspirin, and did not increase risk of any severe bleeding events. [23] A subgroup analysis of the SOCRATES trial showed that compared with aspirin, ticagrelor could reduce the risk of stroke recurrence in TIA and minor ischemic stroke of large-artery stenosis. However, the results were not recommended by guideline because it was a subgroup of analysis. [24]

1.2.2 Statin therapy

1.2.2.1 Secondary prevention study for statin

Stroke Prevention with Aggressive Reductions in Cholesterol Levels (SPARCL) trial is the only study of the efficacy and safety of statin in patients with TIA or ischemic stroke so far. The study enrolled 4731 patients with non-cardiogenic (without atrial fibrillation or other cardiogenic embolism) stroke and (or) TIA, and they were randomized to the treatment of atorvastatin 80 mg/d or placebo, with an average follow-up of 4.9 years. The results showed that the baseline level of serum low-density lipoprotein cholesterol (LDL-C) was 3.3-3.5mmol/L, and after treatment, the level of LDL-C decreased to

1.9mmol/L in atorvastatin group, and 3.4 mmol/L in the placebo group. Intensive atorvastatin therapy significantly reduced the risk of recurrent stroke in patients with stroke and (or) TIA by 16% (P = 0.03), and the risk of primary coronary events by 35% (P = 0.003). This benefit was achieved on the basis of active antihypertensive and antiplatelet therapy. [25]

The carotid stenosis subgroup of the SPARCL study [33] revealed that patients with stroke and (or) TIA combined with carotid stenosis may benefit more from intensive atorvastatin therapy.[26] In the subgroup analysis, intensive atorvastatin(80 mg/d) treatment significantly reduced the risk of recurrent stroke by 33% (P = 0.02), reduced the risk of coronary events by 42% (P < 0.01), and reduced the risk of carotid endarterectomy by 56% (P = 0.006) in 1007 patients (21%) with stroke and (or) TIA combined with carotid stenosis . SPARCL subgroup analyses according to different types of stroke showed that intensive atorvastatin (80 mg/d) had a different degree of benefit for stroke patients with different subtypes, while patients with large-artery atherosclerotic stenosis may benefit more from statins. [27]

1.2.2.2 Reversal effect of statins on carotid plaque

High resolution magnetic resonance imaging (HRMRI) has been widely used in monitoring the effect of statins on carotid atherosclerotic plaques. Early studies mainly focused on changes in atherosclerotic plaque burden (luminal area, wall area, vessel wall thickness, and normalized wall index) before and after medical therapy. [28-33] Most studies found that statin significantly decreased the LDL-C level in serum, and improved the atherosclerotic plaque burden significantly over a certain period of time. Underhill et al reported that after the treatment of rosuvastatin for 2 years, the LDL-C level decreased significantly in atherosclerotic patients and the proportion of the lipid-rich necrotic core (LRNC)in the wall was decreased by 41.4% than that of baseline (P = 0.005). [28] Du et al found that LRNC decreased by an average of 7.3% (P = 0.044) after the treatment of statin for 90 days, which suggested that statin therapy may stabilize atherosclerosis plaques in the first 90 days. [29]

1.2.2.3 Study on statin therapy in acute stage

In addition to their LDL-lowering effects, statins have been found to possess various cytoprotective benefits, including protection of endothelial function, antioxidant properties, and anti-inflammatory effects.[34] Preclinical studies have shown that administering statins immediately after a stroke can reduce the size of the infarct and improve neurological outcomes. [35,36]

Currently, the studies of statin therapy for ischemic cerebrovascular disease were based on cohort studies and randomized controlled exploratory trials of small sample size. The prospective North Dublin Population Stroke Study published in 2011 enrolled 448 patients with ischemic stroke, and the results showed that initiating statin therapy within 72 h after stroke was significantly associated with the decrease of short-term and long-term mortality. [37] A randomized controlled trial published in 2007 enrolled 89 ischemic stroke patients admitted within 24 hours of onset with chronic statin therapy. Patients were randomly assigned either to statin withdrawal for the first 3 days after admission or to immediately receive atorvastatin. The trial found that statin withdrawal was associated with increased risk of death or dependency at 90 days (OR, 4.66; 95% CI, 1.46-14.91).[38] A recent Meta-analysis found that statin administration during hospitalization was associated with good functional outcomes (OR, 1.31;

95%, CI, 1.12-1.53; P = 0.001). The Meta-analysis also revealed that the treatment of statins before stroke is related to better functional outcome (OR: 1.50; 95% CI: 1.29-1.75; P ＜ 0.001) and lower mortality (OR: 0.42; 95%; CI:0.21-0.82; P = 0.0108). Similar results were found in patients with large-artery atherosclerosis and small-artery occlusion, but was not found in patients with cardiogenic embolic stroke.[39]

A recent study enrolled 516 patients with acute ischemic stroke from large-artery atherosclerosis (TOAST subtype) and analyzed the impact of statin pretreatment on short-term outcomes. The study is a multicenter, prospective trial. The study concluded that statins pretreatment before stroke was independently associated with better functional outcomes (mRS score 0-1; OR: 2.44; 95%CI: 1.07-5.53), lower mortality (OR: 0.24; 95%CI: 0.08-0.75) and the rate of stroke recurrence (OR: 0.11; 95%CI: 0.22-0.46) within 1 month.[40] Therefore, this study suggests that statins pretreatment has a positive effect on prevention of stroke.

However, several small-sample randomized controlled trials have found no improvements in stroke recurrence and functional outcomes from early statin initiation. The EUREKA study included 316 patients with ischemic stroke in Korea, who received rosuvastatin with a dose of 20mg daily or placebo within 48 hours of onset and found no significant difference in occurrence of new ischemic lesions on DWI at 5 or 14 days between the two groups.[41] The STARS trial, a randomized controlled trial conducted in Spain, enrolled 104 patients with ischemic stroke with NIHSS score 4-22. The results suggested that simvastatin(40mg/d) started within 12 hours of onset did not improve the proportion of independent patients (mRS score≤2) at 90 days compared with placebo.[42] The ASSORT study conducted in Japan enrolled 257 patients with ischemic stroke (NIHSS score≤20) and randomly assigned to receive statin initiated within 24 hours or 7-day delayed statin, the results of which showed no significant difference in risk of disability between the two therapies.[43] This may be related to the small sample sizes of several studies, the variety of stroke causes enrolled, the severity of the disease, and the insufficient dosage of statins. **It is necessary to conduct large-scale randomized clinical trials for intensive statin therapy in the acute phase of large-artery atherosclerotic ischemic stroke.**

1.3 Current research questions and research hypotheses

1.3.1 Intensive antiplatelet therapy in patients with acute ischemic stroke or high-risk TIA from atherosclerosis

The INSPIRES study, in which patients with acute ischemic stroke or high-risk TIA from atherosclerosis were treated with antiplatelet therapy was conducted to answer the questions unsolved in the CHANCE study:

1）Whether patients with ischemic stroke from atherosclerosis with higher NIHSS scores may benefit from DAPT?

The CLAIR study expanded the NIHSS score of enrolled patients to 8, and the result showed no increased risk of bleeding after DAPT. The SOCRATES trial expanded the enrolled population NIHSS score to 5. Therefore, patients with high NIHSS score may benefit from DAPT. **The INSPIRES trial**

enrolled patients with expanded NIHSS score to 5.

2）Whether patients with ischemic stroke from atherosclerosis with extended time windows of onset may benefit from DAPT?

Meta-analysis published in 2013 showed that ischemic stroke patients enrolled within 72 hours of onset benefit from DAPT. **Therefore, the INSPIRES trial expands the time window of onset to 72 hours.**

3）Whether ischemic stroke with mild stenosis but multiple infarcts may benefit from DAPT?

The CHANCE imaging subgroup analysis showed that patients with multiple infarcts significantly benefited from DAPT, rather than patients with minor stroke with single infarction and TIA. Therefore, **multiple infarctions from embolization may be an important target for DAPT**.

The results of the pooled analysis of the CHANCE and the POINT trials suggested that DAPT with aspirin and clopidogrel initiated within 24 hours of onset in patients with noncardiogenic minor ischemic stroke (NIHSS score ≤ 3) or high-risk TIA (ABCD^2 score ≥ 4) significantly reduced the incidence of major ischemic events relative to aspirin alone, which was also cited by the 2019 American Heart Association/ American Stroke Association Guidelines for the Early Management of Acute Ischemic Stroke as a class A evidence recommendation. [15,44-46] Therefore, patients with non-cardiogenic mild ischemic stroke (NIHSS score≤3) or high-risk TIA (ABCD^2 score≥4) within 24 hours of onset were not included in this study.

1.3.2 Intensive lipid-lowering therapy in patients with acute ischemic stroke or high-risk TIA from atherosclerosis

The SPARCL study did not answer the question of the efficacy and safety of statin therapy for ischemic cerebrovascular disease in the acute phase, because it enrolled patients with ischemic stroke and TIA within 6 months of onset, with a mean time of 87 days. It remains unclear for immediate intensive statin therapy to reduce the early recurrence risk in patients with ischemic stroke from large-artery atherosclerosis.

Moreover, atorvastatin at doses of 40mg/d and 80mg/d are both recommended as intensive statin treatment in the 2018 American Heart Association/ American Stroke Association Guidelines for the Early Management of Patients with Acute Ischemic Stroke. [47] However, the difference between the two doses in terms of therapeutic effect has not been observed in Asian patients. Some studies have shown that statin intolerance varies in race. Compared with Western populations, Chinese have higher incidence of intensive statin intolerance, which makes it difficult for guideline promotion. [48] Therefore, it is necessary to discuss the efficacy and safety of intensive statin treatment with different doses of atorvastatin in acute phase of ischemic stroke. Meanwhile, 2018 American Heart Association/ American Stroke Association Guidelines for the Early Management of Patients with Acute Ischemic Stroke show that high-intensity statin therapy should be given in the acute phase for patients with TIA and ischemic stroke, but there is a lack of evidence on when to initiate the statin therapy. The ASSORT study explored the efficacy and safety of delayed statins, but due to its small sample size, it was not used as evidence

for guideline recommendations [47]. Hence, it is necessary to discuss the efficacy and safety of immediate or delay intensive statin treatment in reducing stroke recurrence and improving functional outcomes.

Based on the above evidence, we made the following 3 hypotheses: in patients with acute ischemic stroke or TIA with high-risk symptomatic intracranial or extracranial atherosclerosis, ①Intensive antiplatelet therapy reduces the risk of stroke recurrence at 90 days without increasing the risk of bleeding compared with standard antiplatelet therapy; ②Immediate intensive statin therapy (atorvastatin 80mg/d) reduces the risk of stroke at 90 days compared with delayed intensive statin therapy(atorvastatin 40mg/d); ③Intensive antiplatelet therapy combined with immediate intensive statin therapy(atorvastatin 80mg/d) reduces the risk of stroke at 90 days compared with standard antiplatelet therapy combined with delayed intensive statin therapy(atorvastatin 40mg/d).

Therefore, a randomized, double-blind, placebo-controlled, multicenter, 2*2 factorial design trial was conducted to evaluate the efficacy and safety of intensive antiplatelet therapy versus standard antiplatelet therapy in reducing the risk of new stroke at 90 days in acute mild ischemic stroke or high-risk TIA patients attributed to intracranial or extracranial arterial atherosclerosis; to evaluate the efficacy and safety of immediate intensive statin therapy versus delayed intensive statin therapy in reducing the risk of stroke at 90 days in acute mild ischemic stroke or high-risk TIA patients attributed to intracranial or extracranial arterial atherosclerosis; To evaluate the effectiveness of intensive antiplatelet therapy combined with immediate intensive statin therapy versus standard antiplatelet therapy combined with delayed intensive statin therapy in reducing the risk of stroke at 90 days in acute mild ischemic stroke or high-risk TIA patients attributed to intracranial or extracranial arterial atherosclerosis.

2. Study Purposes

2.1 Primary purposes:

2.1.1 To evaluate the efficacy and safety of intensive antiplatelet therapy versus standard antiplatelet therapy in reducing the risk of new stroke at 90 days in acute mild ischemic stroke or high-risk TIA patients attributed to extracranial or intracranial atherosclerosis.

2.1.2 To evaluate the efficacy and safety of immediate intensive statin therapy (atorvastatin 80mg/d) versus delayed intensive statin therapy (atorvastatin 40mg/d) in reducing the risk of new stroke at 90 days in acute mild ischemic stroke or high-risk TIA patients attributed to extracranial or intracranial atherosclerosis.

2.1.3 To evaluate the efficacy and safety of intensive antiplatelet combined with immediate intensive statin therapy (atorvastatin 80mg/d) versus standard antiplatelet combined with delayed intensive statin therapy (atorvastatin 40mg/d) in reducing the risk of new stroke at 90 days in acute mild ischemic stroke or high-risk TIA patients attributed to extracranial or intracranial atherosclerosis.

Notes:

- Intensive antiplatelet therapy refers to dual antiplatelet therapy with aspirin and clopidogrel;
- Standard antiplatelet therapy refers to antiplatelet monotherapy with aspirin;
- Immediate intensive statin therapy (atorvastatin 80mg/d) refers to lipid-lowering therapy from the onset with atorvastatin at a dose of 80 mg daily and last for the first 21 days, followed by 40 mg daily for days22-90;
- Delayed intensive statin therapy (atorvastatin 40mg/d) refers to lipid-lowering therapy with atorvastatin at a dose of 40 mg for days 4-90);
- Acute mild ischemic stroke or high-risk TIA patients attributed to extracranial or intracranial atherosclerosis: at least one of the following characteristics:

a) Mild ischemic stroke (NIHSS score 4–5) within 24 hours after onset and either of the following imaging characteristics:

1) Acute single infarction with ≥50% stenosis of a major intracranial or extracranial artery that likely accounts for the infarction and clinical presentation.

2) Acute multiple infarctions documented by head CT or MRI, attributed to large-artery atherosclerosis, including non-stenotic vulnerable plaques.

b) Mild ischemic stroke (NIHSS score ≤5) or high-risk TIA (ABCD^2 score ≥4) within 24-72 hours after onset and meet any of the following imaging characteristics:

1) TIA with ≥50% stenosis of a major intracranial or extracranial artery that likely accounts for the clinical presentation.

2) Acute single infarction with ≥50% stenosis of a major intracranial or extracranial artery that likely accounts for the infarction and clinical presentation.

3) Acute multiple infarctions documented by head CT or MRI, attributed to large-artery atherosclerosis, including non-stenotic vulnerable plaques.

2.2 Secondary purposes:

2.2.1 To evaluate the efficacy of intensive antiplatelet therapy versus standard antiplatelet therapy, immediate intensive statin therapy (atorvastatin 80mg/d) versus delayed intensive statin therapy

(atorvastatin 40mg/d), intensive antiplatelet therapy combined with immediate intensive statin therapy (atorvastatin 80mg/d) versus standard antiplatelet therapy combined with delayed intensive statin therapy respectively (atorvastatin 40mg/d) at 90 days in acute mild ischemic stroke or high-risk TIA patients attributed to intracranial or extracranial atherosclerosis on the incidence of (definitions shown in appendix 1, 2 and 5):

- Combined vascular events: stroke (ischemic or hemorrhagic), myocardial infarction, and cardiovascular death.
- Ischemic stroke
- TIA
- Severity of stroke or TIA on an ordinal scale: a six-level ordered category scale combined vascular events with mRS score at 90 days: fatal stroke (stroke with subsequent death), severe stroke (stroke followed by mRS of 4-5), moderate stroke (stroke followed by mRS of 2-3), mild stroke (stroke followed by mRS of 0-1), TIA, and no stroke/TIA
- Myocardial infarction
- Vascular death
- All-cause death
- Poor functional outcome (mRS score 2-6)
- Poor quality of life (EQ-5D scale index score≤0.5)

2.2.2 To evaluate the efficacy in decreasing early neurological deficits (increase in NIHSS score at 7days), stroke recurrence, all-cause mortality and poor functional outcome (mRS score 2-6) at 1 year of intensive antiplatelet therapy versus standard antiplatelet therapy, immediate intensive statin therapy (atorvastatin 80mg/d) versus delayed intensive statin therapy (atorvastatin 40mg/d), intensive antiplatelet therapy combined with immediate intensive statin therapy (atorvastatin 80mg/d) versus standard antiplatelet therapy combined with delayed intensive statin therapy (atorvastatin 40mg/d) respectively in acute mild ischemic stroke or high-risk TIA patients attributed to intracranial or extracranial atherosclerosis. The new stroke or TIA is classified on a six-level ordered category scale combined vascular events with mRS score at 1 year: fatal stroke (stroke with subsequent death), severe stroke (stroke followed by mRS of 4-5), moderate stroke (stroke followed by mRS of 2-3), mild stroke (stroke followed by mRS of 0-1), TIA, and no stroke/TIA.

2.3 Safety purposes

2.3.1 To evaluate safety of intensive antiplatelet therapy versus standard antiplatelet therapy for 90 days in acute mild ischemic stroke or high-risk TIA patients attributed to intracranial or extracranial atherosclerosis on the incidence of:

- Moderate to severe bleeding (Appendix 6)
- Intracranial hemorrhage
- Hepatotoxicity: Alanine aminotransferase (ALT) or Aspartate aminotransferase (AST) > 3 times the upper limit of normal value.

- Muscle toxicity: Creatine kinase (CK) > 10 times the upper limit of normal value, or the presence of muscle pain, myopathy, or rhabdomyolysis.
- Death
- Other adverse events (AEs) / severe adverse events (SAEs)

2.3.2 To evaluate safety of immediate intensive statin therapy(atorvastatin 80mg/d) versus delayed intensive statin therapy (atorvastatin 40mg/d) for 90 days in acute mild ischemic stroke or high-risk TIA patients attributed to intracranial or extracranial atherosclerosis on the incidence of:

- Moderate to severe bleeding (Appendix 6)
- Intracranial hemorrhage
- Hepatotoxicity: Alanine aminotransferase (ALT) or Aspartate aminotransferase (AST) > 3 times the upper limit of normal value.
- Muscle toxicity: Creatine kinase (CK) > 10 times the upper limit of normal value, or the presence of muscle pain, myopathy, or rhabdomyolysis.
- Death
- Other AEs / SAEs

2.3.3 To evaluate the safety of intensive antiplatelet therapy combined with immediate intensive statin therapy (atorvastatin 80mg/d) versus standard antiplatelet therapy combined with delay intensive statin therapy (atorvastatin 40mg/d) for 90 days in acute mild ischemic stroke or high-risk TIA patients attributed to intracranial or extracranial atherosclerosis on the incidence of:

- Moderate to severe bleeding (Appendix 6)
- Intracranial hemorrhage
- Hepatotoxicity: ALT or AST > 3 times the upper limit of normal value.
- Muscle toxicity: CK > 10 times the upper limit of normal value, or the presence of muscle pain, myopathy, or rhabdomyolysis.
- Death
- Other adverse events / severe adverse events

2.4 Subgroup analysis

2.4.1 To evaluate the efficacy of intensive antiplatelet therapy versus standard antiplatelet therapy, immediate intensive statin therapy (atorvastatin 80mg/d) versus delayed intensive statin therapy (atorvastatin 40mg/d), and intensive antiplatelet combined with immediate intensive statin therapy (atorvastatin 80mg/d) versus standard antiplatelet combined with delayed intensive statin therapy (atorvastatin 40mg/d) in reversing intracranial atherosclerotic artery stenosis and stabilizing the atherosclerotic vulnerable plaque in the high resolution MRI subgroup.

2.4.2 To evaluate the efficacy of intensive antiplatelet therapy versus standard antiplatelet therapy for 90 days on the incidence of the primary outcome in different subgroups:

- Subjects aged > 65 years vs. those aged ≤65 years.
- Female vs. male patients.

- Those with NIHSS score 4-5 vs. those with NIHSS score ≤3 at admission.
- Those randomized within 24 hours of onset vs. those randomized between 24 and 72 hours since onset.
- Those with ischemic stroke/TIA related to extracranial artery atherosclerosis vs. those related to intracranial artery atherosclerosis.
- Those with intracranial stenosis vs. those without intracranial stenosis.
- Those with extracranial stenosis vs. those without extracranial stenosis.
- Those with multiple infarctions vs. those with single infarction vs. those without infarction.
- Those with severe stenosis (≥70%) vs. those with moderate stenosis (50%-69%).
- Those with hypertension vs. those who are normotensive.
- Diabetic patients vs. nondiabetics.
- Those with dyslipidaemia vs. without dyslipidaemia.
- Those with atherogenic dyslipidaemia (HDL-C<40 mg/dL and TG>200 mg/dL) vs. without atherogenic dyslipidaemia.
- Those with statin therapy within 1 month before randomization vs. without statin therapy.

In addition, relevant subgroups will be examined for genetic variability and biomarker characteristics.

2.4.3 To evaluate the efficacy of immediate intensive statin therapy (atorvastatin 80mg/d) versus delayed intensive statin therapy (atorvastatin 40mg/d) for 90 days on the incidence of the primary outcome in subgroups which is the same as those in 2.4.2.

3. Study Design

3.1 Study design

- A Randomized, double-blind, placebo-controlled, multicenter, 2×2 factorial trial
- The trial is intended to enroll 6100 subjects and complete follow-up of all subjects within 5 years.
- Centralized, unified and randomized grouping.
- Subjects will be randomly assigned to the following four groups:

  A: Intensive antiplatelet therapy +immediate intensive statin therapy (atorvastatin 80mg/d)

  B: Intensive antiplatelet therapy +delayed intensive statin therapy (atorvastatin 40mg/d)

  C: Standard antiplatelet therapy +immediate intensive statin therapy (atorvastatin 80mg/d)

  D: Standard antiplatelet therapy +delayed intensive statin therapy (atorvastatin 40mg/d)

Identity of study medication:

Groups	Date after Randomization	Dosage of study medication
Intensive antiplatelet therapy	Day 1	Clopidogrel 300mg/ day + aspirin 100-300mg/ day
	Day 2 - Day21±2	Clopidogrel 75mg/ day + aspirin 100mg/ day
	Day22±2 - Day 90	Clopidogrel 75mg/ day + aspirin placebo
Standard antiplatelet therapy	Day 1	Aspirin 100-300mg/ day + clopidogrel placebo
	Day 2 - Day 90	Aspirin 100mg/ day + clopidogrel placebo
Immediate intensive statin therapy	Day 1 - Day21±2	Atorvastatin 80mg/ day
	Day22±2 - Day 90	Atorvastatin 40mg/ day
Delayed intensive statin therapy	Day 1 - Day3	Atorvastatin placebo
	Day 4 - Day21±2	Atorvastatin 40mg/ day + atorvastatin placebo
	Day22±2 - Day 90	Atorvastatin 40mg/ day

Research design (drawing)

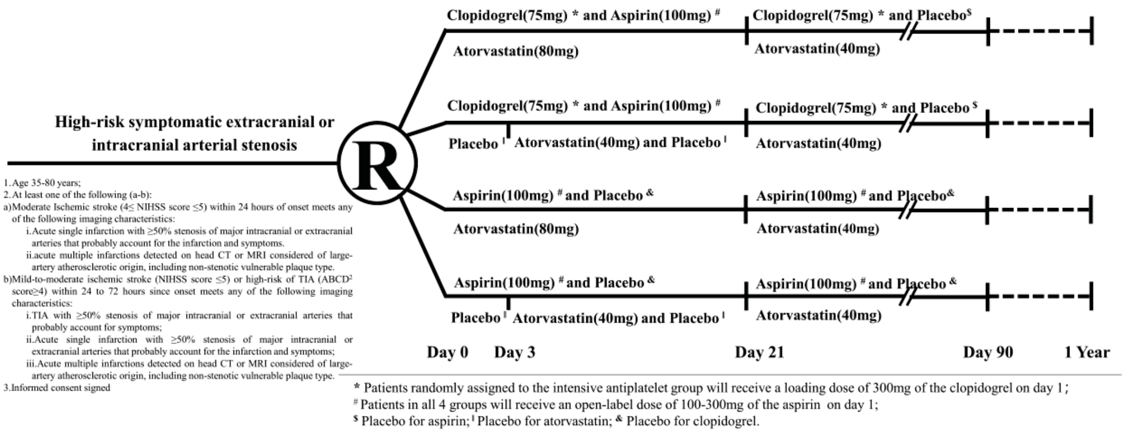

3.2 Follow-up plan

Subjects will receive a face-to-face visit at baseline, Day7±2, Day14 (or hospital discharge) and Day90 ± 7, and a telephone visit at the 12th month±14 days after randomization. In addition, patients will be interviewed when new neurologic symptoms or suspicious events occur, including worsening of index ischemic events, new transient or persistent neurological symptoms.

3.3 Study execute time

Project Work Description	Estimated Completion Time
Study design, ethical review, study registration, sub-center recruitment	2018-06
The project is officially launched and the first subject is enrolled	2018-07
All subjects are enrolled and grouped	2021-12
90-day follow-up are finished for all subjects	2022-03
Data cleaning and database locking	2023-03
Clinical trial report completion	2023-06
Results publication	2023-12

4. Study Outcomes

4.1 Primary outcome

Stroke (ischemic or hemorrhagic)

4.2 Secondary outcomes:

- Combined vascular events： Stroke (ischemic or hemorrhagic), myocardial infarction, or cardiovascular death
- Ischemic stroke
- TIA
- Severity of stroke or TIA on an ordinal scale: (a six-level ordered category scale combined vascular events with mRS score: fatal stroke (stroke with subsequent death), severe stroke (stroke followed by mRS of 4-5), moderate stroke (stroke followed by mRS of 2-3), mild stroke (stroke followed by mRS of 0-1), TIA, and no stroke/TIA)
- Myocardial infarction
- Vascular death
- All-cause death
- Poor functional outcome (mRS score 2-6)
- Poor quality of life (EQ-5D scale index score≤0.5)
- Early neurological deficits (increase in NIHSS score at 7days)

4.3 Primary safety outcome

- Moderate to severe bleeding (Appendix 6)

4.4 Secondary safety outcomes

- Intracranial hemorrhage
- Hepatotoxicity: ALT or AST > 3 times the upper limit of normal value;
- Muscle toxicity: CK > 10 times the upper limit of normal value, or the presence of muscle pain, myopathy, or rhabdomyolysis.
- Death
- Other AEs / SAEs

5. Subjects Selection

5.1 Inclusion Criteria

1. Age :35-80 years;
2. At least one of the followings (a-b):
   a) Mild Ischemic stroke (NIHSS score 4-5) within 24 hours after onset and either of the following imaging characteristics:
      i. Acute single infarction with ≥50% stenosis of a major intracranial or extracranial artery that likely accounts for the infarction and clinical presentation.
      ii. Acute multiple infarctions documented by head CT or MRI, attributed to large-artery atherosclerosis, including non-stenotic vulnerable plaques.
   b) Mild ischemic stroke (NIHSS score ≤5) or high-risk of TIA ($ABCD^2$ score≥4) within 24-72 hours after onset and meet any of the following imaging characteristics:
      i. TIA with ≥50% stenosis of a major intracranial or extracranial artery that likely accounts for the clinical presentation;
      ii. Acute single infarction with ≥50% stenosis of a major intracranial or extracranial artery that likely accounts for the infarction and clinical presentation;
      iii. Acute multiple infarctions documented by head CT or MRI, attributed to large-artery atherosclerosis, including non-stenotic vulnerable plaques.

4. Written informed consent.

Notes:

- Intracranial large arteries include the intracranial portion of internal carotid arteries, middle cerebral arteries (M1/M2) , anterior cerebral arteries (A1/A2), posterior cerebral arteries (P1/P2), intracranial portion of vertebral arteries, and basilar artery; [49,50] The degree of intracranial artery stenosis is defined on MRA, CTA or DSA by criteria from the Warfarin–Aspirin Symptomatic Intracranial Disease (WASID) study.[49]
- Extracranial large arteries include the extracranial portion of carotid arteries and vertebral arteries (including the portion from initiation of carotid arteries and vertebral arteries to the start of intracranial section, except the subclavian artery, aortic arch and brachiocephalic trunk). The degree of extracranial arteries stenosis is defined on carotid duplex ultrasound, CEMRA, CTA, or DSA by criteria from the North American Symptomatic Carotid Endarterectomy Trial (NASCET).[51]
- Acute ischemic stroke with multiple infarction lesions refers to more than one lesion appearing in different locations (separated in space or non-continuous on contiguous slices) on diffusion DWI/ADC imaging however, it will be considered as the same one if the lesion is continuous on the adjacent levels.[3]
- TIA: Sudden neurologic deficit lasting less than 24 hours, without non-ischemic causes.
- TIA patients with acute multiple cerebral infarctions will also be enrolled.

5.2 Exclusion criteria

1. Presumed cardioembolic stroke or TIA (e.g. atrial fibrillation, heart valve prosthesis, atrial myxoma, endocarditis, etc.).
2. Other determined etiology of stroke or TIA (e.g. aortic dissection, cervico-cerebral artery dissection, vasculitis, vascular malformation, moyamoya disease/syndrome, fibromuscular dysplasia, etc.).
3. Non-vascular neurological diseases (e.g. intracranial tumor, multiple sclerosis, etc.).
4. Index infarction affects ≥ 50% of a cerebral lobe (e.g. parietal, frontal, occipital).
5. Hemorrhagic transformation after onset.
6. Contraindications to clopidogrel, aspirin or atorvastatin:
    a. History of hypersensitivity;
    b. Severe heart failure (NYHA classification: III- IV) or asthma;
    c. Coagulation disorder or systemic bleeding;
    d. History of drug-induced hematologic or hepatic abnormalities;
    e. Leukopenia (< 2×10^9/L) or thrombocytopenia (<100×10^9/L);
    f. Active liver disease;
    g. Pregnancy or lactation period;

***Severe heart failure：** New York Heart Association (NYHA) Classification III and IV

I Cardiac disease, but no symptoms and no limitation in ordinary physical activity, e.g. no shortness of breath when walking, climbing stairs etc.

II (mild heart failure) Mild symptoms (mild shortness of breath and/or angina) and slight limitation during ordinary activity.

III (moderate heart failure) Marked limitation in activity due to symptoms, even during less-than-ordinary activity, e.g. walking short distances (20–100 m). Comfortable only at rest.

IV (Severe heart failure) Severe limitations. Experiences symptoms even while at rest. Mostly bedbound patients.

7. Pre-existing disability with mRS score>2.
8. Intra- arterial or intravenous thrombolysis or endovascular therapy after onset.
9. Defibrinogen therapy (e.g. defibrase and lumbrokinase), anticoagulation therapy (e.g. argatroban) , or antiplatelet therapy (e.g. ticagrelor, tirofiban) except for clopidogrel and aspirin after onset.
10. Creatine kinase > 5 times the upper limit of normal value of onset.
11. Drug use related to statin metabolism within 14 days before randomization (e.g. immune-suppressive drugs, antifungal agents, fibrates).
12. Severe hepatic insufficiency (ALT or AST > 2 times the upper limit of normal value) or renal insufficiency (creatinine > 1.5 times the upper limit of normal value or glomerular filtration rate < 40 ml/min/1.73 m2).
13. Dual antiplatelet therapy with aspirin and clopidogrel within 14 days before randomization (Patients who started aspirin plus clopidogrel without loading dose(300mg) of clopidogrel after onset are not excluded from the trial.).
14. High-intensity statin therapy within 14 days before randomization (e.g. atorvastatin ≥ 40mg/d,

rosuvastatin ≥ 20mg/d).

15. History of intracranial hemorrhage (e.g. intracerebral or subarachnoid hemorrhage).
16. Gastrointestinal bleeding or major surgery within 90 days.
17. History of intracranial or extracranial angioplasty.
18. Planned long-term use of antiplatelet drugs or non-steroidal anti-inflammatory drugs except for study drugs.
19. Planned surgery or revascularization that may need to stop taking the study drugs within the next 90 days.
20. Anticipated life expectancy < 90 days.
21. Currently participating in any other investigational drug or device study.
22. Unable to complete the follow-up (e.g. dementia, alcoholism, substance abuse, severe mental disease).

6. Therapeutic Regimen

6.1 Drug information and specification

Drugs	Specifications	Dosage form	Administra-tion route	Production Company	Storage	Term of validity
Clopidogrel	75 mg	tablet	oral	Sanofi Winthrop Industries	No special requirement	36months
Aspirin	100 mg	tablet	oral	Bayer S.p.A.	Below 25°C	36 months
Atorvastatin	20mg	tablet	oral	Beijing Jialin Pharmaceutical CO.,LTD	Below 25°C	36 months

6.2 Drug dosage

Groups	Date after Randomization	Dosage of study medication
Intensive antiplatelet therapy	Day1	Clopidogrel 300mg/day+asprin100-300mg/day
	Day2 - Day21±2	Clopidogrel 75mg/day+ aspirin 100mg/day
	Day22±2 - Day90	Clopidogrel 75mg/ day + aspirin placebo
Standard antiplatelet therapy	Day1	Aspirin 100-300mg/ day + clopidogrel placebo
	Day2 - Day90	Aspirin 100mg/day+ clopidogrel placebo
Immediate intensive statin therapy	Day1 - Day21±2	Atorvastatin 80mg/day
	Day22±2 - Day90	Atorvastatin 40mg/day
Delayed intensive statin therapy	Day1 - Day3	Atorvastatin placebo
	Day4 - Day21±2	Atorvastatin 40mg/day + atorvastatin placebo
	Day22±2 - Day90	Atorvastatin 40mg/day

6.3 Regimen

Administration: oral; The final 4 treatment groups according to the 2×2 factorial design are as follows (enrolled patients were randomized into the following 4 groups, blinded for both investigators and patients):

A: Intensive antiplatelet therapy + immediate intensive statin therapy (atorvastatin 80mg/d)

B: Intensive antiplatelet therapy + delayed intensive statin therapy (atorvastatin 40mg/d)

C: Standard antiplatelet therapy + immediate intensive statin therapy (atorvastatin 80mg/d)

D: Standard antiplatelet therapy + delayed intensive statin therapy (atorvastatin 40mg/d)

7. Study Implementation

7.1 Randomization procedures

Random number lists will be generated by the Statistics and Data Center at the China National Clinical Research Center for Neurological Diseases (Beijing, China). Regimens for patients will be packed according to the random code lists. Patients will be assigned to four groups with the approximate ratio of 1:1:1:1, and receive: A intensive antiplatelet therapy+ immediate intensive statin therapy (atorvastatin 80mg/d), B intensive antiplatelet therapy+ delayed intensive statin therapy (atorvastatin 40mg/d), C standard antiplatelet therapy+ immediate intensive statin therapy (atorvastatin 80mg/d), or D standard antiplatelet therapy+ delayed intensive statin therapy (atorvastatin 40mg/d) respectively. Randomization will be centralized. We applied randomized block design to generate random number to ensure the ratio between the four groups will be approximately 1:1:1:1. If a center runs out of its random code lists, a new block will be placed to the center for new random numbers. Each patient eligible to the INSPIRES trial will be assigned a random number in order strictly and the corresponding study drug box.

7.2 Blinding and Emergency Unblinding

7.2.1 Description of Blinding

Two clopidogrel tablets (75mg clopidogrel and matching placebo) used in this trial were indistinguishable (they were identical in size, shape, color, appearance and smell).

Two aspirin tablets (100mg aspirin and matching placebo) used in this trial were indistinguishable (they were identical in size, shape, color, appearance and smell).

Two atorvastatin tablets (20mg atorvastatin and matching placebo) used in this trial were indistinguishable (they were identical in size, shape, color, appearance and smell).

Centers are not able to apply unblinding with biological experiment in this study. Investigators shall never unblind the random code unless special situations occur such as SAE, when disclosure of the study drug information is important for the treatment of the subject. Efficacy and safety outcomes will be submitted to Adjudication Committee, who should be blinded to the randomization, for the final determination.

7.2.2 Emergency unblinding procedure

Emergency unblinding should only be undertaken when it is essential to identify the study drug information for effective treatment of the patient who suffer from AEs or SAEs. Contact should be made with the Monitoring Committee in advance if possible.

If the investigator finds it essential for the treatment to perform unblinding, he/she should inform the Monitoring Committee and sponsor as soon as possible. All calls should be recorded by the Contract Research Organization (CRO).

The date, time and reason for unblinding should be documented by the investigator and study drug must be discontinued after emergency unblinding.

7.3 Study drug management

7.3.1 Study drug supply and storage

Clopidogrel, aspirin, atorvastatin calcium, and placebo for the study should be purchased by sponsors. The study drug will be stored in a safe place, under the direct management of the investigator or other authorized persons, and stored under the conditions described on the drug label.

7.3.2 Study drug packaging and labeling

7.3.2.1 Study drug packaging

According to the design of the study protocol and follow-up period, the study drugs will be divided into two boxes according to the different phases of the trial, i.e. box 1 and box 2. Each box will contain three study drugs respectively, as follows.:

- **Box1 will provide the drug from day1 to day21 after randomization**
  a) Active clopidogrel (75mg/ tablet) or clopidogrel placebo without open label. A total of 4 plates (7 tablets/ plate). Patients should take 4 tablets on the first day and 1 tablet per day for the rest days.
  b) Active aspirin (100mg/tablet) . A total of 2 plates (15 tablets/ plate). 1-3 tablets should be taken under the direction of doctors on the first day and 1 tablet per day for the rest days.
  c) Active atorvastatin (20mg/tablet) or atorvastatin placebo without open label. There are two types of packaging: 2 plates (7 tablets/ plate) of active atorvastatin or atorvastatin placebo, and 20 bags (4 tablets/ bag) of active atorvastatin or mixture of 2 tablets of active atorvastatin and 2 tablets of atorvastatin placebo. Plates are used for days1 to 3 at a dose of 4 tablets daily. Bags are used for days 4 to 21 at a dose of 1 bag daily.
- **Box 2 will provide the drug from day 22 to day 90 after randomization**
  a) Active clopidogrel (75mg/tablet) or clopidogrel placebo without open label. A total of 11 plates (7 tablets/ plate). Patients need to take 1 tablet per day.
  b) Active aspirin (100mg/tablet) or aspirin placebo without open label. A total of 5 plates (15 tablets/ plate). Patients need to take 1 tablet per day.
  c) Active atorvastatin (20mg/tablet). A total of 21 plates (7 tablets/ plate), patients need to take 2 tablets per day.

Finally, Box1 and Box2 will be packed in a bigger box, which will be randomized and dispensed for each patient.

7.3.2.2 Drug label

The drug box label should contain:

- Abbreviation of the study and logo of the study
- Random code
- Time of medication use, like box1: “drug used at day 1-21 after randomization”, box2: “drug used at day 22-90 after randomization”; The medication bags of atorvastatin should be marked

with: “drug used at day 4-21 after randomization”

- Dosage form, dosage and administration route of the study drug
- Instructions for usage
- Storage conditions
- Expiration date
- “Please take the medication with the instruction of the doctor and bring the remaining tablets back for counting at the next visit”
- “Keep away from children to avoid accidental ingestion by children”
- “For the INSPIRES trial use only”

7.3.3 Responsibility for drug management

Investigators, pharmacist and anyone who store and dispense the drug should make sure the study drugs are stored and dispensed safely in accordance with the demands of the sponsor and the protocol as well.

It is investigators’ responsibility to make sure the study drug be dispensed in accordance with the protocol and document the dispensing and retrieving properly.

Any quality defect in dispensing and usage of the study drug (including appearance, labeling, validity period etc.) should be reported to the sponsors in time.

In no circumstance should the study drug be given to the third party. The study drug cannot be used or handled by any means other than as required by the clinical trial protocol.

7.3.4 Compliance of treatment and reliability

The information of the usage of all drugs including study medication and concomitant medication should be recorded in the appropriate location on the CRF. Subjects will receive drugs for the first period that is sufficient for the first 21 days after randomization. On the day 21±2, the patient will receive the second period drugs that is sufficient to the end of the study. At day 21±2 exchange of study drug and day 90±7 visit, subjects should take back the residual study drug and the empty package. Pill count should be applied for the retrieving drug and documented in the CRF.

Compliance will be accessed by the investigator at each visit using pill-counting for the retrieving drug.

Investigator should record the usage of study and concomitant medication for individual in the corresponding page in CRF.

Time of discontinuation and recovery should be documented.

7.3.5 Return and destruction of the drug

Investigators (or pharmacists) should establish a detailed record for return of the study drugs and investigators and the monitoring team should jointly confirm the record.

Investigators shall not destroy the study drugs (including not used and partly used), unless being authorized in writing by the principal investigator.

Investigators will adopt the recall procedure to recall study drugs if any defect were found in quality. It is the responsibility of the investigator to comply with any requests made by the principle investigator in order to recall the study drug and eliminate potential hazards.

7.4 Concomitant medication and intervention

Any interventions (including medication, surgery, vascular interventions,) performed after randomization, as well as any medication changes during the study should be documented in the CRF.

7.4.1 Concomitant medication contraindicated

1) Anti-platelet therapy: Ticagrelor, dipyridamole, cilostazol. ticlopidine, prasugrel, GPIIb/IIIa receptor antagonist, ozagrel etc.

2) All forms of anticoagulant drugs.

3) Thrombolytic therapy: rt-PA, urokinase and streptokinase etc. Patents who received intravenous or intra-arterial thrombolysis within 24 hours before randomization should not be included. If patients received intravenous or intra-arterial thrombolysis after randomization, the study antiplatelet agents must be discontinued for at least 24 hours before restart. While atorvastatin in the study is not affected.

4) Batroxobin, defibrase, snake venom preparation, lumbrukinase etc.

5) Non-steroidal anti-inflammatory drugs (NSAIDs, inhibitors of Cox1 and Cox2). Usage of NSAIDs (inhibitors of Cox1 and Cox2) within 7 days after randomization is violation to the protocol. If necessary, NSAIDs should be initiated after 24 hours after discontinuation of study drugs for less than 5 days, and should not be used within 8 days after randomization.

6) Potent inhibitor of CYP3A: Ketoconazole, nefazodone, ritonavir, saquinavir, atazanavir, nelfinavir, itraconazole, voriconazole, clarithromycin, telithromycin except for erythromycin and azithromycin;

7) Potent inducer of CYP3A: Rifampicin, dexamethasone, phenytoin, carbamazepine, oxcarbazepine, phenobarbitone;

8) Potent inhibitor of CYP2C19: Omeprazole, esomeprazole, fluvoxamine, fluoxetine, moclobemide, fluconazole, naphthyridine, ciprofloxacin, cimetidine, chloromycin;

9) Potent inhibitor of P-glycoprotein (P-gp): Verapamil, quinidine, cyclosporine;

10) Substrates of P-glycoprotein (P-gp): Digoxin. If necessary, If digoxin is absolutely necessary, reduce the digoxin dose as appropriate and monitor digoxin blood levels closely;

11) Other statins, drugs or food that affect the metabolism of statins: Rosuvastatin, simvastatin, pravastatin, pitavastatin, lovastatin, fiber acid derivative (fibrates), niacin or immunosuppressive regulating the lipid, oral contraceptive, hypocol.

12) Other relative drugs: evening primrose oil, curcumin, capsaicin staggerweed, ginkgo, garlic, the root of red-rooted salvia, cholestyramine.

13) Chinese patent drug： Chinese medicines with antiplatelet and antithrombotic effects in the instructions, including tablets and injections.

7.4.2 Concomitant medication permitted

Medications not listed in 7.4.1 can be used in this study. Patients with comorbidities (such as hypertension, diabetes, coronary heart disease, seizures etc.) can use diuretic, beta-blockers, ACEI, ARB, CCB, vasodilator, anti-diabetes agents (including insulin), anti-seizures agents and so on. The diagnosis, medications, dosage and administration should be documented in the CRF in detail. When other

treatments have to be added due to special circumstances (e.g., another disease during the study period), they must be described in the case report form, including the diagnosis, medications, dosage and administration.

1) When an emergency occurs, such as acute coronary syndrome (ACS) or when percutaneous coronary intervention (PCI) is necessary to intensify antiplatelet therapy, the dose of antiplatelet agents could be increased temporally. The study dose will be restored after the condition has stabilized, and the drug adjustment will be recorded in the CRF.

2) H2 receptor blockers: All except cimetidine.

3) Proton pump inhibitors (PPIs): PPIs could be used if necessary except omeprazole and lansoprazole, for example, dexlansoprazole, lansoprazole, pantoprazole.

4) 5-serotonin reuptake inhibitors (SSRIs): When using paroxetine, sertraline and citalopram, attention should be paid on preventing hemorrhage due these drugs may increase the risk of the hemorrhagic event.

7.4.3 Surgery and invasive procedures

If surgery or invasive procedures (including vascular surgery, angiography or angioplasty of coronary, internal and external carotid artery) is necessary within 90 days after randomization: for procedures with major hemorrhagic risk, the study drug should be discontinued for at least 5 days prior to the procedure and restarted when the investigator deems the risk of bleeding to be low after operation. For other surgical/ invasive procedures, the study drug should be continued or suspended according to the investigator's judgment.

7.5 Temporary, permanent discontinuation of the study drug(s) or loss to follow-up

Study drug should be maintained as long as possible. Judgment for temporary discontinuation should be made when facing withdrawal. Permanent discontinuation is the last resort. Any forms of discontinuation of the study drug should be documented in CRF in detail. Subjects should participate in the study as long as possible in any situations. After randomization, study drug discontinuation for any reason does not constitute withdrawal from the study or stopping follow-up, evaluation and visit should be continued.

7.5.1 Temporary discontinuation of study drug

If the study drug is temporarily discontinued, it should be restarted as soon as medically justified in the opinions of the investigators.

Study drug should be temporarily discontinued in the situations listed below:

1) Severe thrombocytopenia (platelet count <50,000/uL, 50×10^9/L). The study drug should be restarted once the patient recovers from the severe thrombocytopenia.

2) Severe bleeding events (shown in appendix 6)

3) Use of contraindicated concomitant medications (listed in 7.4.1).

4) Operation and procedures probably causing major bleeding (listed in 7.4.3).

5) Abnormal liver function (transaminase level above twice the normal value). The study drug should be restarted to patients once the transaminase level is back to normal.

For other operation and procedures, continuation and discontinuation should be justified in the opinions of the investigators.

Once investigators justify the study drug unrelated to adverse events and with no contraindication for discontinuation, study drugs should be restarted to the patient as soon as possible in close and reasonable monitoring. The time of temporary discontinuation of study drug should be carefully documented in the CRF.

7.5.2 Discontinuation from the study drug permanently

1) Patient's decision. Patients can discontinue the study anytime they want unless it will affect their treatment.

2) Investigator's decision. Including but not limited to the following circumstances:

h) Primary outcomes (ischemic or hemorrhage stroke) occur
i) Pregnancy or preparation for pregnancy
j) Incorrect enrollment that does not meet the inclusion/exclusion criteria puts the patient at higher risk
k) Serious noncompliance
l) Emergence of adverse events that investigators believe may put patients at higher risk of continuing treatment
m) Definite indications for anticoagulant agent
n) Investigators decide it is inappropriate to restart the study drug after discontinuation

7.5.3 Measures after permanent discontinuation

The investigators should provide follow-up medical care for all patients who are permanently discontinuation from the study, and should inquire the cause of drug withdrawal, presence of the primary outcomes and adverse events in detail. Besides, investigators should follow up on primary outcomes and adverse events and record in the CRF. Investigators should recall the remaining unused study drugs.

Patients discontinuing from study drugs before the last visit should accomplish the 90-day follow up as planned or when recovered from adverse events to normal/stable condition (the later time point should be chosen). Investigators should document all definite withdrawal carefully in the CRF.

7.5.4 Loss to follow-up

Investigators should try their best to keep contact with every patient, making sure the reason of loss to follow-up and their health situation. All CRFs of missed patients should be recorded until the last follow-up visit.

7.6 Quit from study

Those who quit the study should not be included again. The randomization number and study drugs of this subject should not be used again. Randomized patients must not be replaced. Investigators should confirm the withdrawal along with the monitoring committee. Subjects who were randomized and had one or more doses recorded were required to complete the calendar visits according to the protocol.

8. Blood Sample Collection

Blood sample collection refers to serum, plasma and leukocyte (for detailed information, refer to the “collection and management of the biological sample”) that requires to be collected to the corresponding laboratory and transported thereafter with cold-chain to Beijing Tiantan Hospital for centralized storage and analysis. The time of collection should be documented carefully in the CRF.

8.1 Collection time, place and volume

Two blood samples per patient need to be collected at the enrollment hospital, at the time points of "within 24 hours after randomization" and "90±7 days after randomization"; 10ml blood was collected each time, half of which is collected using non-anticoagulant blood collection tube and EDTA anticoagulant blood collection tube respectively.

8.2 Place and manner of sample processing and storage

Blood sample processing is completed at the enrollment hospital, and the Beijing Tiantan Hospital, Capital Medical University provides standardized processing procedures (shown in the biological sample collection and processing manual) and training. The extracted serum, plasma and leukocytes are stored at -20℃~-80℃ at the enrollment hospital after separation, and should be regularly transferred to Beijing Tiantan Hospital, Capital Medical University for centralized storage via cold chain transportation for subsequent testing and analysis.

8.3 Disposal of remaining samples

Surplus samples will be destroyed by Beijing Tiantan Hospital, Capital Medical University.

1) Samples that have been used and wastes should be destroyed uniformly after sterilization.

2) If there is a special need such as repeat tests, samples can be destroyed after appropriate short-term storage.

3) Samples that have not been used 15 years after the end of the study follow-up and wastes should be disinfected and destroyed.

4) Biological samples prohibited by the written request of the sample provider and wastes should be disinfected and then uniformly destroyed.

5) In some other special conditions, such as sample contamination or the discovery of HIV and other viral infected samples, the project leader will be contacted for sample destruction in a timely manner after discovery.

6) All samples destruction need subject leader's knowledge and signature is required

7) The method of sample destruction is to use pressure steam sterilization, sterilization temperature 121℃, sterilization time 20 minutes, after the treatment of the sample into a special medical garbage bag according to the hospital medical waste treatment;

8) All the disposal process of samples should be recorded accordingly, and the operator should fill in the Sample Destruction Record Form.

9. Data Collection

Investigators should finish the CRF promptly as instructed by the protocol and make sure those documents are accurate, complete and without delay. The MRI data of all included patients will be collected in the DICOM form with complete sequence information (sequences T1+T2+FLAIR+DWI/ADC+MRA are required, SWI/GRE-T2* if qualified). Data collected from vascular test, transthoracic echocardiography, 24hrs Holter and lab tests should be uploaded as original photo or in the DICOM form.

10. Study Procedure

All subjects should be screened by investigators with a screening table containing the sub-center number. The screening table will be used to analysis representativeness of subjects included in the study in each sub-center.

10.1 Screening and enrollment

- Patients meeting the inclusion criteria and not the exclusion criteria should sign the informed consent document.
- Confirm the time of the onset of the initial event
- Perform laboratory tests assessment, including the first emergency routine, emergency renal function (serum creatinine, blood urea nitrogen), emergency liver function (serum transaminase) and emergency blood clotting after onset.
- Perform imaging assessment: brain histology assessment, and/or intracranial artery assessment, and/or extracranial artery assessment. Brain histology assessment includes head CT or MRI for diagnosis and differential diagnosis of TIA and cerebral infarction and possible cerebral infarction model evaluation. Intracranial and extracranial artery assessment examination are used for screening. Intracranial artery assessment examination includes any test of MRA、CTA、DSA. Extracranial artery assessment examination includes any test of neck vascular ultrasound, CEMRA, CTA on the aortic arch, DSA;

Note: If a patient is found to be arterial stenosis only by assessing the intracranial arteries and meets the inclusion criteria, he/she can be enrolled without extracranial artery assessment examination. Similarly, if a patient is found to be arterial stenosis only by assessing the extracranial arteries and meets the inclusion criteria, he/she can be enrolled without intracranial artery assessment examination.

(As these radiological tests are recommended as routine tests for ischemic stroke and TIA patients, these examinations will be at patients' own expense)

A thorough explanation of the information of the study will be outlined and shown by the investigators in the written material.

10.2 Day of Randomization

- Record basic demographic information, medical and medication history and physical examination of subjects
- Conduct $ABCD^2$ score (for TIA patients), EQ-5D score and neurological examination (including mRS and NIHSS).
- Apply 12-lead electrocardiogram (ECG) to rule out atrial fibrillation, sick sinus syndrome and second or third degree atrioventricular block.
- Subjects obtain random code, and receive a drug box (for phase 1) in accordance to the

randomization number to supply the study drug used from day 1 to day 21. Study drugs should be taken as soon as the randomization is finished (within 1 hour after randomization). Instructions for taking the first study drug should be given by the investigator. (The first dose includes the total dose of medication taken by the patient after the onset of illness, taken in the ambulance and emergency room, and taken after admission to the hospital).

- Fasting laboratory tests should be completed within 24 hours after randomization (Including routine blood counts, biochemical panel [hepatic function, kidney function, blood lipid, creatine kinase], glycated hemoglobin, homocysteine, coagulation function). Fasting venous blood sample should be collected at the same time, and serum, plasma and white blood cells should be extracted and stored separately (Instructions are listed in the biological sample collection handbook). If not, 72 hours after randomization is the final deadline.
- Complete the initial diagnosis of the initial event for subjects.
- High-resolution MRI of intracranial arteries (high-resolution MRI subgroup only): need to be done within 24 hours of randomization completion.

10.3 Day7 visit

- Record NIHSS score.
- Finish brain histological examinations: including MRT and CT scan. If the brain MRI isn't done before randomization, it should be finished within 7 days after randomization, with complete sequence information (sequences T1+T2+FLAIR+DWI/ADC+MRA are required, SWI/GRE-T2* if qualified). If head MRI is finished, head CT would not be necessary. If the vascular assessment was completed prior to randomization, it doesn't need to be repeated.
- Finish vascular assessments: assessments of intracranial arteries (including MRA, CTA, DSA) and assessments of extracranial arteries (including neck vascular ultrasound, CEMRA, CTA on the arch, DSA), at least one item each is necessary. If the assessment was completed prior to randomization, there is no requirement to review.
- Cardiac examinations: including echocardiography (transthoracic or transesophageal) and electrocardiography (12-lead ECG or long-range HOLTER).
- Finish high-resolution intracranial arterial MRI (only in high-resolution MRI subgroups): examination needs to be completed within 24 hours since randomization.
- Finish laboratory tests: blood routine, biochemistry (including liver function, renal function, blood lipids, creatine kinase) and coagulation function. If the test is completed within 24 hours of screening or randomization, there is no requirement to review.

10.4 Day 14 or Hospital discharge visit

- Record NIHSS score.
- Record the final diagnosis of this event.
- Record concurrent medication since last visit.
- Evaluate subjects' compliance.

- Record new strokes and new vascular events since the last visit.
- Record adverse events, bleeding events since the last visit.
- Make an appointment for drug change on day 21±2 of randomization, recall of remaining study drugs in Box1, dispense study drug Box2 (study drugs from day22±2 to day90±7 after randomization). Study physicians should instruct patients in the second phase of medication administration.
- Make an appointment of day 90 ± 7 visit.

. **10.5 Day 90±7 visit**

- Finish clinical scores (NIHSS, mRS, EQ-5D).
- Finish intracranial arterial high-resolution MRI (only for high resolution MRI subgroups).
- Finish laboratory tests: blood routine and biochemical panel (including liver function, renal function, blood lipids, creatine kinase). Fasting venous blood sample should be collected at the same time, and serum, plasma and white blood cells should be extracted and stored separately (Instructions are listed in the biological sample collection handbook).
- Record concurrent medication since last visit.
- Evaluate the compliance of the subject since the last visit.
- Record new strokes and new vascular events since the last visit.
- Record adverse events, bleeding events since the last visit.
- Retrieve study drug box of phase 2 (Study drug from day 22±2 to day 90).
- Summarize the whole study.
- The subject and the investigator decide which anti-platelet drug should be used after the study together.
- Inform the subject for long-term follow-up, make an appointment of the next visit: 12th month ± 14 days after administration.

10.6 12th month± 14 days visit

- Record clinical scores (mRS, EQ-5D).
- Record personal history of the subject, especially smoking and drinking history.
- Record concurrent medication since last visit.
- Record new strokes and new vascular events since the last visit.
- Record adverse events, bleeding events since the last visit.

10.7 Clinical outcomes assessment

If suspected neurologic symptoms occur (including deterioration of the initial ischemic cerebrovascular disease, new onset transient or persistent neurological symptoms), full data should be submitted within 72 hours by the sub-center and should include:

- Physical examination: vital signs (bilateral brachial artery systolic/diastolic pressure, heart rate, pulse) and neurological examination.
- NIHSS score.

- Head CT or MRI (sequences T1+T2+FLAIR+DWI/ADC+MRA are required, SWI/GRE-T2 if qualified) shall be performed and stored in DICOM form, which should be sent to the radiology department for further assessment; CT is enough for the diagnosis of hemorrhagic stroke while MRI is strongly recommended for ischemic events.
- If suspected cardiovascular events occur, cardiac function should be assessed (including ECG, serum cardiac enzyme and troponin). If myocardial infarction is highly suspected, data should be collected and uploaded to CRO for further assessment within 72 hours since the incident.
- Collect information including the use of study drugs, concurrent medication and adverse events.

10.8 Adverse event handling process

10.8.1 Adverse event/serious adverse event handling principles

- Determine whether the adverse event is related to the study drug: if it is related to the study drug, the investigator should suspend the study drug, let the clinician determine the treatment plan according to the subject's condition, closely observe the subject's condition, and decide whether to resume the study drug according to the subject's recovery. If the adverse event is not related to the study drug, the patient could continue the study drug, and the clinician need determine the treatment plan according to the subject's condition.
- Collect information on which the event was based, such as imaging images and reports, test results, etc.; complete the relevant event form.
- If the event is a serious adverse event, it should be reported in accordance with the serious adverse event handling process.
- If it is a bleeding event, the investigator need to confirm the severity of bleeding according to the GUSTO criteria in the study protocol and fill out the relevant event report form.
- Complete the follow-up visits and record the relevant information.

10.8.2 Reporting of serious adverse events

Investigators needn’t report serious adverse events for the following conditions that occur after the subject's first dose of the study drug through the study follow-up period.

- Life-threatening, permanent or severe disability or loss of function, or hospitalization or prolonged hospitalization resulting from a recurrence of ischemic stroke.
- Life-threatening, permanent or severe disability or loss of function, or hospitalization or prolonged hospitalization resulting from a secondary endpoint event as defined in this study.
- Congenital anomalies or birth defects discovered after enrollment.
- Subjects who are hospitalized or have an extended hospital stay due to transfer to a rehabilitation unit, etc. for long-term post-stroke rehabilitation.

Serious adverse events need to be reported by the investigator if any of the following occur after the subject's first dose of the study drug through the study follow-up period.

- All-cause Death.
- Life-threatening, permanent or severe disability or loss of function, or hospitalization or prolonged hospitalization resulting from a recurrence of hemorrhagic stroke.
- Other unanticipated serious adverse events.

If any of these serious adverse events are reported, the investigator must collect the relevant information, complete a Serious Adverse Event Report Form and report it to the Ethics Committee within 24 hours of being informed.

10.9 Table of study procedure

Measures	Screening and randomization (1st visit)	Treatment period			Observation period
		Day7 (2nd visit)	Day 14 or Hospital discharge (3rd visit)	Day 90±7 (4th visit)	12th month ± 14 days (5th visit)
Inclusion/Exclusion	√				
Informed consent	√				
Onset	√				
First blood pressure and auxiliary examination	√				
Basic information	√				
Past medical history	√				
Personal/family history	√				
Medication history before randomization	√				
Physical examination	√				
ABCD^2 for TIA only	√				
NIHSS	√	√	√	√	
mRS	√			√	√
EQ-5D scale	√			√	√
Imaging assessment for enrollment	√[1]				
Laboratory test	√[2]	√[3]		√[3]	
ECG	√	√			
Randomization	√				
First medication time after randomization	√				
Primary diagnosis	√				
Final diagnosis			√		
Drug dispense/Retrieve	√			√	
Blood specimen (fasting)	√[4]			√[4]	
HR-MRI subgroup	√[5]			√[5]	
Endpoints		√	√	√	√
AEs/SAEs		√	√	√	√
Drug compliance		√	√	√	
Concurrent medication		√	√	√	√

1. Imaging assessments include brain histology and assessments of intracranial and extracranial arteries. Brain histological examination includes head MRI or CT scan for diagnosis of TIA and cerebral infarction, and mode assessment for cerebral infarction (single infarction or multiple infarctions). If MRI cannot be completed before randomization, it must be done within 7 days after randomization. The brain MRI must include the T1+T2+FLAIR+DWI/ADC+MRA sequences. GRE-T2* or SWI shall be done according to the conditions of each sub-center. If head MRI is completed, head CT is not required. Intracranial and extracranial artery assessments are used for screening. Intracranial artery assessment examinations include any test of MRA、CTA、DSA. Extracranial artery assessment examinations include any test of neck vascular ultrasound, CEMRA, CTA on the aortic arch, DSA. **Note**: If a patient applying only one assessment for intracranial or extracranial artery is found to meet the inclusion criteria, he or she can be included without performing both intracranial and extracranial artery assessment. For example, if a TIA patient has only finished a neck vascular ultrasound, and finds out the responsibility artery stenosis ≥50% and is in accord with the standard set of circumstances, the patient can be recruited without assessment of intracranial arteries. **All the above assessments need to be completed within 72 hours after randomization and needs no repetition if they are completed before randomization. The cervical vascular ultrasound should be uploaded in original photo, other image data should be uploaded to Beijing Tiantan hospital with DICOM format.**
2. Emergency laboratory assessment should be finished at screening, including emergency blood routine, emergency liver function (serum transaminases), emergency renal function (creatinine, urea nitrogen) and emergency blood clotting. The fasting laboratory tests (including biochemical kits, glycosylated hemoglobin, homocysteine, etc.) should be completed in the early morning of the second day after randomization. If not, 72 hours after randomization is the final deadline.
3. Routine blood counts, biochemical panel (including alanine transaminase, aspartate aminotransferase, alkaline phosphatase, creatine kinase, creatine kinase-MB) and coagulation function should be performed on day7 (If the test had been completed within 24 hours of screening or randomization, there was no requirement to review it).Routine blood counts and biochemical panel (including alanine transaminase, aspartate aminotransferase, alkaline phosphatase, creatine kinase, creatine kinase-MB, low-density lipoprotein cholesterol, high-density lipoprotein cholesterol, total cholesterol, triglyceride) should be performed on day 90±7 after randomization to monitor hepatotoxicity and muscle toxicity of statin.
4. Fasting venous blood should be collected within 24 hours of randomization (If not, 72 hours after randomization is the final deadline) and 90±7 days after randomization.
5. High-resolution MRI examinations should be performed for patient enrolled in the subgroup of high-resolution MRI, within 24 hours and 90 days ± 7 hours after randomization.

11. Ethics and Regulations

11.1 Ethical principles

This clinical study will follow the rules of the Declaration of Helsinki. This clinical Study will be conducted in accordance with the principles laid down by the 18th World Medical Assembly, the ICH guidelines for Good Clinical Practice. This protocol has been reviewed by an independent Ethics Committee before being conducted. The investigators should fully inform the subjects of all pertinent aspects, including the aim, procedures and risk of this study. A written information is required giving approval opinion. All patients should be informed to the fullest extent possible about the study, and that they have the right to withdraw from this study. The signed and dated written Informed Consent should be well-kept for further investigation. Personal privacy and data confidentiality should be protected during this study.

11.2 Laws and regulations

This clinical study will be conducted in compliance with all international laws and regulations, and national laws and regulations of the countries in which the clinical study is performed, as well as any applicable guidelines.

11.3 Informed consent procedures

The investigator should fully inform the subjects of all pertinent aspects of the clinical study permitted by the Ethics Committee. All subjects should be informed to the fullest extent possible about the study, in a language and terms they are able to understand. If the subject can’t express his own will, the above introduction and explanation should be provided to legal representative of the subject.

Prior to a subject’s participation in the Clinical Trial, a written Informed Consent Form should be signed, name filled in and personally dated by the patient or by the patient’s legally acceptable representative, and by the person who conducted the informed consent discussion. If the subjects or legal representative can't read, an impartial witness must be present throughout the process of witness. A copy of the signed and dated written Informed Consent Form will be provided to the patient.

Approval and favorable opinion from the Sponsor is required before the informed consent being submitted to the Ethics Committee.

11.4 Institutional Review Board/Independent Ethics Committee (IRB/IEC)

Before the trial, the Investigator or the Sponsor must submit this Clinical Trial Protocol to the appropriate Ethics Committee (IRB/IEC), and is required to forward to the Sponsor a copy of the written and dated approval/favorable opinion signed by the Chairman with Ethics Committee (IRB/IEC) composition.

The Clinical Trial (study number, Clinical Trial Protocol title and version number), the documents reviewed (Clinical Trial Protocol, Informed Consent Form, Investigator’s Brochure, Investigator’s CV, etc.), the list of voting members and their qualifications and the date of the review should be stated on

the written (IRB/IEC) approval/favorable opinion.

Investigational Product must not be released to sub-centers prior to the start of the trial. The Clinical Trial must not begin until a copy of the written and dated approval/favorable opinion is received by the Sponsor.

During the Clinical Trial, any amendment or modification to the Clinical Trial Protocol should be submitted to the Ethics Committee (IRB/IEC) , and execute after being approved. Any event likely to affect the safety of patients or the continuation of the Clinical Trial, particularly any change in safety outcomes, should also be reported to IRB/IEC. All updates to the Investigator's Brochure will be sent to the Ethics Committee (IRB/IEC).

If requested, a progress report and a summary of the Clinical Trial's outcome at the end of the Clinical Trial shall be sent to the Ethics Committee (IRB/IEC) annually.

12. Research Organization

12.1 Research organization

- **Main investigator**

Prof. Yilong Wang，Department of Neurology, Beijing Tiantan Hospital, Capital Medical University.

Prof. Yongjun Wang，Department of Neurology, Beijing Tiantan Hospital, Capital Medical University.

- **Steering Committee**
  - Steering Committee will provide scientific and strategic recommendations, and will be responsible for the design and conduct of the study and publication of the main study results.
  - Steering Committee will maintain contact with investigators to ensure high quality data collection, conduct and management.
  - Steering Committee will approve the main protocol and guidelines before conducting the study
  - Steering Committee will discuss and report progression of the study in telephone conference or face-to-face conference periodically.
  - The member of the Steering Committee and their duties will be described and confirmed before the study being conducted.
- **Executive Committee**

Executive Committee will evaluate the progression and blinded-data, and give appropriate advice to the study. Main decisions should be made in face-to-face conferences of the Executive Committee. The member of the Executive Committee and their duties will be described and confirmed before the study being conducted.

- **Data and Safety Monitoring Board (DSMB)**

To meet the study's highest ethical responsibility and safety to its subjects, a data safety monitoring board (DSMB) will monitor results during the study periodically. The board includes academic members such as biostatisticians, and they have no formal involvement or conflict of interest with the subjects, the investigators or the clinical sites. The members of the DSMB and their duties will be described and confirmed before the study being conducted. A report should be submitted to the chairman of the committee immediately after the DSMB conference.

- **Event Adjudication Committee**

Clinical Endpoint events (stroke, MI, death, major bleeding) and safety outcomes will be reviewed by independent specialists (in neurology and cardiology). The member of the Event Adjudication Committee (member, qualification, duties) should be qualified by Steering and Executive Committee.

The radiology information of the clinical event should be first assessed locally and meanwhile the original picture should be submitted to the main investigator. The adjudication report should include radiology report.

12.2 Qualifications and Training

The Executive Committee should make sure every clinical center receives GCP training, as well as screening, follow-up and outcome evaluation training (such as NIHSS, mRS). The main investigator and coordinator of each sub-center should finish the training and be certified before the study as required. ALL investigators should receive training and be certified

- Study Procedure
- Guidelines for diagnosis of ischemic stroke and TIKA
- $ABCD^2$ score
- Inclusion and Exclusion Criteria
- mRS scoring
- NIHSS scoring
- TOAST classification
- Primary adjudication of the outcomes event
- Definition of GUSTO bleeding criteria
- EQ-5D scale
- Collection and procession of biologic specimen (blood)
- Collection of radiology information in DICOM form

Investigators and coordinators in each clinical site must be trained and qualified before conducting the study. PI of the study center and the main investigator should attend a periodically telephone conference to solve the problem meted in the study. Qualification shall be certificated by training center.

Detailed manuals will be used as the main references for the investigators. It serves as the instruction of the training, and will be updated on the "INSPIRES" website throughout the entire research period. Executive Committee and coordinators will keep contact with investigators through telephone, e-mail and fax, to help them solve the problem. The INSPIRES Executive and Monitoring Committee will answer the problems each sub-center meets and deliver it to all sub-centers together.

The Executive Committee will inspect all clinical centers in the field and make sure the accuracy and integrity of the data. Every clinical center shall be inspected at least one time during the study and shall be inspected more if the quality of data is poor or there is problem in subject inclusion.

13. Quality Control and Quality Assurance

13.1 Responsibilities of investigators

The Investigator(s) undertake(s) to perform the Clinical Trial in accordance with this Clinical Trial Protocol, ICH guidelines for Good Clinical Practice and the applicable regulatory requirements.

The Investigator is required to ensure compliance with all procedures required by the Clinical Trial Protocol and with all study procedures provided by the Sponsor (including security rules). The Investigator agrees to provide reliable data and all information requested by the Clinical Trial Protocol (with the help of the Case Report Form [CRF], Discrepancy Resolution Form [DRF] or other appropriate instrument) in an accurate and legible manner according to the instructions provided and to ensure direct access to source documents by Sponsor representatives.

The Investigator may appoint such other individuals as he/she may deem appropriate as Sub-Investigators to assist in the conduct of the Clinical Trial in accordance with the Clinical Trial Protocol. All Sub-Investigators shall be appointed and listed in a timely manner. The Sub-Investigators will be supervised by and under the responsibility of the Investigator. The Investigator will provide them with a copy of the Clinical Trial Protocol and all necessary information. The sponsors shall be responsible for the health authority, taking any possible methods to ensure the appropriate management of the clinical study, these methods including Ethics, compliance of the study plan, the integrity and validity of the clinical data.

13.2 Study Monitoring

The main responsibility of the Monitoring Committee is to assist the investigators and sponsors in the all aspects of the clinical study in a highly ethics, scientific, professional and normalization manner. According to the ICH guidelines for Good Clinical Practice, the Monitoring Team must check the Case Report Form entries against the source documents.

At regular intervals during the Clinical Trial, the site will be contacted, through monitoring visits, letters or telephone calls, by a representative of the Monitoring Team to review study progress, investigator and patient compliance with Clinical Trial Protocol requirements and any emergent problems. During these monitoring visits, the following but not exhaustive list of points will be scrutinized with the investigator: patient informed consent, patient recruitment and follow-up, Serious Adverse Event documentation and reporting, Investigational Product allocation, patient compliance with the Clinical Trial Protocol and the Investigational Product regimen, concomitant therapy use and quality of data.

14. Data Preservation

All CRFs and imagine data should be delivered to data management center by the inspector and should be checked and received by the directors of the data storage center. All CRFs which should be kept in this center should be completed entirely in a neat, legible manner to ensure accurate interpretation of data. After the data entry was completed, the CRFS should be saved by the research center.

15. Safety Monitoring of Data

Corresponding data security monitoring plan will be developed based on the risk of the Clinical Trial. All adverse events will be recorded in detail, properly handled and tracked until events are resolved properly or the condition becomes stable. Serious adverse events and other events should be reported to the ethics committee, competent authorities, sponsors, and drug supervision and management departments according to applicable regulations. The main investigators will regularly review all adverse events. Researchers meeting will be held to assess the risks and benefits of the study if necessary. For studies that exceeds the minimum risk, independent data monitor will be assigned to monitor the research data. An independent data safety inspection committee will be established for high-risk research to monitor the accumulated safety data and effectiveness, making recommendations on whether to proceed with the proposal or not.

During the clinical trial, the subject's data should be collected anonymously on the CRFs. Subjects can only be identified as the subject number and the “pinyin” abbreviation of name. Due to security or administrative instructions, those who accidentally knows the identity of the subject should be responsible for confidentiality along with the investigators. Informed Consent Forms allow authorized sponsors, ethical committees, and authoritative agencies to directly access relevant original data on CRFs (such as patient medical records, appointment records, original laboratory records, etc.). The above personnel should follow the professional secrecy regulations and must keep confidential data of patients’ personally identifiable information or medical information.

16. Statistical Processing

Main null hypothesis

1) In patients with acute mild ischemic stroke or high-risk TIA patients attributed to extracranial or intracranial atherosclerosis treated within 72 hours of ictus, there is no difference in the risk of a new stroke within 90 days between subjects with intensive antiplatelet therapy and those with standard antiplatelet therapy.
2) In patients with acute mild ischemic stroke or high-risk TIA patients attributed to extracranial or intracranial atherosclerosis treated within 72 hours of ictus, there is no difference in the risk of a new stroke within 90 days between subjects with immediate intensive statin therapy (atorvastatin 80mg/d) and delayed intensive statin therapy (atorvastatin 40mg/d).
3) In patients with acute mild ischemic stroke or high-risk TIA patients attributed to extracranial or intracranial atherosclerosis treated within 72 hours of ictus, there is no difference in the risk of a new stroke within 90 days between subjects with intensive antiplatelet combined with immediate intensive statin therapy (atorvastatin 80mg/d) and standard antiplatelet combined with delayed intensive statin therapy (atorvastatin 40mg/d).

16.1 Estimate Sample Size

The minimal sample size for the trial is determined by the necessity that a clinically meaningful difference in effectiveness between treatment and control groups has to be detected. Based on previous studies, the risk of new stroke during 90 days is presumed to be 11.5% in the group with standard antiplatelet therapy (with half delayed intensive statin therapy and half early high-intensity statin therapy) and 11.5% in the delayed intensive statin therapy group (with half standard antiplatelet therapy and half dual antiplatelet therapy) and 13% in the group with standard antiplatelet therapy plus delayed intensive statin therapy, intensive antiplatelet therapy and immediate intensive statin therapy can reduce this risk by 22%, and the effects of intensive antiplatelet and lipid lowering therapy will be similar and additive. With a sample size of 6100 subjects, a two-sided α of 0.05 and 5% loss to follow up, we will have 97% power to detect that the risk is decreased by 35% in the group with dual antiplatelet therapy plus immediate intensive statin therapy compared to standard antiplatelet therapy plus delayed intensive therapy, and 80% power to detect that the risk is reduced by 20% by intensive antiplatelet therapy compared to standard antiplatelet therapy, and immediate intensive statin compared to delayed intensive statin therapy, respectively.

Table1 For different risk rates (12% to 14%) of the results in the control group, the sample size required for observing intensive antiplatelet combining intensive statin therapy

Power	A single set of sample size (no expulsion)	A single set of sample size (5% expulsion rate)	Four groups of sample size	The stroke recurrence rate in treatment group	The stroke recurrence rate in control group	Relative risk
0.966	1448	1525	6100	0.078	0.120	0.65

0.975	1448	1525	6100	0.085	0.130	0.65
0.985	1448	1525	6100	0.091	0.140	0.65
0.824	1448	1525	6100	0.096	0.130	0.74

Table2 For different risk rates (12% to 14%) of the results in the control group, the sample size required for the marginal effect analysis of observed intensive antiplatelet combining intensive statin therapy

**Power**	**A single set of sample size (no expulsion)**	**A single set of sample size (5% expulsion rate)**	**Four groups of sample size**	**The stroke recurrence rate in treatment group**	**The stroke recurrence rate in control group**	**Relative risk**
0.807	2896	3050	6100	0.0930	0.1157	0.80

Explanations of data sources on recurrence risk and the risk of stroke recurrence after treatment:

At present, evidence in early risks of recurrent stroke in high-risk symptomatic intracranial or extracranial artery stenosis patients is still lacking. The data currently available for reference include: a) In the studies of WASID and SAMMPRIS, the recurrence risk of stroke in the medication treatment group with symptomatic intracranial artery stenosis (stenosis rate > 70%) were 21.9% and 12.6% at 1 year, respectively. [52,53] The recurrence risk of 12.6% at 1 year in SAMMPRIS research was based on the combination of dual anti-platelet therapy, intensive lipid-lowering and blood-pressure-lowering therapy. Therefore, for the recurrence risk of the standard therapy group, 13% was a conservative figure in our study. [54] b) The subgroup study of the CHANCE Trial has revealed a recurrence risk of 12.5% in patients with intracranial arterial stenosis (stenosis rate > 50%) at day 90.[4] In the CHANCE trial, patients with intracranial arterial stenosis associated with multiple infarcts had a recurrent stroke risk of a striking number of 18% at 90 days.[21] Therefore, as the recurrence risk of the standard therapy group, 13% was a conservative data in our study.

Concerning that dual anti-platelet therapy reduces the risk of recurrence of 22%, the CHANCE study confirmed that dual anti-platelet therapy decreased the risk of stroke recurrence by 90 days for TIA and minor stroke by 32% compared with anti-platelet monotherapy. [15] In the CHANCE subgroup, compared with anti-platelet monotherapy, dual anti-platelet therapy decreased the relative risk of stroke recurrence by 21% in patients with intracranial arterial stenosis, and by 50% in patients with multiple cerebral infarctions. [4,22] Therefore, with dual anti-platelet therapy, our estimate of a relative reduction of 22% in recurrent stroke risk was a conservative data in our study.

Concerning the source of evidence for intensive lipid-lowering combined with intensive antiplatelet therapy decreases the risk of stroke by 35%, a comparative study of SAMMPRIS and WASID before and after pointed out that the risks of recurrent stroke were 21.9% and 12.6% in the intensive drug therapy group and anti-platelet monotherapy group at 1 year, respectively. The risk was decreased by about 50%.

Therefore, the estimated reduction of recurrence risk by 35% was a conservative data in our study. [53]

16.2 Data collection and record

All requirements of the test program in the CRF table must be provided, and the parts not provided should be explained.

Electronic Data Capture (EDC) System will be used to input the data in CRF. All test protocol requirements in the CRF table must be provided, and the part not provided should be explained. The administrator will check the data and correct the obvious errors. Other errors or missing data in the project will be returned to the research center. The research center will verify the original information and relevant information to answer and correct the data query form. The process of queries, responses and corrections will be completed and recorded through the EDC system.

As for paper-based information such as incident report forms, researchers must fill in the data requested by the test program in black or blue pen in carbonless copies of the case report forms to ensure that the copy is clear and readable. If the information needs to be modified, it should not be altered or overwritten. The correct information should be written beside the original information and signed and dated by the modifier. The inspector will make sure the accuracy and completeness of the case report forms, and guide the researchers to make necessary corrections and supplementary questions.

After receiving the data, the sponsor will carry out computer data analysis, additional requirements may arise, and researchers must answer these requirements by confirming or modifying data. The content of the corrections will be added to the EDC through the investigator.

16.3 statistical analysis

This section is an overview of the statistical analysis. It provides general provisions on how to collect data and display data in clinical studies. The final version of the statistical analysis plan (SAP) will be completed before the database is locked. SAP will specify all "pre-defined, planned analyzes".

16.3.1 Statistical analysis of data sets

Full Analysis Set (FAS): The full analysis set is the main efficacy evaluation population of this study, and all valid variables will be analyzed by FAS. According to the basic principle of intention-to-treat(ITT) analysis, all subjects randomized into groups and those with more than one medication record and efficacy evaluation will be included in the full analysis set. When choosing FAS to analyze, the estimate of the missing value, the last observation carried forward (LOCF) estimation method can be used.

Per Protocol Set (PPS): Includes all subjects who complete the protocol or have no serious breaches of the trial. The exact definition of a serious violation of the program will be finalized at the time of data review and may generally include the following situations (but not limited to these cases): failing to meet the main inclusion criteria, serious disturbance of drug efficacy after treatment, poor compliance, exceeding the time window of following-up and so on. PPS is the secondary analysis of the effectiveness of the crowd, but if the results are inconsistent with the whole analysis set, detailed analysis of the

inconsistent results are needed.

Safety Data Set (SS): A safety data set is defined as a subject that receives drug treatment at least once. In the entire safety analysis, patients with incorrect treatment (for example, randomized to the standard antiplatelet combining delayed intensive statin therapy groups were given enhanced antiplatelet therapy) would be assigned to the actual treatment group.

16.3.2 Statistical analysis methods

16.3.2.1 The balance analysis of basic values (comparison between groups of baseline indicators)

1) The measurement data will be using t test or Wilcoxon rank sum test;

2) Enumeration data between groups will be compared with chi-square test, Fisher's exact probability method or Wilcoxon rank sum test, when appropriate.

16.3.2.2 Efficacy analysis

1) Primary outcome:

Survival curves will be estimated for the primary outcome using the Kaplan-Meier procedure and compared using a Cox regression model Wald test, stratified by the opposite arm of the factorial design. First, marginal effects will be compared between intensive and standard antiplatelet therapy, and also between immediate intensive statin therapy (atorvastatin 80mg/d) and delayed intensive statin therapy (atorvastatin 40mg/d). As these are independent comparisons, they have no effect on the type I error rate. For both treatment comparisons, the primary outcome of stroke will be judged to be statistically significant if the treatment test p-value is < 0.05. Second, if either intensive antiplatelet or immediate intensive statin treatment (atorvastatin 80mg/d) is statistically significant, the effects between combined intensive antiplatelet and immediate intensive statin treatment (atorvastatin 80mg/d) and combined standard antiplatelet and delayed intensive statin treatment (atorvastatin 40mg/d) will be tested. Cox regression model stratified by the opposite arm of the factorial design, will be used in the curative effect assessment and evaluation between the subgroups.

2) Secondary outcomes:

d. Kaplan-Meier curve will be used to simulate the cumulative risk of 90-day follow-up combined with secondary endpoint events such as vascular events, ischemic stroke, TIA, myocardial infarction, vascular death and all-cause death. Cox proportional risk model will be used to calculate HR and the 95% confidence interval and the Log-rank test would be used to evaluate the efficacy;

e. For ordinal stroke or TIA, ordinal logistic regression will be used to calculate OR and 95% confidence interval. For the poor functional outcome (mRS score 2-6) and early neurological deficits (increase in NIHSS score at 7days) indexes, logistic regression will be used to calculate OR and 95% confidence interval.

f. The validity of the extreme value would be examined, and the sensitivity analysis would be carried out.

3) Safety analysis

The overall of safety assessment is all used test medications and the safety follow-up case should be

recorded at least once. The safety evaluation data includes adverse reactions observed during the trial and changes in laboratory data before and after treatment.

Safety evaluation will be analyzed using safety data set.

Moderate to severe bleeding, intracranial hemorrhage, and overall mortality will be calculated using the Kaplan-Meier curve to simulate the 3-month cumulative risk, and the Cox proportional hazards model to calculate the HR and 95% confidence interval.

For other adverse events and laboratory abnormalities, the cases which was normal before the treatment and abnormal after the treatment would be mainly analyzed and listed, in order to the comparison of differences before and after treatment.

17. Confidentiality and Announcement of Results

The main investigators possess complete intellectual property rights. Information of the subjects are strictly protected through the research process and data analysis process. The Executive Committee will publish the results in accordance with the regulations and procedures. After the database is locked, the test results will be published as soon as possible. With 6100 patients, this study will investigate detailed data of the treatments, medical measures and clinical outcomes. The biostatistics experts participating in the study have the right to access the data set but are unable to identify any patient in this trial. Finally, the comma-separated text format of the disk storage data (including the text format of the data dictionary) will be sent to the intention of the third party to deal with.

REFERENCE:

1. Lovett JK, Coull AJ, Rothwell PM. Early risk of recurrence by subtype of ischemic stroke in population-based incidence studies. *Neurology*. 2004;62:569-573. doi: 10.1212/01.wnl.0000110311.09970.83
2. Petty GW, Brown RD, Jr., Whisnant JP, Sicks JD, O'Fallon WM, Wiebers DO. Ischemic stroke subtypes : a population-based study of functional outcome, survival, and recurrence. *Stroke*. 2000;31:1062-1068. doi: 10.1161/01.str.31.5.1062
3. Amarenco P, Lavallee PC, Labreuche J, Albers GW, Bornstein NM, Canhao P, Caplan LR, Donnan GA, Ferro JM, Hennerici MG, et al. One-Year Risk of Stroke after Transient Ischemic Attack or Minor Stroke. *The New England journal of medicine*. 2016;374:1533-1542. doi: 10.1056/NEJMoa1412981
4. Liu L, Wong KS, Leng X, Pu Y, Wang Y, Jing J, Zou X, Pan Y, Wang A, Meng X, et al. Dual antiplatelet therapy in stroke and ICAS: Subgroup analysis of CHANCE. *Neurology*. 2015;85:1154-1162. doi: 10.1212/wnl.0000000000001972
5. Ois A, Cuadrado-Godia E, Rodriguez-Campello A, Giralt-Steinhauer E, Jimenez-Conde J, Lopez-Cuina M, Ley M, Soriano C, Roquer J. Relevance of stroke subtype in vascular risk prediction. *Neurology*. 2013;81:575-580. doi: 10.1212/WNL.0b013e31829e6f37
6. Liu X, Xu G, Wu W, Zhang R, Yin Q, Zhu W. Subtypes and one-year survival of first-ever stroke in Chinese patients: The Nanjing Stroke Registry. *Cerebrovascular diseases (Basel, Switzerland)*. 2006;22:130-136. doi: 10.1159/000093241
7. Kasner SE, Chimowitz MI, Lynn MJ, Howlett-Smith H, Stern BJ, Hertzberg VS, Frankel MR, Levine SR, Chaturvedi S, Benesch CG, et al. Predictors of ischemic stroke in the territory of a symptomatic intracranial arterial stenosis. *Circulation*. 2006;113:555-563. doi: 10.1161/circulationaha.105.578229
8. Wang Y, Zhao X, Liu L, Soo YO, Pu Y, Pan Y, Wang Y, Zou X, Leung TW, Cai Y, et al. Prevalence and outcomes of symptomatic intracranial large artery stenoses and occlusions in China: the Chinese Intracranial Atherosclerosis (CICAS) Study. *Stroke*. 2014;45:663-669. doi: 10.1161/strokeaha.113.003508
9. Kang DW, Latour LL, Chalela JA, Dambrosia J, Warach S. Early ischemic lesion recurrence within a week after acute ischemic stroke. *Ann Neurol*. 2003;54:66-74. doi: 10.1002/ana.10592
10. Wen HM, Lam WW, Rainer T, Fan YH, Leung TW, Chan YL, Wong KS. Multiple acute cerebral infarcts on diffusion-weighted imaging and risk of recurrent stroke. *Neurology*. 2004;63:1317-1319. doi: 10.1212/01.wnl.0000140490.22251.b6
11. Kang DW, Chu K, Ko SB, Kwon SJ, Yoon BW, Roh JK. Lesion patterns and mechanism of ischemia in internal carotid artery disease: a diffusion-weighted imaging study. *Arch Neurol*. 2002;59:1577-1582. doi: 10.1001/archneur.59.10.1577

12. Lee DK, Kim JS, Kwon SU, Yoo SH, Kang DW. Lesion patterns and stroke mechanism in atherosclerotic middle cerebral artery disease: early diffusion-weighted imaging study. *Stroke*. 2005;36:2583-2588. doi: 10.1161/01.Str.0000189999.19948.14
13. Wong KS, Gao S, Chan YL, Hansberg T, Lam WW, Droste DW, Kay R, Ringelstein EB. Mechanisms of acute cerebral infarctions in patients with middle cerebral artery stenosis: a diffusion-weighted imaging and microemboli monitoring study. *Ann Neurol*. 2002;52:74-81. doi: 10.1002/ana.10250
14. Kernan WN, Ovbiagele B, Black HR, Bravata DM, Chimowitz MI, Ezekowitz MD, Fang MC, Fisher M, Furie KL, Heck DV, et al. Guidelines for the prevention of stroke in patients with stroke and transient ischemic attack: a guideline for healthcare professionals from the American Heart Association/American Stroke Association. *Stroke*. 2014;45:2160-2236. doi: 10.1161/str.0000000000000024
15. Wang Y, Wang Y, Zhao X, Liu L, Wang D, Wang C, Wang C, Li H, Meng X, Cui L, et al. Clopidogrel with aspirin in acute minor stroke or transient ischemic attack. *The New England journal of medicine*. 2013;369:11-19. doi: 10.1056/NEJMoa1215340
16. Wong KS, Wang Y, Leng X, Mao C, Tang J, Bath PM, Markus HS, Gorelick PB, Liu L, Lin W, et al. Early dual versus mono antiplatelet therapy for acute non-cardioembolic ischemic stroke or transient ischemic attack: an updated systematic review and meta-analysis. *Circulation*. 2013;128:1656-1666. doi: 10.1161/circulationaha.113.003187
17. Hong KS, Lee SH, Kim EG, Cho KH, Chang DI, Rha JH, Bae HJ, Lee KB, Kim DE, Park JM, et al. Recurrent Ischemic Lesions After Acute Atherothrombotic Stroke: Clopidogrel Plus Aspirin Versus Aspirin Alone. *Stroke*. 2016;47:2323-2330. doi: 10.1161/strokeaha.115.012293
18. Markus HS, Droste DW, Kaps M, Larrue V, Lees KR, Siebler M, Ringelstein EB. Dual antiplatelet therapy with clopidogrel and aspirin in symptomatic carotid stenosis evaluated using doppler embolic signal detection: the Clopidogrel and Aspirin for Reduction of Emboli in Symptomatic Carotid Stenosis (CARESS) trial. *Circulation*. 2005;111:2233-2240. doi: 10.1161/01.Cir.0000163561.90680.1c
19. Wong KS, Chen C, Fu J, Chang HM, Suwanwela NC, Huang YN, Han Z, Tan KS, Ratanakorn D, Chollate P, et al. Clopidogrel plus aspirin versus aspirin alone for reducing embolisation in patients with acute symptomatic cerebral or carotid artery stenosis (CLAIR study): a randomized, open-label, blinded-endpoint trial. *Lancet Neurol*. 2010;9:489-497. doi: 10.1016/s1474-4422(10)70060-0
20. Wang X, Lin WH, Zhao YD, Chen XY, Leung TW, Chen C, Fu J, Markus H, Hao Q, Wong KS. The effectiveness of dual antiplatelet treatment in acute ischemic stroke patients with intracranial arterial stenosis: a subgroup analysis of CLAIR study. *Int J Stroke*. 2013;8:663-668. doi: 10.1111/j.1747-4949.2012.00828.x
21. Pan Y, Meng X, Jing J, Li H, Zhao X, Liu L, Wang D, Johnston SC, Wang Y, Wang Y. Association of multiple infarctions and ICAS with outcomes of minor stroke and TIA. *Neurology*. 2017;88:1081-1088. doi: 10.1212/wnl.0000000000003719

22. Jing J, Meng X, Zhao X, Liu L, Wang A, Pan Y, Li H, Wang D, Johnston SC, Wang Y, et al. Dual Antiplatelet Therapy in Transient Ischemic Attack and Minor Stroke With Different Infarction Patterns: Subgroup Analysis of CHANCE Randomized Clinical Trial. *JAMA Neurol*. 2018. doi: 10.1001/jamaneurol.2018.0247
23. Johnston SC, Amarenco P, Albers GW, Denison H, Easton JD, Evans SR, Held P, Jonasson J, Minematsu K, Molina CA, et al. Ticagrelor versus Aspirin in Acute Stroke or Transient Ischemic Attack. *The New England journal of medicine*. 2016;375:35-43. doi: 10.1056/NEJMoa1603060
24. Amarenco P, Albers GW, Denison H, Easton JD, Evans SR, Held P, Hill MD, Jonasson J, Kasner SE, Ladenvall P, et al. Efficacy and safety of ticagrelor versus aspirin in acute stroke or transient ischaemic attack of atherosclerotic origin: a subgroup analysis of SOCRATES, a randomized, double-blind, controlled trial. *Lancet Neurol*. 2017;16:301-310. doi: 10.1016/s1474-4422(17)30038-8
25. Amarenco P, Bogousslavsky J, Callahan A, 3rd, Goldstein LB, Hennerici M, Rudolph AE, Sillesen H, Simunovic L, Szarek M, Welch KM, et al. High-dose atorvastatin after stroke or transient ischemic attack. *The New England journal of medicine*. 2006;355:549-559. doi: 10.1056/NEJMoa061894
26. Sillesen H, Amarenco P, Hennerici MG, Callahan A, Goldstein LB, Zivin J, Messig M, Welch KM. Atorvastatin reduces the risk of cardiovascular events in patients with carotid atherosclerosis: a secondary analysis of the Stroke Prevention by Aggressive Reduction in Cholesterol Levels (SPARCL) trial. *Stroke*. 2008;39:3297-3302. doi: 10.1161/strokeaha.108.516450
27. Amarenco P, Benavente O, Goldstein LB, Callahan A, 3rd, Sillesen H, Hennerici MG, Gilbert S, Rudolph AE, Simunovic L, Zivin JA, et al. Results of the Stroke Prevention by Aggressive Reduction in Cholesterol Levels (SPARCL) trial by stroke subtypes. *Stroke*. 2009;40:1405-1409. doi: 10.1161/strokeaha.108.534107
28. Underhill HR, Yuan C, Zhao XQ, Kraiss LW, Parker DL, Saam T, Chu B, Takaya N, Liu F, Polissar NL, et al. Effect of rosuvastatin therapy on carotid plaque morphology and composition in moderately hypercholesterolemic patients: a high-resolution magnetic resonance imaging trial. *American heart journal*. 2008;155:584.e581-588. doi: 10.1016/j.ahj.2007.11.018
29. Du R, Cai J, Zhao XQ, Wang QJ, Liu DQ, Leng WX, Gao P, Wu HM, Ma L, Ye P. Early decrease in carotid plaque lipid content as assessed by magnetic resonance imaging during treatment of rosuvastatin. *BMC cardiovascular disorders*. 2014;14:83. doi: 10.1186/1471-2261-14-83
30. Lee JM, Robson MD, Yu LM, Shirodaria CC, Cunnington C, Kylintireas I, Digby JE, Bannister T, Handa A, Wiesmann F, et al. Effects of high-dose modified-release nicotinic acid on atherosclerosis and vascular function: a randomized, placebo-controlled, magnetic resonance imaging study. *J Am Coll Cardiol*. 2009;54:1787-1794. doi: 10.1016/j.jacc.2009.06.036
31. Corti R, Fayad ZA, Fuster V, Worthley SG, Helft G, Chesebro J, Mercuri M, Badimon JJ. Effects of lipid-lowering by simvastatin on human atherosclerotic lesions: a longitudinal study by high-resolution, noninvasive magnetic resonance imaging. *Circulation*. 2001;104:249-252.

32. Corti R, Fuster V, Fayad ZA, Worthley SG, Helft G, Smith D, Weinberger J, Wentzel J, Mizsei G, Mercuri M, et al. Lipid lowering by simvastatin induces regression of human atherosclerotic lesions: two years' follow-up by high-resolution noninvasive magnetic resonance imaging. *Circulation*. 2002;106:2884-2887. doi: 10.1161/01.cir.0000041255.88750.f0
33. Sibley CT, Vavere AL, Gottlieb I, Cox C, Matheson M, Spooner A, Godoy G, Fernandes V, Wasserman BA, Bluemke DA, et al. MRI-measured regression of carotid atherosclerosis induced by statins with and without niacin in a randomized controlled trial: the NIA plaque study. *Heart*. 2013;99:1675-1680. doi: 10.1136/heartjnl-2013-303926
34. Oesterle A, Laufs U, Liao JK. Pleiotropic Effects of Statins on the Cardiovascular System. *Circulation research*. 2017;120:229-243. doi: 10.1161/circresaha.116.308537
35. Amin-Hanjani S, Stagliano NE, Yamada M, Huang PL, Liao JK, Moskowitz MA. Mevastatin, an HMG-CoA reductase inhibitor, reduces stroke damage and upregulates endothelial nitric oxide synthase in mice. *Stroke*. 2001;32:980-986. doi: 10.1161/01.str.32.4.980
36. Chen J, Zhang ZG, Li Y, Wang Y, Wang L, Jiang H, Zhang C, Lu M, Katakowski M, Feldkamp CS, et al. Statins induce angiogenesis, neurogenesis, and synaptogenesis after stroke. *Ann Neurol*. 2003;53:743-751. doi: 10.1002/ana.10555
37. D NC, Callaly EL, Duggan J, Merwick Á, Hannon N, Sheehan Ó, Marnane M, Horgan G, Williams EB, Harris D, et al. Association between acute statin therapy, survival, and improved functional outcome after ischemic stroke: the North Dublin Population Stroke Study. *Stroke*. 2011;42:1021-1029. doi: 10.1161/strokeaha.110.596734
38. Blanco M, Nombela F, Castellanos M, Rodriguez-Yáñez M, García-Gil M, Leira R, Lizasoain I, Serena J, Vivancos J, Moro MA, et al. Statin treatment withdrawal in ischemic stroke: a controlled randomized study. *Neurology*. 2007;69:904-910. doi: 10.1212/01.wnl.0000269789.09277.47
39. Hong KS, Lee JS. Statins in Acute Ischemic Stroke: A Systematic Review. *J Stroke*. 2015;17:282-301. doi: 10.5853/jos.2015.17.3.282
40. Tsivgoulis G, Katsanos AH, Sharma VK, Krogias C, Mikulik R, Vadikolias K, Mijajlovic M, Safouris A, Zompola C, Faissner S, et al. Statin pretreatment is associated with better outcomes in large artery atherosclerotic stroke. *Neurology*. 2016;86:1103-1111. doi: 10.1212/wnl.0000000000002493
41. Heo JH, Song D, Nam HS, Kim EY, Kim YD, Lee KY, Lee KJ, Yoo J, Kim YN, Lee BC, et al. Effect and Safety of Rosuvastatin in Acute Ischemic Stroke. *J Stroke*. 2016;18:87-95. doi: 10.5853/jos.2015.01578
42. Montaner J, Bustamante A, García-Matas S, Martínez-Zabaleta M, Jiménez C, de la Torre J, Rubio FR, Segura T, Masjuán J, Cánovas D, et al. Combination of Thrombolysis and Statins in Acute Stroke Is Safe: Results of the STARS Randomized Trial (Stroke Treatment With Acute Reperfusion and Simvastatin). *Stroke*. 2016;47:2870-2873. doi: 10.1161/strokeaha.116.014600
43. Yoshimura S, Uchida K, Daimon T, Takashima R, Kimura K, Morimoto T. Randomized Controlled Trial of Early Versus Delayed Statin Therapy in Patients With Acute Ischemic Stroke: ASSORT Trial

(Administration of Statin on Acute Ischemic Stroke Patient). *Stroke*. 2017;48:3057-3063. doi: 10.1161/strokeaha.117.017623

44. Johnston SC, Easton JD, Farrant M, Barsan W, Conwit RA, Elm JJ, Kim AS, Lindblad AS, Palesch YY. Clopidogrel and Aspirin in Acute Ischemic Stroke and High-Risk TIA. *New England Journal of Medicine*. 2018. doi: 10.1056/NEJMoa1800410
45. Pan Y, Elm JJ, Li H, Easton JD, Wang Y, Farrant M, Meng X, Kim AS, Zhao X, Meurer WJ, et al. Outcomes Associated With Clopidogrel-Aspirin Use in Minor Stroke or Transient Ischemic Attack: A Pooled Analysis of Clopidogrel in High-Risk Patients With Acute Non-Disabling Cerebrovascular Events (CHANCE) and Platelet-Oriented Inhibition in New TIA and Minor Ischemic Stroke (POINT) Trials. *JAMA Neurology*. 2019;76:1466-1473. doi: 10.1001/jamaneurol.2019.2531
46. Powers WJ, Rabinstein AA, Ackerson T, Adeoye OM, Bambakidis NC, Becker K, Biller J, Brown M, Demaerschalk BM, Hoh B, et al. Guidelines for the Early Management of Patients With Acute Ischemic Stroke: 2019 Update to the 2018 Guidelines for the Early Management of Acute Ischemic Stroke: A Guideline for Healthcare Professionals From the American Heart Association/American Stroke Association. *Stroke*. 2019;50:e344-e418. doi: 10.1161/str.0000000000000211
47. Grundy SM, Stone NJ, Bailey AL, Beam C, Birtcher KK, Blumenthal RS, Braun LT, de Ferranti S, Faiella-Tommasino J, Forman DE, et al. 2018 AHA/ACC/AACVPR/AAPA/ABC/ACPM/ADA/AGS/APhA/ASPC/NLA/PCNA Guideline on the Management of Blood Cholesterol: A Report of the American College of Cardiology/American Heart Association Task Force on Clinical Practice Guidelines. *Circulation*. 2019;139:e1082-e1143. doi: 10.1161/cir.0000000000000625
48. HPS2-THRIVE randomized placebo-controlled trial in 25 673 high-risk patients of ER niacin/laropiprant: trial design, pre-specified muscle and liver outcomes, and reasons for stopping study treatment. *Eur Heart J*. 2013;34:1279-1291. doi: 10.1093/eurheartj/eht055
49. Chimowitz MI, Lynn MJ, Howlett-Smith H, Stern BJ, Hertzberg VS, Frankel MR, Levine SR, Chaturvedi S, Kasner SE, Benesch CG, et al. Comparison of warfarin and aspirin for symptomatic intracranial arterial stenosis. *The New England journal of medicine*. 2005;352:1305-1316. doi: 10.1056/NEJMoa043033
50. Wong KS, Chen C, Ng PW, Tsoi TH, Li HL, Fong WC, Yeung J, Wong CK, Yip KK, Gao H, et al. Low-molecular-weight heparin compared with aspirin for the treatment of acute ischaemic stroke in Asian patients with large artery occlusive disease: a randomized study. *Lancet Neurol*. 2007;6:407-413. doi: 10.1016/s1474-4422(07)70079-0
51. Barnett HJM, Taylor DW, Haynes RB, Sackett DL, Peerless SJ, Ferguson GG, Fox AJ, Rankin RN, Hachinski VC, Wiebers DO, et al. Beneficial effect of carotid endarterectomy in symptomatic patients with high-grade carotid stenosis. *The New England journal of medicine*. 1991;325:445-453.

52. Chimowitz MI, Lynn MJ, Derdeyn CP, Turan TN, Fiorella D, Lane BF, Janis LS, Lutsep HL, Barnwell SL, Waters MF, et al. Stenting versus aggressive medical therapy for intracranial arterial stenosis. *The New England journal of medicine*. 2011;365. doi: 10.1056/NEJMoa1105335
53. Chaturvedi S, Turan TN, Lynn MJ, Derdeyn CP, Fiorella D, Janis LS, Chimowitz MI. Do Patient Characteristics Explain the Differences in Outcome Between Medically Treated Patients in SAMMPRIS and WASID? *Stroke*. 2015;46:2562-2567. doi: 10.1161/strokeaha.115.009656
54. Waters MF, Hoh BL, Lynn MJ, Kwon HM, Turan TN, Derdeyn CP, Fiorella D, Khanna A, Sheehan TO, Lane BF, et al. Factors Associated With Recurrent Ischemic Stroke in the Medical Group of the SAMMPRIS Trial. *JAMA Neurol*. 2016;73:308-315. doi: 10.1001/jamaneurol.2015.4315

Appendix 1. The Definition of Cardiac-cerebral Vascular Events

Event	Definition
**Stroke**	A sudden onset of focal or global brain, spinal cord or retinal vascular damage, resulting in symptoms and signs of acute nervous system defects, which is associated with cerebral circulation disorders.
**Ischemic Stroke**	Acute focal cerebral or retinal infarction meeting any of the following conditions:<br>(1) Clinical signs or radiological evidence of acute onset of new focal neurological damage lasting longer than 24 hours, excluding other non-ischemic etiologies (such as brain infections, brain injuries, brain tumors, seizures, severe metabolic diseases, degenerative diseases of the nervous system and side effects of drugs);<br>(2) Acute cerebral or retinal ischemic events, excluding other non-ischemic etiologies, focal symptoms or signs sustaining less than 24 hours, but with radiological evidence of new infarction;<br>(3) The worsening of pre-existing symptoms of vascular origin ischemic stroke (i.e. NIHSS increased ≥ 4 based on primary ischemic stroke, excluding the hemorrhagic transformation after infarction or symptomatic intracranial hemorrhage) persisting for more than 24 hours, with or without deterioration of ischemic lesions on MRI or CT. Etiologic typing is based on the TOAST criteria.
**Transient Ischemic Attack**	Neurologic deficit caused by sudden focal brain or retinal ischemia that can fully recover, lasting less than 24 hours, with no evidence of new cerebral infarction on imaging (CT or MR). Other non-ischemic causes (such as brain infections, brain injuries, brain tumors, epilepsy, severe metabolic diseases, or degenerative neurological diseases) are excluded.
**Hemorrhagic Stroke**	Hemorrhagic stroke is defined as acute neurological dysfunction of the focal or whole brain or spinal cord caused by non-traumatic brain parenchymal, intraventricular, and subarachnoid hemorrhage.
**Hemorrhagic Transformation after Cerebral Infraction**	Any non-traumatic extravascular hemorrhage in acute / subacute infarcts, which could cause related neurological symptoms (symptomatic) or non-neurological symptoms (asymptomatic). Among them:<br>(1) Ischemic stroke transformed into symptomatic hemorrhagic stroke: The following two conditions must be met at the same time:<br>a. Imaging evidence (CT or MRI) of extravascular hemorrhage in the infarct area;<br>b. Symptoms are related to hemorrhagic transformation. The hemorrhagic transformation must be able to partially explain the clinical manifestations of the patient's neurological performance, such as:<br>iv). Symptoms cannot be fully explained by infarct size and location<br>v). Clinical deterioration referring to an increase of 4 points or more in NIHSS score after the initial ischemic event, or death, which is caused by hemorrhagic transformation;<br>vi). Clinical symptoms caused by volume effect secondary to hemorrhagic transformation;<br>(2) Ischemic stroke transformed into asymptomatic hemorrhagic stroke: The following two conditions must be met at the same time:<br>a. Imaging evidence (CT or MRI) of extravascular hemorrhage in the infarct area;<br>b. Hemorrhagic transformation does not cause symptoms, or cause symptoms with an increase of less than 4 points in NIHSS score after the initial ischemic event.
**Myocardial Infraction**	Acute myocardial infarction diagnosed by the third universal definition (Thygesen, 2012)<br>If there is clinical evidence of myocardial necrosis consistent with acute myocardial ischemia (MI), acute MI should be diagnosed. It can be diagnosed if it meets any of the following criteria:<br>(1) A rise and/ or fall of cardiac biomarkers (preferably troponin [cTn]) values with at least one value above the 99th percentile URL, and any of the followings is required:<br>a. Clinical symptoms of myocardial ischemia;<br>b. New myocardial ischemic changes in the ECG, including new ST-segment changes or left bundle branch block (LBBB) [According to whether there is ST-segment elevation in the ECG, it is classified as acute ST-segment elevation myocardial infarction (STEMI) and non-ST segment elevation myocardial infarction (NSTEMI)];<br>c. Pathological Q wave detected in ECG;<br>d. Imaging demonstration of new loss of viable myocardium or new regional wall motion abnormality;

	e. Coronary thrombosis confirmed by angiography or autopsy.<br>(2) Cardiac death with symptoms suggestive of myocardial ischaemia and presumed new ischaemic ECG changes or new LBBB, but death occurring before cardiac biomarkers could be obtained, before cardiac biomarker could rise, or in rare cases cardiac biomarkers were not collected.<br>(3) Myocardial infarction related to percutaneous coronary intervention (PCI) is arbitrarily defined by elevation of cTn values >5×99th percentile URL in patients with normal baseline values (≤99th percentile URL) or a rise of cTn values >20% if the baseline values are elevated and are stable or falling. In addition, any of the followings is required:<br>a. Symptoms suggestive of myocardial ischemia;<br>b. New ischemic ECG changes or new LBBB;<br>c. Angiographic loss of patency of a major coronary artery or a side branch or persistent slow- or no-flow or embolization;<br>d. Imaging demonstration of new loss of viable myocardium or new regional wall motion abnormality.<br>(4) Myocardial infarction related to stent thrombosis is detected by coronary angiography or autopsy in the setting of myocardial ischemia and with a rise and/ or fall of cardiac biomarkers values with at least one value above the 99th percentile URL.<br>(5) Myocardial infarction related to coronary artery bypass grafting (CABG) is arbitrarily defined by elevation of cardiac biomarker values＞10×99th percentile URL in patients with normal baseline cTn values (≤99th percentile URL). In addition, any of the followings is required:<br>a. new pathological Q waves or new LBBB;<br>b. angiographic documented new graft or new native coronary artery occlusion;<br>c. imaging evidence of new loss of viable myocardium or new regional wall motion abnormality
**Vascular Death**	Vascular death includes sudden cardiac death, death due to stroke, acute myocardial infarction, heart failure, pulmonary embolism, cardiac/cerebrovascular intervention or surgery (unrelated to acute MI) and other cardiovascular causes [e.g. arrhythmia irrelevant with sudden cardiac death, aortic aneurysm rupture, or peripheral artery disease].<br>Any death of unknown/unclear cause within 30 d after stroke, myocardial infarction, or cardio-cerebrovascular operation/surgery will be regarded as death due to stroke, myocardial infarction, or cardio-cerebrovascular operation/surgery, respectively.

Appendix 2. Evaluation Method of Modified Rankin Scale

The modified Rankin scale is used to measure functional recovery after stroke. Boldface shows the formal definition of each level. Italics provide further guidance to reduce the possible errors among observers, but the form of interview is not restricted. Please take in account only the symptoms that occur after stroke. If the patient does not require outside help, if he is able to walk with the aid of certain assistive devices, then he is considered to be able to walk independently.

If the two level seems equally applicable to the patient and further questions are not likely to make the absolute right choice, the more severe one should be selected.

0- No symptoms

Although there may be mild symptoms, the patient is not aware of any new functional limitations or symptoms after the stroke.

1- No significant disability. Able to carry out all usual activities, despite some symptoms.

Some patients have symptoms caused by stroke, whether physical or cognitive (such as influencing speech, reading and writing; or body movement or feeling; or vision; or swallowing; or emotion), but they are still able to continue to engage in all previous work, social and leisure activities. The key question used to distinguish between grades 1 and 2 (see below) can be, "is there anything you often did, but you can't do it after the stroke?". Activities with frequencies exceeding once a month are considered "often".

2- Slight disability; Able to look after own affairs without assistance, but unable to carry out all previous activities.

Patients are able to finish activities (such as driving, dancing, reading, or work) before stroke and can no longer able to finish after stroke, but they still can take care of themselves every day without the help of others. Patients can dress, walk, dine, go to the bathroom, prepare simple food, shop, travel locally, etc. Their life require no supervision. It is assumed that patients at this level can live alone for a week or more without care.

3- Moderate disability; Requires some help, but able to walk unassisted

At this level, patients can walk independently (assisted by walking machinery), dress independently, go to the bathroom, dine and so on, but more complex tasks need to be done with the help of others. For example, they need someone else to do the shopping, cooking cleaning, and visiting them more than once a week to ensure the completion of these activities. What needs assistance is not only to take care of the body, but also to give advice: for example, patients at this level will need supervision or encouragement to deal with finances.

4- Moderately severe disability; Unable to attend to own bodily needs without assistance, and unable to walk unassisted. Patients require others to help with their daily lives in walking, dressing, going to the bathroom or eating. Patients need to be taken care of at least once a day, usually twice or more times, or must live very close to caregivers. To differentiate between grades 4

and 5 (see below), consider whether they can routinely live alone for a proper amount of time during the day.<br>**5- Severe disability; Requires constant nursing care and attention, bedridden, incontinent.** Although no trained nurses are neccessary, they need to be taken care of throughout the day and night.

Appendix 3. NIHSS Score

Administer stroke scale items in the order listed. Record performance in each category after each subscale exam. Do not go back and change scores. Follow directions provided for each exam technique. Scores should reflect what the patient does, not what the clinician thinks the patient can do. The clinician should record answers while administering the exam and work quickly. Except where indicated, the patient should not be coached (ie, repeated requests to patient to make a special effort).

	Examination	Grade	Score
1a	Level of consciousness:<br>The investigator must choose a response if a full evaluation is prevented by such obstacles as an endotracheal tube, language barrier, orotracheal trauma/bandages. A 3 is scored only if the patient makes no movement (other than reflexive posturing) in response to noxious stimulation.	0 = **Alert;** keenly responsive.<br>1 = **Not alert;** but arousable by minor stimulation to obey, answer, or respond.<br>2 = **Not alert;** requires repeated stimulation to attend, or is obtunded and requires strong or painful stimulation to make movements (not stereotyped).<br>3 = Responds only with reflex motor or autonomic effects or totally unresponsive, flaccid, and areflexic.	
1b	**1b. LOC questions:**The patient is asked the month and his/her age. The answer must be correct - there is no partial credit for being close. Aphasic and stuporous patients who do not comprehend the questions will score 2. Patients unable to speak because of endotracheal intubation, orotracheal trauma, severe dysarthria from any cause, language barrier, or any other problem not secondary to aphasia are given a 1. It is important that only the initial answer be graded and that the examiner not "help" the patient with verbal or non-verbal cues.	0 = **Answers** both questions correctly.<br>1 = **Answers** one question correctly.<br>2 = **Answers** neither question correctly.	
1c	**LOC commands:**The patient is asked to open and close the eyes and then to grip and release the non-paretic hand. Substitute another one step command if the hands cannot be used. Credit is given if an unequivocal attempt is made but not completed due to weakness. If the patient does not respond to command, the task should be demonstrated to him or her (pantomime), and the result scored (ie, follows none, one or two commands). Patients with trauma, amputation, or other physical impediments should be given suitable one-step commands. Only the first attempt is scored.	0 = **Performs** both tasks correctly.<br>1 = **Performs** one task correctly.<br>2 = **Performs** neither task correctly.	
2	**Best gaze:** Only horizontal eye movements will be tested. Voluntary or reflexive (oculocephalic) eye movements will be scored, but caloric testing is not done. If the patient has a conjugate deviation of the eyes that can be overcome by voluntary or reflexive activity, the score will be 1. If a patient has an isolated peripheral nerve paresis (CN III, IV or VI), score a 1. Gaze is testable in all aphasic patients. Patients with ocular trauma, bandages, pre-existing blindness, or other disorder of visual acuity or fields should be tested with reflexive movements,	0 = **Normal.**<br>1 = **Partial gaze palsy;**gaze is abnormal in one or both eyes, but forced deviation or total gaze paresis is not present.<br>2 = **Forced deviation,** or total gaze paresis not overcome by the oculocephalic maneuver.	

	and a choice made by the investigator. Establishing eye contact and then moving about the patient from side to side will occasionally clarify the presence of a partial gaze palsy.		
3	**Visual:** Visual fields (upper and lower quadrants) are tested by confrontation, using finger counting or visual threat, as appropriate. Patients may be encouraged, but if they look at the side of the moving fingers appropriately, this can be scored as normal. If there is unilateral blindness or enucleation, visual fields in the remaining eye are scored. Score 1 only if a clear-cut asymmetry, including quadrantanopia, is found. If patient is blind from any cause, score 3. Double simultaneous stimulation is performed at this point. If there is extinction, patient receives a 1, and the results are used to respond to item 11.	0 = No vision loss<br>1 = Partial hemianopia<br>2 = Complete hemianopia<br>3 = Bilateral Blindness, (blind, including cortical blindness)	
4	**Facial palsy:** Ask - or use pantomime to encourage - the patient to show teeth or raise eyebrows and close eyes. Score symmetry of grimace in response to noxious stimuli in the poorly responsive or non-comprehending patient. If facial trauma/bandages, orotracheal tube, tape or other physical barriers obscure the face, these should be removed to the extent possible.	0 = **Normal** symmetrical movements.<br>1 = **Minor paralysis**(flattened nasolabial fold, asymmetry on smiling).<br>2 = **Partial paralysis** (total or near-total paralysis of lower face).<br>3 = **Complete paralysis** of one or both sides (absence of facial movement in the upper and lower face).	
5	**Motor arm:** The limb is placed in the appropriate position: extend the arms (palms down) 90 degrees (if sitting) or 45 degrees (if supine). Drift is scored if the arm falls before 10 seconds. The aphasic patient is encouraged using urgency in the voice and pantomime, but not noxious stimulation. Each limb is tested in turn, beginning with the non-paretic arm. Only in the case of amputation or joint fusion at the shoulder, the examiner should record the score as 9, and clearly write the explanation for this choice.	0 = **No drift;** limb holds 90 (or 45) degrees for full 10 seconds.<br>1 = **Drift;** limb holds 90 (or 45) degrees, but drifts down before full 10 seconds; does not hit bed or other support.<br>2 = **Some effort against gravity;** limb cannot get to or maintain (if cued) 90 (or 45) degrees, drifts down to bed, but has some effort against gravity.<br>3 = **No effort against gravity;** limb falls.<br>4 = **No movement.**<br>9 = **Amputation** or joint fusion, explain:________________	5a left arm _____<br>5B right arm _____
6	**Motor leg:** The limb is placed in the appropriate position: hold the leg at 30 degrees (always tested supine). Drift is scored if the leg falls before 5 seconds. The aphasic patient is encouraged using urgency in the voice and pantomime, but not noxious stimulation. Each limb is tested in turn, beginning with the non-paretic leg. Only in the case of amputation or joint fusion at the hip, the examiner should record the score as 9, and clearly write the explanation for this choice.	0 = **No drift;** leg holds 30-degree position for full 5 seconds.<br>1 = **Drift;** leg falls by the end of the 5-second period but does not hit bed.<br>2 = **Some effort against gravity;** leg falls to bed by 5 seconds, but has some effort against gravity.	6a left leg _____<br>6a right leg _____

		3 = **No effort against gravity;** leg falls to bed immediately.<br>4 = **No movement.**<br>9 = **Amputation** or joint fusion, explain:________________	
7	**Limb ataxia:** This item is aimed at finding evidence of a unilateral cerebellar lesion. Test with eyes open. In case of visual defect, ensure testing is done in intact visual field. The finger-nose-finger and heel-shin tests are performed on both sides, and ataxia is scored only if present out of proportion to weakness. Ataxia is absent in the patient who cannot understand or is paralyzed. Only in the case of amputation or joint fusion, the examiner should record the score as 9, and clearly write the explanation for this choice. In case of blindness, test by having the patient touch nose from extended arm position.	0 = **Absent.**<br>1 = **Present in one limb.**<br>2 = **Present in two limbs.**<br>9 = **Amputation** or joint fusion, explain:________________	
8	**Sensory:** Sensation or grimace to pinprick when tested, or withdrawal from noxious stimulus in the obtunded or aphasic patient. Only sensory loss attributed to stroke is scored as abnormal and the examiner should test as many body areas (arms [not hands], legs, trunk, face) as needed to accurately check for hemisensory loss. A score of 2, "severe or total sensory loss," should only be given when a severe or total loss of sensation can be clearly demonstrated. Stuporous and aphasic patients will, therefore, probably score 1 or 0. The patient with brainstem stroke who has bilateral loss of sensation is scored 2. If the patient does not respond and is quadriplegic, score 2. Patients in a coma (item 1a=3) are automatically given a 2 on this item.	0 = Normal, without sensory loss<br>0 = **Normal;** no sensory loss.<br>1 = **Mild-to-moderate sensory loss;** patient feels pinprick is less sharp or is dull on the affected side; or there is a loss of superficial pain with pinprick, but patient is aware of being touched.<br>2 = **Severe to total sensory loss;** patient is not aware of being touched in the face, arm, and leg.	
9	**Best language:** A great deal of information about comprehension will be obtained during the preceding sections of the examination. For this scale item, the patient is asked to describe what is happening in the attached picture, to name the items on the attached naming sheet and to read from the attached list of sentences. Comprehension is judged from responses here, as well as to all of the commands in the preceding general neurological exam. If visual loss interferes with the tests, ask the patient to identify objects placed in the hand, repeat, and produce speech. The intubated patient should be asked to write. The patient in a coma (item 1a=3) will automatically score 3 on this item. The examiner must choose a score for the patient with stupor or limited cooperation, but a score of 3 should be used only if the patient is mute and follows no one-step commands.	0 = **No aphasia;** normal.<br>1 = **Mild-to-moderate aphasia;** some obvious loss of fluency or facility of comprehension, without significant limitation on ideas expressed or form of expression. Reduction of speech and/or comprehension, however, makes conversation about provided materials difficult or impossible. For example, in conversation about provided materials, examiner can identify picture or naming card content from patient's response.<br>2 = **Severe aphasia;** all communication is through fragmentary expression; great need for inference, questioning, and guessing by the listener. Range of information that can be exchanged is limited; listener carries burden of	

		communication. Examiner cannot identify materials provided from patient response.<br>3 = **Mute, global aphasia;** no usable speech or auditory comprehension.	
10	**Dysarthria:** If patient is thought to be normal, an adequate sample of speech must be obtained by asking patient to read or repeat words from the attached list. If the patient has severe aphasia, the clarity of articulation of spontaneous speech can be rated. Only if the patient is intubated or has other physical barriers to producing speech, the examiner should record the score as 9, and clearly write an explanation for this choice. Do not tell the patient why he or she is being tested.	0 = **Normal.**<br>1 = **Mild-to-moderate dysarthria;** patient slurs at least some words and, at worst, can be understood with some difficulty.<br>2 = **Severe dysarthria;** patient's speech is so slurred as to be unintelligible in the absence of or out of proportion to any dysphasia, or is mute/anarthric.<br>9 = **Intubated** or other physical barrier, explain:________________	
11	**Extinction and inattention (formerly neglect):**Sufficient information to identify neglect may be obtained during the prior testing. If the patient has a severe visual loss preventing visual double simultaneous stimulation, and the cutaneous stimuli are normal, the score is normal. If the patient has aphasia but does appear to attend to both sides, the score is normal. The presence of visual spatial neglect or anosognosia may also be taken as evidence of abnormality. Since the abnormality is scored only if present, the item is never untestable.	0 = **No abnormality.**<br>1 = **Visual, tactile, auditory, spatial, or personal inattention** or extinction to bilateral simultaneous stimulation in one of the sensory modalities.<br>2 = **Profound hemi-inattention or extinction to more than one modality;** does not recognize own hand or orients to only one side of space.	
Total			

Attachment:

1. 9th, 10th inspection's chart

Reading inspection chart 1 Reading inspection chart 2

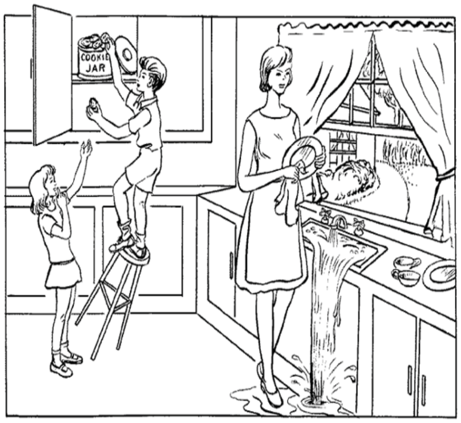

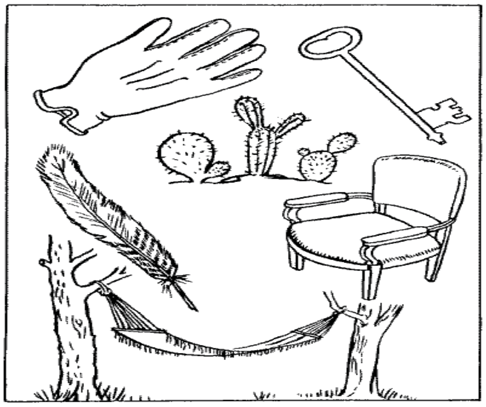

Reading inspection chart 3

Reading inspection chart 4

Please read the following sentences:

know

Walk down the stairs

Home cooking

Review at school

Deliver a wonderful speech

Please read the following words

Mom

earth

Aircraft

silk

Start on time

Eat grapes without spitting grapes

2 How NIHSS scores are rated for special conditions?

➢ How is the NIHSS score assessed in uncooperative patients?

If a patient is uncooperative on an item, document it clearly. All untested items are to be reviewed by the medical inspector and discussed with the examiner if necessary.

➢ How is the NIHSS score assessed in coma patients?

If patients scored 3 in this factor, the default coma scores should be used when applicable.

1a = 3 if the patient makes only reflexive posturing movements to repeated painful stimuli, or, they are totally unresponsive. If 1a = 3, other items should be evaluated as:

- 1b- level of consciousness questions: score 2
- 1c- level of consciousness commands: score 2
- 2- best gaze: see if the patient's best gaze can be balanced by oculoucephalogyric reflex. If balanced, score 1; if not, score 2.
- 3- visual: use visual threat to evaluate.
- 4- facial palsy: score 3
- 5 and 6- motor arm and motor leg: score 4 for each limb
- 7- limb ataxia: score only when there exists ataxia. If the patient fails due to muscle weakness, score 0.
- 8- sensory: score 2
- 9- best language: score 3
- 10- dysarthria: score 2
- 11- extinction and inattention: score 2 as coma means losing all cognitive abilities

➢ How is the NIHSS score assessed in coma patients?

- 1b - Consciousness level questioning: 2 points for aphasics who cannot understand the question. Scoring can only be based on the initial response. This item can never be scored as "unable to check".
- 1c - Level of Consciousness Instructions: If the patient does not respond to instructions, the examiner demonstrates (gestures) and then scores based on the outcome (e.g., compliance with 0, 1, or 2 instructions). The patient is then scored based on the outcome (e.g., compliance with 0, 1, or 2 commands). Only the first attempt can be scored and the question can be asked only once.
- 2 - Optimal Gaze: For all aphasic individuals, gaze is checked. In aphasic patients, it is helpful to establish eye contact and walk around the bed. This item is an exception to the principle of observing the first response and the inability to train. If the patient is unable to actively gaze, head-eye movements, eye gaze and tracking the examiner's methods can be used to provide a stronger examination stimulus.
- 3-Field of vision: not limited by aphasia.
- 4-Facial palsy: Patients with aphasia are scored based on symmetry of expression during injurious

stimulation.

- 5 - Upper limb movements: for aphasic patients guided by voice or gesture without injurious stimulation. If you measure the non-hemiplegic side of the limb first, the aphasic patient may understand what you are trying to measure.
- 6 - Lower extremity movements: Use voice or gesture guidance for the aphasic person, without injurious stimuli. If you measure the non-hemiplegic limb first, the aphasic person may understand what you are trying to measure.
- 7 - Limb ataxia: If the patient cannot understand, score 0. If the limb is initially moved passively by the examinee, the aphasic patient often completes the examination correctly. The aphasic patient often completes the examination correctly if the limb is initially moved by the examiner.
- 8 - Sensory: examines the aphasic person's avoidance of injurious stimuli. A score of 2 can only be given if a severe or complete sensory deficit is clearly established. given. Therefore, the aphasic person may also be scored 1 or 0.
- 9 - Best Language: Ask the patient to look at pictures and talk, name objects on cards, and read sentences from the utterance list. Slight aphasia is scored as 1 point. Use all the materials provided Use all materials provided to decide whether to choose 1 or 2 points. It is estimated that the patient missed more than 2/3 of the named objects and sentences or performed very few and simple one-step instructions. If the patient missed more than 2/3 of the named objects and sentences or performed very few and simple one-step instructions, a score of 2 was assigned.
- 10 - Dysarthria: Patients with aphasia are scored based on their spontaneous speech and by having them repeat the words you read aloud. If there is severe aphasia, the Scores are based on the clarity of articulation in spontaneous speech.
- 11 - Sensory fade and loss of attention (formerly neglect): If aphasic, but do notice bilaterally, score normal. Because abnormalities are recorded only when the performance is abnormal, this item must be scored as normal. abnormality is recorded only when there is abnormal performance, so this item must be measurable

3 How to calculate the total score of NIHSS?

When calculating total score, the following items should not be included in the total score:

- Item 5, 6 in limb movements “9= amputation or fusion of joints"
- Item 7 of ataxia “9=Amputation or joint fusion, explain:”
- Item 10 of dysarthria “ Intubated or other physical barrier, explain:__”

Appendix 4. ABCD^2 Score

Items		Options	Score
A	Age	≥ 60 years	1
		< 60years	0
B	First Blood Pressure after TIA	SBP≥ 140mmHg or DBP≥ 90mmHg	1
		SBP< 140mmHg and DBP< 90mmHg	0
C	Clinical Symptom	Unilateral motor weakness	2
		Speech disturbance (either dysarthria or dysphasia or both) without weakness	1
		Other symptoms	0
D	Duration of TIA symptoms	≥ 60 minutes	2
		10-59 minutes	1
		< 10minutes	0
D	Diabetes	with	1
		without	0

Appendix5. EQ-5D Scale

Which of the followings best describes your state of health today?

Mobility

○ I have no problems in walking about

○ I have some problems in walking about

○ I am confined to bed

Self-care

○ I have no problems with self-care

○ I have some problems washing or dressing myself

○ I am unable to wash or dress myself

Usual activities (e.g. work, study, housework, family or leisure activities)

○ I have no problems with performing my usual activities

○ I have some problems with performing my usual activities

○ I am unable to perform my usual activities

Pain / discomfort

○ I have no pain or discomfort

○ I have moderate pain or discomfort

○ I have extreme painful or uncomfortable

Anxiety / depression

○ I am not anxious or depressed

○ I am moderately anxious or depressed

○ I am extremely anxious or depressed

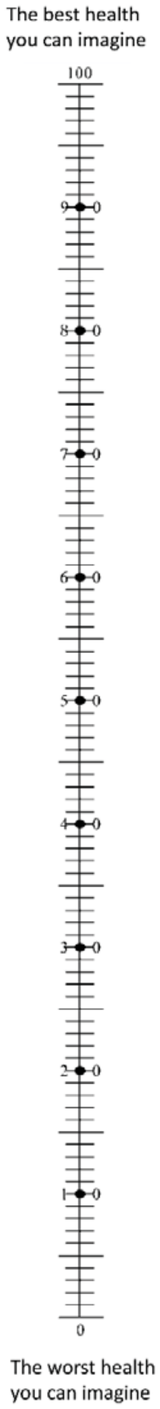

We would like to know how good or bad your health is TODAY.

This scale is numbered from 0 to 100.

100 means the best health you can imagine. 0 means the worst health you can imagine.

Mark an X on the scale to indicate how your health is TODAY.

Now, please write the number you marked on the scale in the box below.

How good or bad your own health is today?

How is your health today?

The questionnaire was completed by:

○1 patients themselves

○2 patients with the help of the third

○3 agents (family members of patients)

Appendix 6. GUSTO Bleeding Criteria

If the patient's bleeding events are consistent with multiple classifications, it should be classified as the most severe one.

1. Severe bleeding

Fatal or intracranial hemorrhage or bleeding resulting in haemodynamic changes requiring blood or fluid transfusions, cardiac stress therapy, ventricular assist devices, surgery, or cardiopulmonary resuscitation.

2. Moderate bleeding

Bleeding requiring transfusion therapy but not resulting in hemodynamic changes that require intervention.

3. Minor bleeding

Bleeding that does not require transfusion or fluid therapy and does not result in hemodynamic changes. This includes subcutaneous bleeding, small hematomas or bruises at the puncture site.

During the study, all transfusion of blood products will be recorded on the case report form, including infusion units and capacity.

1, Hb reference range 130 to 180 g/L (male); 120 to 160 g/L (female)

2, Consider the impact of blood transfusion, Hb measurements must adjust the infusion of PRBC or whole blood between two hematological tests. Suppose 1 units of blood transfusion cause Hb to increase by 10 g/L. Thus, when the blood transfusion is performed between the two blood tests, the actual values of Hb can be calculated by using the following formula: **Hb = [baseline Hb -Hb after blood transfusion]+ [infusion unit x Hb conversion coefficient *]**

*Hb conversion factor = 10 g/L

Appendix 7. Clinical Crucial Laboratory Abnormalities

Bleeding tendency	Prothrombin time more than 1.5 times or platelet count < 10 x 10^9 / L
Medium and severe anemia	Hemoglobin (Hb) < 90 g/L
Abnormal liver function	More than 2 times higher than normal transaminase
Abnormal renal function	Serum creatinine > 1.5 mg/dl or creatinine clearance < 50 ml/min

Protocol changes version 2.0 (Apr. 28th, 2018) to version 3.0 (Feb 28th, 2019)

Protocol V2.0	Protocol V3.0
**Summary-Follow-up Plan**<br>Face to face interviews will be performed at baseline, Day7±2 days, Day14 (or hospital discharge), Day 21±2, and Day90 ± 7 days, and telephone interviews will be performed at the 6th month±14 days and 12th month±14 days after randomization.	**Summary-Follow-up Plan**<br>Face to face interviews will be performed at baseline, Day7±2 days, Day14 (or hospital discharge) and Day90 ± 7, and telephone interviews will be performed at the 12th month±14 days after randomization.
**10.4 Day 14 or Hospital discharge visit**<br>• Make an appointment of day 21±2 visit.	**10.4 Day 14 or Hospital discharge visit**<br>• Make an appointment for drug change on day 21±2 of randomization, recall of remaining study drugs in Box1, dispense study drug Box2 (study drugs from day22±2 to day90±7 after randomization). Study physicians should instruct patients in the second phase of medication administration.<br>• Make an appointment of day 90 ± 7 visit.
**10.5 Day 21±2 visit**<br>• Record concurrent medication since last follow-up.<br>• Evaluate subjects' compliance.<br>• Record new strokes and new vascular events since the last follow-up.<br>• Record adverse events, bleeding events since the last follow-up.<br>• Recall of remaining study drugs in Box1, dispense study drug Box2 (study drugs from day22±2 to day90±7 after randomization). Study physicians should instruct patients in the second phase of medication administration.<br>• Make an appointment of day90 ± 7 visit.	**Delete**
**10.6 Day 90±7 visit**<br>• Inform the subject for long-term follow-up, make an appointment of the next visit: the 6th month ± 14 days after administration.	**10.5 Day 90±7 visit**<br>• Inform the subject for long-term follow-up, make an appointment of the next visit: the 12th month ± 14 days after administration.
**10.7 6th month± 14 days visit**	**Delete**

• Record personal history of the subject, especially smoking and drinking history.<br>• Record concurrent medication since last follow-up.<br>• Record new strokes and new vascular events since the last follow-up.<br>• Record adverse events, bleeding events since the last follow-up.<br>• Make an appointment of the 12th month ± 14 days visit.	

Protocol changes version 3.0 (Feb 28th, 2019) to version 4.0 (Jun 6th, 2020)

Protocol V3.0	Protocol V4.0
**Inclusion Criteria:**<br>2. Less than 72 hours from the onset;<br>3. At least one of the followings (a-c)<br>a) High-risk TIA (ABCD2 ≥ 4) with ≥50% stenosis of a major intracranial or extracranial artery that likely accounts for clinical presentation.<br>b) Acute single cerebral infarction (NIHSS≤5) with ≥50% stenosis of a major intracranial or extracranial artery that likely accounts for the infarction and clinical presentation.<br>c) Acute multiple cerebral infarction (NIHSS≤5) with stenosis of extracranial or intracranial arteries (stenosis degree unlimited) that likely accounts for the presentation.	**Inclusion Criteria:**<br>2.At least one of the following (a-b):<br>a) Mild ischemic stroke (NIHSS score 4-5) within 24 hours after onset and either of the following imaging characteristics:<br>1) Acute single infarction with ≥50% stenosis of a major intracranial or extracranial artery that likely accounts for the infarction and clinical presentation.<br>2) Acute multiple infarctions documented by head CT or MRI, attributed to large-artery atherosclerosis, including non-stenotic vulnerable plaques.<br>b) Mild ischemic stroke (NIHSS score ≤5) or high-risk of TIA (ABCD2 score≥4) within 24-72 hours after onset and meet any of the following imaging characteristics:<br>1) TIA with ≥50% stenosis of a major intracranial or extracranial artery that likely accounts for the clinical presentation;<br>2) Acute single infarction with ≥50% stenosis of a major intracranial or extracranial artery that likely accounts for the infarction and clinical presentation;

	3) Acute multiple infarctions documented by head CT or MRI, attributed to large-artery atherosclerosis, including non-stenotic vulnerable plaques
**Exclusion criteria**<br>9.Defibrinogen therapy (e.g. defibrase and lumbrokinase) after onset;	**Exclusion criteria**<br>9. Defibrinogen therapy (e.g. defibrase and lumbrokinase), anticoagulation therapy (e.g. argatroban), or antiplatelet therapy (e.g. ticagrelor, tirofiban) except for clopidogrel and aspirin after onset.
**1.3.1 Intensive antiplatelet therapy in patients with acute ischemic stroke or high-risk TIA from atherosclerosis**<br>Although results of the CHANCE trial confirmed that, in patients with TIA or minor stroke treated within 24 hours of onset, DAPT with clopidogrel and aspirin reduced the risk of stroke in the first 90 days compared with aspirin alone and the risk of bleeding did not increase. However, it has not been added to the indication in the package insert of clopidogrel. Some physicians in the real world follow the guidelines, but some follow the instructions. Therefore, patients will be enrolled in this study on a voluntary basis.	**1.3.1 Intensive antiplatelet therapy in patients with acute ischemic stroke or high-risk TIA from atherosclerosis**<br>The results of the pooled analysis of the CHANCE and the POINT trials suggested that DAPT with aspirin and clopidogrel initiated within 24 hours of onset in patients with noncardiogenic minor ischemic stroke (NIHSS score ≤ 3) or high-risk TIA (ABCD2 score ≥ 4) significantly reduced the incidence of major ischemic events relative to aspirin alone, which was also cited by the 2019 American Heart Association/ American Stroke Association Guidelines for the Early Management of Acute Ischemic Stroke as a class A evidence recommendation.<br>Therefore, patients with non-cardiogenic mild ischemic stroke (NIHSS score≤3) or high-risk TIA (ABCD2 score≥4) within 24 hours of onset were not included in this study.
/	**Add the following to 8. Blood Sample Collection:**<br>**8.1 Collection time, place and volume**<br>Two blood samples per patient need to be collected at the enrollment hospital, at the time points of "within 24 hours after randomization" and "90±7 days after randomization"; 10ml blood was collected each time, half of which is collected using non-anticoagulant blood collection tube and EDTA anticoagulant blood collection tube respectively.<br>**8.2 Place and manner of sample processing and storage**

	Blood sample processing is completed at the enrollment hospital, and the Beijing Tiantan Hospital, Capital Medical University provides standardized processing procedures (shown in the biological sample collection and processing manual) and training. The extracted serum, plasma and leukocytes are stored at -20℃~-80℃ at the enrollment hospital after separation, and should be regularly transferred to Beijing Tiantan Hospital, Capital Medical University for centralized storage via cold chain transportation for subsequent testing and analysis.<br>**8.3 Disposal of remaining samples**<br>Surplus samples will be destroyed by Beijing Tiantan Hospital, Capital Medical University.<br>1) Samples that have been used and wastes should be destroyed uniformly after sterilization.<br>2) If there is a special need such as repeat tests, samples can be destroyed after appropriate short-term storage.<br>3) Samples that have not been used 15 years after the end of the study follow-up and wastes should be disinfected and destroyed.<br>4) Biological samples prohibited by the written request of the sample provider and wastes should be disinfected and then uniformly destroyed.<br>5) In some other special conditions, such as sample contamination or the discovery of HIV and other viral infected samples, the project leader will be contacted for sample destruction in a timely manner after discovery.<br>6) All samples destruction need subject leader's knowledge and signature is required<br>7) The method of sample destruction is to use pressure steam sterilization, sterilization temperature 121℃, sterilization time 20 minutes, after the treatment of the sample into a special medical garbage bag according to the hospital medical waste treatment;<br>8) All the disposal process of samples should be recorded accordingly, and the operator should fill in the Sample Destruction Record Form.
