# Supplementary material for: Immediate- or Delayed-Intensive Statin in Acute Cerebral Ischemia: The INSPIRES Randomized Clinical Trial
Source: JAMA Neurol. 2024 May 28;81(7):741–51. doi: 10.1001/jamaneurol.2024.1433 (PMC11134282; doi:10.1001/jamaneurol.2024.1433)
Supplement: Supplement 2. — Statistical Analysis Plan. [file jamaneurol-e241433-s002.pdf]

Supplement 2

Statistical analysis plan for: Delayed Dual Antiplatelet Treatment in Ischemic Stroke.

This statistical analysis plan has been provided by the authors to give readers additional information about the work.

This supplement contains the following items:

No.	Item	Version	Date	Page No.
1	Original statistical analysis plan	V1.0	Apr. 28th, 2018	2 to 20
2	Final statistical analysis plan	V2.0	Jun. 6th, 2020	21 to 39
3	Summary of statistical analysis plan amendments	-	-	40

Intensive Statin and Antiplatelet Therapy for High-risk Intracranial or Extracranial Atherosclerosis (INSPIRES)

Statistical Analysis Plan

Principal Investigator

Yilong Wang, Yongjun Wang

Beijing Tiantan Hospital, Capital Medical University, Beijing, China

Prepared by

Yuesong Pan, PhD

Aoming Jin, PhD

Hongyi Yan, MM

Mengxing Wang, MM

Beijing Tiantan Hospital, Capital Medical University, Beijing, China

Version 1.0

Apr. 28th, 2018

Table of Contents

1. Introduction 4
2. Study Purposes 4
3. Study Outcomes 6
Primary outcome: 6
Secondary outcomes: 6
Safety outcomes 7
4. Statistical Hypotheses 7
5. Design 8
6. Sample size estimates 10
7. Analysis populations 12
Full Analysis Set (FAS)： 12
Per Protocol Set (PPS) 12
Safety Set (SS) 12
8. Treatment comparisons 12
9. General considerations for data analyses 13
Multicenter Studies 13
Examination of Subgroups 13
Multiple Comparisons and Multiplicity 14
10.Data handling conventions 14
Premature Withdrawal and Missing Data 14
Event Rates 15
Time to Event Analysis 15
11.Study Population 15
Disposition of Patients 15
Protocol Deviations 15
Demographic and Baseline Characteristics 16
12. Efficacy Analyses 16
Primary Efficacy Analysis 16
Secondary Efficacy Analyses 17
13. Safety Analyses 18
14. References 19

1. Introduction

This statistical analysis plan (SAP) documents the planned statistical analyses for the INSPIRES study and is based on the protocol, together with any subsequent amendments.

This SAP is intended for the use of project team members and should be read in conjunction with the aforementioned protocol.

2. Study Purposes

The primary purposes of the study are:

1) To evaluate the efficacy and safety of intensive antiplatelet therapy versus standard antiplatelet therapy in reducing the risk of new stroke at 90 days in acute mild ischemic stroke or high-risk transient ischemic attack (TIA) patients attributed to extracranial or intracranial atherosclerosis;
2) To evaluate the efficacy and safety of immediate intensive statin therapy (atorvastatin 80mg/d) versus delayed intensive statin therapy (atorvastatin 40mg/d) in reducing the risk of new stroke at 90 days in acute mild ischemic stroke or high-risk TIA patients attributed to extracranial or intracranial atherosclerosis;
3) To evaluate the efficacy and safety of intensive antiplatelet combined with immediate intensive statin therapy (atorvastatin 80mg/d) versus standard antiplatelet combined with delayed intensive statin therapy (atorvastatin 40mg/d) in reducing the risk of new stroke at 90 days in acute mild ischemic stroke or high-risk TIA patients attributed to extracranial or intracranial atherosclerosis.

The secondary purposes of the study are:

1) To evaluate the efficacy of intensive antiplatelet therapy versus standard antiplatelet therapy, immediate intensive statin therapy (atorvastatin 80mg/d) versus delayed intensive statin therapy (atorvastatin 40mg/d), intensive antiplatelet therapy combined with immediate intensive statin therapy (atorvastatin 80mg/d) versus standard antiplatelet therapy combined with delayed intensive statin therapy respectively (atorvastatin 40mg/d) at 90 days in acute mild ischemic stroke or high-risk TIA patients attributed to intracranial or extracranial atherosclerosis on the incidence of:
   - Combined vascular events: stroke (ischemic or hemorrhagic), myocardial infarction, and cardiovascular death.
   - Ischemic stroke
   - Transient ischemic attack (TIA)
   - Severity of stroke or TIA on an ordinal scale: a six-level ordered category scale combined vascular events with mRS score at 90 days: fatal stroke (stroke with subsequent death), severe stroke (stroke followed by mRS of 4-5), moderate stroke (stroke followed by mRS of 2-3), mild stroke (stroke followed by mRS of 0-1), TIA, and no stroke/TIA
   - Myocardial infarction

- Vascular death
- All-cause death
- Poor functional outcome (mRS score 2-6)
- Poor quality of life (EQ-5D scale index score≤0.5)
- Early neurological deficits (increase in NIHSS score at 7days)

2) To evaluate the efficacy in decreasing early neurological deficits (increase in NIHSS score at 7days), stroke recurrence, all-cause mortality and poor functional outcome (mRS score 2-6) at 1 year of intensive antiplatelet therapy versus standard antiplatelet therapy, immediate intensive statin therapy (atorvastatin 80mg/d) versus delayed intensive statin therapy (atorvastatin 40mg/d), intensive antiplatelet therapy combined with immediate intensive statin therapy (atorvastatin 80mg/d) versus standard antiplatelet therapy combined with delayed intensive statin therapy (atorvastatin 40mg/d) respectively in acute mild ischemic stroke or high-risk TIA patients attributed to intracranial or extracranial atherosclerosis. The new stroke or TIA is classified on a six-level ordered category scale combined vascular events with mRS score at 1 year: fatal stroke (stroke with subsequent death), severe stroke (stroke followed by mRS of 4-5), moderate stroke (stroke followed by mRS of 2-3), mild stroke (stroke followed by mRS of 0-1), TIA, and no stroke/TIA.

Safety purposes of the study are:

1) To evaluate safety of intensive antiplatelet therapy versus standard antiplatelet therapy for 90 days in acute mild ischemic stroke or high-risk TIA patients attributed to intracranial or extracranial atherosclerosis on the incidence of:

- Moderate to severe bleeding
- Intracranial hemorrhage
- Hepatotoxicity: Alanine aminotransferase (ALT) or Aspartate aminotransferase (AST) > 3 times the upper limit of normal value
- Muscle toxicity: Creatine kinase (CK) > 10 times the upper limit of normal value, or the presence of muscle pain, myopathy, or rhabdomyolysis
- Death
- Other adverse events (AEs) / severe adverse events (SAEs)

2) To evaluate safety of immediate intensive statin therapy (atorvastatin 80mg/d) versus delayed intensive statin therapy (atorvastatin 40mg/d) for 90 days in acute mild ischemic stroke or high-risk TIA patients attributed to intracranial or extracranial atherosclerosis on the incidence of :

- Moderate to severe bleeding
- Intracranial hemorrhage

- Hepatotoxicity: Alanine aminotransferase (ALT) or Aspartate aminotransferase (AST) > 3 times the upper limit of normal value;
- Muscle toxicity: Creatine kinase (CK) > 10 times the upper limit of normal value, or the presence of muscle pain, myopathy, or rhabdomyolysis.
- Death
- Other AEs / SAEs

3) To evaluate the safety of intensive antiplatelet therapy combined with immediate intensive statin therapy (atorvastatin 80mg/d) versus standard antiplatelet therapy combined with delay intensive statin therapy (atorvastatin 40mg/d) for 90 days in acute mild ischemic stroke or high-risk TIA patients attributed to intracranial or extracranial atherosclerosis on the incidence of:

- Moderate to severe bleeding
- Intracranial hemorrhage
- Hepatotoxicity: ALT or AST > 3 times the upper limit of normal value;
- Muscle toxicity: CK > 10 times the upper limit of normal value, or the presence of muscle pain, myopathy, or rhabdomyolysis.
- Death
- Other adverse events / severe adverse events

3. Study Outcomes

Primary outcome:

Stroke (ischemic or hemorrhagic)

Secondary outcomes:

1) Combined vascular events : Stroke (ischemic or hemorrhagic), myocardial infarction, or cardiovascular death;
2) Ischemic stroke;
3) TIA;
4) Severity of stroke or TIA on an ordinal scale: (a six-level ordered category scale combined vascular events with mRS score: fatal stroke (stroke with subsequent death), severe stroke (stroke followed by mRS of 4-5), moderate stroke (stroke followed by mRS of 2-3), mild stroke (stroke followed by mRS of 0-1), TIA, and no stroke/TIA);
5) Myocardial infarction;
6) Vascular death;
7) All-cause death;
8) Poor functional outcome (mRS score 2-6);
9) Poor quality of life (EQ-5D scale index score≤0.5);
10) Early neurological deficits (increase in NIHSS score at 7days)

Safety outcomes:

1) Primary safety outcome
   - Moderate to severe bleeding;
2) Secondary safety outcomes
   - Intracranial hemorrhage
   - Hepatotoxicity: ALT or AST > 3 times the upper limit of normal value;
   - Muscle toxicity: CK > 10 times the upper limit of normal value, or the presence of muscle pain, myopathy, or rhabdomyolysis.
   - Death
   - Other AEs / SAEs

4. Statistical Hypotheses

The primary outcome for this study is the recurrence rate of the stroke at the time of 90 days follow-up.

1) In patients with acute mild ischemic stroke or high-risk TIA patients attributed to extracranial or intracranial atherosclerosis treated within 72 hours of ictus, the null hypothesis of no difference in the risk of a new stroke within 90 days between subjects with intensive antiplatelet therapy and those with standard antiplatelet therapy will be tested using a two-sided test at the 5% level of significance.

H_0: $\lambda_1/\lambda_2=1$

H_1: $\lambda_1/\lambda_2\neq1$

Where λ_1 is the recurrence rate of the stroke at the time of 90 days follow-up in the group treated with intensive antiplatelet therapy and λ_2 is the same endpoint in the group treated with standard antiplatelet therapy.

2) In patients with acute mild ischemic stroke or high-risk TIA patients attributed to extracranial or intracranial atherosclerosis treated within 72 hours of ictus, the null hypothesis of no difference in the risk of a new stroke within 90 days between subjects with immediate intensive statin therapy (atorvastatin 80mg/d) and delayed intensive statin therapy (atorvastatin 40mg/d) will be tested using a two-sided test at the 5% level of significance.

H_0: $\lambda_1/\lambda_2=1$

H_1: $\lambda_1/\lambda_2\neq1$

Where λ_1 is the recurrence rate of the stroke at the time of 90 days follow-up in the group treated with immediate intensive statin therapy (atorvastatin 80mg/d) and λ_2 is the same endpoint in the group treated with delayed intensive statin therapy (atorvastatin 40mg/d).

3) In patients with acute mild ischemic stroke or high-risk TIA patients attributed to extracranial or intracranial atherosclerosis treated within 72 hours of ictus, the null hypothesis of no difference in the risk of a new stroke within 90 days between subjects with intensive antiplatelet combined with immediate intensive statin therapy (atorvastatin 80mg/d) and standard antiplatelet combined with

delayed intensive statin therapy (atorvastatin 40mg/d) will be tested using a two-sided test at the 5% level of significance.

H_0: $\lambda_1/\lambda_2=1$

H_1: $\lambda_1/\lambda_2\neq1$

Where λ_1 is the recurrence rate of the stroke at the time of 90 days follow-up in the group treated with intensive antiplatelet combined with immediate intensive statin therapy (atorvastatin 80mg/d) and λ_2 is the same endpoint in the group treated with standard antiplatelet combined with delayed intensive statin therapy (atorvastatin 40mg/d).

5. Design

Study design

- A Randomized, double-blind, placebo-controlled, multicenter, 2×2 factorial trial.
- The trial is intended to enroll 6100 subjects and complete follow-up of all subjects within 5 years.
- Centralized, unified and randomized grouping.
- Subjects will be randomly assigned to the following four groups:

  A: Intensive antiplatelet therapy + immediate intensive statin therapy (atorvastatin 80mg/d)

  B: Intensive antiplatelet therapy + delayed intensive statin therapy (atorvastatin 40mg/d)

  C: Standard antiplatelet therapy + immediate intensive statin therapy (atorvastatin 80mg/d)

  D: Standard antiplatelet therapy + delayed intensive statin therapy (atorvastatin 40mg/d)

Identity of study medication:

Groups	Date after Randomization	Dosage of study medication
Intensive antiplatelet therapy	Day 1	Clopidogrel 300mg/ day + aspirin 100-300mg/ day
	Day 2 - Day21±2	Clopidogrel 75mg/ day + aspirin 100mg/ day
	Day22±2 - Day 90	Clopidogrel 75mg/ day + aspirin placebo
Standard antiplatelet therapy	Day 1	Aspirin 100-300mg/ day + clopidogrel placebo
	Day 2 - Day 90	Aspirin 100mg/ day + clopidogrel placebo
Immediate intensive statin therapy	Day 1 - Day21±2	Atorvastatin 80mg/ day
	Day22±2 - Day 90	Atorvastatin 40mg/ day
Delayed intensive statin therapy	Day 1 - Day3	Atorvastatin placebo
	Day 4 - Day21±2	Atorvastatin 40mg/ day + atorvastatin placebo
	Day22±2 - Day 90	Atorvastatin 40mg/ day

Research design(drawing)

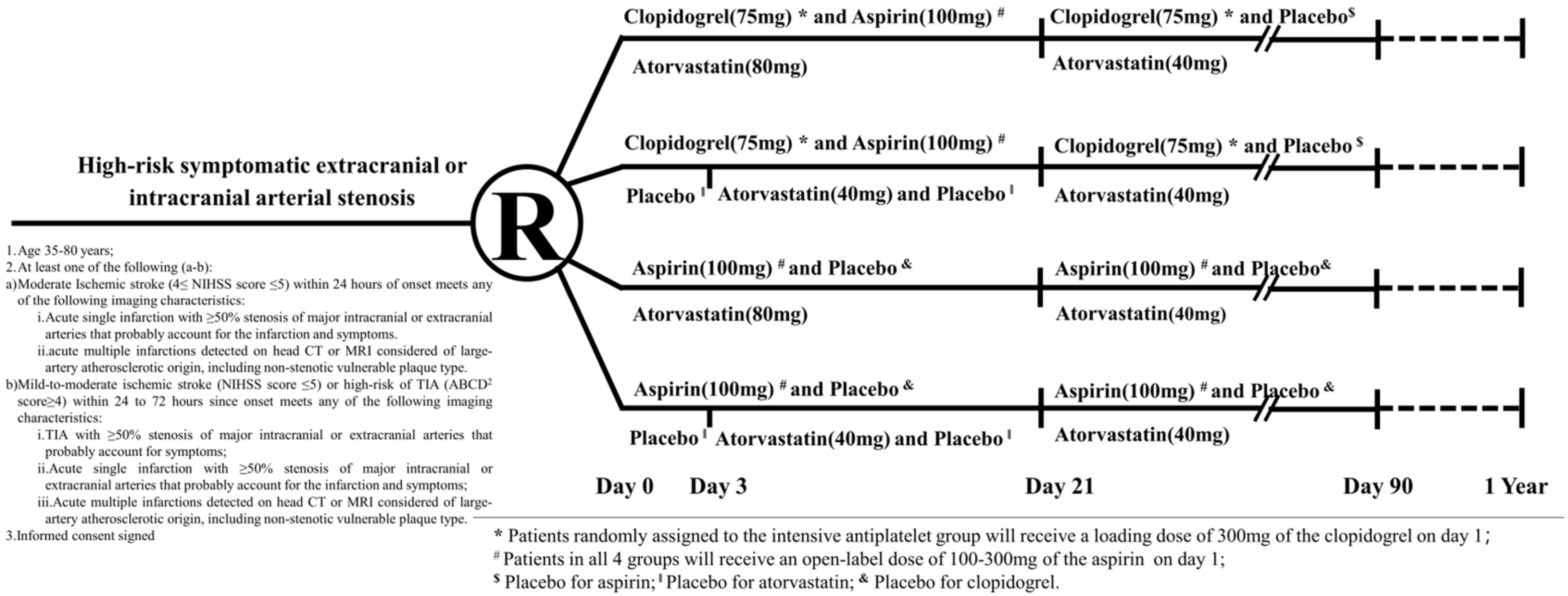

Follow-up plan

Subjects will receive a face-to-face visit at baseline, Day7±2, Day14 (or hospital discharge), Day 21±2 and Day90 ± 7, and telephone visits at the 6th month±14 days and 12th month±14 days after randomization. In addition, patients will be interviewed when new neurologic symptoms or suspicious events occur, including worsening of index ischemic events, new transient or persistent neurological symptoms.

Planned Analyses

The analyses that are detailed in this SAP will be performed only when the database has been locked, all protocol violators identified, and treatment allocations have been unblinded. Membership of the Full Analysis and Per Protocol populations will be determined using the rules set out in this SAP. At a date to be agreed within the project team, a data look will be performed. This will involve production of all data displays on a subset of the data using dummy treatment codes. These are produced purely as an aide to the pre-programming of the study and no unblinding will occur.

Interim Analyses

No interim analyses are planned for this study. However, a Data and Safety Monitoring Board (DSMB) is in place to ensure the safety of patients in the study. An independent statistician will prepare unblinded summary tables of SAEs, selected demographic data and patients exposure data and these will be examined by the DSMB. These tables will be provided to the DSMB at regular intervals. If the tables give rise to safety concerns for any treatment, the DSMB may recommend that the trial should be modified or stopped prematurely. The Steering Committee will, in conjunction with the Sponsor, decide whether to act on this recommendation. Further discussion of these safety tabulations is provided in a specific study protocol.

6. Sample size estimates

The minimal sample size for the trial is determined by the necessity that a clinically meaningful difference in effectiveness between treatment and control groups has to be detected. Based on previous studies, the risk of new stroke during 90 days is presumed to be 11.5% in the group with standard antiplatelet therapy (with half delayed intensive statin therapy and half early high-intensity statin therapy) and 11.5% in the delayed intensive statin therapy group (with half standard antiplatelet therapy and half dual antiplatelet therapy) and 13% in the group with standard antiplatelet therapy plus delayed intensive statin therapy, intensive antiplatelet therapy and immediate intensive statin therapy can reduce this risk by 22%, and the effects of intensive antiplatelet and lipid lowering therapy will be similar and additive. With a sample size of 6100 subjects, a two-sided α of 0.05 and 5% loss to follow up, we will have 97% power to detect that the risk is decreased by 35% in the group with dual antiplatelet therapy plus immediate intensive statin therapy compared to standard antiplatelet therapy plus delayed intensive therapy, and 80% power to detect that the risk is reduced by 20% by intensive

antiplatelet therapy compared to standard antiplatelet therapy, and immediate intensive statin compared to delayed intensive statin therapy, respectively.

Table1 For different risk rates (12% to 14%) of the results in the control group, the sample size required for observing intensive antiplatelet combining intensive statin therapy

Power	A single set of sample size (no expulsion)	A single set of sample size (5% expulsion rate)	Four groups of sample size	The stroke recurrence rate in treatment group	The stroke recurrence rate in control group	Relative risk
0.966	1448	1525	6100	0.078	0.120	0.65
0.975	1448	1525	6100	0.085	0.130	0.65
0.985	1448	1525	6100	0.091	0.140	0.65
0.824	1448	1525	6100	0.096	0.130	0.74

Table2 For different risk rates (12% to 14%) of the results in the control group, the sample size required for the marginal effect analysis of observed intensive antiplatelet combining intensive statin therapy

Power	A single set of sample size (no expulsion)	A single set of sample size (5% expulsion rate)	Four groups of sample size	The stroke recurrence rate in treatment group	The stroke recurrence rate in control group	Relative risk
0.807	2896	3050	6100	0.0930	0.1157	0.80

Explanations of data sources on recurrence risk and the risk of stroke recurrence after treatment:

At present, evidence in early risks of recurrent stroke in high-risk symptomatic intracranial or extracranial artery stenosis patients is still lacking. The data currently available for reference include: a). In the studies of WASID and SAMMPRIS, the recurrence risk of stroke in the medication treatment group with symptomatic intracranial artery stenosis (stenosis rate > 70%) were 21.9% and 12.6% at 1 year, respectively [1,2]. The recurrence risk of 12.6% at 1 year in SAMMPRIS research was based on the combination of dual anti-platelet therapy, intensive lipid-lowering and blood-pressure-lowering therapy. Therefore, for the recurrence risk of the standard therapy group, 13% was a conservative figure in our study [3]. b) The subgroup study of the CHANCE Trial has revealed a recurrence risk of 12.5% in patients with intracranial arterial stenosis (stenosis rate > 50%) at day 90[4]. In the CHANCE trial, patients with intracranial arterial stenosis associated with multiple infarcts had a recurrent stroke risk of a striking number of 18% at 90 days [5]. Therefore, as the recurrence risk of the standard therapy group, 13% was a conservative data in our study.

Concerning that dual anti-platelet therapy reduces the risk of recurrence of 22%, the CHANCE study confirmed that dual anti-platelet therapy decreased the risk of stroke recurrence by 90 days for TIA and minor stroke by 32% compared with anti-platelet monotherapy [6]. In the CHANCE subgroup, compared with anti-platelet monotherapy, dual anti-platelet therapy decreased the relative risk of stroke

recurrence by 21% in patients with intracranial arterial stenosis [4], and by 50% in patients with the multiple cerebral infarction [7]. Therefore, with dual anti-platelet therapy, our estimate of a relative reduction of 22% in recurrent stroke risk was a conservative data in our study.

Concerning the source of evidence for intensive lipid-lowering combined with intensive antiplatelet therapy decreases the risk of stroke by 35%, a comparative study of SAMMPRIS and WASID before and after pointed out that the risks of recurrent stroke were 21.9% and 12.6% in the intensive drug therapy group and anti-platelet monotherapy group at 1 year, respectively. The risk was decreased by about 50%. Therefore, the estimated reduction of recurrence risk by 35% was a conservative data in our study. [2].

7. Analysis populations

Full Analysis Set (FAS):

The full analysis set is the main efficacy evaluation population of this study, and all valid variables will be analyzed by FAS. According to the basic principle of intention-to-treat analysis (ITT), all subjects randomized into groups and those with more than one medication record and efficacy evaluation will be included in the full analysis set.

Per Protocol Set (PPS)

Includes all subjects who complete the protocol and have no major deviation of the trial. The exact definition of a major deviation of the program will be finalized at the time of data review and may generally include the following situations (but not limited to these cases): failing to meet the main inclusion criteria, serious disturbance of drug efficacy after treatment, poor compliance, exceeding the time window of following-up and so on. PPS is the secondary analysis of the effectiveness of the crowd, but if the results are inconsistent with the whole analysis set, detailed analysis of the inconsistent results are needed.

Safety Set (SS)

A safety data set is defined as a subject that receives drug treatment at least once. In the entire safety analysis, patients with incorrect treatment (for example, randomized to the standard antiplatelet combining delayed intensive statin therapy groups were given enhanced antiplatelet therapy) would be assigned to the actual treatment group.

8. Treatment comparisons

The treatment comparisons of interest in this study are:

1) To evaluate the efficacy and safety of intensive antiplatelet therapy versus standard antiplatelet therapy in reducing the risk of new stroke at 90 days in acute mild ischemic stroke or high-risk TIA patients attributed to extracranial or intracranial atherosclerosis;
2) To evaluate the efficacy and safety of immediate intensive statin therapy (atorvastatin 80mg/d) versus delayed intensive statin therapy (atorvastatin 40mg/d) in reducing the risk of new stroke at

90 days in acute mild ischemic stroke or high-risk TIA patients attributed to extracranial or intracranial atherosclerosis;

3) To evaluate the efficacy and safety of intensive antiplatelet combined with immediate intensive statin therapy (atorvastatin 80mg/d) versus standard antiplatelet combined with delayed intensive statin therapy (atorvastatin 40mg/d) in reducing the risk of new stroke at 90 days in acute mild ischemic stroke or high-risk TIA patients attributed to extracranial or intracranial atherosclerosis.

9. General considerations for data analyses

All programming will be performed using SAS Version 9.4. All analysis output will use the following treatment group naming conventions and treatment order: intensive antiplatelet therapy, standard antiplatelet therapy, immediate intensive statin therapy and delayed intensive statin therapy.

All statistics were two sided with a $P<0.05$ considered significant, and the confidence interval was reliable two sided with 95%.

Multicenter Studies

Centers with less than 20 patients will be pooled with larger centers within the same geographic region so that centers are of a reasonable size for the purpose of the statistical analyses. This process will be performed and finalized before the treatment codes are unblinded.

In multicenter randomized controlled clinical study, there were some different effect in different center due to different baseline, clinical practice or other factor, therefore, center effect analysis was required. Stratified analysis was used to exclude the mixed effect of results caused by center effect: each center was served as a stratum, calculating the HR by Cox proportional hazards model.

Examination of Subgroups

1) To evaluate the efficacy of intensive antiplatelet therapy versus standard antiplatelet therapy, immediate intensive statin therapy (atorvastatin 80mg/d) versus delayed intensive statin therapy (atorvastatin 40mg/d), and intensive antiplatelet combined with immediate intensive statin therapy (atorvastatin 80mg/d) versus standard antiplatelet combined with delayed intensive statin therapy (atorvastatin 40mg/d) in reversing intracranial atherosclerotic artery stenosis and stabilizing the atherosclerotic vulnerable plaque in the high resolution MRI subgroup.
2) To evaluate the efficacy of intensive antiplatelet therapy versus standard antiplatelet therapy for 90 days on the incidence of the primary outcome in different subgroups:

- Subjects aged > 65 years vs. those aged ≤65 years.
- Female vs. male patients.
- Those with NIHSS score 4-5 vs. those with NIHSS score ≤3 at admission.
- Those randomized within 24 hours of onset vs. those randomized between 24 and 72 hours since onset.
- Those with ischemic stroke/TIA related to extracranial artery atherosclerosis vs. those related to

intracranial artery atherosclerosis.

- Those with intracranial stenosis vs. those without intracranial stenosis.
- Those with extracranial stenosis vs. those without extracranial stenosis.
- Those with multiple infarctions vs. those with single infarction vs. those without infarction.
- Those with severe stenosis (≥70%) vs. those with moderate stenosis (50%-69%).
- Those with hypertension vs. those who are normotensive.
- Diabetic patients vs. nondiabetics.
- Those with dyslipidaemia vs. without dyslipidaemia.
- Those with atherogenic dyslipidaemia (HDL-C<40 mg/dL and TG>200 mg/dL) vs. without atherogenic dyslipidaemia.
- Those with statin therapy within 1 month before randomization vs. without statin therapy.

In addition, relevant subgroups will be examined for genetic variability and biomarker characteristics.

3) To evaluate the efficacy of immediate intensive statin therapy (atorvastatin 80mg/d) versus delayed intensive statin therapy (atorvastatin 40mg/d) for 90 days on the incidence of the primary outcome in subgroups which is the same as above.

Multiple Comparisons and Multiplicity

A single primary efficacy variable has been defined for this study, with all other efficacy variables identified as secondary or other. Comparison of intensive antiplatelet therapy versus standard antiplatelet therapy, immediate intensive statin therapy versus delayed intensive statin therapy, intensive antiplatelet combined with immediate intensive statin therapy versus standard antiplatelet combined with delayed intensive statin therapy were conducted under separate hypotheses; therefore, there are no requirements to adjust for multiple comparisons or multiple endpoints within this study.

10. Data handling conventions

Premature Withdrawal and Missing Data

1) Loss to follow-up

Investigators should try their best to keep contact with every patient, making sure the reason of loss to follow-up and their health situation. All CRFs of missed patients should be recorded until the last follow-up visit.

2) Quit from study

Those who quit the study should not be included again. The randomization number and study drugs of this subject should not be used again. Randomized patients must not be replaced. Investigators should confirm the withdrawal along with the monitoring committee. Subjects who were randomized and had one or more doses recorded were required to complete the calendar visits according to the protocol.

Event Rates

The number of people of events should be recorded in detail and showing the event rate in 90 days of each treatment group in summary statement.

The event rate for each treatment group will be calculated as: the sum of number of event for all the patients / the number of patients enrolled in this group.

Time to Event Analysis

Differences between treatments in the risk of recurrent stroke event and clinical vascular events during maximum 90-day follow-up were assessed using standard Kaplan-Meier time-to-event approaches. The time to the first event was used in the model when there were multiple events of the same type. Patients were considered censored at the time of study termination or death if there were no events occurred during the study.

11. Study Population

Disposition of Patients

The number of patients in each analysis population will be presented, patients to be excluded from the Per Protocol population will be listed, and the total number of patients attending each clinic visit will also be summarized by treatment group.

The number of patients randomized, completed and prematurely withdrawn from the study will be presented for each treatment group. The primary reasons for withdrawal both prior to and post randomization will also be presented.

A data display listing and summary of deviations from the inclusion/exclusion criteria will be presented for all patients who were either entered or randomized into the trial.

Protocol Deviations

Patient data will be examined for evidence of protocol violators in order to assess how well the protocol was followed. Inclusion and exclusion criteria are detailed in the study protocol.

Patients who commit protocol violations will be included in the FAS Population but excluded from the Per Protocol Population. These protocol violations will be shown in a listing. Patients can either be full or partial protocol violators. A full protocol violator is completely excluded from the Per Protocol Population. A partial protocol violator has only some data excluded. For patients who violated the protocol during the treatment period due to unpermitted changes in the medication or prohibited concurrent medication, the analysis will only use data recorded prior to the violation. For all violations which reference the treatment period, the treatment start date will be used as the reference date.

A listing of all possible protocol violators will be produced for clinical review. The final list of patients who are protocol violators and are therefore excluded from the Per-Protocol population will be agreed by the study team.

Demographic and Baseline Characteristics

Demographic, Medical, histories and baseline characteristics information will be listed and summarized for patients in each treatment group based on the FAS population.

Vital signs including supine systolic blood pressure, diastolic blood pressure, and heart rate will also be listed and summarized in each treatment group.

The continuous data followed normal distribution will be presented as mean and standard deviation, and the continuous data followed skewness distribution will be presented as median and interquartile range; categorical data will be presented as n (%). T-test or Wilcoxon rank sum test will be used for comparison between two continuous data, and Chi-squared tests or Fisher exact test will be used for comparison between two categorical data.

12. Efficacy Analyses

Primary Efficacy Analysis

The primary endpoint is stroke (both hemorrhagic and ischemic stroke). FAS will be the primary population for efficacy analyses. PPS will be used as secondary population for the efficacy analyses. If the results in the PPS population are inconsistent with the FAS population, detailed analysis of the inconsistent results is required.

Main Model

The time to stroke (both hemorrhagic and ischemic stroke) reported during the 90-day treatment period for the ITT Population will be summarized by treatment group using Kaplan-Meier estimates. The hazard ratio for the treatment comparison will be derived using a Cox proportional hazards model, including the pooled study center as a random effect. The hazards ratios (HR) with 95% CI will be reported. This will also be presented graphically on a Kaplan-Meier plot. The log-rank test will be used to evaluate the statistical significance of the treatment effect.

Interactions with Subgroups

To evaluate the efficacy of intensive antiplatelet therapy versus standard antiplatelet therapy, immediate intensive statin therapy (atorvastatin 80mg/d) versus delayed intensive statin therapy (atorvastatin 40mg/d), and intensive antiplatelet combined with immediate intensive statin therapy (atorvastatin 80mg/d) versus standard antiplatelet combined with delayed intensive statin therapy (atorvastatin 40mg/d) in reversing intracranial atherosclerotic artery stenosis and stabilizing the atherosclerotic vulnerable plaque in the high resolution MRI subgroup.

To evaluate the efficacy of intensive antiplatelet therapy versus standard antiplatelet therapy for 90 days on the incidence of the primary outcome in different subgroups:

- Subjects aged > 65 years vs. those aged ≤65 years.
- Female vs. male patients.
- Those with NIHSS score 4-5 vs. those with NIHSS score ≤3 at admission.

- Those randomized within 24 hours of onset vs. those randomized between 24 and 72 hours since onset.
- Those with ischemic stroke/TIA related to extracranial artery atherosclerosis vs. those related to intracranial artery atherosclerosis.
- Those with intracranial stenosis vs. those without intracranial stenosis.
- Those with extracranial stenosis vs. those without extracranial stenosis.
- Those with multiple infarctions vs. those with single infarction vs. those without infarction.
- Those with severe stenosis (≥70%) vs. those with moderate stenosis (50%-69%).
- Those with hypertension vs. those who are normotensive.
- Diabetic patients vs. nondiabetics.
- Those with dyslipidaemia vs. without dyslipidaemia.
- Those with atherogenic dyslipidaemia (HDL-C<40 mg/dL and TG>200 mg/dL) vs. without atherogenic dyslipidaemia.
- Those with statin therapy within 1 month before randomization vs. without statin therapy.

In addition, relevant subgroups will be examined for genetic variability and biomarker characteristics.

To evaluate the efficacy of immediate intensive statin therapy (atorvastatin 80mg/d) versus delayed intensive statin therapy (atorvastatin 40mg/d) for 90 days on the incidence of the primary outcome in subgroups which is the same as above.

Secondary Efficacy Analyses

Combined vascular events: Strokes (ischemic or hemorrhagic), myocardial infarction, and cardiovascular death

Combined vascular events: Strokes (ischemic or hemorrhagic), myocardial infarction, and cardiovascular death will be compared by Chi-squared tests. The difference of the incidence rates between two groups with 95% CI will be reported. Cox proportional risk model including the pooled study center as a random effect will be used to calculate HR and the 95% confidence interval and the log-rank test would be used to evaluate the efficacy;

Ischemic stroke

The difference of the rates between two groups with 95% CI will be reported. Cox proportional risk model will be used to calculate HR and the 95% confidence interval and the log-rank test would be used to evaluate the efficacy;

Transient ischemic attack (TIA)

The difference of the rates between two groups with 95% CI will be reported. Cox proportional risk model including the pooled study center as a random effect will be used to calculate HR and the 95% confidence interval and the log-rank test would be used to evaluate the efficacy;

Severity of stroke or TIA on an ordinal scale: (a six-level ordered category scale combined vascular events with mRS score: fatal stroke (stroke with subsequent death), severe stroke (stroke followed by mRS of 4-5), moderate stroke (stroke followed by mRS of 2-3), mild stroke (stroke followed by mRS of 0-1), TIA, and no stroke/TIA)

The difference of the rates between two groups with 95% CI will be reported. Ordinal logistic regression test including the pooled study center as a random effect will be used to calculate common odds ratio (OR) and the 95% confidence interval;

Myocardial infarction

The difference of the rates between two groups with 95% CI will be reported. Cox proportional risk model including the pooled study center as a random effect will be used to calculate HR and the 95% confidence interval and the log-rank test would be used to evaluate the efficacy;

Vascular death

The difference of the rates between two groups with 95% CI will be reported. Cox proportional risk model including the pooled study center as a random effect will be used to calculate HR and the 95% confidence interval and the log-rank test would be used to evaluate the efficacy;

All-cause death

The difference of the rates between two groups with 95% CI will be reported. Cox proportional risk model including the pooled study center as a random effect will be used to calculate HR and the 95% confidence interval and the log-rank test would be used to evaluate the efficacy;

Poor functional outcome (mRS score 2-6)

The difference of the rates between two groups with 95% CI will be reported. Logistic regression test will be used to calculate OR and the 95% confidence interval;

Poor quality of life (EQ-5D scale index score≤0.5)

The difference of the rates between two groups with 95% CI will be reported. Logistic regression test will be used to calculate OR and the 95% confidence interval;

Early neurological deficits (NIHSS score increase of no less than 2points within 7 days)

The difference of the rates between two groups with 95% CI will be reported. Logistic regression test will be used to calculate OR and the 95% confidence interval;

All statistical data will be inspected with two-sided $P<0.05$ as statistically significant.

13. Safety Analyses

The overall of safety assessment is all used test medications and the safety follow-up case should be recorded at least once. The safety evaluation data includes adverse reactions observed during the trial and changes in laboratory data before and after treatment.

Primary safety outcome

- Moderate to severe bleeding

Secondary safety outcomes

- Intracranial hemorrhage
- Hepatotoxicity: ALT or AST > 3 times the upper limit of normal value;
- Muscle toxicity: CK > 10 times the upper limit of normal value, or the presence of muscle pain, myopathy, or rhabdomyolysis.
- Death
- Other AEs / SAEs

Safety evaluation will be analyzed using safety data set.

- Moderate to severe bleeding, intracranial hemorrhage, and overall mortality will be calculated using the Kaplan-Meier curve to simulate the 3-month cumulative risk, and the Cox proportional hazards model to calculate the HR and 95% confidence interval.
- For hepatotoxicity, muscle toxicity and other adverse events and serious adverse events, the cases which was normal before the treatment and abnormal after the treatment would be mainly analyzed and listed, in order to the comparison of differences before and after treatment.

Adverse Events

Adverse events (AEs) will be coded using the MedDRA coding dictionary (Version 6.0 or a later release) and grouped by system organ class (as detailed in the study protocol). Separate data display listings and summaries will be presented for adverse events that start prior to first dose of study medication (pre-treatment), whilst on study medication (during treatment) and after the last dose of study medication (post-treatment).

Within each treatment group, the number and percentage of patients experiencing an AE will be summarized by system organ class and preferred term and Fisher's Exact test will be used to compare the number of each grouped AE event between treatment groups. In addition, a separate summary will be provided for AEs experienced by more than 5% of patients in either of the treatment groups.

Serious Adverse Events

Summary tables and data displays will be provided for serious adverse events (as detailed in the study protocol). In addition, all serious AE's will be documented in a case narrative format in the clinical study report. The number of events occurring over the treatment period will be summarized and Fisher's Exact test will be used to compare the number of events between treatment groups.

14. References

1. Chimowitz MI, Lynn MJ, Derdeyn CP, et al. Stenting versus aggressive medical therapy for

intracranial arterial stenosis. N Engl J Med. 2011 Sep 15;365(11): 993-1003.

2. Chaturvedi S, Turan TN, Lynn MJ, et al. Do Patient Characteristics Explain the Differences in Outcome Between Medically Treated Patients in SAMMPRIS and WASID? Stroke. 2015 Sep;46(9)：2562-7.
3. Waters MF, Hoh BL, Lynn MJ, et al. Factors Associated with Recurrent Ischemic Stroke in the Medical Group of the SAMMPRIS Trial. JAMA Neurol. 2016;73(3): 308-15.
4. Liu L, Wong KS, Leng X, et al. Dual antiplatelet therapy in stroke and ICAS: Subgroup analysis of CHANCE. Neurology, 2015,85(13):1154-1162.
5. Pan Y, Meng X, Jing J, et al. Association of multiple infarctions and ICAS with outcomes of minor stroke and TIA. Neurology. 2017; 88:1081-1088.
6. Wang Y, Wang Y, Zhao X, et al. Clopidogrel with aspirin in acute minor stroke or transient ischemic attack. N Engl J Med. 2013 Jul 4;369(1): 11-9.
7. Jing J, Meng X, Zhao X, et al. Dual antiplatelet therapy in transient ischemic attack and minor stroke with different infarction patterns: Subgroup analysis of chance randomized clinical trial. JAMA Neurology. 2018.

Intensive Statin and Antiplatelet Therapy for High-risk Intracranial or Extracranial Atherosclerosis (INSPIRES)

Statistical Analysis Plan

Principal Investigator

Yilong Wang, Yongjun Wang

Beijing Tiantan Hospital, Capital Medical University, Beijing, China

Prepared by

Yuesong Pan, PhD

Aoming Jin, PhD

Hongyi Yan, MM

Mengxing Wang, MM

Beijing Tiantan Hospital, Capital Medical University, Beijing, China

Version 2.0

Jun. 6th, 2020

Table of Contents

1. Introduction 23
2. Study Purposes 23
3. Study Outcomes 25
Primary outcome: 25
Secondary outcomes: 25
Safety outcomes 26
4. Statistical Hypotheses 26
5. Design 27
6. Sample size estimates 29
7. Analysis populations 31
Full Analysis Set (FAS)： 31
Per Protocol Set (PPS) 31
Safety Set (SS) 31
8. Treatment comparisons 31
9. General considerations for data analyses 32
Multicenter Studies 32
Examination of Subgroups 32
Multiple Comparisons and Multiplicity 33
10. Data handling conventions 33
Premature Withdrawal and Missing Data 33
Event Rates 34
Time to Event Analysis 34
11. Study Population 34
Disposition of Patients 34
Protocol Deviations 34
Demographic and Baseline Characteristics 35
12. Efficacy Analyses 35
Primary Efficacy Analysis 35
Secondary Efficacy Analyses 36
13. Safety Analyses 38
14. References 39

1. Introduction

This statistical analysis plan (SAP) documents the planned statistical analyses for the INSPIRES study and is based on the protocol, together with any subsequent amendments.

This SAP is intended for the use of project team members and should be read in conjunction with the aforementioned protocol.

2. Study Purposes

The primary purposes of the study are:

1) To evaluate the efficacy and safety of intensive antiplatelet therapy versus standard antiplatelet therapy in reducing the risk of new stroke at 90 days in acute mild ischemic stroke or high-risk transient ischemic attack (TIA) patients attributed to extracranial or intracranial atherosclerosis;
2) To evaluate the efficacy and safety of immediate intensive statin therapy (atorvastatin 80mg/d) versus delayed intensive statin therapy (atorvastatin 40mg/d) in reducing the risk of new stroke at 90 days in acute mild ischemic stroke or high-risk TIA patients attributed to extracranial or intracranial atherosclerosis;
3) To evaluate the efficacy and safety of intensive antiplatelet combined with immediate intensive statin therapy (atorvastatin 80mg/d) versus standard antiplatelet combined with delayed intensive statin therapy (atorvastatin 40mg/d) in reducing the risk of new stroke at 90 days in acute mild ischemic stroke or high-risk TIA patients attributed to extracranial or intracranial atherosclerosis.

The secondary purposes of the study are:

1) To evaluate the efficacy of intensive antiplatelet therapy versus standard antiplatelet therapy, immediate intensive statin therapy (atorvastatin 80mg/d) versus delayed intensive statin therapy (atorvastatin 40mg/d), intensive antiplatelet therapy combined with immediate intensive statin therapy (atorvastatin 80mg/d) versus standard antiplatelet therapy combined with delayed intensive statin therapy respectively (atorvastatin 40mg/d) at 90 days in acute mild ischemic stroke or high-risk TIA patients attributed to intracranial or extracranial atherosclerosis on the incidence of:
   - Combined vascular events: stroke (ischemic or hemorrhagic), myocardial infarction, and cardiovascular death.
   - Ischemic stroke
   - Transient ischemic attack (TIA)
   - Severity of stroke or TIA on an ordinal scale: a six-level ordered category scale combined vascular events with mRS score at 90 days: fatal stroke (stroke with subsequent death), severe stroke (stroke followed by mRS of 4-5), moderate stroke (stroke followed by mRS of 2-3), mild stroke (stroke followed by mRS of 0-1), TIA, and no stroke/TIA
   - Myocardial infarction

- Vascular death
- All-cause death
- Poor functional outcome (mRS score 2-6)
- Poor quality of life (EQ-5D scale index score≤0.5)
- Early neurological deficits (increase in NIHSS score at 7days)

2) To evaluate the efficacy in decreasing early neurological deficits (increase in NIHSS score at 7days), stroke recurrence, all-cause mortality and poor functional outcome (mRS score 2-6) at 1 year of intensive antiplatelet therapy versus standard antiplatelet therapy, immediate intensive statin therapy (atorvastatin 80mg/d) versus delayed intensive statin therapy (atorvastatin 40mg/d), intensive antiplatelet therapy combined with immediate intensive statin therapy (atorvastatin 80mg/d) versus standard antiplatelet therapy combined with delayed intensive statin therapy (atorvastatin 40mg/d) respectively in acute mild ischemic stroke or high-risk TIA patients attributed to intracranial or extracranial atherosclerosis. The new stroke or TIA is classified on a six-level ordered category scale combined vascular events with mRS score at 1 year: fatal stroke (stroke with subsequent death), severe stroke (stroke followed by mRS of 4-5), moderate stroke (stroke followed by mRS of 2-3), mild stroke (stroke followed by mRS of 0-1), TIA, and no stroke/TIA.

Safety purposes of the study are:

1) To evaluate safety of intensive antiplatelet therapy versus standard antiplatelet therapy for 90 days in acute mild ischemic stroke or high-risk TIA patients attributed to intracranial or extracranial atherosclerosis on the incidence of:

- Moderate to severe bleeding
- Intracranial hemorrhage
- Hepatotoxicity: Alanine aminotransferase (ALT) or Aspartate aminotransferase (AST) > 3 times the upper limit of normal value
- Muscle toxicity: Creatine kinase (CK) > 10 times the upper limit of normal value, or the presence of muscle pain, myopathy, or rhabdomyolysis
- Death
- Other adverse events (AEs) / severe adverse events (SAEs)

2) To evaluate safety of immediate intensive statin therapy (atorvastatin 80mg/d) versus delayed intensive statin therapy (atorvastatin 40mg/d) for 90 days in acute mild ischemic stroke or high-risk TIA patients attributed to intracranial or extracranial atherosclerosis on the incidence of :

- Moderate to severe bleeding
- Intracranial hemorrhage

- Hepatotoxicity: Alanine aminotransferase (ALT) or Aspartate aminotransferase (AST) > 3 times the upper limit of normal value
- Muscle toxicity: Creatine kinase (CK) > 10 times the upper limit of normal value, or the presence of muscle pain, myopathy, or rhabdomyolysis
- Death
- Other AEs / SAEs

3) To evaluate the safety of intensive antiplatelet therapy combined with immediate intensive statin therapy (atorvastatin 80mg/d) versus standard antiplatelet therapy combined with delay intensive statin therapy (atorvastatin 40mg/d) for 90 days in acute mild ischemic stroke or high-risk TIA patients attributed to intracranial or extracranial atherosclerosis on the incidence of:

- Moderate to severe bleeding
- Intracranial hemorrhage
- Hepatotoxicity: ALT or AST > 3 times the upper limit of normal value;
- Muscle toxicity: CK > 10 times the upper limit of normal value, or the presence of muscle pain, myopathy, or rhabdomyolysis.
- Death
- Other adverse events / severe adverse events

3. Study Outcomes

Primary outcome:

Stroke (ischemic or hemorrhagic)

Secondary outcomes:

1) Combined vascular events : Stroke (ischemic or hemorrhagic), myocardial infarction, or cardiovascular death;

2) Ischemic stroke;

3) TIA;

4) Severity of stroke or TIA on an ordinal scale: (a six-level ordered category scale combined vascular events with mRS score: fatal stroke (stroke with subsequent death), severe stroke (stroke followed by mRS of 4-5), moderate stroke (stroke followed by mRS of 2-3), mild stroke (stroke followed by mRS of 0-1), TIA, and no stroke/TIA);

5) Myocardial infarction;

6) Vascular death;

7) All-cause death;

8) Poor functional outcome (mRS score 2-6);

9) Poor quality of life (EQ-5D scale index score≤0.5);

10) Early neurological deficits (increase in NIHSS score at 7days)

Safety outcomes

1) Primary safety outcome

- Moderate to severe bleeding;

2) Secondary safety outcomes

- Intracranial hemorrhage
- Hepatotoxicity: ALT or AST > 3 times the upper limit of normal value;
- Muscle toxicity: CK > 10 times the upper limit of normal value, or the presence of muscle pain, myopathy, or rhabdomyolysis.
- Death
- Other AEs / SAEs

4. Statistical Hypotheses

The primary outcome for this study is the recurrence rate of the stroke at the time of 90 days follow-up.

1) In patients with acute mild ischemic stroke or high-risk TIA patients attributed to extracranial or intracranial atherosclerosis treated within 72 hours of ictus, the null hypothesis of no difference in the risk of a new stroke within 90 days between subjects with intensive antiplatelet therapy and those with standard antiplatelet therapy will be tested using a two-sided test at the 5% level of significance.

H_0: $\lambda_1/\lambda_2=1$

H_1: $\lambda_1/\lambda_2\neq1$

Where λ_1 is the recurrence rate of the stroke at the time of 90 days follow-up in the group treated with intensive antiplatelet therapy and λ_2 is the same endpoint in the group treated with standard antiplatelet therapy.

2) In patients with acute mild ischemic stroke or high-risk TIA patients attributed to extracranial or intracranial atherosclerosis treated within 72 hours of ictus, the null hypothesis of no difference in the risk of a new stroke within 90 days between subjects with immediate intensive statin therapy (atorvastatin 80mg/d) and delayed intensive statin therapy (atorvastatin 40mg/d) will be tested using a two-sided test at the 5% level of significance.

H_0: $\lambda_1/\lambda_2=1$

H_1: $\lambda_1/\lambda_2\neq1$

Where λ_1 is the recurrence rate of the stroke at the time of 90 days follow-up in the group treated with immediate intensive statin therapy (atorvastatin 80mg/d) and λ_2 is the same endpoint in the group treated with delayed intensive statin therapy (atorvastatin 40mg/d).

3) In patients with acute mild ischemic stroke or high-risk TIA patients attributed to extracranial or intracranial atherosclerosis treated within 72 hours of ictus, the null hypothesis of no difference in the risk of a new stroke within 90 days between subjects with intensive antiplatelet combined with immediate intensive statin therapy (atorvastatin 80mg/d) and standard antiplatelet combined with

delayed intensive statin therapy (atorvastatin 40mg/d) will be tested using a two-sided test at the 5% level of significance.

H_0: $\lambda_1/\lambda_2=1$

H_1: $\lambda_1/\lambda_2\neq1$

Where λ_1 is the recurrence rate of the stroke at the time of 90 days follow-up in the group treated with intensive antiplatelet combined with immediate intensive statin therapy (atorvastatin 80mg/d) and λ_2 is the same endpoint in the group treated with standard antiplatelet combined with delayed intensive statin therapy (atorvastatin 40mg/d).

5. Design

Study design

- A Randomized, double-blind, placebo-controlled, multicenter, 2×2 factorial trial.
- The trial is intended to enroll 6100 subjects and complete follow-up of all subjects within 5 years.
- Centralized, unified and randomized grouping.
- Subjects will be randomly assigned to the following four groups:

A: Intensive antiplatelet therapy + immediate intensive statin therapy (atorvastatin 80mg/d)

B: Intensive antiplatelet therapy + delayed intensive statin therapy (atorvastatin 40mg/d)

C: Standard antiplatelet therapy + immediate intensive statin therapy (atorvastatin 80mg/d)

D: Standard antiplatelet therapy + delayed intensive statin therapy (atorvastatin 40mg/d)

Identity of study medication:

Groups	Date after Randomization	Dosage of study medication
Intensive antiplatelet therapy	Day 1	Clopidogrel 300mg/ day + aspirin 100-300mg/ day
	Day 2 - Day21±2	Clopidogrel 75mg/ day + aspirin 100mg/ day
	Day22±2 - Day 90	Clopidogrel 75mg/ day + aspirin placebo
Standard antiplatelet therapy	Day 1	Aspirin 100-300mg/ day + clopidogrel placebo
	Day 2 - Day 90	Aspirin 100mg/ day + clopidogrel placebo
Immediate intensive statin therapy	Day 1 - Day21±2	Atorvastatin 80mg/ day
	Day22±2 - Day 90	Atorvastatin 40mg/ day
Delayed intensive statin therapy	Day 1 - Day3	Atorvastatin placebo
	Day 4 - Day21±2	Atorvastatin 40mg/ day + atorvastatin placebo
	Day22±2 - Day 90	Atorvastatin 40mg/ day

Research design (drawing)

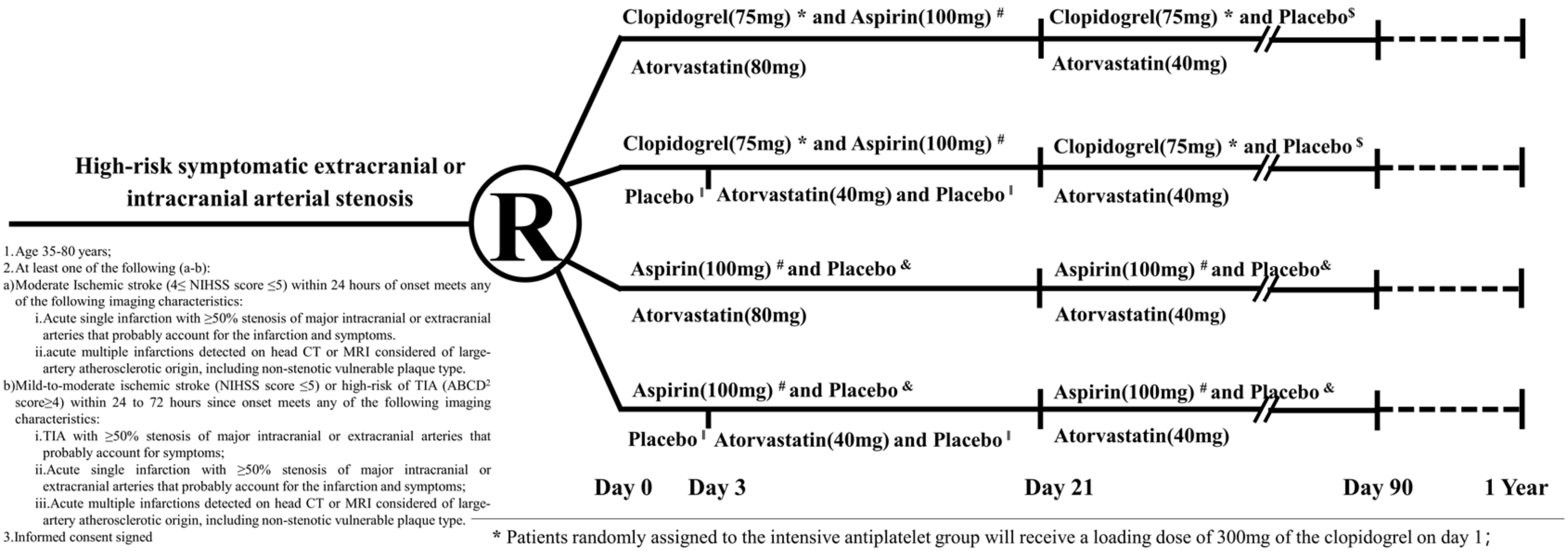

* Patients randomly assigned to the intensive antiplatelet group will receive a loading dose of 300mg of the clopidogrel on day 1;
# Patients in all 4 groups will receive an open-label dose of 100-300mg of the aspirin on day 1;
$ Placebo for aspirin; ǀ Placebo for atorvastatin; & Placebo for clopidogrel.

Follow-up plan

Subjects will receive a face-to-face visit at baseline, Day7±2, Day14 (or hospital discharge) and Day90 ± 7, and a telephone visit at the 12th month±14 days after randomization. In addition, patients will be interviewed when new neurologic symptoms or suspicious events occur, including worsening of index ischemic events, new transient or persistent neurological symptoms.

Planned Analyses

The analyses that are detailed in this SAP will be performed only when the database has been locked, all protocol violators identified, and treatment allocations have been unblinded. Membership of the Full Analysis and Per Protocol populations will be determined using the rules set out in this SAP. At a date to be agreed within the project team, a data look will be performed. This will involve production of all data displays on a subset of the data using dummy treatment codes. These are produced purely as an aide to the pre-programming of the study and no unblinding will occur.

Interim Analyses

No interim analyses are planned for this study. However, a Data and Safety Monitoring Board (DSMB) is in place to ensure the safety of patients in the study. An independent statistician will prepare unblinded summary tables of SAEs, selected demographic data and patients exposure data and these will be examined by the DSMB. These tables will be provided to the DSMB at regular intervals. If the tables give rise to safety concerns for any treatment, the DSMB may recommend that the trial should be modified or stopped prematurely. The Steering Committee will, in conjunction with the Sponsor, decide whether to act on this recommendation. Further discussion of these safety tabulations is provided in a specific study protocol.

6. Sample size estimates

The minimal sample size for the trial is determined by the necessity that a clinically meaningful difference in effectiveness between treatment and control groups has to be detected. Based on previous studies, the risk of new stroke during 90 days is presumed to be 11.5% in the group with standard antiplatelet therapy (with half delayed intensive statin therapy and half early high-intensity statin therapy) and 11.5% in the delayed intensive statin therapy group (with half standard antiplatelet therapy and half dual antiplatelet therapy) and 13% in the group with standard antiplatelet therapy plus delayed intensive statin therapy, intensive antiplatelet therapy and immediate intensive statin therapy can reduce this risk by 22%, and the effects of intensive antiplatelet and lipid lowering therapy will be similar and additive. With a sample size of 6100 subjects, a two-sided α of 0.05 and 5% loss to follow up, we will have 97% power to detect that the risk is decreased by 35% in the group with dual antiplatelet therapy plus immediate intensive statin therapy compared to standard antiplatelet therapy plus delayed intensive therapy, and 80% power to detect that the risk is reduced by 20% by intensive antiplatelet therapy compared to standard antiplatelet therapy, and immediate intensive statin compared

to delayed intensive statin therapy, respectively.

Table1 For different risk rates (12% to 14%) of the results in the control group, the sample size required for observing intensive antiplatelet combining intensive statin therapy

Power	A single set of sample size (no expulsion)	A single set of sample size (5% expulsion rate)	Four groups of sample size	The stroke recurrence rate in treatment group	The stroke recurrence rate in control group	Relative risk
0.966	1448	1525	6100	0.078	0.120	0.65
0.975	1448	1525	6100	0.085	0.130	0.65
0.985	1448	1525	6100	0.091	0.140	0.65
0.824	1448	1525	6100	0.096	0.130	0.74

Table2 For different risk rates (12% to 14%) of the results in the control group, the sample size required for the marginal effect analysis of observed intensive antiplatelet combining intensive statin therapy

Power	A single set of sample size (no expulsion)	A single set of sample size (5% expulsion rate)	Four groups of sample size	The stroke recurrence rate in treatment group	The stroke recurrence rate in control group	Relative risk
0.807	2896	3050	6100	0.0930	0.1157	0.80

Explanations of data sources on recurrence risk and the risk of stroke recurrence after treatment:

At present, evidence in early risks of recurrent stroke in high-risk symptomatic intracranial or extracranial artery stenosis patients is still lacking. The data currently available for reference include: a). In the studies of WASID and SAMMPRIS, the recurrence risk of stroke in the medication treatment group with symptomatic intracranial artery stenosis (stenosis rate > 70%) were 21.9% and 12.6% at 1 year, respectively [1,2]. The recurrence risk of 12.6% at 1 year in SAMMPRIS research was based on the combination of dual anti-platelet therapy, intensive lipid-lowering and blood-pressure-lowering therapy. Therefore, for the recurrence risk of the standard therapy group, 13% was a conservative figure in our study [3]. b) The subgroup study of the CHANCE Trial has revealed a recurrence risk of 12.5% in patients with intracranial arterial stenosis (stenosis rate > 50%) at day 90[4]. In the CHANCE trial, patients with intracranial arterial stenosis associated with multiple infarcts had a recurrent stroke risk of a striking number of 18% at 90 days [5]. Therefore, as the recurrence risk of the standard therapy group, 13% was a conservative data in our study.

Concerning that dual anti-platelet therapy reduces the risk of recurrence of 22%, the CHANCE study confirmed that dual anti-platelet therapy decreased the risk of stroke recurrence by 90 days for TIA and minor stroke by 32% compared with anti-platelet monotherapy [6]. In the CHANCE subgroup, compared with anti-platelet monotherapy, dual anti-platelet therapy decreased the relative risk of stroke

recurrence by 21% in patients with intracranial arterial stenosis [4], and by 50% in patients with the multiple cerebral infarction [7]. Therefore, with dual anti-platelet therapy, our estimate of a relative reduction of 22% in recurrent stroke risk was a conservative data in our study.

Concerning the source of evidence for intensive lipid-lowering combined with intensive antiplatelet therapy decreases the risk of stroke by 35%, a comparative study of SAMMPRIS and WASID before and after pointed out that the risks of recurrent stroke were 21.9% and 12.6% in the intensive drug therapy group and anti-platelet monotherapy group at 1 year, respectively. The risk was decreased by about 50%. Therefore, the estimated reduction of recurrence risk by 35% was a conservative data in our study. [2].

7. Analysis populations

Full Analysis Set (FAS):

The full analysis set is the main efficacy evaluation population of this study, and all valid variables will be analyzed by FAS. According to the basic principle of intention-to-treat analysis (ITT), all subjects randomized into groups and those with more than one medication record and efficacy evaluation will be included in the full analysis set.

Per Protocol Set (PPS)

Includes all subjects who complete the protocol and have no major deviation of the trial. The exact definition of a major deviation of the program will be finalized at the time of data review and may generally include the following situations (but not limited to these cases): failing to meet the main inclusion criteria, serious disturbance of drug efficacy after treatment, poor compliance, exceeding the time window of following-up and so on. PPS is the secondary analysis of the effectiveness of the crowd, but if the results are inconsistent with the whole analysis set, detailed analysis of the inconsistent results are needed.

Safety Set (SS)

A safety data set is defined as a subject that receives drug treatment at least once. In the entire safety analysis, patients with incorrect treatment (for example, randomized to the standard antiplatelet combining delayed intensive statin therapy groups were given enhanced antiplatelet therapy) would be assigned to the actual treatment group.

8. Treatment comparisons

The treatment comparisons of interest in this study are:

1) To evaluate the efficacy and safety of intensive antiplatelet therapy versus standard antiplatelet therapy in reducing the risk of new stroke at 90 days in acute mild ischemic stroke or high-risk TIA patients attributed to extracranial or intracranial atherosclerosis;

2) To evaluate the efficacy and safety of immediate intensive statin therapy (atorvastatin 80mg/d) versus delayed intensive statin therapy (atorvastatin 40mg/d) in reducing the risk of new stroke at 90 days in acute mild ischemic stroke or high-risk TIA patients attributed to extracranial or intracranial atherosclerosis;
3) To evaluate the efficacy and safety of intensive antiplatelet combined with immediate intensive statin therapy (atorvastatin 80mg/d) versus standard antiplatelet combined with delayed intensive statin therapy (atorvastatin 40mg/d) in reducing the risk of new stroke at 90 days in acute mild ischemic stroke or high-risk TIA patients attributed to extracranial or intracranial atherosclerosis.

9. General considerations for data analyses

All programming will be performed using SAS Version 9.4. All analysis output will use the following treatment group naming conventions and treatment order: intensive antiplatelet therapy, standard antiplatelet therapy, immediate intensive statin therapy and delayed intensive statin therapy.

All statistics were two sided with a *P*<0.05 considered significant, and the confidence interval was reliable two sided with 95%.

Multicenter Studies

Centers with less than 20 patients will be pooled with larger centers within the same geographic region so that centers are of a reasonable size for the purpose of the statistical analyses. This process will be performed and finalized before the treatment codes are unblinded.

In multicenter randomized controlled clinical study, there were some different effect in different center due to different baseline, clinical practice or other factor, therefore, center effect analysis was required. Stratified analysis was used to exclude the mixed effect of results caused by center effect: each center was served as a stratum, calculating the HR by Cox proportional hazards model.

Examination of Subgroups

1) To evaluate the efficacy of intensive antiplatelet therapy versus standard antiplatelet therapy, immediate intensive statin therapy (atorvastatin 80mg/d) versus delayed intensive statin therapy (atorvastatin 40mg/d), and intensive antiplatelet combined with immediate intensive statin therapy (atorvastatin 80mg/d) versus standard antiplatelet combined with delayed intensive statin therapy (atorvastatin 40mg/d) in reversing intracranial atherosclerotic artery stenosis and stabilizing the atherosclerotic vulnerable plaque in the high resolution MRI subgroup.
2) To evaluate the efficacy of intensive antiplatelet therapy versus standard antiplatelet therapy for 90 days on the incidence of the primary outcome in different subgroups:

- Subjects aged > 65 years vs. those aged ≤65 years.
- Female vs. male patients.
- Those with NIHSS score 4-5 vs. those with NIHSS score ≤3 at admission.

- Those randomized within 24 hours of onset vs. those randomized between 24 and 72 hours since onset.
- Those with ischemic stroke/TIA related to extracranial artery atherosclerosis vs. those related to intracranial artery atherosclerosis.
- Those with intracranial stenosis vs. those without intracranial stenosis.
- Those with extracranial stenosis vs. those without extracranial stenosis.
- Those with multiple infarctions vs. those with single infarction vs. those without infarction.
- Those with severe stenosis (≥70%) vs. those with moderate stenosis (50%-69%).
- Those with hypertension vs. those who are normotensive.
- Diabetic patients vs. nondiabetics.
- Those with dyslipidaemia vs. without dyslipidaemia.
- Those with atherogenic dyslipidaemia (HDL-C<40 mg/dL and TG>200 mg/dL) vs. without atherogenic dyslipidaemia.
- Those with statin therapy within 1 month before randomization vs. without statin therapy.

In addition, relevant subgroups will be examined for genetic variability and biomarker characteristics.

3) To evaluate the efficacy of immediate intensive statin therapy (atorvastatin 80mg/d) versus delayed intensive statin therapy (atorvastatin 40mg/d) for 90 days on the incidence of the primary outcome in subgroups which is the same as above.

Multiple Comparisons and Multiplicity

A single primary efficacy variable has been defined for this study, with all other efficacy variables identified as secondary or other. Comparison of intensive antiplatelet therapy versus standard antiplatelet therapy, immediate intensive statin therapy versus delayed intensive statin therapy, intensive antiplatelet combined with immediate intensive statin therapy versus standard antiplatelet combined with delayed intensive statin therapy were conducted under separate hypotheses; therefore, there are no requirements to adjust for multiple comparisons or multiple endpoints within this study.

10. Data handling conventions

Premature Withdrawal and Missing Data

1) Loss to follow-up

Investigators should try their best to keep contact with every patient, making sure the reason of loss to follow-up and their health situation. All CRFs of missed patients should be recorded until the last follow-up visit.

2) Quit from study

Those who quit the study should not be included again. The randomization number and study drugs of this subject should not be used again. Randomized patients must not be replaced. Investigators should confirm the withdrawal along with the monitoring committee. Subjects who were randomized and had one or more doses recorded were required to complete the calendar visits according to the protocol.

Event Rates

The number of people of events should be recorded in detail and showing the event rate in 90 days of each treatment group in summary statement.

The event rate for each treatment group will be calculated as: the sum of number of event for all the patients / the number of patients enrolled in this group.

Time to Event Analysis

Differences between treatments in the risk of recurrent stroke event and clinical vascular events during maximum 90-day follow-up were assessed using standard Kaplan-Meier time-to-event approaches. The time to the first event was used in the model when there were multiple events of the same type. Patients were considered censored at the time of study termination or death if there were no events occurred during the study.

11. Study Population

Disposition of Patients

The number of patients in each analysis population will be presented, patients to be excluded from the Per Protocol population will be listed, and the total number of patients attending each clinic visit will also be summarized by treatment group.

The number of patients randomized, completed and prematurely withdrawn from the study will be presented for each treatment group. The primary reasons for withdrawal both prior to and post randomization will also be presented.

A data display listing and summary of deviations from the inclusion/exclusion criteria will be presented for all patients who were either entered or randomized into the trial.

Protocol Deviations

Patient data will be examined for evidence of protocol violators in order to assess how well the protocol was followed. Inclusion and exclusion criteria are detailed in the study protocol.

Patients who commit protocol violations will be included in the FAS Population but excluded from the Per Protocol Population. These protocol violations will be shown in a listing. Patients can either be full or partial protocol violators. A full protocol violator is completely excluded from the Per Protocol Population. A partial protocol violator has only some data excluded. For patients who violated the protocol during the treatment period due to unpermitted changes in the medication or prohibited concurrent medication, the analysis will only use data recorded prior to the violation. For all violations

which reference the treatment period, the treatment start date will be used as the reference date.

A listing of all possible protocol violators will be produced for clinical review. The final list of patients who are protocol violators and are therefore excluded from the Per-Protocol population will be agreed by the study team.

Demographic and Baseline Characteristics

Demographic, Medical, histories and baseline characteristics information will be listed and summarized for patients in each treatment group based on the FAS population.

Vital signs including supine systolic blood pressure, diastolic blood pressure, and heart rate will also be listed and summarized in each treatment group.

The continuous data followed normal distribution will be presented as mean and standard deviation, and the continuous data followed skewness distribution will be presented as median and interquartile range; categorical data will be presented as n (%). T-test or Wilcoxon rank sum test will be used for comparison between two continuous data, and Chi-squared tests or Fisher exact test will be used for comparison between two categorical data.

12. Efficacy Analyses

Primary Efficacy Analysis

The primary endpoint is stroke (both hemorrhagic and ischemic stroke). FAS will be the primary population for efficacy analyses. PPS will be used as secondary population for the efficacy analyses. If the results in the PPS population are inconsistent with the FAS population, detailed analysis of the inconsistent results is required.

Main Model

The time to stroke (both hemorrhagic and ischemic stroke) reported during the 90-day treatment period for the ITT Population will be summarized by treatment group using Kaplan-Meier estimates. The hazard ratio for the treatment comparison will be derived using a Cox proportional hazards model, including the pooled study center as a random effect. The hazards ratios (HR) with 95% CI will be reported. This will also be presented graphically on a Kaplan-Meier plot. The log-rank test will be used to evaluate the statistical significance of the treatment effect.

Interactions with Subgroups

To evaluate the efficacy of intensive antiplatelet therapy versus standard antiplatelet therapy, immediate intensive statin therapy (atorvastatin 80mg/d) versus delayed intensive statin therapy (atorvastatin 40mg/d), and intensive antiplatelet combined with immediate intensive statin therapy (atorvastatin 80mg/d) versus standard antiplatelet combined with delayed intensive statin therapy (atorvastatin 40mg/d) in reversing intracranial atherosclerotic artery stenosis and stabilizing the atherosclerotic vulnerable plaque in the high resolution MRI subgroup.

To evaluate the efficacy of intensive antiplatelet therapy versus standard antiplatelet therapy for 90 days on the incidence of the primary outcome in different subgroups:

- Subjects aged > 65 years vs. those aged ≤65 years.
- Female vs. male patients.
- Those with NIHSS score 4-5 vs. those with NIHSS score ≤3 at admission.
- Those randomized within 24 hours of onset vs. those randomized between 24 and 72 hours since onset.
- Those with ischemic stroke/TIA related to extracranial artery atherosclerosis vs. those related to intracranial artery atherosclerosis.
- Those with intracranial stenosis vs. those without intracranial stenosis.
- Those with extracranial stenosis vs. those without extracranial stenosis.
- Those with multiple infarctions vs. those with single infarction vs. those without infarction.
- Those with severe stenosis (≥70%) vs. those with moderate stenosis (50%-69%).
- Those with hypertension vs. those who are normotensive.
- Diabetic patients vs. nondiabetics.
- Those with dyslipidaemia vs. without dyslipidaemia.
- Those with atherogenic dyslipidaemia (HDL-C<40 mg/dL and TG>200 mg/dL) vs. without atherogenic dyslipidaemia.
- Those with statin therapy within 1 month before randomization vs. without statin therapy.

In addition, relevant subgroups will be examined for genetic variability and biomarker characteristics.

To evaluate the efficacy of immediate intensive statin therapy (atorvastatin 80mg/d) versus delayed intensive statin therapy (atorvastatin 40mg/d) for 90 days on the incidence of the primary outcome in subgroups which is the same as above.

Secondary Efficacy Analyses

Combined vascular events: Strokes (ischemic or hemorrhagic), myocardial infarction, and cardiovascular death

Combined vascular events: Strokes (ischemic or hemorrhagic), myocardial infarction, and cardiovascular death will be compared by Chi-squared tests. The difference of the incidence rates between two groups with 95% CI will be reported. Cox proportional risk model including the pooled study center as a random effect will be used to calculate HR and the 95% confidence interval and the log-rank test would be used to evaluate the efficacy;

Ischemic stroke

The difference of the rates between two groups with 95% CI will be reported. Cox proportional risk

model will be used to calculate HR and the 95% confidence interval and the log-rank test would be used to evaluate the efficacy;

Transient ischemic attack (TIA)

The difference of the rates between two groups with 95% CI will be reported. Cox proportional risk model including the pooled study center as a random effect will be used to calculate HR and the 95% confidence interval and the log-rank test would be used to evaluate the efficacy;

Severity of stroke or TIA on an ordinal scale: (a six-level ordered category scale combined vascular events with mRS score: fatal stroke (stroke with subsequent death), severe stroke (stroke followed by mRS of 4-5), moderate stroke (stroke followed by mRS of 2-3), mild stroke (stroke followed by mRS of 0-1), TIA, and no stroke/TIA)

The difference of the rates between two groups with 95% CI will be reported. Ordinal logistic regression test including the pooled study center as a random effect will be used to calculate common odds ratio (OR) and the 95% confidence interval;

Myocardial infarction

The difference of the rates between two groups with 95% CI will be reported. Cox proportional risk model including the pooled study center as a random effect will be used to calculate HR and the 95% confidence interval and the log-rank test would be used to evaluate the efficacy;

Vascular death

The difference of the rates between two groups with 95% CI will be reported. Cox proportional risk model including the pooled study center as a random effect will be used to calculate HR and the 95% confidence interval and the log-rank test would be used to evaluate the efficacy;

All-cause death

The difference of the rates between two groups with 95% CI will be reported. Cox proportional risk model including the pooled study center as a random effect will be used to calculate HR and the 95% confidence interval and the log-rank test would be used to evaluate the efficacy;

Poor functional outcome (mRS score 2-6)

The difference of the rates between two groups with 95% CI will be reported. Logistic regression test will be used to calculate OR and the 95% confidence interval;

Poor quality of life (EQ-5D scale index score≤0.5)

The difference of the rates between two groups with 95% CI will be reported. Logistic regression test will be used to calculate OR and the 95% confidence interval;

Early neurological deficits (NIHSS score increase of no less than 2points within 7 days)

The difference of the rates between two groups with 95% CI will be reported. Logistic regression test will be used to calculate OR and the 95% confidence interval;

All statistical data will be inspected with two-sided P<0.05 as statistically significant.

13. Safety Analyses

The overall of safety assessment is all used test medications and the safety follow-up case should be recorded at least once. The safety evaluation data includes adverse reactions observed during the trial and changes in laboratory data before and after treatment.

Primary safety outcome

- Moderate to severe bleeding

Secondary safety outcomes

- Intracranial hemorrhage
- Hepatotoxicity: ALT or AST > 3 times the upper limit of normal value;
- Muscle toxicity: CK > 10 times the upper limit of normal value, or the presence of muscle pain, myopathy, or rhabdomyolysis.
- Death
- Other AEs / SAEs

Safety evaluation will be analyzed using safety data set.

- Moderate to severe bleeding, intracranial hemorrhage, and overall mortality will be calculated using the Kaplan-Meier curve to simulate the 3-month cumulative risk, and the Cox proportional hazards model to calculate the HR and 95% confidence interval.
- For hepatotoxicity, muscle toxicity and other adverse events and serious adverse events, the cases which was normal before the treatment and abnormal after the treatment would be mainly analyzed and listed, in order to the comparison of differences before and after treatment.

Adverse Events

Adverse events (AEs) will be coded using the MedDRA coding dictionary (Version 6.0 or a later release) and grouped by system organ class (as detailed in the study protocol). Separate data display listings and summaries will be presented for adverse events that start prior to first dose of study medication (pre-treatment), whilst on study medication (during treatment) and after the last dose of study medication (post-treatment).

Within each treatment group, the number and percentage of patients experiencing an AE will be summarized by system organ class and preferred term and Chi-square test or Fisher's Exact test will be used to compare the number of each grouped AE event between treatment groups. In addition, a separate summary will be provided for AEs experienced by more than 5% of patients in either of the treatment groups.

Serious Adverse Events

Summary tables and data displays will be provided for serious adverse events (as detailed in the study protocol). In addition, all serious AE's will be documented in a case narrative format in the clinical study report. The number of events occurring over the treatment period will be summarized and Chi-square test or Fisher's Exact test will be used to compare the number of events between treatment groups.

14. References

1. Chimowitz MI, Lynn MJ, Derdeyn CP, et al. Stenting versus aggressive medical therapy for intracranial arterial stenosis. N Engl J Med. 2011 Sep 15;365(11): 993-1003.
2. Chaturvedi S, Turan TN, Lynn MJ, et al. Do Patient Characteristics Explain the Differences in Outcome Between Medically Treated Patients in SAMMPRIS and WASID? Stroke. 2015 Sep;46(9)：2562-7.
3. Waters MF, Hoh BL, Lynn MJ, et al. Factors Associated with Recurrent Ischemic Stroke in the Medical Group of the SAMMPRIS Trial. JAMA Neurol. 2016;73(3): 308-15.
4. Liu L, Wong KS, Leng X, et al. Dual antiplatelet therapy in stroke and ICAS: Subgroup analysis of CHANCE. Neurology, 2015,85(13):1154-1162.
5. Pan Y, Meng X, Jing J, et al. Association of multiple infarctions and ICAS with outcomes of minor stroke and TIA. Neurology. 2017; 88:1081-1088.
6. Wang Y, Wang Y, Zhao X, et al. Clopidogrel with aspirin in acute minor stroke or transient ischemic attack. N Engl J Med. 2013 Jul 4;369(1): 11-9.
7. Jing J, Meng X, Zhao X, et al. Dual antiplatelet therapy in transient ischemic attack and minor stroke with different infarction patterns: Subgroup analysis of chance randomized clinical trial. JAMA Neurology. 2018.

SAP changes version 1.0 (Sep 17th, 2018) to version 2.0 (Jun 6th, 2020)

SAP V1.0	SAP V2.0
Follow-up plan<br>Subjects will receive a face-to-face visit at baseline, Day7±2, Day14 (or hospital discharge), Day 21±2 and Day90 ± 7, and telephone visits at the 6th month±14 days and 12th month±14 days after randomization. In addition, patients will be interviewed when new neurologic symptoms or suspicious events occur, including worsening of index ischemic events, new transient or persistent neurological symptoms.	***Follow-up plan***<br>Subjects will receive a face-to-face visit at baseline, Day7±2, Day14 (or hospital discharge) and Day90 ± 7, and a telephone visit at the 12th month±14 days after randomization. In addition, patients will be interviewed when new neurologic symptoms or suspicious events occur, including worsening of index ischemic events, new transient or persistent neurological symptoms.
