# Supplementary material for: Immediate- or Delayed-Intensive Statin in Acute Cerebral Ischemia: The INSPIRES Randomized Clinical Trial
Source: JAMA Neurol. 2024 May 28;81(7):741–51. doi: 10.1001/jamaneurol.2024.1433 (PMC11134282; doi:10.1001/jamaneurol.2024.1433)
Supplement: Supplement 3. — eAppendix. eFigure 1. Hazard Ratio for Stroke in Prespecified Subgroups eFigure 2. Odds Ratio for Poor Functional Outcome in Prespecified Subgroups eFigure 3. Subgroup Analysis for Stroke by Center eTable 1. Baseline Characteristics of the Patients eTable 2. Concomitant Treatment Within 90 Days eTable 3. Efficacy and Safety Outcomes in Per-Protocol Population eTable 4. Number of Patients With Adverse Events by System Organ Class (Excluding Strokes) Up to 3-Month Visit eTable 5. Number of Patients With Serious Adverse Events By System Organ Class (Excluding Strokes) Up To 3-Month Visit eTable 6. Number of Patients With Adverse Events or Serious Adverse Events Leading to Premature Permanent Drug Discontinuation by System Organ Class Up to 3-Month Visit eTable 7. mRS Outcome Adjusted by the Baseline Proportion of TIA and Infarction and Baseline NIHSS Score [file jamaneurol-e241433-s003.pdf]

Supplementary Online Content

Gao Y, Jiang L, Pan Y, et al; INSPIRES Investigators. Immediate- or delayed-intensive statin in acute cerebral ischemia: the INSPIRES randomized clinical trial. *JAMA Neurol*. Published online May 28, 2024. doi:10.1001/jamaneurol.2024.1433

eAppendix.

eFigure 1. Hazard Ratio for Stroke in Prespecified Subgroups

eFigure 2. Odds Ratio for Poor Functional Outcome in Prespecified Subgroups

eFigure 3. Subgroup Analysis for Stroke by Center

eTable 1. Baseline Characteristics of the Patients

eTable 2. Concomitant Treatment Within 90 Days

eTable 3. Efficacy and Safety Outcomes in Per-Protocol Population

eTable 4. Number of Patients With Adverse Events by System Organ Class (Excluding Strokes) Up to 3-Month Visit

eTable 5. Number of Patients With Serious Adverse Events By System Organ Class (Excluding Strokes) Up To 3-Month Visit

eTable 6. Number of Patients With Adverse Events or Serious Adverse Events Leading to Premature Permanent Drug Discontinuation by System Organ Class Up to 3-Month Visit

eTable 7. mRS Outcome Adjusted by the Baseline Proportion of TIA and Infarction and Baseline NIHSS Score

This supplementary material has been provided by the authors to give readers additional information about their work.

© 2024 Gao Y et al. *JAMA Neurology.*

eAppendix.

Listing of committees in the INSPIRES Trial

Steering Committee:

Yilong Wang MD, PhD., Yongjun Wang MD., S. Claiborne Johnston MD, PhD., Pierre Amarenco MD., Philip M. W. Bath D.Sc., Xingquan Zhao MD, PhD., Liping Liu MD, PhD., and investigators from the participating hospitals.

Executive Committee:

Yilong Wang MD, PhD., Ying Gao MD., Jing Jing MD, PhD., Chunjuan Wang MD, PhD., Weiqi Chen MD., Xia Meng MD, PhD., Jinxi Lin MD, PhD., Lingling Jiang PhD., Yingying Yang MD., Tingting Wang MD., Shangrong Han MD.

Data and Safety Monitoring Board:

David Wang BA, MD., Hao Li PhD., Haifeng Li MD, PhD.

Clinical Event Adjudication Committee:

James Wang MD, PhD., Yuming Xu MD, PhD., Kehui Dong MD, PhD., Xiaoling Liao MD, PhD., Hui Qu MD, PhD.

Clinical Coordinating Center:

Ying Gao MD., Jing Jing MD, PhD., Chunjuan Wang MD, PhD., Xia Meng MD, PhD., Jinxi Lin MD, PhD., Yingying Yang MD., Tingting Wang MD., Shangrong Han MD., Li Liu MM., Jie Song MM., Shuting Liu, Xiaoyu Che, Xianhong Liang, Shangzhi Li, Nan Qi, Xiaolei Chen, Zhiyuan Ji (SMO), Jianying Li (SMO), Chenhui Liu MM., Jingtao Pi MM., Yu Tian MM., Nan Wang MM., Zhengyang Li MM., Biyang Luo MM., Fanfang Yue MM., Lei Guo MM., Kun Hu MM., Xi Zhong MM., Jiawei Lu, Long Wang, Jiandong Yu, Xiaowu Zhang, Mengyuan Zhou MD., Yiyi Chen MD..

Statistical and Data Management Center:

Yuesong Pan PhD., Hongyi Yan MM., Aoming Jin PhD., Mengxing Wang MM.

Drug Distribution Center: Haibo Wu (CRO)

Independent Medical Monitor: Haibo Wu (CRO)

© 2024 Gao Y et al. *JAMA Neurology.*

Study design and treatment allocation

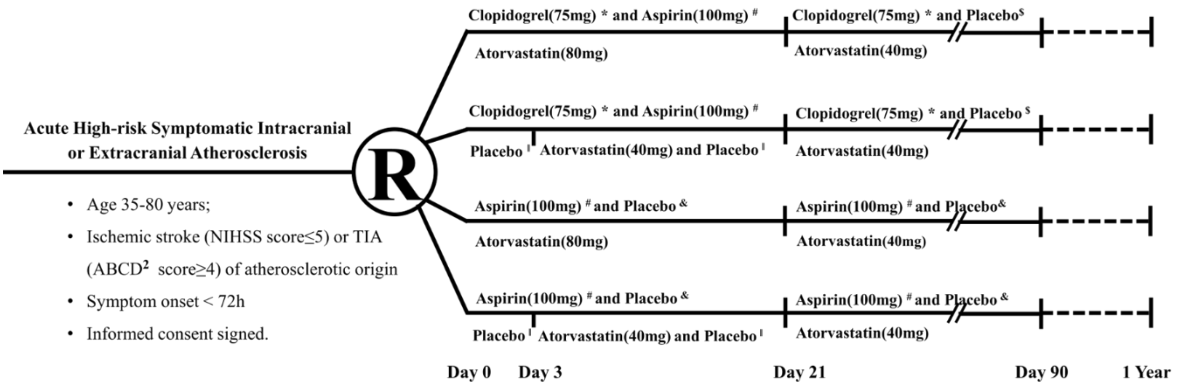

* Patients randomly assigned to the intensive antiplatelet group will receive a loading dose of 300mg of the clopidogrel on day 1 ;
# Patients in all 4 groups will receive an open-label dose of 100-300mg of the aspirin on day 1;
$ Placebo for aspirin; ¦ Placebo for atorvastatin; & Placebo for clopidogrel.

© 2024 Gao Y et al. *JAMA Neurology*.

Inclusion and exclusion criteria

Inclusion criteria:

1.	Age 35-80 years;
2.	At least one of the followings (a-b):<br>a) Mild ischemic stroke (NIHSS score 4-5) within 24 hours after onset and either of the following imaging characteristics:<br>i. Acute single infarction with ≥50% stenosis of a major intracranial or extracranial artery that likely accounts for the infarction and clinical presentation.<br>ii. Acute multiple infarctions documented by head CT or MRI, attributed to large-artery atherosclerosis, including non-stenotic vulnerable plaques.<br>b) Mild ischemic stroke (NIHSS score ≤5) or high-risk TIA ($ABCD^2$ score≥4) within 24 to 72 hours after onset and meet any of the following imaging characteristics:<br>i. TIA with ≥50% stenosis of a major intracranial or extracranial artery that likely accounts for the clinical presentation.<br>ii. Acute single infarction with ≥50% stenosis of a major intracranial or extracranial artery that likely accounts for the infarction and clinical presentation.<br>iii. Acute multiple infarctions documented by head CT or MRI, attributed to large-artery atherosclerosis, including non-stenotic vulnerable plaques.
3.	Written informed consent.

Exclusion criteria:

1.	Presumed cardioembolic stroke or TIA (e.g. atrial fibrillation, heart valve prosthesis, atrial myxoma, endocarditis, etc.);
2.	Other determined etiology of stroke or TIA (e.g. aortic dissection, cervico-cerebral artery dissection, vasculitis, vascular malformation, Moyamoya disease/syndrome, fibromuscular dysplasia, etc.);
3.	Non-vascular neurological diseases (e.g. intracranial tumor, multiple sclerosis, etc.);
4.	Index infarction affects >50% of a cerebral lobe (e.g. parietal, frontal, occipital);
5.	Hemorrhagic transformation after onset;

© 2024 Gao Y et al. *JAMA Neurology.*

6.	Contraindications to clopidogrel, aspirin or atorvastatin: a) History of hypersensitivity; b) Severe heart failure (New York Heart Association classification: III- IV) or asthma; c) Coagulation disorder or systemic bleeding; d) History of drug-induced hematologic or hepatic abnormalities; e) Leukopenia (< 2×10^9/L) or thrombocytopenia (<100×10^9/L); f) Active liver disease; g) Pregnancy or lactation period
7.	Pre-existing disability with modified Rankin Scale score >2；
8.	Intra-arterial or intravenous thrombolysis, or endovascular therapy after onset;
9.	Defibrinogen therapy (e.g. defibrase and lumbrokinase), anticoagulation therapy (e.g. argatroban), or antiplatelet therapy (e.g. ticagrelor, tirofiban) except for clopidogrel and aspirin after onset;
10.	Creatine kinase > 5 times the upper limit of normal value of onset;
11.	Drug use related to statin metabolism within 14 days before randomization (e.g. immune-suppressive drugs, antifungal agents, fibrates);
12.	Severe hepatic insufficiency (alanine transaminase or aspartate transaminase > 2 times the upper limit of normal value) or renal insufficiency (creatinine > 1.5 times the upper limit of normal value or glomerular filtration rate < 40 ml/min/1.73 m^2);
13.	Dual antiplatelet therapy with aspirin and clopidogrel within 14 days before randomization*;
14.	High-intensity statin therapy within 14 days before randomization (e.g. atorvastatin ≥ 40mg/d, rosuvastatin ≥ 20mg/d);
15.	History of intracranial hemorrhage (e.g. intracerebral or subarachnoid hemorrhage);
16.	Gastrointestinal bleeding or major surgery within 90 days;
17.	History of intracranial or extracranial angioplasty;
18.	Planned long-term use of antiplatelet drugs or non-steroidal anti-inflammatory drugs except for study drugs;
19.	Planned surgery or revascularization that may need to stop taking the study drugs within the next 90 days;
20.	Anticipated life expectancy < 90 days;
21.	Pregnant women, or patients of child-bearing potential with neither using birth control nor

© 2024 Gao Y et al. *JAMA Neurology*.

	pregnancy test records;
22.	Currently participating in any other investigational drug or device study;
23.	Unable to complete the follow-up (e.g. dementia, alcoholism, substance abuse, severe mental disease).

© 2024 Gao Y et al. *JAMA Neurology*.

Definitions of cardiac-cerebral vascular events

Event	Definition
**Stroke**	A sudden onset of focal or global brain, spinal cord or retinal vascular damage, resulting in symptoms and signs of acute nervous system defects, which is associated with cerebral circulation disorders.
**Ischemic Stroke**	Acute focal cerebral or retinal infarction meeting any of the following conditions:<br>(1) Recurrent stroke: clinical signs or radiological evidence of acute onset of new focal neurological damage lasting longer than 24 hours, excluding other non-ischemic etiologies (such as brain infections, brain injuries, brain tumors, seizures, severe metabolic diseases, degenerative diseases of the nervous system and side effects of drugs);<br>(2) TIA with infarctions: acute cerebral or retinal ischemic events, excluding other non-ischemic etiologies, focal symptoms or signs sustaining less than 24 hours, but with radiological evidence of new infarction;<br>(3) Progressive stroke: the worsening of pre-existing symptoms of vascular origin ischemic stroke (i.e. NIHSS increased ≥ 4 based on primary ischemic stroke, excluding the hemorrhagic transformation after infarction or symptomatic intracranial hemorrhage) persisting for more than 24 hours, with or without deterioration of ischemic lesions on MRI or CT. Etiologic typing is based on the TOAST criteria.
**Transient Ischemic Attack**	Neurologic deficit caused by sudden focal brain or retinal ischemia that can fully recover, lasting less than 24 hours, with no evidence of new cerebral infarction on imaging (CT or MR). Other non-ischemic causes (such as brain infections, brain injuries, brain tumors, epilepsy, severe metabolic diseases, or degenerative neurological diseases) are excluded.

© 2024 Gao Y et al. *JAMA Neurology.*

**Hemorrhagic Stroke**	Hemorrhagic stroke is defined as acute neurological dysfunction of the focal or whole brain or spinal cord caused by non-traumatic brain parenchymal, intraventricular, and subarachnoid hemorrhage.
**Hemorrhagic Transformation after Cerebral Infraction**	Any non-traumatic extravascular hemorrhage in acute / subacute infarcts, which could cause related neurological symptoms (symptomatic) or non-neurological symptoms (asymptomatic). Among them:<br>(1) Ischemic stroke transformed into symptomatic hemorrhagic stroke: The following two conditions must be met at the same time:<br>a. Imaging evidence (CT or MRI) of extravascular hemorrhage in the infarct area;<br>b. Symptoms are related to hemorrhagic transformation. The hemorrhagic transformation must be able to partially explain the clinical manifestations of the patient's neurological performance, such as:<br>i). Symptoms cannot be fully explained by infarct size and location<br>ii). Clinical deterioration referring to an increase of 4 points or more in NIHSS score after the initial ischemic event, or death, which is caused by hemorrhagic transformation;<br>iii). Clinical symptoms caused by volume effect secondary to hemorrhagic transformation;<br>(2) Ischemic stroke transformed into asymptomatic hemorrhagic stroke: The following two conditions must be met at the same time:<br>a. Imaging evidence (CT or MRI) of extravascular hemorrhage in the infarct area;<br>b. Hemorrhagic transformation does not cause symptoms, or cause symptoms with an increase of less than 4 points in NIHSS score

© 2024 Gao Y et al. *JAMA Neurology*.

	after the initial ischemic event.
**Myocardial Infarction**	Acute myocardial infarction diagnosed by the third universal definition.[1]<br><br>If there is clinical evidence of myocardial necrosis consistent with acute myocardial ischemia (MI), acute MI should be diagnosed. It can be diagnosed if it meets any of the following criteria:<br><br>(1) A rise and/ or fall of cardiac biomarkers (preferably troponin [cTn]) values with at least one value above the 99th percentile URL, and any of the followings is required:<br><br>a. Clinical symptoms of myocardial ischemia;<br>b. New myocardial ischemic changes in the ECG, including new ST-segment changes or left bundle branch block (LBBB) [According to whether there is ST-segment elevation in the ECG, it is classified as acute ST-segment elevation myocardial infarction (STEMI) and non-ST segment elevation myocardial infarction (NSTEMI)];<br>c. Pathological Q wave detected in ECG;<br>d. Imaging demonstration of new loss of viable myocardium or new regional wall motion abnormality;<br>e. Coronary thrombosis confirmed by angiography or autopsy.<br><br>(2) Cardiac death with symptoms suggestive of myocardial ischemia and presumed new ischaemic ECG changes or new LBBB, but death occurring before cardiac biomarkers could be obtained, before cardiac biomarker could rise, or in rare cases cardiac biomarkers were not collected.<br><br>(3) Myocardial infarction related to percutaneous coronary intervention (PCI) is arbitrarily defined by elevation of cTn values >5×99th percentile URL in patients with normal baseline values (≤99th percentile URL) or a rise of cTn values >20% if the baseline values are elevated and are stable or falling. In addition, any

© 2024 Gao Y et al. *JAMA Neurology.*

	of the followings is required:<br>a. Symptoms suggestive of myocardial ischemia;<br>b. New ischemic ECG changes or new LBBB;<br>c. Angiographic loss of patency of a major coronary artery or a side branch or persistent slow- or no-flow or embolization;<br>d. Imaging demonstration of new loss of viable myocardium or new regional wall motion abnormality.<br>(4) Myocardial infarction related to stent thrombosis is detected by coronary angiography or autopsy in the setting of myocardial ischemia and with a rise and/ or fall of cardiac biomarkers values with at least one value above the 99th percentile URL.<br>(5) Myocardial infarction related to coronary artery bypass grafting (CABG) is arbitrarily defined by elevation of cardiac biomarker values＞10×99th percentile URL in patients with normal baseline cTn values (≤99th percentile URL). In addition, any of the followings is required:<br>a. new pathological Q waves or new LBBB;<br>b. angiographic documented new graft or new native coronary artery occlusion;<br>c. imaging evidence of new loss of viable myocardium or new regional wall motion abnormality
**Vascular Death**	Vascular death includes sudden cardiac death, death due to stroke, acute myocardial infarction, heart failure, pulmonary embolism, cardiac/cerebrovascular intervention or surgery (unrelated to acute MI) and other cardiovascular causes [e.g. arrhythmia irrelevant with sudden cardiac death, aortic aneurysm rupture, or peripheral artery disease].<br>Any death of unknown/unclear cause within 30 d after stroke, myocardial infarction, or cardio-cerebrovascular operation/surgery will be regarded as death due to stroke, myocardial infarction, or cardio-cerebrovascular operation/surgery, respectively.

© 2024 Gao Y et al. *JAMA Neurology.*

Reference:

1. Thygesen K, Alpert JS, Jaffe AS, et al. Third universal definition of myocardial infarction. J Am Coll Cardiol 2012;60:1581-98.

© 2024 Gao Y et al. *JAMA Neurology.*

Additional Analyses

eFigure 1. Hazard ratio for stroke in prespecified subgroups.

The trial was not powered to allow definite conclusions based on the results of the subgroup analyses. Distribution of criminal arterial stenosis data was missing in 436 cases due to the absence of both intracranial and extracranial arterial vascular assessments. Degree of symptomatic stenosis data was missing in 394 cases due to the absence of both intracranial and extracranial arterial vascular assessments; or subjects did not have occlusion in intracranial (or extracranial) arteries, but was missing in extracranial (or intracranial) vascular assessments. The body-mass index is the weight in kilograms divided by the square of the height in meters. TIA denotes transient ischemic attack; ICAS, intracranial artery stenosis; ECAS, extracranial artery stenosis.

Subgroup	No. of Patients	Immediate Intensive Statin	Delayed Intensive Statin	Hazard Ratio (95%CI)	P for interaction
		no. of events (%)			
Overall	6100	245 (8.0)	256 (8.4)	0.95 (0.80 to 1.13)	
Body to mass index					0.62
<25	3520	138 (7.9)	152 (8.6)	0.91 (0.72 to 1.15)	
≥25	2580	107 (8.2)	104 (8.1)	0.99 (0.76 to 1.30)	
Qualifying event					0.20
Ischemic stroke	5299	234 (8.9)	240 (9.0)	0.99 (0.83 to 1.19)	
TIA	801	11 (2.6)	16 (4.3)	0.59 (0.28 to 1.28)	
ABCD² score in qualifying TIA					0.96
≤5	641	8 (2.4)	12 (4.0)	0.60 (0.25 to 1.48)	
>5	160	3 (3.3)	4 (5.7)	0.57 (0.13 to 2.54)	
Distribution of criminal arterial stenosis					0.12
No ICAS/ECAS/occlusion	1053	24(4.5)	36(6.9)	0.65 (0.39 to 1.09)	
ICAS/occlusion only	3408	128(7.6)	119(6.9)	1.09 (0.85 to 1.40)	
ECAS/occlusion only	445	18(7.8)	28(13.1)	0.58 (0.32 to 1.05)	
ICAS/occlusion and ECAS/occlusion	758	51(13.3)	49(13.1)	1.01 (0.68 to 1.49)	
Degree of symptomatic stenosis					0.29
Non stenosis	1053	24(4.5)	36(6.9)	0.65 (0.39 to 1.09)	
50 to 99% stenosis	3315	120(7.3)	117(7.0)	1.04 (0.80 to 1.34)	
Occlusion	1338	81(12.0)	86(13.0)	0.91 (0.67 to 1.24)	

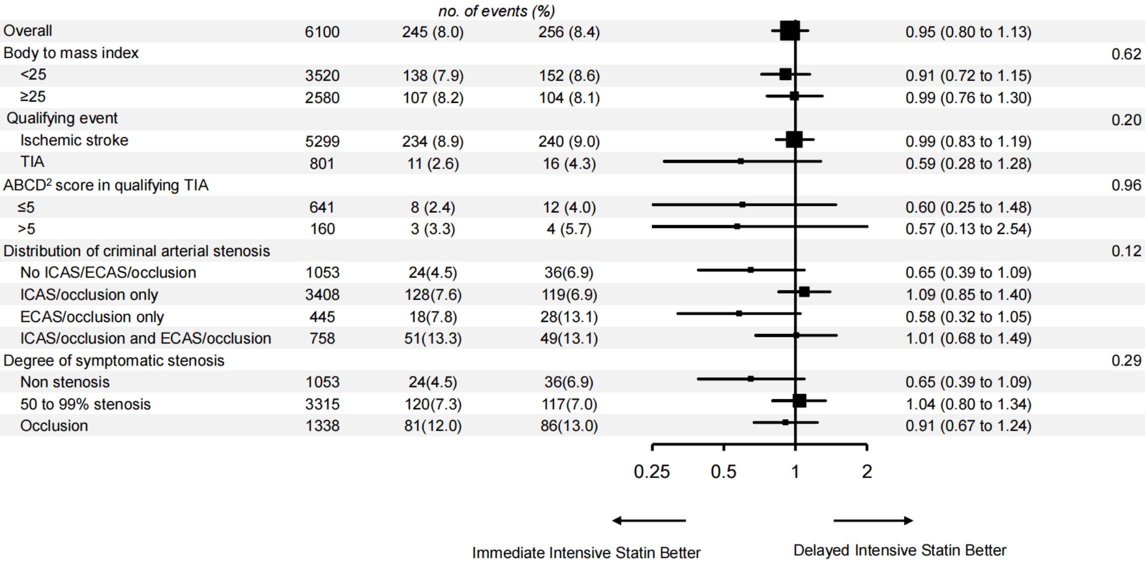

© 2024 Gao Y et al. *JAMA Neurology*.

eFigure 2. Odds ratio for poor functional outcome in prespecified subgroups.

The trial was not powered to allow definite conclusions based on the results of the subgroup analyses. The mRS score at 90 days data were missing in 7 patients, systolic blood pressure data were missing in 13 patients, with ≥50% symptomatic stenosis data was missing in 132 patients, distribution of criminal arterial stenosis data was missing in 436 cases, and Degree of symptomatic stenosis data was missing in 394 cases. The body-mass index is the weight in kilograms divided by the square of the height in meters. TIA denotes transient ischemic attack.

Subgroup	No. of Patients	Immediate Intensive Statin	Delayed Intensive Statin	Odds radio (95%CI)	P for interaction
		no. of events (%)			
Overall	6093	299(9.8)	348(11.4)	0.83 (0.71 to 0.98)	
Age					0.62
<65 yr	3135	118(7.6)	146(9.3)	0.80 (0.62 to 1.03)	
≥65 yr	2958	181(12.2)	202(13.7)	0.87 (0.70 to 1.08)	
Sex					0.64
Men	3910	192(9.6)	209(10.9)	0.87 (0.71 to 1.07)	
Women	2183	107(10.1)	139(12.3)	0.80 (0.61 to 1.05)	
Systolic Blood Pressure					0.71
<140mmHg	2137	84(7.8)	94(8.8)	0.88 (0.65 to 1.21)	
≥140mmHg	3943	215(10.9)	253(12.8)	0.82 (0.68 to 1.00)	
Hypertension					0.62
Yes	4079	217(10.6)	255(12.5)	0.81 (0.67 to 0.99)	
No	2014	82(8.2)	93(9.2)	0.88 (0.64 to 1.20)	
Diabetes mellitus					0.01
Yes	1657	119(14.7)	110(13.0)	1.12 (0.84 to 1.48)	
No	4436	180(8.1)	238(10.8)	0.72 (0.59 to 0.89)	
Dyslipidaemia					0.06
Yes	225	15(13.0)	7(6.4)	2.21 (0.86 to 5.68)	
No	5868	284(9.7)	341(11.6)	0.81 (0.68 to 0.96)	
Qualifying event					0.91
TIA	800	12(2.8)	14(3.8)	0.73 (0.33 to 1.61)	
Acute single infarction	1174	51(8.8)	61(10.3)	0.82 (0.55 to 1.22)	
Acute multiple infarctions	4119	236(11.6)	273(13.1)	0.87 (0.72 to 1.05)	
NIHSS in qualifying ischemic stroke					0.93
0 to 1	1882	40(4.4)	47(4.8)	0.92 (0.60 to 1.42)	
2 to 3	2147	100(9.2)	112(10.6)	0.85 (0.64 to 1.13)	
>3	1264	147(23.7)	175(27.2)	0.81 (0.62 to 1.05)	
Time to randomization					0.009
≤ 48h	3331	156(9.3)	211(12.8)	0.70 (0.56 to 0.87)	
>48h	2762	143(10.5)	137(9.8)	1.07 (0.84 to 1.37)	
With ≥50% symptomatic stenosis					0.34
Yes	4909	250(10.2)	295(12.0)	0.83 (0.69 to 0.99)	
No	1052	41(7.7)	38(7.3)	1.07 (0.67 to 1.70)	
Previous antiplatelet therapy					0.18
Yes	821	54(12.9)	71(17.6)	0.66 (0.45 to 0.98)	
No	5272	245(9.3)	277(10.5)	0.88 (0.73 to 1.05)	
Previous statin therapy					0.22
Yes	565	36(12.5)	50(18.1)	0.62 (0.39 to 1.00)	
No	5528	263(9.5)	298(10.8)	0.87 (0.73 to 1.04)	
Antiplatelet therapy assignment					0.49
Clopidogrel to Aspirin	3047	143(9.4)	158(10.4)	0.89 (0.70 to 1.13)	
Aspirin	3046	156(10.2)	190(12.5)	0.80 (0.64 to 1.00)	
Body to mass index					0.68
<25	3516	180(10.3)	218(12.4)	0.81 (0.66 to 1.00)	
≥25	2577	119(9.2)	130(10.1)	0.87 (0.67 to 1.14)	
Qualifying event					0.66
Ischemic stroke	5293	287(11.0)	334(12.5)	0.86 (0.73 to 1.02)	
TIA	800	12(2.8)	14(3.8)	0.73 (0.33 to 1.61)	
ABCD² score in qualifying TIA					0.28
≤5	640	8(2.4)	7(2.3)	1.02 (0.37 to 2.86)	
>5	160	4(4.4)	7(10.0)	0.39 (0.10 to 1.46)	
Distribution of criminal arterial stenosis					0.30
No ICAS/ECAS/occlusion	1052	41(7.7)	38(7.3)	1.07 (0.67 to 1.70)	
ICAS/occlusion only	3403	159(9.4)	176(10.3)	0.91 (0.72 to 1.14)	
ECAS/occlusion only	445	24(10.4)	35(16.4)	0.59 (0.34 to 1.04)	
ICAS/occlusion and ECAS/occlusion	757	46(12.0)	61(16.3)	0.70 (0.46 to 1.06)	
Degree of symptomatic stenosis					0.41
No stenosis	1052	41(7.7)	38(7.3)	1.06 (0.67 to 1.68)	
50 to 99% stenosis	3311	138(8.4)	162(9.7)	0.85 (0.67 to 1.08)	
Occlusion	1336	95(14.0)	120(18.2)	0.73 (0.54 to 0.99)	

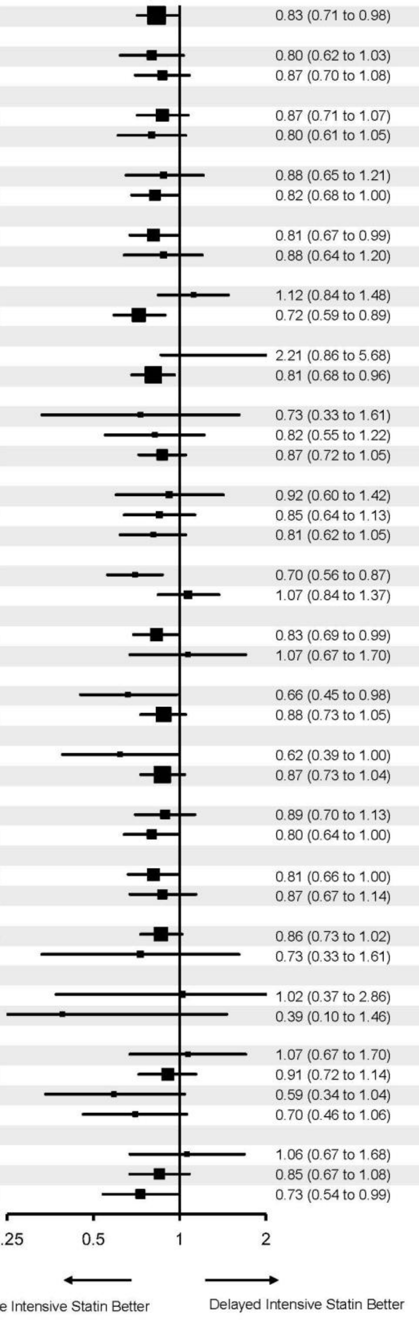

© 2024 Gao Y et al. *JAMA Neurology.*

eFigure 3. Subgroup analysis for stroke by center.

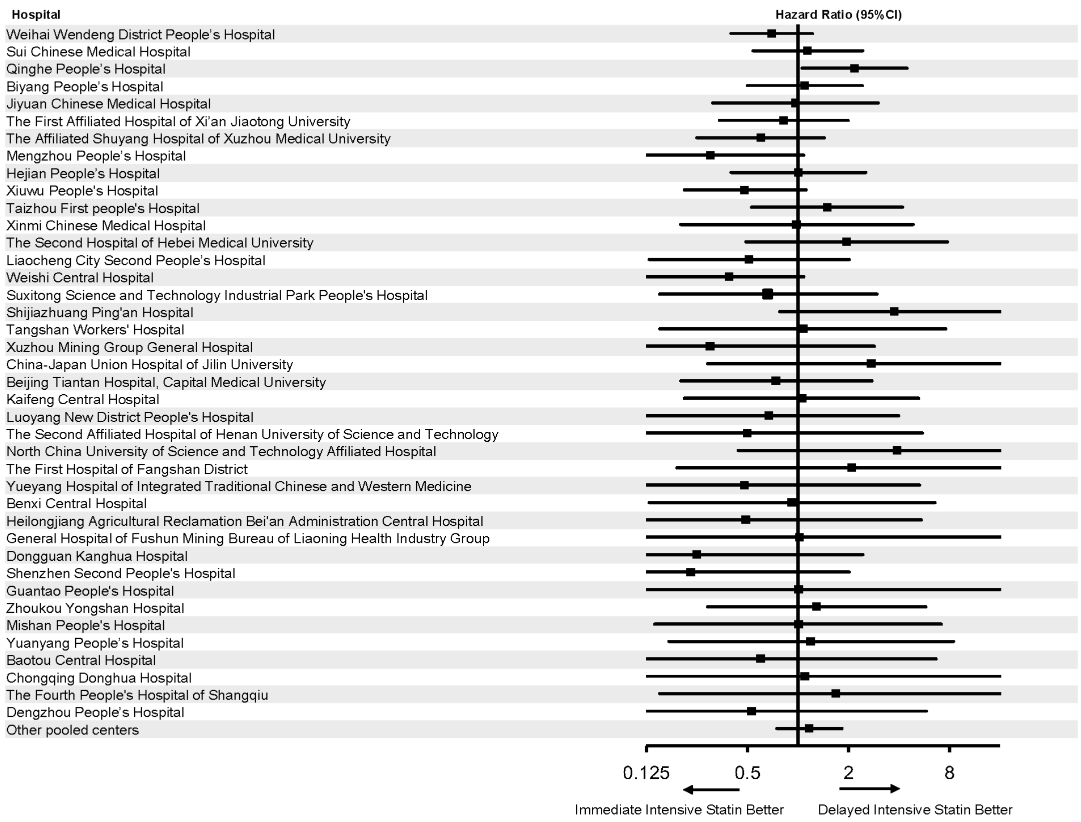

© 2024 Gao Y et al. *JAMA Neurology.*

eTable 1. Baseline characteristics of the patients.

Characteristic	Immediate Intensive Statin (N=3050)	Delayed Intensive Statin (N=3050)
Ethnicity, no. (%)[a]		
Han	2999 (98.3)	3012 (98.8)
Zhuang	8 (0.3)	7 (0.2)
Hui	16 (0.5)	9 (0.3)
Manchu	11 (0.4)	8 (0.3)
Uygur	1 (0.03)	4 (0.1)
Others	15 (0.5)	10 (0.3)
Blood Pressure, median (IQR), mmHg[b]		
Systolic	146 (132-160)	146 (133-160)
Diastolic	85 (78-93)	85 (78-94)
Medical History, no. (%)		
Coronary artery disease	356 (11.7)	336 (11.0)
Peripheral arterial disease	8 (0.3)	9 (0.3)
Application of drugs within 1 month before onset of symptoms, no. (%)		
Aspirin+ clopidogrel	12 (0.4)	10 (0.3)
Antihypertensive agents	1520 (49.8)	1509 (49.5)
Hypoglycemic agents	685 (22.5)	717 (23.5)
Folic acid	13 (0.4)	9 (0.3)
Distribution of criminal arterial stenosis/ occlusion, no. (%) [c]		
No intracranial/extracranial artery stenosis/ occlusion	533/2839(18.8)	520/2825(18.4)
Intracranial artery stenosis/ occlusion only	1692/2839(59.6)	1716/2825(60.7)
Extracranial artery stenosis/ occlusion only	231/2839(8.1)	214/2825(7.6)
Intracranial and extracranial artery stenosis/ occlusion	383/2839(13.5)	375/2825(13.3)
Stenosis degree of symptomatic qualifying artery, no. (%) [d]		
No stenosis/ occlusion	533/2857(18.7)	520/2849(18.3)
50-99% stenosis	1647/2857(57.7)	1668/2849(58.6)
Occlusion	677/2857(23.7)	661/2849(23.2)
Time to randomization after onset of symptoms, no. (%)		
≤ 24h	391 (12.8)	392 (12.9)
24h-48h	1289 (42.3)	1263 (41.4)
>48h	1370 (44.9)	1395 (45.7)
mRS score before onset of symptoms, no. (%)[e]		
0	2398 (78.6)	2424 (79.5)
1	533 (17.5)	487 (16.0)
2	119 (3.9)	138 (4.5)

[a] Ethnic group was reported by the patient and verified by identification card.

[b] IQR denotes interquartile range. Data were missing in 13 cases.

[c] The stenosis degree of intracranial artery was defined on MRA, CTA or DSA by criteria from the Warfarin–Aspirin Symptomatic

© 2024 Gao Y et al. *JAMA Neurology*.

Intracranial Disease study and assessments of extracranial arteries stenosis degree was based on carotid duplex ultrasound, CTA, CE-MRA or DSA by standards from the North American Symptomatic Carotid Endarterectomy Trial. The criminal intracranial artery stenosis and extracranial artery stenosis were defined as more than 50% stenosis of intracranial and extracranial major arteries, respectively, which likely accounts for the infarction and clinical presentation. Data were missing in 132 cases due to the absence of both intracranial or extracranial arterial vascular assessments.

[d] Data was missing in 394 cases due to the absence of both intracranial and extracranial arterial vascular assessments; or subjects did not have occlusion in intracranial (or extracranial) arteries, but was missing in extracranial (or intracranial) vascular assessments.

[e] The modified Rankin scale assesses measure functional recovery after stroke, with scores ranging from 0 to 6 and higher scores indicating more severe disability.

© 2024 Gao Y et al. *JAMA Neurology*.

eTable 2. Concomitant treatment within 90 days.

Concomitant Prohibited Medication	Immediate Intensive Statin (N=3050) no. (%)	Delayed Intensive Statin (N=3050) no. (%)
**Medicine use during hospitalization**		
Antihypertensive	1638 (53.7)	1653 (54.2)
Diuretics	123 (4.0)	138 (4.5)
Calcium Antagonists	908 (29.8)	954 (31.3)
Angiotensin-Converting Enzyme Inhibitors	153 (5.0)	144 (4.7)
Angiotensin Receptor blockers	274 (9.0)	227 (7.4)
Adrenoceptor Antagonist	78 (2.6)	75 (2.5)
Antidiabetic	865 (28.4)	927 (30.4)
Oral Antidiabetics	573 (18.8)	607 (19.9)
Insulin	171 (5.6)	162 (5.3)
**Medicine use at 90-day follow-up**		
Antihypertensive	1568 (51.4)	1584 (51.9)
Diuretics	110 (3.6)	127 (4.2)
Calcium Antagonists	1223 (40.1)	1257 (41.2)
Angiotensin-Converting Enzyme Inhibitors	143 (4.7)	153 (5.0)
Angiotensin Receptor blockers	411 (13.5)	394 (12.9)
Adrenoceptor Antagonist	109 (3.6)	98 (3.2)
Antidiabetic	787 (25.8)	826 (27.1)
Oral Antidiabetics	704 (23.1)	752 (24.7)
Insulin	210 (6.9)	190 (6.2)

© 2024 Gao Y et al. *JAMA Neurology.*

eTable 3. Efficacy and safety outcomes in per-protocol population.

Outcome	Immediate Intensive Statin (N=2816)		Delayed Intensive Statin (N=2820)		Treatment Effect [b] (95% CI)	P Value
	Patients with Event-no.	Event Rate[a], %	Patients with Event-no.	Event Rate[a], %		
**Primary outcome**						
Stroke (including ischemic and hemorrhagic stroke)	227	8.1	240	8.5	0.94 (0.79 to 1.13)	0.52
**Secondary outcomes**						
Composite vascular event (Stroke, myocardial infarction, or vascular death)	231	8.2	244	8.7	0.94 (0.79 to 1.13)	0.53
Ischemic stroke	217	7.7	231	8.2	0.94 (0.78 to 1.13)	0.49
Recurrent stroke	157	5.7	178	6.4	0.88 (0.71 to 1.09)	0.24
TIA with infarction	10	0.4	6	0.2	1.65 (0.60 to 4.55)	0.33
Progressive stroke	50	1.8	47	1.7	1.07 (0.72 to 1.59)	0.75
Hemorrhagic stroke	11	0.4	9	0.3	1.23 (0.51 to 2.96)	0.65
TIA	20	0.7	17	0.6	1.17 (0.62 to 2.24)	0.63
Myocardial infarction	2	0.07	3	0.1	0.67 (0.11 to 4.00)	0.66
Vascular death	17	0.6	16	0.6	1.06 (0.54 to 2.11)	0.86
Poor functional outcome (mRS 2-6) [c]	257/2816	9.1	306/2818	10.9	0.82 (0.69 to 0.98)	0.03
Early neurological deterioration (the change of NIHSS score at 7 days), median (IQR)[d]	0 (-1 to 0)		0 (-1 to 0)		-0.001(-0.100 to 0.097)	0.98
Ordinal stroke or TIA[e]					0.96 (0.80 to 1.15)	0.65
Fatal stroke: score of 6 on mRS	15/2816	0.5	15/2819	0.5		
Severe stroke: score of 4 or 5 on mRS	28/2816	1.0	24/2819	0.9		
Moderate stroke: score of 2 or 3 on mRS	65/2816	2.3	90/2819	3.2		
Mild stroke: score of 0 or 1 on mRS	119/2816	4.2	110/2819	3.9		
TIA	19/2816	0.7	16/2819	0.6		
No stroke or TIA	2570/2816	91.3	2564/2819	91.0		
**Primary safety outcomes**						
Moderate-to-severe bleeding [f]	17	0.6	14	0.5	1.22 (0.60 to 2.48)	0.58
**Secondary safety outcomes**						
Hepatotoxicity [g]	27	1.0	20	0.7	1.35 (0.76 to 2.42)	0.31
Muscle toxicity [h]	2	0.07	1	0.04	2.00 (0.18 to 22.12)	0.57
All-cause mortality	25	0.9	29	1.0	0.86 (0.51 to 1.47)	0.59
Any bleeding [f]	58	2.1	54	1.9	1.08 (0.75 to 1.57)	0.68
Intracranial hemorrhage	14	0.5	11	0.4	1.28 (0.58 to 2.81)	0.54
Mild bleeding	45	1.6	40	1.4	1.14 (0.74 to 1.74)	0.55

[a] The event rates of poor functional outcome (mRS 2-6), ordinal stroke or TIA, hepatotoxicity and muscle toxicity are raw estimates, whereas the

© 2024 Gao Y et al. *JAMA Neurology*.

rates of other outcomes are Kaplan–Meier estimates of the percentage of patients with events at 90 days.

[b] The odds ratios are shown for poor functional outcome (mRS 2-6), hepatotoxicity and muscle toxicity. The common odds ratio is shown for ordinal stroke or TIA. Beta coefficient is shown for Early neurological deterioration (the change of NIHSS score at 7 days). Hazard ratios are shown for other outcomes. The widths of the confidence intervals for secondary outcomes were not adjusted for multiplicity and may not be used for hypothesis testing.

[c] Modified Rankin Scale (mRS) scores range from 0 to 6, with 0 indicating no symptoms; 1, symptoms without clinically significant disability; 2, slight disability; 3, moderate disability; 4, moderately severe disability; 5, severe disability; and 6, death. The mRS score data at 90days was missing in 2 patients in the delayed intensive statin group.

[d] The change of NIHSS score at 7 days was analyzed by generalized linear models with continuous variables.

[e] The severity of stroke or TIA is classified on a six-level ordered categorical scale combined vascular events with modified Rankin scale (mRS). The mRS score at 90days was missing in 1 patient with new stroke in the delayed intensive statin group.

[f] Bleeding events were defined according to the Global Utilization of Streptokinase and Tissue Plasminogen Activator for Occluded Coronary Arteries criteria.

[g] Hepatotoxicity was defined as alkaline phosphatase or aspartate aminotransferase >3 times the upper limit of normal value.

[h] Muscle toxicity was defined as creatine kinase >10 times the upper limit of normal value, or presence of muscle pain, myopathy or rhabdomyolysis.

© 2024 Gao Y et al. *JAMA Neurology*.

eTable 4. Number of patients with adverse events[a,b] by system organ class (excluding strokes) up to 3-month visit.

System organ class	Immediate Intensive Statin (N=3050) no. (%)	Delayed Intensive Statin (N=3050) no. (%)	P value
Overall	693(22.7)	605(19.8)	0.006
Blood and lymphatic system disorders	3(0.1)	3(0.1)	>0.99
Cardiac disorders	33(1.1)	19(0.6)	0.051
Arrhythmia	23(0.8)	9(0.3)	0.01
Coronary heart disease	4(0.1)	2(0.07)	0.69
Ear and labyrinth disorders	5(0.2)	7(0.2)	0.56
Endocrine disorders	0(0.0)	1(0.03)	>0.99
Eye disorders	6(0.2)	7(0.2)	0.78
Gastrointestinal disorders	94(3.1)	78(2.6)	0.22
Gastrointestinal Hemorrhage	2(0.07)	0(0.0)	0.50
Gingival bleeding	4(0.1)	3(0.1)	>0.99
General disorders and administration site conditions	12(0.4)	9(0.3)	0.51
Hepatobiliary disorders	14(0.5)	16(0.5)	0.71
Immune system disorders	8(0.3)	6(0.2)	0.59
Infections and infestations	67(2.2)	61(2.0)	0.59
Lung infection	23(0.8)	11(0.4)	0.04
Upper respiratory tract infection	18(0.6)	17(0.6)	0.87
Injury, poisoning and procedural complications	7(0.2)	9(0.3)	0.62
Investigations	287(9.4)	230(7.5)	0.01
Metabolism and nutrition disorders	63(2.1)	66(2.2)	0.79
Musculoskeletal and connective tissue disorders	14(0.5)	21(0.7)	0.24
Neoplasms benign, malignant and unspecified (incl cysts and polyps)	24(0.8)	22(0.7)	0.77
Nervous system disorders	112(3.7)	86(2.8)	0.06
Psychiatric disorders	29(1.0)	26(0.9)	0.68
Renal and urinary disorders	11(0.4)	16(0.5)	0.33
Reproductive system and breast disorders	12(0.4)	7(0.2)	0.25
Respiratory, thoracic and mediastinal disorders	17(0.6)	17(0.6)	>0.99
Dyspnea	0(0.0)	1(0.03)	>0.99
Epistaxis	5(0.2)	6(0.2)	0.76
Skin and subcutaneous tissue disorders	15(0.5)	17(0.6)	0.72

© 2024 Gao Y et al. *JAMA Neurology.*

Subcutaneous Hemorrhage/Dermatorrhagia	6(0.2)	7(0.2)	0.78
Rash/Pruritus/Urticaria	9(0.3)	9(0.3)	>0.99
Surgical and medical procedures	16(0.5)	21(0.7)	0.41
Vascular disorders	22(0.7)	26(0.9)	0.56

[a] Adverse events did not include serious adverse events. Includes adverse events with an onset date on or after the date of first dose and up to the date of last dose of study medication.

[b] Patients with multiple events of one type were counted once.

© 2024 Gao Y et al. *JAMA Neurology.*

eTable 5. Number of patients with serious adverse events[a] by system organ class (excluding strokes) up to 3-month visit.

System organ class	Immediate Intensive Statin (N=3050) no. (%)	Delayed Intensive Statin (N=3050) no. (%)	P value
Overall	96(3.2)	100(3.3)	0.77
Blood and lymphatic system disorders	0(0.0)	1(0.03)	>0.99
Cardiac disorders	8(0.3)	14(0.5)	0.20
Ear and labyrinth disorders	0(0.0)	1(0.03)	>0.99
Eye disorders	1(0.03)	0(0.0)	>0.99
Gastrointestinal disorders	9(0.3)	9(0.3)	>0.99
General disorders and administration site conditions	4(0.1)	7(0.2)	0.55
Hepatobiliary disorders	1(0.03)	3(0.1)	0.62
Infections and infestations	6(0.2)	6(0.2)	>0.99
Injury, poisoning and procedural complications	4(0.1)	5(0.2)	>0.99
Investigations	3(0.1)	3(0.1)	>0.99
Metabolism and nutrition disorders	0(0.0)	2(0.07)	0.50
Neoplasms benign, malishergnant and unspecified (incl cysts and polyps)	3(0.1)	3(0.1)	>0.99
Nervous system disorders	46(1.5)	35(1.2)	0.22
Psychiatric disorders	1(0.03)	4(0.1)	0.37
Respiratory, thoracic and mediastinal disorders	2(0.07)	5(0.2)	0.45
Skin and subcutaneous tissue disorders	1(0.03)	0(0.0)	>0.99
Surgical and medical procedures	10(0.3)	10(0.3)	>0.99
Vascular disorders	4(0.1)	0(0.0)	0.12

[a] Patients with multiple events of one type were counted once. Includes adverse events with an onset date on or after the date of first dose and up to the date of last dose of study medication.

© 2024 Gao Y et al. *JAMA Neurology.*

eTable 6. Number of patients with adverse events or serious adverse events leading to premature permanent drug discontinuation by system organ class up to 3-month visit.

System organ class	Immediate Intensive Statin (N=3050) no. (%)	Delayed Intensive Statin (N=3050) no. (%)	P value
Blood and lymphatic system disorders	1(0.03)	1(0.03)	>0.99
Cardiac disorders	13(0.4)	10(0.3)	0.53
Ear and labyrinth disorders	0(0.0)	1(0.03)	>0.99
Endocrine disorders	0(0.0)	0(0.0)	-
Eye disorders	0(0.0)	1(0.03)	>0.99
Gastrointestinal disorders	14(0.5)	13(0.4)	0.85
General disorders and administration site conditions	3(0.1)	3(0.1)	>0.99
Hepatobiliary disorders	1(0.03)	1(0.03)	>0.99
Immune system disorders	1(0.03)	1(0.03)	>0.99
Infections and infestations	10(0.3)	3(0.1)	0.09
Injury, poisoning and procedural complications	1(0.03)	2(0.07)	>0.99
Investigations	12(0.4)	10(0.3)	0.67
Metabolism and nutrition disorders	0(0.0)	1(0.03)	>0.99
Musculoskeletal and connective tissue disorders	1(0.03)	2(0.07)	>0.99
Neoplasms benign, malignant and unspecified (incl cysts and polyps)	2(0.07)	3(0.10)	>0.99
Nervous system disorders	30(1.0)	35(1.2)	0.53
Psychiatric disorders	1(0.03)	3(0.1)	0.62
Renal and urinary disorders	0(0.0)	1(0.03)	>0.99
Reproductive system and breast disorders	0(0.0)	0(0.0)	-
Respiratory, thoracic and mediastinal disorders	5(0.2)	3(0.1)	0.73
Skin and subcutaneous tissue disorders	1(0.03)	3(0.1)	0.62
Surgical and medical procedures	18(0.6)	19(0.6)	0.87
Vascular disorders	7(0.2)	3(0.1)	0.34

© 2024 Gao Y et al. *JAMA Neurology.*

eTable 7. mRS outcome adjusted by the baseline proportion of TIA and infarction and baseline NIHSS score. [a]

Outcome	Crude OR (95% CI)	P Value	Adjust OR [b] (95% CI)	P Value
**Secondary outcomes**				
Poor functional outcome (mRS 2-6)	0.83 (0.71 to 0.98)	0.03	0.83 (0.68 to 1.00)	0.05
Poor functional outcome (mRS) [c]	0.99 (0.90 to 1.09)	0.81	0.99 (0.90 to 1.10)	0.87
Poor functional outcome (mRS 2-6) [d]	0.86 (0.71 to 1.04)	0.11	0.86 (0.71 to 1.05)	0.14
Poor functional outcome (mRS) [e]	1.01 (0.91 to 1.11)	0.90	1.01 (0.91 to 1.13)	0.81

[a] mRS denotes modified Rankin scale.

[b] The resulted were adjusted by baseline proportion of TIA and infarction and baseline NIHSS score. The odds ratios are shown for poor functional outcome (mRS 2-6), the common odds ratio is shown for ordinal stroke or TIA (mRS).

[c] The post hoc shift analysis was performed.

[d] The patients with pre-stroke mRS 2 were excluded.

[e] The post hoc shift analysis was performed after excluding the patients with pre-stroke mRS.

© 2024 Gao Y et al. *JAMA Neurology*.
