# Supplementary material for: Immediate- or Delayed-Intensive Statin in Acute Cerebral Ischemia: The INSPIRES Randomized Clinical Trial
Source: JAMA Neurol. 2024 May 28;81(7):741–51. doi: 10.1001/jamaneurol.2024.1433 (PMC11134282; doi:10.1001/jamaneurol.2024.1433)
Supplement: Supplement 4. — Nonauthor Collaborators. INSPIRES Investigators [file jamaneurol-e241433-s004.pdf]

Supplemental Online Content: Nonauthor Collaborators
*First name, last name, and suffix (if applicable) are required and will appear in PubMed.

***Group Name(s): INSPIRES Investigators**

*First Name and Middle Initial(s)	*Last Name	*Suffix (eg, Jr, III)	Academic Degrees	Institution	Location (city, state/province, country)	Role or Contribution, eg, chair, principal investigator	Group (if more than 1 Group listed in the byline) and/or Subgroup (eg, Steering Committee)
Yilong	Wang	N/A	MD,PhD	Beijing Tiantan Hospital, Capital Medical University	Beijing, China	Principal Investigator	Principal Investigator
Yongjun	Wang	N/A	MD	Beijing Tiantan Hospital, Capital Medical University	Beijing, China	Principal Investigator	Principal Investigator
Jinguo	Zhao	N/A	MD	Weihai Wendeng District People ’ s Hospital	Weihai, Shandong, China	Site Principal Investigator	Research Site Investigators
Ying	Li	N/A	MD	Sui Chinese Medical Hospital	Shangqiu, Henan, China	Site Principal Investigator	Research Site Investigators
Yingzhuo	Zang	N/A	MD	Qinghe People’s Hospital	Xingtai, Hebei, China	Site Principal Investigator	Research Site Investigators
Shuo	Zhang	N/A	MD	Biyang People’s Hospital	Zhumadian, Henan, China	Site Principal Investigator	Research Site Investigators
Hongqin	Yang	N/A	MD	Jiyuan Chinese Medical Hospital	Jiyuan, Henan, China	Site Principal Investigator	Research Site Investigators
Jianbo	Yang	N/A	MD	The First Affiliated Hospital of Xi ’ an Jiaotong University	Xi’an, Shaanxi, China	Site Principal Investigator	Research Site Investigators
Yuanwei	Wang	N/A	MD	The Affiliated Shuyang Hospital of Xuzhou Medical University	Suqian, Jiangsu, China	Site Principal Investigator	Research Site Investigators
Dali	Li	N/A	MD	Mengzhou People’s Hospital	Jiaozuo, Henan, China	Site Principal Investigator	Research Site Investigators
Yanxia	Wang	N/A	MD	Hejian People’s Hospital	Cangzhou, Hebei, China	Site Principal Investigator	Research Site Investigators
Dongqi	Liu	N/A	MD	Hejian People’s Hospital	Cangzhou, Hebei, China	Site Principal Investigator	Research Site Investigators
Guangming	Kang	N/A	MD	Xiuwu People's Hospital	Jiaozuo, Henan, China	Site Principal Investigator	Research Site Investigators
Zhimin	Wang	N/A	MD	Taizhou First people's Hospital	Taizhou, Zhejiang, China	Site Principal Investigator	Research Site Investigators
Jianmin	Guo	N/A	MD	Xinmi Chinese Medical Hospital	Zhengzhou, Henan, China	Site Principal Investigator	Research Site Investigators
Xiujuan	Song	N/A	MD	The Second Hospital of Hebei Medical University	Shijiazhuang, Hebei, China	Site Principal Investigator	Research Site Investigators

*First Name and Middle Initial(s)	*Last Name	*Suffix (eg, Jr, III)	Academic Degrees	Institution	Location (city, state/province, country)	Role or Contribution, eg, chair, principal investigator	Group (if more than 1 Group listed in the byline) and/or Subgroup (eg, Steering Committee)
Xinqiang	Wang	N/A	MD	Liaocheng City Second People's Hospital	Liaocheng, Shandong, China	Site Principal Investigator	Research Site Investigators
Weifeng	Lu	N/A	MD	Weishi Central Hospital	Kaifeng, Henan, China	Site Principal Investigator	Research Site Investigators
Panbing	Huang	N/A	MD	Suxitong Science and Technology Industrial Park People's Hospital	Nantong, Jiangsu, China	Site Principal Investigator	Research Site Investigators
Feng	Li	N/A	MD	Pingyu People's Hospital	Zhumadian, Henan, China	Site Principal Investigator	Research Site Investigators
Lihua	Wang	N/A	MD	The Second Hospital of Harbin Medical University	Harbin, Heilongjiang, China	Site Principal Investigator	Research Site Investigators
Weigang	Xiao	N/A	MD	Shijiazhuang Ping'an Hospital	Shijiazhuang, Hebei, China	Site Principal Investigator	Research Site Investigators
Yibin	Cao	N/A	MD	Tangshan Workers' Hospital	Tangshan, Hebei, China	Site Principal Investigator	Research Site Investigators
Liangqun	Rong	N/A	MD	Xuzhou Mining Group General Hospital	Xuzhou, Jiangsu, China	Site Principal Investigator	Research Site Investigators
Ying	Xing	N/A	MD	China-Japan Union Hospital of Jilin University	Changchun, Jilin, China	Site Principal Investigator	Research Site Investigators
Lili	Ma	N/A	MD	Kaifeng Central Hospital	Kaifeng, Henan, China	Site Principal Investigator	Research Site Investigators
Yanhua	Zhou	N/A	MD	Panjin Central Hospital	Panjin, Liaoning, China	Site Principal Investigator	Research Site Investigators
YuQing	Han	N/A	MD	Tianjin Xiqing Hospital	Tianjin, China	Site Principal Investigator	Research Site Investigators
Jingxian	Fang	N/A	MD	The First people's Hospital of Nanyang	Nanyang, Henan, China	Site Principal Investigator	Research Site Investigators
Jie	Liu	N/A	MD	Luoyang New District People's Hospital	Luoyang, Henan, China	Site Principal Investigator	Research Site Investigators
Wen	Shangguan	N/A	MD	The Second Affiliated Hospital of Henan University of Science and Technology	Luoyang, Henan, China	Site Principal Investigator	Research Site Investigators
Bin	Liu	N/A	MD	North China University of Science and Technology Affiliated Hospital	Tangshan, Hebei, China	Site Principal Investigator	Research Site Investigators

Supplemental Online Content: Nonauthor Collaborators
*First name, last name, and suffix (if applicable) are required and will appear in PubMed.

*First Name and Middle Initial(s)	*Last Name	*Suffix (eg, Jr, III)	Academic Degrees	Institution	Location (city, state/province, country)	Role or Contribution, eg, chair, principal investigator	Group (if more than 1 Group listed in the byline) and/or Subgroup (eg, Steering Committee)
Jianhua	Li	N/A	MD	The First Hospital of Fangshan District	Beijing, China	Site Principal Investigator	Research Site Investigators
Yan	Han	N/A	MD	Yueyang Hospital of Integrated Traditional Chinese and Western Medicine, Shanghai University of Traditional Chinese Medicine	Shanghai, China	Site Principal Investigator	Research Site Investigators
Chengguang	Song	N/A	MD	Benxi Central Hospital	Benxi, Liaoning, China	Site Principal Investigator	Research Site Investigators
Xuhong	Song	N/A	MD	Heilongjiang Agricultural Reclamation Bei'an Administration Central Hospital	Heihe, Heilongjiang, China	Site Principal Investigator	Research Site Investigators
Yuanfeng	Lv	N/A	MD	General Hospital of Fushun Mining Bureau of Liaoning Health Industry Group	Fushun, Liaoning, China	Site Principal Investigator	Research Site Investigators
Changqing	Xu	N/A	MD	Dongguan Kanghua Hospital	Dongguan, Guangdong, China	Site Principal Investigator	Research Site Investigators
Congmin	Ma	N/A	MD	Luoyang Central Hospital	Luoyang, Henan, China	Site Principal Investigator	Research Site Investigators
Zhihui	Duan	N/A	MD	Luoyang Central Hospital	Luoyang, Henan, China	Site Principal Investigator	Research Site Investigators
Yungao	Pan	N/A	MD	Nanle Zhongxing Hospital	Puyang, Henan, China	Site Principal Investigator	Research Site Investigators
Lijie	Ren	N/A	MD	Shenzhen Second People's Hospital	Shenzhen, Guangdong, China	Site Principal Investigator	Research Site Investigators
Bin	Li	N/A	MD	Guantao People's Hospital	Handan, Hebei, China	Site Principal Investigator	Research Site Investigators
Jinqi	Fan	N/A	MD	Zhoukou Yongshan Hospital	Zhoukou, Henan, China	Site Principal Investigator	Research Site Investigators
Yuanren	Zhang	N/A	MD	Mishan People's Hospital	Jixi, Heilongjiang, China	Site Principal Investigator	Research Site Investigators
Jingfang	Li	N/A	MD	Yuanyang People's Hospital	Xinxiang, Henan, China	Site Principal Investigator	Research Site Investigators
Baojun	Wang	N/A	MD	Baotou Central Hospital	Baotou, Inner Mongolia, China	Site Principal Investigator	Research Site Investigators

Supplemental Online Content: Nonauthor Collaborators
*First name, last name, and suffix (if applicable) are required and will appear in PubMed.

*First Name and Middle Initial(s)	*Last Name	*Suffix (eg, Jr, III)	Academic Degrees	Institution	Location (city, state/province, country)	Role or Contribution, eg, chair, principal investigator	Group (if more than 1 Group listed in the byline) and/or Subgroup (eg, Steering Committee)
Jun	Gu	N/A	MD	Rudong People's Hospital	Nantong, Jiangsu, China	Site Principal Investigator	Research Site Investigators
Xiaoping	Yin	N/A	MD	Affiliated Hospital of Jiujiang College	Jiujiang, Jiangxi, China	Site Principal Investigator	Research Site Investigators
Xiao	Wang	N/A	MD	Zouping People's Hospital	Binzhou, Shandong, China	Site Principal Investigator	Research Site Investigators
Liguo	Chang	N/A	MD	The Third People's Hospital of Liaocheng	Liaocheng, Shandong, China	Site Principal Investigator	Research Site Investigators
Kaoling	Gong	N/A	MD	Shimen People's Hospital	Changde, Hunan, China	Site Principal Investigator	Research Site Investigators
Wenhua	Zhang	N/A	MD	Zhoukou Renhe Hospital	Zhoukou, Henan, China	Site Principal Investigator	Research Site Investigators
Yu	Che	N/A	MD	Chongqing Donghua Hospital	Chongqing, China	Site Principal Investigator	Research Site Investigators
Yinyuan	Wan	N/A	MD	Fengqiu People's Hospital	Xinxiang, Henan, China	Site Principal Investigator	Research Site Investigators
Linying	Gui	N/A	MD	The Sixth People's Hospital of Hengshui	Hengshui, Hebei, China	Site Principal Investigator	Research Site Investigators
Ping	Sun	N/A	MD	The Second People's Hospital of Guiyang	Guiyang, Guizhou, China	Site Principal Investigator	Research Site Investigators
Zhonghai	Jia	N/A	MD	Mengjin People's Hospital	Luoyang, Henan, China	Site Principal Investigator	Research Site Investigators
Haichao	Liu	N/A	MD	The Fourth People's Hospital of Shangqiu	Shangqiu, Henan, China	Site Principal Investigator	Research Site Investigators
Qinglian	Meng	N/A	MD	Gucheng People's Hospital	Hengshui, Hebei, China	Site Principal Investigator	Research Site Investigators
Donghe	Chai	N/A	MD	Dengzhou People's Hospital	Nanyang, Henan, China	Site Principal Investigator	Research Site Investigators
Lei	Zhang	N/A	MD	Zhenping People's Hospital	Nanyang, Henan, China	Site Principal Investigator	Research Site Investigators
Guofeng	Li	N/A	MD	Ruyang People's Hospital	Luoyang, Henan, China	Site Principal Investigator	Research Site Investigators
Huafeng	Jin	N/A	MD	Changzhou Wujin Traditional Chinese Medicine Hospital	Changzhou, Jiangsu, China	Site Principal Investigator	Research Site Investigators

Supplemental Online Content: Nonauthor Collaborators
*First name, last name, and suffix (if applicable) are required and will appear in PubMed.

*First Name and Middle Initial(s)	*Last Name	*Suffix (eg, Jr, III)	Academic Degrees	Institution	Location (city, state/province, country)	Role or Contribution, eg, chair, principal investigator	Group (if more than 1 Group listed in the byline) and/or Subgroup (eg, Steering Committee)
Gexia	Liu	N/A	MD	Changge People's Hospital	Xuchang, Henan, China	Site Principal Investigator	Research Site Investigators
Yonghong	Tang	N/A	MD	Affiliated Nanhua Hospital, University of South China	Hengyang, Hunan, China	Site Principal Investigator	Research Site Investigators
Xiaomin	Mei	N/A	MD	Luoning People's Hospital	Luoyang, Henan, China	Site Principal Investigator	Research Site Investigators
Guoping	Zou	N/A	MD	Ningde People's Hospital	Ningde, Fujian, China	Site Principal Investigator	Research Site Investigators
Yuefeng	Yang	N/A	MD	Nanshi Hospital of Nanyang	Nanyang, Henan, China	Site Principal Investigator	Research Site Investigators
Quanhao	Li	N/A	MD	Gaomi People's Hospital	Weifang, Shandong, China	Site Principal Investigator	Research Site Investigators
Xiju	Tian	N/A	MD	Baoding NO.1 Hospital	Baoding, Hebei, China	Site Principal Investigator	Research Site Investigators
Hong	Chen	N/A	MD	The Central Hospital of Jiamusi	Jiamusi, Heilongjiang, China	Site Principal Investigator	Research Site Investigators
Jialiang	Xiao	N/A	MD	The Second People's Hospital of Xi	Xinyang, Henan, China	Site Principal Investigator	Research Site Investigators
Xiaoming	Song	N/A	MD	Mianchi Chinese Medical Hospital	Sanmenxia, Henan, China	Site Principal Investigator	Research Site Investigators
Guangning	Li	N/A	MD	Huadu District People's Hospital of Guangzhou	Guangzhou, Guangdong, China	Site Principal Investigator	Research Site Investigators
Guozhong	Li	N/A	MD	The First Hospital of Harbin Medical University	Harbin, Heilongjiang, China	Site Principal Investigator	Research Site Investigators
Chunjie	Yang	N/A	MD	Ningjin People's Hospital	Dezhou, Shandong, China	Site Principal Investigator	Research Site Investigators
Xiting	Zhang	N/A	MD	Liaocheng Central Hospital	Liaocheng, Shandong, China	Site Principal Investigator	Research Site Investigators
Chun	Wang	N/A	MD	The First people's Hospital of Ruzhou	Pingdingshan, Henan, China	Site Principal Investigator	Research Site Investigators
Jizheng	Hu	N/A	MD	Taikang People's Hospital	Zhoukou, Henan, China	Site Principal Investigator	Research Site Investigators
Wei	Hu	N/A	MD	Anshan Central Hospital	Anshan, Liaoning, China	Site Principal Investigator	Research Site Investigators

Supplemental Online Content: Nonauthor Collaborators
*First name, last name, and suffix (if applicable) are required and will appear in PubMed.

*First Name and Middle Initial(s)	*Last Name	*Suffix (eg, Jr, III)	Academic Degrees	Institution	Location (city, state/province, country)	Role or Contribution, eg, chair, principal investigator	Group (if more than 1 Group listed in the byline) and/or Subgroup (eg, Steering Committee)
Zhen	Jiao	N/A	MD	Anshan Central Hospital	Anshan, Liaoning, China	Site Principal Investigator	Research Site Investigators
Yunnan	Lu	N/A	MD	Wuxi Xishan People's Hospital	Wuxi, Jiangsu, China	Site Principal Investigator	Research Site Investigators
Zhangyong	Xia	N/A	MD	Liaocheng People's Hospital	Liaocheng, Shandong, China	Site Principal Investigator	Research Site Investigators
Yufen	Wang	N/A	MD	Heping Hospital affiliated to Changzhi Medical College	Changzhi, Shanxi, China	Site Principal Investigator	Research Site Investigators
Yinshan	Wang	N/A	MD	Xuchang Central Hospital	Xuchang, Henan, China	Site Principal Investigator	Research Site Investigators
Jinxing	Qi	N/A	MD	Anyang District Hospital	Anyang, Henan, China	Site Principal Investigator	Research Site Investigators
Xiaoping	Wang	N/A	MD	Jixi People's Hospital	Jixi, Heilongjiang, China	Site Principal Investigator	Research Site Investigators
Shuqin	Liu	N/A	MD	Jixi People's Hospital	Jixi, Heilongjiang, China	Site Principal Investigator	Research Site Investigators
Bo	Li	N/A	MD	Beijing Hepingli Hospital	Beijing, China	Site Principal Investigator	Research Site Investigators
Yinghui	Li	N/A	MD	Xuchang Hospital of Traditional Chinese Medicne	Xuchang, Henan, China	Site Principal Investigator	Research Site Investigators
Yaming	Sun	N/A	MD	Zhangjiagang Traditional Chinese Medicine Hospital	Suzhou, Jiangsu, China	Site Principal Investigator	Research Site Investigators
Ping	Jing	N/A	MD	Wuhan Central Hospital	Wuhan, Hubei, China	Site Principal Investigator	Research Site Investigators
Jialing	Wu	N/A	MD	Tianjin Huanhu Hospital	Tianjin, China	Site Principal Investigator	Research Site Investigators
Bo	Yang	N/A	MD	Luzhou Chinese Medical Hospital	Luzhou, Sichuan, China	Site Principal Investigator	Research Site Investigators
Jinghua	Zhang	N/A	MD	Zhecheng Chinese Medical Hospital	Shangqiu, Henan, China	Site Principal Investigator	Research Site Investigators
Tianxia	Zhang	N/A	MD	Xun People's Hospital	Hebi, Henan, China	Site Principal Investigator	Research Site Investigators
Chunling	Zheng	N/A	MD	Luohe Central Hospital	Luohe, Henan, China	Site Principal Investigator	Research Site Investigators

Supplemental Online Content: Nonauthor Collaborators
*First name, last name, and suffix (if applicable) are required and will appear in PubMed.

*First Name and Middle Initial(s)	*Last Name	*Suffix (eg, Jr, III)	Academic Degrees	Institution	Location (city, state/province, country)	Role or Contribution, eg, chair, principal investigator	Group (if more than 1 Group listed in the byline) and/or Subgroup (eg, Steering Committee)
Lejun	Li	N/A	MD	Wuxi Affiliated Hospital of Nanjing University of Chinese Medicine	Wuxi, Jiangsu, China	Site Principal Investigator	Research Site Investigators
Huimin	Li	N/A	MD	The Third People's Hospital of Luohe	Luohe, Henan, China	Site Principal Investigator	Research Site Investigators
Yunnan	Lu	N/A	MD	The Second People's Hospital of Wuxi	Wuxi, Jiangsu, China	Site Principal Investigator	Research Site Investigators
Fumin	Yu	N/A	MD	The Second People's Hospital of Mudanjiang	Mudanjiang, Heilongjiang, China	Site Principal Investigator	Research Site Investigators
Shufang	Yao	N/A	MD	Sanmenxia Central Hospital	Sanmenxia, Henan, China	Site Principal Investigator	Research Site Investigators
Jianjun	Chang	N/A	MD	Xinyang Central Hospital	Xinyang, Henan, China	Site Principal Investigator	Research Site Investigators
Dongqun	Li	N/A	MD	Yingkou Central Hospital	Yingkou, Liaoning, China	Site Principal Investigator	Research Site Investigators
Ganqin	Du	N/A	MD	The first Affiliated Hospital of Henan University of Science and Technology	Luoyang, Henan, China	Site Principal Investigator	Research Site Investigators
Yi	Zhao	N/A	MD	Tongzhou District 8th People's Hospital of Nantong City	Nantong, Jiangsu, China	Site Principal Investigator	Research Site Investigators
Pengfei	Liang	N/A	MD	Pingyao People's Hospital	Jinzhong, Shanxi, China	Site Principal Investigator	Research Site Investigators
Ming	Wang	N/A	MD	Dehong Prefecture People's Hospital	Dehong, Yunnan, China	Site Principal Investigator	Research Site Investigators
Qi	Fang	N/A	MD	The First Affiliated Hospital of Soochow University	Suzhou, Jiangsu, China	Site Principal Investigator	Research Site Investigators
Youqing	Deng	N/A	MD	The First Hospital of Nanchang	Nanchang, Jiangxi, China	Site Principal Investigator	Research Site Investigators
Xuzhao	Gao	N/A	MD	Xinxiang Central Hospital	Xinxiang, Henan, China	Site Principal Investigator	Research Site Investigators
Runxiu	Zhu	N/A	MD	Inner Mongolia People's Hospital	Hohhot, Inner Mongolia, China	Site Principal Investigator	Research Site Investigators
Yimin	Xie	N/A	MD	Zhangjiakou Xuangang Hospital	Zhangjiakou, Hebei, China	Site Principal Investigator	Research Site Investigators
Yanshu	Liu	N/A	MD	Anyang People's Hospital	Anyang, Henan, China	Site Principal Investigator	Research Site Investigators

Supplemental Online Content: Nonauthor Collaborators
*First name, last name, and suffix (if applicable) are required and will appear in PubMed.

*First Name and Middle Initial(s)	*Last Name	*Suffix (eg, Jr, III)	Academic Degrees	Institution	Location (city, state/province, country)	Role or Contribution, eg, chair, principal investigator	Group (if more than 1 Group listed in the byline) and/or Subgroup (eg, Steering Committee)
Yi	Yuan	N/A	MD	The Third Xiangya Hospital of Central South University	Changsha, Hunan, China	Site Principal Investigator	Research Site Investigators
Qiuyi	Wu	N/A	MD	The First People's Hospital of Zhangjiagang	Suzhou, Jiangsu, China	Site Principal Investigator	Research Site Investigators
Guimei	Zhao	N/A	MD	General Hospital of Angang Group Company	Anshan, Liaoning, China	Site Principal Investigator	Research Site Investigators
Yan	Yang	N/A	MD	Qi People's Hospital	Hebi, Henan, China	Site Principal Investigator	Research Site Investigators
Yong	Lu	N/A	MD	Xi People's Hospital	Xinyang, Henan, China	Site Principal Investigator	Research Site Investigators
Weidong	Zhao	N/A	MD	Mianchi People's Hospital	Sanmenxia, Henan, China	Site Principal Investigator	Research Site Investigators
Tianbao	Chen	N/A	MD	Xingyang People's Hospital	Zhengzhou, Henan, China	Site Principal Investigator	Research Site Investigators
Deng	Pan	N/A	MD	The First people's Hospital of Xinxiang	Xinxiang, Henan, China	Site Principal Investigator	Research Site Investigators
Min	Yang	N/A	MD	The Fourth People's Hospital of Xinyang	Xinyang, Henan, China	Site Principal Investigator	Research Site Investigators
Baoguo	Xue	N/A	MD	Mengzhou Chinese Medical Hospital	Jiaozuo, Henan, China	Site Principal Investigator	Research Site Investigators
Ge	Zhang	N/A	MD	Luoyang Dongfang Hospital	Luoyang, Henan, China	Site Principal Investigator	Research Site Investigators
Yanjiang	Zhao	N/A	MD	Suiping Renan Hospital	Zhumadian, Henan, China	Site Principal Investigator	Research Site Investigators
Yunfei	Wei	N/A	MD	The Second Affiliated Hospital of Guangxi Medical University	Nanning, Guangxi, China	Site Principal Investigator	Research Site Investigators
Wenwei	Yun	N/A	MD	The Second People's Hospital of Changzhou	Changzhou, Jiangsu, China	Site Principal Investigator	Research Site Investigators
Xiaoqi	Chen	N/A	MD	Huangzhou District People's Hospital	Huanggang, Hubei, China	Site Principal Investigator	Research Site Investigators
Danhong	Wu	N/A	MD	The Fifth People's Hospital of Shanghai	Shanghai, China	Site Principal Investigator	Research Site Investigators
Lifang	Zhang	N/A	MD	Changzhi People's Hospital	Changzhi, Shanxi, China	Site Principal Investigator	Research Site Investigators

Supplemental Online Content: Nonauthor Collaborators
*First name, last name, and suffix (if applicable) are required and will appear in PubMed.

*First Name and Middle Initial(s)	*Last Name	*Suffix (eg, Jr, III)	Academic Degrees	Institution	Location (city, state/province, country)	Role or Contribution, eg, chair, principal investigator	Group (if more than 1 Group listed in the byline) and/or Subgroup (eg, Steering Committee)
Baoying	Sheng	N/A	MD	The First Affiliated Hospital of Jiamusi University	Jiamusi, Heilongjiang, China	Site Principal Investigator	Research Site Investigators
Zhigang	Cui	N/A	MD	The Third People's Hospital of Datong	Datong, Shanxi, China	Site Principal Investigator	Research Site Investigators
Xiangdong	Xie	N/A	MD	The Second People's Hospital of Jiaozuo	Jiaozuo, Henan, China	Site Principal Investigator	Research Site Investigators
Guanghui	Cheng	N/A	MD	Kangping People's Hospital	Shenyang, Liaoning, China	Site Principal Investigator	Research Site Investigators
Yifei	Zhang	N/A	MD	The First People's Hospital of Lingbao	Sanmenxia, Henan, China	Site Principal Investigator	Research Site Investigators
Ruiming	Zhu	N/A	MD	Henan Shenhuo Group staff General Hospital	Shangqiu, Henan, China	Site Principal Investigator	Research Site Investigators
Yong	Chen	N/A	MD	Ningbo Medical Center Li Huili Hospital	Ningbo, Zhejiang, China	Site Principal Investigator	Research Site Investigators
GuoHua	Liu	N/A	MD	Cangzhou Hospital of Integrated TCM-WM Hebei	Cangzhou, Hebei, China	Site Principal Investigator	Research Site Investigators
Lei	Feng	N/A	MD	Zaozhuang Mining Group Zaozhuang Hospital	Zaozhuang, Shandong, China	Site Principal Investigator	Research Site Investigators
Zhihua	Long	N/A	MD	Zhumadian Central Hospital	Zhumadian, Henan, China	Site Principal Investigator	Research Site Investigators
Huisheng	Chen	N/A	MD	Northern Theater General Hospital	Shenyang, Liaoning, China	Site Principal Investigator	Research Site Investigators
Ping	Zhang	N/A	MD	The First Affiliated Hospital of Xinxiang Medical College	Xinxiang, Henan, China	Site Principal Investigator	Research Site Investigators
Yuanliang	Cui	N/A	MD	Tanghe People's Hospital	Nanyang, Henan, China	Site Principal Investigator	Research Site Investigators
Yongli	Zhang	N/A	MD	Wenxian People's Hospital	Jiaozuo, Henan, China	Site Principal Investigator	Research Site Investigators
Yazhou	Han	N/A	MD	Qinyang People's Hospital	Jiaozuo, Henan, China	Site Principal Investigator	Research Site Investigators
Yajie	Bai	N/A	MD	Qinyang People's Hospital	Jiaozuo, Henan, China	Site Principal Investigator	Research Site Investigators
Tieyu	Tang	N/A	MD	Affiliated Hospital of Yangzhou University	Yangzhou, Jiangsu, China	Site Principal Investigator	Research Site Investigators

*First name, last name, and suffix (if applicable) are required and will appear in PubMed.

*First Name and Middle Initial(s)	*Last Name	*Suffix (eg, Jr, III)	Academic Degrees	Institution	Location (city, state/province, country)	Role or Contribution, eg, chair, principal investigator	Group (if more than 1 Group listed in the byline) and/or Subgroup (eg, Steering Committee)
Songdi	Wu	N/A	MD	Xi'an First Hospital	Xi'an, Shaanxi, China	Site Principal Investigator	Research Site Investigators
Wenping	Gong	N/A	MD	Shengzhou People's Hospital	Shaoxing, Zhejiang, China	Site Principal Investigator	Research Site Investigators
Jun	Wang	N/A	MD	Nanxishan Hospital of Guangxi Zhuang Autonomous Region	Guilin, Guangxi, China	Site Principal Investigator	Research Site Investigators
Zhishan	Zhu	N/A	MD	Shenzhen Luohu People's Hospital	Shenzhen, Guangdong, China	Site Principal Investigator	Research Site Investigators
Xiaoyan	Ma	N/A	MD	The Second People's Hospital of Mengjin	Luoyang, Henan, China	Site Principal Investigator	Research Site Investigators
Leyi	Yao	N/A	MD	Baofeng People's Hospital	Pingdingshan, Henan, China	Site Principal Investigator	Research Site Investigators
Runhui	Li	N/A	MD	The Affiliated Central Hospital of Shenyang Medical College	Shenyang, Liaoning, China	Site Principal Investigator	Research Site Investigators
Shuanggen	Zhu	N/A	MD	Shenzhen Longhua District People's Hospital	Shenzhen, Guangdong, China	Site Principal Investigator	Research Site Investigators
Juntao	Li	N/A	MD	Handan Central Hospital	Handan, Hebei, China	Site Principal Investigator	Research Site Investigators
Xiuhui	Qi	N/A	MD	Jilin Electric Power Hospital	Changchun, Jilin, China	Site Principal Investigator	Research Site Investigators
Zhongping	Jiang	N/A	MD	Dongying District People's Hospital	Dongying, Shandong, China	Site Principal Investigator	Research Site Investigators
Zhigang	Liang	N/A	MD	Yantai Yuhuangding Hospital	Yantai, Shandong, China	Site Principal Investigator	Research Site Investigators
Chunping	Liu	N/A	MD	Dazhou Central Hospital	Dazhou, Sichuan, China	Site Principal Investigator	Research Site Investigators
Dongjuan	Xu	N/A	MD	Dongyang People's Hospital	Jinhua, Zhejiang, China	Site Principal Investigator	Research Site Investigators
Tao	Qiu	N/A	MD	The First People's Hospital of Zigong	Zigong, Sichuan, China	Site Principal Investigator	Research Site Investigators
Chunping	Chen	N/A	MD	Linfen People's Hospital	Linfen, Shanxi, China	Site Principal Investigator	Research Site Investigators
Hong	Tan	N/A	MD	First hospital of Changsha city	Changsha, Hunan, China	Site Principal Investigator	Research Site Investigators

Supplemental Online Content: Nonauthor Collaborators
*First name, last name, and suffix (if applicable) are required and will appear in PubMed.

*First Name and Middle Initial(s)	*Last Name	*Suffix (eg, Jr, III)	Academic Degrees	Institution	Location (city, state/province, country)	Role or Contribution, eg, chair, principal investigator	Group (if more than 1 Group listed in the byline) and/or Subgroup (eg, Steering Committee)
Shengli	Chen	N/A	MD	Chongqing Three Gorges Central Hospital	Chongqing, China	Site Principal Investigator	Research Site Investigators
Chunshui	Yang	N/A	MD	Shenzhen Nanshan District People's Hospital	Shenzhen, Guangdong, China	Site Principal Investigator	Research Site Investigators
Wei	Jun	N/A	MD	The First People's Hospital of Yibin	Yibin, Sichuan, China	Site Principal Investigator	Research Site Investigators
Qing	He	N/A	MD	Xuzhou NO.1 People's Hospital	Xuzhou, Jiangsu, China	Site Principal Investigator	Research Site Investigators
Zengqiang	Sun	N/A	MD	Zibo Municipal Hospital	Zibo, Shandong, China	Site Principal Investigator	Research Site Investigators
Shen	Li	N/A	MD	Dalian Central Hospital	Dalian, Liaoning, China	Site Principal Investigator	Research Site Investigators
Cunju	Guo	N/A	MD	Liaocheng People's Hospital	Liaocheng, Shandong, China	Site Principal Investigator	Research Site Investigators
Hongliang	Wang	N/A	MD	The Sixth People's Hospital of Nantong	Nantong, Jiangsu, China	Site Principal Investigator	Research Site Investigators
Dongfang	Li	N/A	MD	Second Hospital of Shanxi Medical University	Taiyuan, Shanxi, China	Site Principal Investigator	Research Site Investigators
Hongbin	Wu	N/A	MD	General Hospital of Shanxi Lu'an Mining (Group) Co., Ltd	Changzhi, Shanxi, China	Site Principal Investigator	Research Site Investigators
Wenxu	Zhen	N/A	MD	Dalian Friendship Hospital	Dalian, Liaoning, China	Site Principal Investigator	Research Site Investigators
Lijun	Wang	N/A	MD	The Fourth Central Hospital of Tianjin	Tianjin, China	Site Principal Investigator	Research Site Investigators
Wenke	Hong	N/A	MD	The Second Hospital of Ningbo	Ningbo, Zhejiang, China	Site Principal Investigator	Research Site Investigators
Xuerong	Qiu	N/A	MD	The First Hospital of Qiqihar	Qiqihar, Heilongjiang, China	Site Principal Investigator	Research Site Investigators
Xinxia	Wei	N/A	MD	Yellow River Central Hospital of Yellow River Water Conservancy Commission	Zhengzhou, Henan, China	Site Principal Investigator	Research Site Investigators
Yongtao	Lv	N/A	MD	Shandong Province Third Hospital	Jinan, Shandong, China	Site Principal Investigator	Research Site Investigators

Supplemental Online Content: Nonauthor Collaborators
*First name, last name, and suffix (if applicable) are required and will appear in PubMed.

*First Name and Middle Initial(s)	*Last Name	*Suffix (eg, Jr, III)	Academic Degrees	Institution	Location (city, state/province, country)	Role or Contribution, eg, chair, principal investigator	Group (if more than 1 Group listed in the byline) and/or Subgroup (eg, Steering Committee)
Xiangyang	Feng	N/A	MD	Xihua People's Hospital	Zhoukou, Henan, China	Site Principal Investigator	Research Site Investigators
Gaiqing	Yang	N/A	MD	Zhengzhou Central Hospital	Zhengzhou, Henan, China	Site Principal Investigator	Research Site Investigators
Songjun	Lin	N/A	MD	Shenzhen Traditional Chinese Medicine Hospital	Shenzhen, Guangdong, China	Site Principal Investigator	Research Site Investigators
Aihua	Cao	N/A	MD	The Second Affiliated Hospital of Luohe Medical College	Luohe, Henan, China	Site Principal Investigator	Research Site Investigators
Dejin	Sun	N/A	MD	The Third People's Hospital of Shenzhen	Shenzhen, Guangdong, China	Site Principal Investigator	Research Site Investigators
Pengcheng	Fu	N/A	MD	Shenzhen Longhua District Central Hospital	Shenzhen, Guangdong, China	Site Principal Investigator	Research Site Investigators
Cao	Cao	N/A	MD	Jiangxi People's Hospital	Nanchang, Jiangxi, China	Site Principal Investigator	Research Site Investigators
Wenjun	Xue	N/A	MD	The First People's Hospital of Pingdingshan	Pingdingshan, Henan, China	Site Principal Investigator	Research Site Investigators
Haiyan	Liu	N/A	MD	Jilin People's Hospital	Jilin, Jilin, China	Site Principal Investigator	Research Site Investigators
Shanshan	Li	N/A	MD	Jilin People's Hospital	Jilin, Jilin, China	Site Principal Investigator	Research Site Investigators
Fang	Qu	N/A	MD	The Second People's Hospital of Dalian	Dalian, Liaoning, China	Site Principal Investigator	Research Site Investigators
Zhengguo	Zhou	N/A	MD	The Second People's Hospital of Dalian	Dalian, Liaoning, China	Site Principal Investigator	Research Site Investigators
Ping	Liu	N/A	MD	Dali Bai Autonomous Prefecture People's Hospital	Dali, Yunnan, China	Site Principal Investigator	Research Site Investigators
Lixia	Wang	N/A	MD	Tieling Central Hospital	Tieling, Liaoning, China	Site Principal Investigator	Research Site Investigators
Yiping	Wu	N/A	MD	The First Hospital of Handan	Handan, Hebei, China	Site Principal Investigator	Research Site Investigators
Jinxing	Liu	N/A	MD	Qingyun People's Hospital	Dezhou, Shandong, China	Site Principal Investigator	Research Site Investigators
Xin	Sun	N/A	MD	The First Hospital Of Jilin University	Changchun, Jilin, China	Site Principal Investigator	Research Site Investigators

Supplemental Online Content: Nonauthor Collaborators
*First name, last name, and suffix (if applicable) are required and will appear in PubMed.

*First Name and Middle Initial(s)	*Last Name	*Suffix (eg, Jr, III)	Academic Degrees	Institution	Location (city, state/province, country)	Role or Contribution, eg, chair, principal investigator	Group (if more than 1 Group listed in the byline) and/or Subgroup (eg, Steering Committee)
Qingyong	Wang	N/A	MD	University of Chinese Academy of Sciences Shenzhen Hospital	Shenzhen, Guangdong, China	Site Principal Investigator	Research Site Investigators
Xiaomei	Li	N/A	MD	Shenzhen Longgang District People's Hospital	Shenzhen, Guangdong, China	Site Principal Investigator	Research Site Investigators
Qizhang	Wang	N/A	MD	Shenzhen Bao'an District Shajing People's Hospital	Shenzhen, Guangdong, China	Site Principal Investigator	Research Site Investigators
Yongxiong	Wu	N/A	MD	Beijing University of Chinese Medicine Shenzhen Hospital	Shenzhen, Guangdong, China	Site Principal Investigator	Research Site Investigators
Chunfeng	Liu	N/A	MD	The Second Affiliated Hospital of Soochow University	Suzhou, Jiangsu, China	Site Principal Investigator	Research Site Investigators
Yanxin	Zhao	N/A	MD	Shanghai Tenth People's Hospital	Shanghai, China	Site Principal Investigator	Research Site Investigators
Zhijian	Lin	N/A	MD	Peking University Shenzhen Hospital	Shenzhen, Guangdong, China	Site Principal Investigator	Research Site Investigators
Rui	Ma	N/A	MD	Nanyang Zhangzhongjing Hospital	Nanyang, Henan, China	Site Principal Investigator	Research Site Investigators
Jiedan	Li	N/A	MD	Nanyang Yuxi Union Hospital	Nanyang, Henan, China	Site Principal Investigator	Research Site Investigators
Zhishun	Zhu	N/A	MD	Sheqi People's Hospital	Nanyang, Henan, China	Site Principal Investigator	Research Site Investigators
Lili	Guo	N/A	MD	The Sixth People's Hospital of Luoyang	Luoyang, Henan, China	Site Principal Investigator	Research Site Investigators
Bing	Sun	N/A	MD	The Third People's Hospital of Luoyang	Luoyang, Henan, China	Site Principal Investigator	Research Site Investigators
Jun	Tan	N/A	MD	The Third Affiliated Hospital of Xinxiang Medical College	Xinxiang, Henan, China	Site Principal Investigator	Research Site Investigators
Ke	Yang	N/A	MD	Henan Hongli Hospital	Xinxiang, Henan, China	Site Principal Investigator	Research Site Investigators
Yong	Bi	N/A	MD	The Fourth People's Hospital of Shanghai	Shanghai, China	Site Principal Investigator	Research Site Investigators
Yingjie	Duan	N/A	MD	Liaoning Health Industry Group Fuxin Mine General Hospital	Fuxin, Liaoning, China	Site Principal Investigator	Research Site Investigators
Shaochun	Li	N/A	MD	The First People's Hospital of Yuanping	Yuanping, Shanxi, China	Site Principal Investigator	Research Site Investigators

Supplemental Online Content: Nonauthor Collaborators
*First name, last name, and suffix (if applicable) are required and will appear in PubMed.

***First Name and Middle Initial(s)**	***Last Name**	***Suffix (eg, Jr, III)**	**Academic Degrees**	**Institution**	**Location (city, state/province, country)**	**Role or Contribution, eg, chair, principal investigator**	**Group (if more than 1 Group listed in the byline) and/or Subgroup (eg, Steering Committee)**
Xiaoliang	Lou	N/A	MD	The Fourth Affiliated Hospital of Nanchang University	Nanchang, Jiangxi, China	Site Principal Investigator	Research Site Investigators
Xiaosong	Huang	N/A	MD	Hunan Brain Hospital	Changsha, Hunan, China	Site Principal Investigator	Research Site Investigators
Fucai	Zang	N/A	MD	Xinhua Hospital affiliated to Dalian University	Dalian, Liaoning, China	Site Principal Investigator	Research Site Investigators
Yonghua	Dong	N/A	MD	Runan People's Hospital	Zhumadian, Henan, China	Site Principal Investigator	Research Site Investigators
Jingyan	Zhao	N/A	MD	Sui People's Hospital	Shangqiu, Henan, China	Site Principal Investigator	Research Site Investigators
Bin	Li	N/A	MD	The First people's Hospital of Xiangcheng	Zhoukou, Henan, China	Site Principal Investigator	Research Site Investigators
Yanzeng	Cui	N/A	MD	Gongyi People's Hospital	Zhengzhou, Henan, China	Site Principal Investigator	Research Site Investigators
Zili	Zhang	N/A	MD	Luyi Zhenyuan Hospital	Zhoukou, Henan, China	Site Principal Investigator	Research Site Investigators
Yali	Zhang	N/A	MD	Xinye People's Hospital	Nanyang, Henan, China	Site Principal Investigator	Research Site Investigators
Peng	Yan	N/A	MD	Yongcheng People's Hospital	Shangqiu, Henan, China	Site Principal Investigator	Research Site Investigators
Huixian	Fan	N/A	MD	Dengfeng People's Hospital	Zhengzhou, Henan, China	Site Principal Investigator	Research Site Investigators
Qiuhong	Ji	N/A	MD	Affiliated Hospital of Nantong University	Nantong, Jiangsu, China	Site Principal Investigator	Research Site Investigators
Aisheng	Wu	N/A	MD	The Forth People's Hospital of Hengsshui	Hengshui, Hebei, China	Site Principal Investigator	Research Site Investigators
Xinshen	Han	N/A	MD	Kaifeng Central Hospital	Kaifeng, Henan, China	Site Principal Investigator	Research Site Investigators
Li	Liu	N/A	MM	Beijing Tiantan Hospital, Capital Medical University	Beijing, China	Research Assistant	Clinical Coordinating Center
Jie	Song	N/A	MM	Beijing Tiantan Hospital, Capital Medical University	Beijing, China	Research Assistant	Clinical Coordinating Center
Shuting	Liu	N/A	BM	Beijing Tiantan Hospital, Capital Medical University	Beijing, China	Research Assistant	Clinical Coordinating Center

*First Name and Middle Initial(s)	*Last Name	*Suffix (eg, Jr, III)	Academic Degrees	Institution	Location (city, state/province, country)	Role or Contribution, eg, chair, principal investigator	Group (if more than 1 Group listed in the byline) and/or Subgroup (eg, Steering Committee)
Xiaoyu	Che	N/A	BM	Beijing Tiantan Hospital, Capital Medical University	Beijing, China	Research Assistant	Clinical Coordinating Center
Xianhong	Liang	N/A	BM	Beijing Tiantan Hospital, Capital Medical University	Beijing, China	Research Assistant	Clinical Coordinating Center
Shangzhi	Li	N/A	BM	Beijing Tiantan Hospital, Capital Medical University	Beijing, China	Research Assistant	Clinical Coordinating Center
Nan	Qi	N/A	BM	Beijing Tiantan Hospital, Capital Medical University	Beijing, China	Research Assistant	Clinical Coordinating Center
Xiaolei	Chen	N/A	BM	Beijing Tiantan Hospital, Capital Medical University	Beijing, China	Research Assistant	Clinical Coordinating Center
Chenhui	Liu	N/A	MM	Beijing Tiantan Hospital, Capital Medical University	Beijing, China	Research Assistant	Clinical Coordinating Center
Jingtao	Pi	N/A	MM	Beijing Tiantan Hospital, Capital Medical University	Beijing, China	Research Assistant	Clinical Coordinating Center
Yu	Tian	N/A	MM	Beijing Tiantan Hospital, Capital Medical University	Beijing, China	Research Assistant	Clinical Coordinating Center
Nan	Wang	N/A	MM	Beijing Tiantan Hospital, Capital Medical University	Beijing, China	Research Assistant	Clinical Coordinating Center
Zhengyang	Li	N/A	MM	Beijing Tiantan Hospital, Capital Medical University	Beijing, China	Research Assistant	Clinical Coordinating Center
Biyang	Luo	N/A	MM	Beijing Tiantan Hospital, Capital Medical University	Beijing, China	Research Assistant	Clinical Coordinating Center
Fanfang	Yue	N/A	MM	Beijing Tiantan Hospital, Capital Medical University	Beijing, China	Research Assistant	Clinical Coordinating Center
Lei	Guo	N/A	MM	Beijing Tiantan Hospital, Capital Medical University	Beijing, China	Research Assistant	Clinical Coordinating Center
Kun	Hu	N/A	MM	Beijing Tiantan Hospital, Capital Medical University	Beijing, China	Research Assistant	Clinical Coordinating Center
Xi	Zhong	N/A	MM	Beijing Tiantan Hospital, Capital Medical University	Beijing, China	Research Assistant	Clinical Coordinating Center
Jiawei	Lu	N/A	BM	Beijing Tiantan Hospital, Capital Medical University	Beijing, China	Research Assistant	Clinical Coordinating Center
Long	Wang	N/A	BM	Beijing Tiantan Hospital, Capital Medical University	Beijing, China	Research Assistant	Clinical Coordinating Center

Supplemental Online Content: Nonauthor Collaborators
*First name, last name, and suffix (if applicable) are required and will appear in PubMed.

*First Name and Middle Initial(s)	*Last Name	*Suffix (eg, Jr, III)	Academic Degrees	Institution	Location (city, state/province, country)	Role or Contribution, eg, chair, principal investigator	Group (if more than 1 Group listed in the byline) and/or Subgroup (eg, Steering Committee)
Jiandong	Yu	N/A	BM	Beijing Tiantan Hospital, Capital Medical University	Beijing, China	Research Assistant	Clinical Coordinating Center
Xiaowu	Zhang	N/A	BM	Beijing Tiantan Hospital, Capital Medical University	Beijing, China	Research Assistant	Clinical Coordinating Center
Mengyuan	Zhou	N/A	MD	Beijing Tiantan Hospital, Capital Medical University	Beijing, China	Research Assistant	Clinical Coordinating Center
Yiyi	Chen	N/A	MD	Beijing Tiantan Hospital, Capital Medical University	Beijing, China	Research Assistant	Clinical Coordinating Center
Zhiyuan	Ji	N/A	BM	Nanjing Service Medical Technology Co.,LTD	Beijing, China	Clinical Research Coordinator Manager	Clinical Coordinating Center (SMO)
Jianying	Li	N/A	BM	Beijing Tianrunjinghua Medical Technology Co.,LTD	Beijing, China	Clinical Research Coordinator Manager	Clinical Coordinating Center (SMO)
Hongyi	Yan	N/A	MD	Beijing Tiantan Hospital, Capital Medical University	Beijing, China	Statistician	Statistical and Data Management Center
Mengxing	Wang	N/A	MD	Beijing Tiantan Hospital, Capital Medical University	Beijing, China	Statistician	Statistical and Data Management Center
Haibo	Wu	N/A	MD	Giant Med-Pharma Services (BeiJing), INC	Beijing, China	Clinical Research Associate Manager	Independent Medical Monitor (CRO)
