# Supplementary material for: Immediate- or Delayed-Intensive Statin in Acute Cerebral Ischemia: The INSPIRES Randomized Clinical Trial
Source: JAMA Neurol. 2024 May 28;81(7):741–51. doi: 10.1001/jamaneurol.2024.1433 (PMC11134282; doi:10.1001/jamaneurol.2024.1433)
Supplement: Supplement 5. — Data Sharing Statement. [file jamaneurol-e241433-s005.pdf]

Data Sharing Statement

Gao. Immediate- or Delayed-Intensive Statin in Acute Cerebral Ischemia. *JAMA Neurol*. Published May 28, 2024. doi:10.1001/jamaneurol.2024.1433

Data
Data available: No
